# Studies towards the Synthesis of (+)‐Dictyoxetane

**DOI:** 10.1002/chem.202202429

**Published:** 2022-10-27

**Authors:** Joseph Benford‐Ward, Sanaz Ahmadipour, Aliya Sembayeva, Louise Male, Richard S. Grainger

**Affiliations:** ^1^ School of Chemistry University of Birmingham Edgbaston Birmingham B15 2TT UK

**Keywords:** cyclization, cycloaddition, diastereoselectivity, natural products, oxygen heterocycles

## Abstract

The dolabellane‐type diterpene dictyoxetane represents a significant challenge to synthetic organic chemistry. Methodology directed towards the total synthesis of naturally occurring (+)‐dictyoxetane is reported. Catalytic asymmetric synthesis of the *trans*‐hydrindane ring system is achieved through chemoselective deoxygenation of the Hajos‐Parrish ketone. An alternative to the Garst‐Spencer furan annulation is developed for the synthesis of a 2,5‐dimethyl, tetrasubstituted furan, employing a tandem 5‐*exo*‐dig alcohol to alkyne cyclisation/aromatisation reaction as a key step. The (4+3) cycloaddition reaction of an oxyallyl cation with a tetrasubstituted furan is established on a cyclohexanone‐derived model system, and a range of related (4+3) cycloadditions investigated on a homochiral, *trans*‐hydrindane‐fused furan, where regio‐ and diastereoselectivity is required for the natural product synthesis. In an alternative (4+2) Diels‐Alder approach, a C_2_‐symmetric vinyl sulfoxide‐based chiral ketene equivalent is used to prepare oxanorbornenes with the same oxygen bridge stereochemistry found in the 2,7‐dioxatricyclo[4.2.1.0^3,8^]nonane ring system of the natural product.

## Graphical abstract

**The Hajos‐Parrish ketone** is used to prepare a key building block for the total synthesis of the marine natural product dictyoxetane in enantiopure form. A synthetic alternative to the Garst‐Spencer furan annelation is developed, and the reactivity and diastereoselectivity of the resulting tetrasubstituted chiral furan in cycloaddition reactions towards an appropriately functionalised carbocyclic core of dictyoxetane are explored.

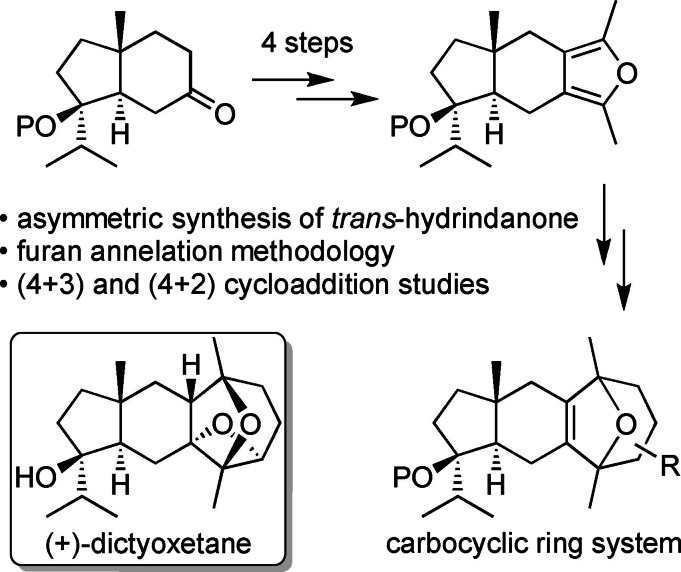

## Introduction

**Scheme 1:**
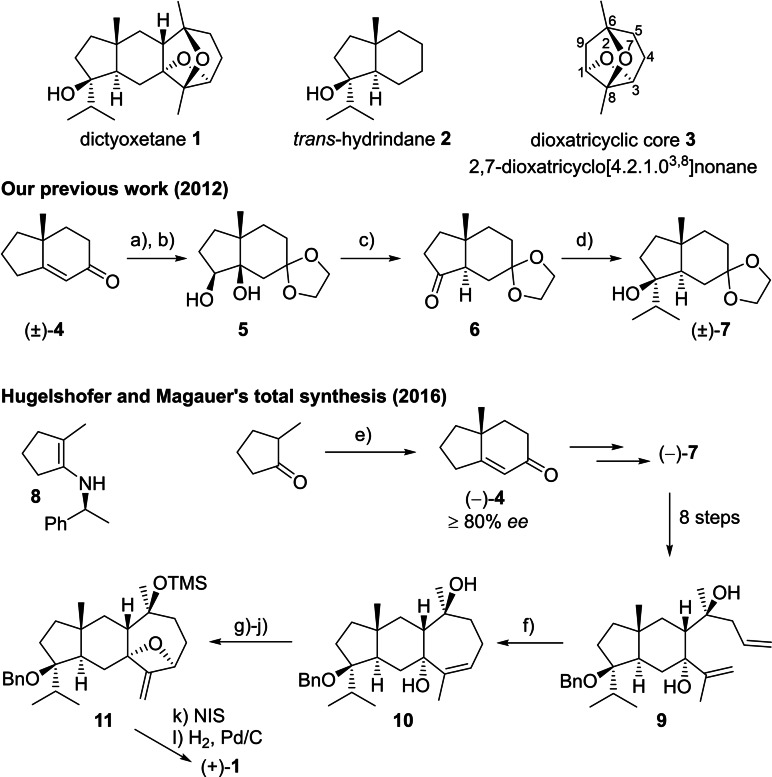
Structure of (+)‐dictyoxetane **1** and core ring systems, our previously reported synthesis of racemic *trans*‐hydrindane **7**, and application in Hugelshofer and Magauer's asymmetric synthesis of dictyoxetane. Conditions: **a)** ethylene glycol, *p*TSA, toluene, reflux under Dean‐Stark; **b)** OsO_4_, NMO, H_2_O, THF, ^*t*^BuOH, 54 % (over 2 steps); **c)** PPh_3_, C_2_Cl_6_, ^*i*^Pr_2_NEt, MeCN, 0→82 °C, 80 %; **d)** CeCl_3_, ^*i*^PrMgCl, THF, 23 °C, 91 %; **e)** i) (*S*)‐(‐)‐α‐methylbenzylamine, *p*TSA, toluene, reflux under Dean‐Stark, then methyl vinyl ketone, 40 °C, then AcOH, H_2_O, 23 °C; ii) KOH, EtOH, 78 °C, 49 % (over 2 steps); **f)** Stewart‐Grubbs cat. (25 mol %), 2,6‐dichloro‐1,4‐benzoquinone, toluene, 111 °C, 55 %, 25 % recovered **9**; **g)** TMSI, CH_2_Cl_2_, 0→23 °C, 92 %; **h)** O_2_, hυ, TPP, DCE, 0 °C; PPh_3_, 23 °C, 71 %; **i)** MsCl, NEt_3_, CH_2_Cl_2_, −78 °C; **j)** NaH, THF, 66 °C, 88 % (over 2 steps); **k)** NIS, CH_2_Cl_2_, 23 °C; **l)** H_2_, Pd/C, THF, 23 °C, 80 % (over 2 steps); *p*TSA=*para*‐toluenesulfonic acid, NMO=*N*‐methylmorpholine‐*N*‐oxide, TPP=tetraphenylporphyrin, Ms=methanesulfonyl, NIS=*N*‐iodosuccinimide.

Dictyoxetane **1**, a dolabellane‐type diterpene isolated in 1985 from the brown alga *Dictyota dichotoma*,[^1^, ^2^] is one of the few natural products containing an oxetane ring (Scheme 1). The structure is comprised of *trans*‐hydrindane **2** fused to the 2,7‐dioxatricyclo[4.2.1.0^3,8^]nonane ring system **3**, so far unique in nature. Although the biological activity of dictyoxetane is unknown, the Hoffmann group has prepared several model derivatives of the dioxatricyclic core which show promising anticancer activity.^[3]^

In 2012, we reported a synthesis of the *trans*‐hydrindane ring system **2** of dictyoxetane.^[4]^ Racemic **7** was prepared in 4 steps from enone (±)‐**4**, the Robinson annulation product of 2‐methyl cyclopentanone with methyl vinyl ketone (Scheme 1). A phosphorus‐mediated pinacol‐like rearrangement of diol **5** was used to establish the requisite *trans*‐ring junction stereochemistry.[^5^, ^6^, ^7^] Our methodology was subsequently adapted by Hugelshofer and Magauer to the asymmetric total synthesis of (+)‐dictyoxetane using the chiral auxiliary approach developed by d'Angelo et al.[^8^, ^9^] Enone (‐)‐**4**, derived from enamine **8**, was estimated to be of ≥80 % *ee* by comparison of optical rotation with literature values. Scalemic tertiary alcohol (‐)‐**7** was converted in a further 8 steps to diene **9**, which allowed for 7‐membered ring annulation through a challenging ring‐closing metathesis reaction to form trisubstituted alkene **10**. The late‐stage formation of the 2,7‐dioxatricyclo[4.2.1.0^3,8^]nonane ring system was achieved through sequential 4‐*exo‐*tet cyclisation to form oxetane **11**, followed by a transannular 5‐*exo‐*trig cyclisation to form the tetrahydrofuran ring. In addition to being the first, and to date only, total synthesis of dictyoxetane, this work established the absolute stereochemistry of the natural product for the first time.

**Scheme 2:**
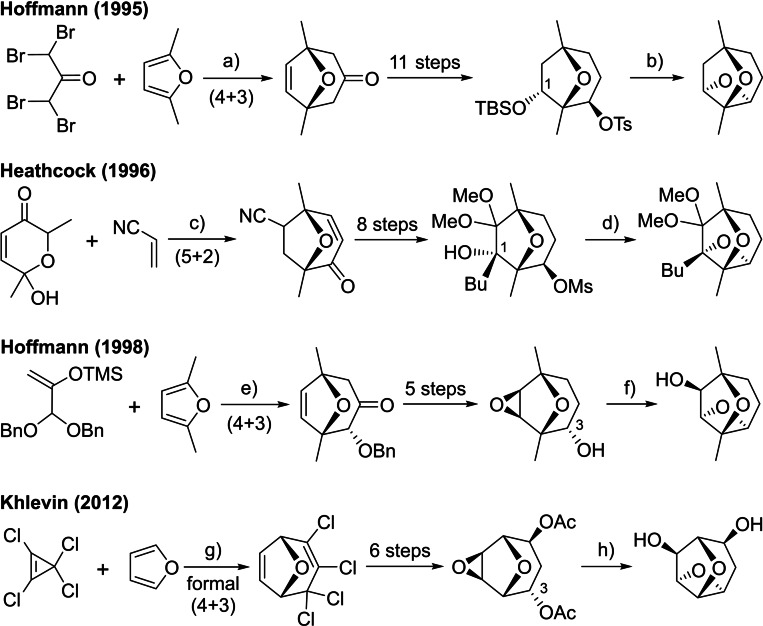
Previous approaches to the dioxatricyclic core of dictyoxetane. Conditions: **a)** Zn, B(OEt)_3,_ THF, rt, then Zn, CuCl, NH_4_Cl, MeOH, 15 °C→rt, 59 %; **b)** TBAF, THF, rt→reflux; **c)** MsCl, ^*i*^Pr_2_NEt, MeCN, reflux, 45 %, isomer ratio 10 : 1 : 1 (only major shown); **d)** NaH, THF, reflux, 88 %; **e)** TMSOTf, CH_2_Cl_2_, −78 °C, 53 % (over 2 steps); **f)** BF_3_⋅OEt_2_, CH_2_Cl_2_, 0 °C, 72 %; **g)** CCl_4_, 80 °C, 92 %; **h)** NaOH, MeOH, rt; TBAF=tetrabutylammonium fluoride, Ms=methanesulfonyl, TMS=trimethylsilyl, Tf=trifluoromethanesulfonyl, NIS=*N*‐iodosuccinimide, Ts=*para*‐toluenesulfonyl, Ac=acetyl.

Hugelshofer and Magauer's approach to dictyoxetane differs from prior model syntheses of the dioxatricyclic core **3** in the order of formation of the oxygen heterocycles. Hoffmann,^[3]^ Heathcock,^[10]^ and Khlevin^[11]^ first established the 8‐oxabicyclo‐[3.2.1]octane subunit via (4+3) cycloadditions^[12]^ reactions of furans or (5+2) cycloadditions of oxidopyrylium betaines, then constructed the oxetane through intramolecular S_N_2‐like displacements of alkoxy nucleophiles at C‐1 or C‐3 (core **3** numbering) under basic or Lewis acidic conditions (Scheme 2). However, these model systems do not contain the additional 1,9‐ring fusion found in the natural product, and attempts to build a hydrindane‐like ring system onto an 8‐oxabicyclo[3.2.1]octane prior to oxetane formation were unsuccessful.^[13]^

**Scheme 3:**
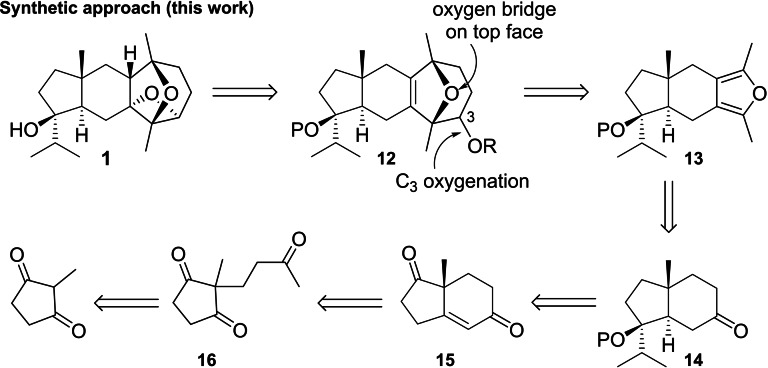
Proposed approach to dictyoxetane via tetrasubstituted furan **13**.

Building on these prior studies, we thought that the reverse strategy of annulation of the oxatricyclic ring system **3** onto *trans*‐hydrindane **2** would be viable if: i) existing furan cycloaddition reactions to prepare 8‐oxabicyclo[3.2.1]oct‐6‐ene ring systems could be extended to tetrasubstituted chiral furan **13**; and ii) oxetane ring‐closure could be effected from tetrasubstituted alkene **12**, or a derivative thereof (Scheme 3). Tetrasubstituted furan **13** was proposed to be formed from regioselective annulation of ketone **14**. Scalemic **14** can be derived from the Hajos‐Parrish ketone **15**, available in high *ee* from an intramolecular asymmetric aldol reaction of **16**, the Michael addition product of 2‐methylcyclopentan‐1,3‐dione with methyl vinyl ketone.^[14]^ Although **15** contains additional oxygenation compared with **4**, we believed that the extra steps required to remove the unwanted carbonyl would be compensated for by the high enantioselectivity of the Hajos‐Parrish ketone preparation, without significantly affecting overall yields.

We herein report synthetic studies towards dictyoxetane based on the retrosynthetic analysis shown in Scheme 3. Through this research, we have developed a large‐scale, asymmetric synthesis of the key protected *trans*‐hydrindanone **14** and a novel furan annulation sequence required for the conversion of cyclohexanone **14** to **13**. We also report rare examples of (4+3) and (4+2) cycloaddition reactions on tetrasubstituted furans towards accessing key intermediate **12**.

## Results and Discussion

### Asymmetric synthesis of *trans*‐hydrindanone 24

**Scheme 4:**
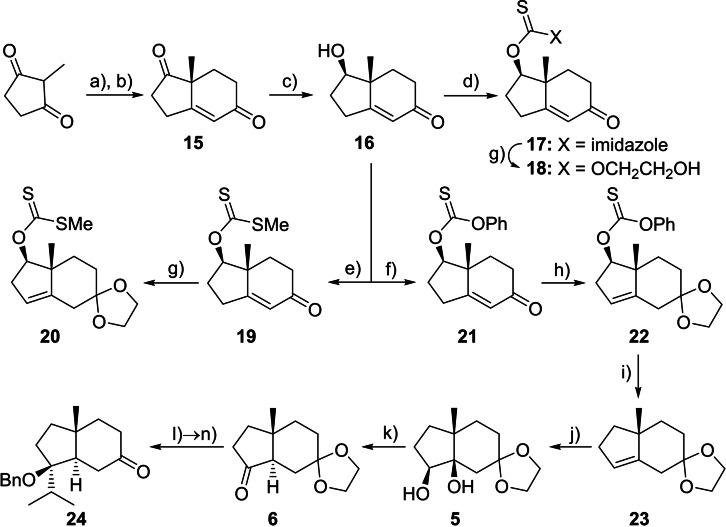
Asymmetric synthesis of benzyl‐protected *trans*‐hydrindanone **24** from 2‐methylcyclopentan‐1,3‐dione. Conditions: **a)** methyl vinyl ketone, AcOH, H_2_O, 70 °C; **b)** (D)‐proline, DMF, 23 °C, then H_2_SO_4_, 73 % (over 2 steps), *er* 99.4:0.6; **c)** NaBH_4_, MeOH, −20 °C, >99 %; **d)** Im_2_CS, toluene, 110 °C, 87 %; **e)** NaH, CS_2_, MeI, THF, 23 °C, 85 %; **f)** PhOC(S)Cl, Py, DMAP, CH_2_Cl_2_, 23 °C; **g)** ethylene glycol, *p*TSA, benzene, reflux under Dean‐Stark, **18** 42 %; **20** 75 %; **h)** ethylene glycol, *p*TSA, benzene, reflux under Dean‐Stark, **22** 72 % (2 steps); **i)** (TMS)_3_SiH, ACCN, toluene, 110 °C; **j)** K_2_OsO_2_(OH)_4_, NMO, ^*t*^BuOH, H_2_O, 85 °C, 62 % (over 4 steps from **16**); **k)** PPh_3_, C_2_Cl_6_, ^*i*^Pr_2_NEt, MeCN, 0→82 °C, 96 %; **l)** ^*i*^PrMgCl, CeCl_3_, THF, 0 °C, 99 %; **m)** KHMDS, BnBr, THF, 0 °C; **n)** HCl, THF, 23 °C, 80 % (over 2 steps); Im=imidazole, Py=pyridine, DMAP=4‐dimethylaminopyridine, *p*TSA=*para*‐toluenesulfonic acid, ACCN=1,1′‐azobis(cyclohexanecarbonitrile), NMO=*N*‐methylmorpholine‐*N*‐oxide, KHMDS=potassium bis(trimethylsilyl)amide.

Hajos‐Parrish ketone **15** was prepared in two steps from commercially available 2‐methylcyclopentan‐1,3‐dione in >98 % *ee* and on >100 g scale using the proline‐catalysed aldol reaction under the conditions initially reported by Hajos and Parrish (Scheme 4).^[14]^ Selective deoxygenation of the non‐conjugated ketone in **15** has not been previously reported, although chemo‐ and stereoselective reduction to alcohol **16** is well known;^[15]^ thus we elected to investigate a radical‐mediated deoxygenation approach.^[16]^ Initially, three thiono derivatives were synthesised (**17**, **19**, and **21**) in high yield, but deoxygenation proved problematic (see Supporting Information for further discussion). Suspecting that the enone may be the issue, we explored acetalisation prior to deoxygenation. Both **19** and **21** proved compatible with the conditions required for acetalisation with concomitant double bond shift,[^17^, ^18^] giving **20** and **22** respectively, but thionocarbamate **17** was acid‐sensitive and lost the imidazole unit, forming the glycol thionocarbonate **18** in low yield.

Although we tested several radical deoxygenation conditions (H_3_PO_2_/Et_3_N/ACCN,^[19] *n*^Bu_3_GeH/ACCN,^[20] *n*^Bu_3_SnH/ACCN,^[16]^ (Bu_4_N)_2_S_2_O_8_/NaOOCH^[21]^), only (TMS)_3_SiH^[22]^ afforded acetal **23** from **20** and **22** in acceptable yields. However, on larger scale, removal of the reaction by‐products from **23**, either by column chromatography or Nussbaumer et al.’s modified work‐up,^[23]^ proved difficult. Furthermore, we observed the partial conversion of the thiono group in both **21** and **22** to a carbonyl upon silica gel chromatography (see Supporting Information).^[24]^ One solution to avoid silica gel was to recrystallise acetal **22**, giving a 72 % yield over two steps from alcohol **16**. However, we found the best solution was to not purify **21** or **22**, and instead vacuum distil **23** to remove most of the impurities, including the by‐products from the deoxygenation; recrystallisation of diol **5** then gave a 62 % yield over 4 steps from **16** with excellent purity. In contrast, xanthate **20** was less amenable to scale up, and could not be recrystallised like **22**.

As in our previous racemic synthesis,^[4]^ diastereoselective Upjohn dihydroxylation of acetal **23** to diol **5** and subsequent phosphorus‐mediated pinacol‐like rearrangement set the key *trans* ring junction stereochemistry. Stereoselective Grignard addition to ketone **6** gave the tertiary alcohol **7**, which we initially chose to protect as a benzyl ether using benzyl bromide and either NaH/NaI/DMF or Hugelshofer and Magauer's KHMDS/THF conditions.^[8]^ Acetal hydrolysis then gave **24**. Overall, our new route allows for the asymmetric synthesis of *trans*‐hydrindanone **24** in excellent enantiopurity over 11 steps and 34 % yield on multi‐gram scale.

### Modelling the furan annulation and (4+3) cycloaddition

**Scheme 5:**
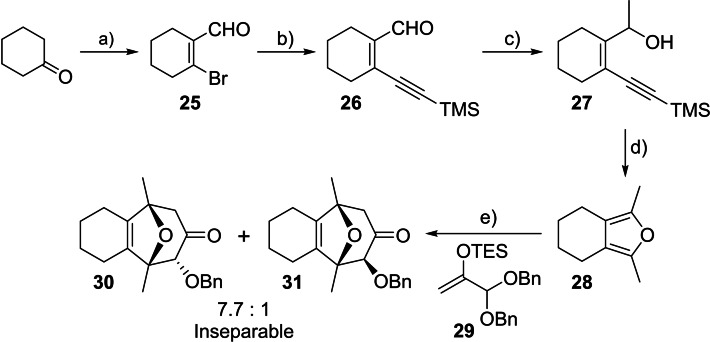
Synthesis of model dioxatricyclic core precursor **30**. Conditions: **a)** PBr_3_, DMF, CH_2_Cl_2_, 88 %; **b)** Pd(PPh_3_)_2_Cl_2_, CuI, Et_3_N, TMSCCH, DMF, 82 %; **c)** MeMgBr, THF, −78 °C, 93 %; **d)** TBAF, THF, 66 °C, used crude; **e) 29**, TMSOTf, CH_2_Cl_2_, −78 °C, 87 % (over 2 steps), *dr* 7.7 : 1. TMS=trimethylsilyl, Tf=trifluoromethanesulfonyl, TBAF=tetrabutylammonium fluoride, TES=triethylsilyl.

Despite extensive research into the (4+3) cycloaddition reaction of allylic cations with furans, there is remarkably little precedent for the use of simple tetrasubstituted furans as dienes in these transformations.[^25^, ^26^, ^27^] We therefore elected to study the furan annulation ‐ (4+3) cycloaddition sequence on a simpler cyclohexanone‐based model system in the first instance. Transformation of cyclohexanone to dimethylfuran **28** in four steps has been previously reported based on a variant of the Garst‐Spencer furan annulation to prepare a 2‐thiosubstituted furan, followed by the sequential introduction of two methyl groups.^[28]^ Preliminary investigation suggested that the Garst‐Spencer approach was not suitable for delivering significant quantities of **28**, and so we developed an alternative sequence that was more readily scalable (Scheme 5). Haloformylation of cyclohexanone under Vilsmeier‐Haack conditions gave bromoenal **25**, which was cross‐coupled to trimethylsilylacetylene in a Sonogashira reaction to give enynal **26**.^[29]^ Addition of MeMgBr gave enynol **27**, which underwent cyclisation‐isomerisation to furan **28** upon treatment with tetrabutylammonium fluoride (TBAF).[^30^, ^31^]

Tetrasubstituted furan **28** proved to be an excellent diene for a (4+3) cycloaddition reaction with an oxygen‐substituted oxyallyl cation using Hoffmann's conditions.^[3b]^ Reaction of **28** with oxyallyl cation precursor **29** at −78 °C gave oxabicycles **30** and **31** as an inseparable 7.7 : 1 mixture of diastereomers in excellent overall yield over two steps from **27** (Scheme 5). The major stereoisomer was assigned as **30** on the basis of the greater downfield shift of the C−H adjacent to the OBn group compared to **31**, consistent with an equatorial OBn and axial H on the 6‐membered oxygen heterocycle as is typically observed in related 2‐alkoxyl‐8‐oxabicyclo[3.2.1]octan‐3‐one ring systems derived from (4+3) cycloadditions of oxygen‐substituted oxyallyl cations.[^32^, ^33^]

### Hydrindanone annulation and furan cycloaddition

Having established a new furan annulation and the feasibility of the (4+3) cycloaddition of an oxyallyl cation with a tetrasubstituted furan, we attempted to apply the methodology on hydrindanone **24**. Unexpectedly, the haloformylation conditions previously used to synthesise bromoacraldehyde **25** resulted in decomposition of **24**, with no evidence of formation of **32** using either PBr_3_ or POBr_3_. Treatment of **24** with POCl_3_ afforded chloroenal **33** in 75 % yield, with regioselectivity consistent with reactions on related *trans*‐hydrindanones.^[34]^ However, despite ample precedent for the cross‐coupling of chloroenals and vinyl chlorides, the Sonogashira reaction with trimethylsilylacetylene failed to deliver **34** under a range of conditions.^[35]^

**Scheme 6:**
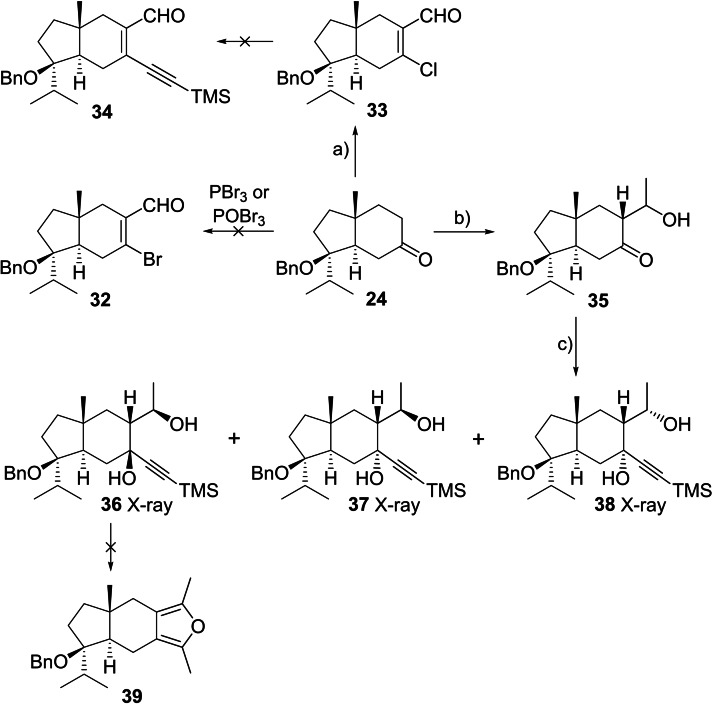
Attempted furan annulation on benzyl‐protected hydrindanone **24**. Conditions: **a)** POCl_3_, DMF, CH_2_Cl_2_, 75 %; **b)** CH_3_CHO, LiHMDS, THF, −78 °C; **c)** CeCl_3_, ^*n*^BuLi, TMSCCH, THF, −78 °C, 43 % **36**, 8 % **37**, 7 % **38**, 25 % **36**+**37** (over 2 steps); LiHMDS=lithium bis(trimethylsilyl)amide.

The failure of the bromoformylation reaction was surprising given the usually robust nature of the benzyl group. We envisaged access to the target furan **39** in an alternative manner via an acetylenic 1,3‐diol, followed by cyclisation with dehydration.^[36]^ A mixture of three 1,3‐diols **36**–**38** was prepared in two steps from ketone **24** by a regio‐ and stereoselective aldol reaction with acetaldehyde to synthesise **35**,^[8]^ followed by a CeCl_3_‐mediated addition of lithium (trimethylsilyl)acetylide to the ketone (Scheme 6).^[37]^ Structural information for the 1,3‐diols **36**–**38** was obtained via X‐ray diffraction analysis (see Supporting Information).^[38]^ The major compound **36** is derived from addition of the alkyne to the lower face of ketone **35**. Diol **37** results from alkyne addition from the upper face, while **38** is derived from the minor aldol product. Unfortunately, attempts to cyclise major diol **36** to furan **39** under a range of conditions, including KO^*t*^Bu,^[31]^ Pd^II^,[^30^, ^39^] Ag^I^,^[40]^ HCl,^[39c]^ and Cu^II^,[^39c^, ^41^] were unsuccessful.

**Scheme 7:**
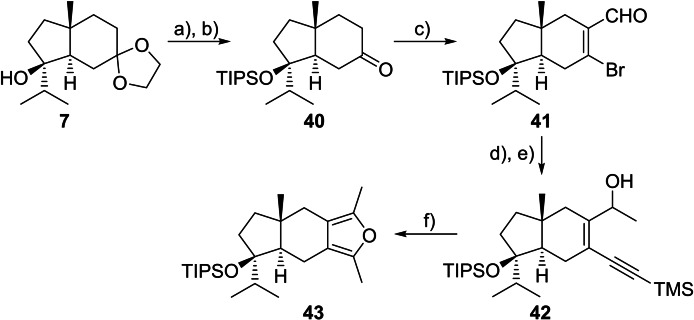
Synthesis and furan annulation of TIPS‐protected hydrindanone **40**. Conditions: **a)** KHMDS, TIPSCl, DMF, 0 °C; **b)** 4 M HCl, THF, 30 °C, 82 % (over 2 steps); **c)** PBr_3_, DMF, 0→80 °C, 75 %; **d)** TMSCCH, Pd(PPh_3_)_2_Cl_2_, Et_3_N, THF, 65 °C; **e)** MeMgCl, THF, −78 °C, 91 % (over 2 steps), *dr* 3 : 1; **f)** K_2_CO_3_, MeOH, THF, 60 °C, 98 %; KHMDS=potassium bis(trimethylsilyl)amide, TIPS=triisopropylsilyl.

With the failure to annulate benzyl‐protected hydrindanone **24** to furan **39**, we elected to reinvestigate the bromoformylation route with an alternative alcohol protecting group that could tolerate the Vilsmeier‐Haack conditions. Following protection of tertiary alcohol **7** as a TIPS ether and acetal deprotection, haloformylation of ketone **40** with PBr_3_/DMF afforded bromoenal **41** in 75 % yield as a single regioisomer (Scheme 7).^[29]^ Copper‐free Sonogashira cross‐coupling^[35]^ followed by the addition of MeMgCl afforded an inconsequential diastereomeric mixture (3 : 1) of enynols **42**. Conversion of **42** to furan **43** using TBAF/THF^[30]^ produced multiple products, while Marshall's KO^*t*^Bu/^*t*^BuOH conditions^[31]^ only partially removed the TMS group before cyclisation. However use of K_2_CO_3_/MeOH/THF delivered furan **43** in an excellent 98 % yield.

With furan **43** in hand, methods for the construction of the 8‐oxabicyclo[3.2.1]octane subunit were investigated. In contrast to prior model systems (Schemes 2 and 5), the cycloaddition of chiral furan **43** requires control over both facial selectivity and a means to regio‐ and stereoselectively incorporate an alcohol at C‐3 (dioxatricyclic core **3** numbering). We anticipated the axially orientated methyl group at the hydrindane ring junction would influence facial selectivity, as was observed for the aldol reaction of **24**, directing reaction through the lower face of furan **43**.

**Scheme 8:**
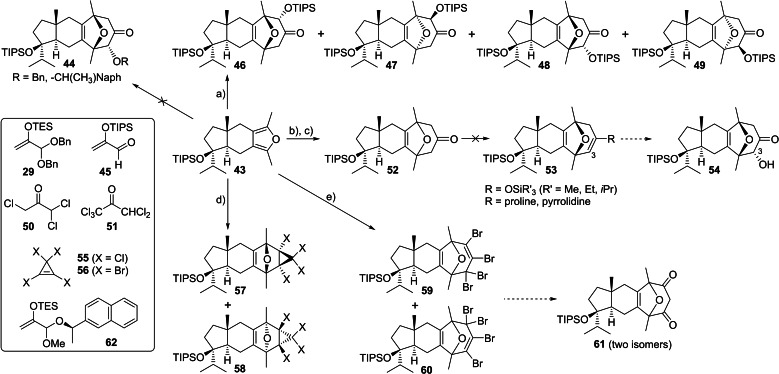
Evaluation of a formal (4+3) cycloaddition approach to dictyoxetane using furan **43**. Conditions: **a) 45**, Sc(OTf)_3_, CH_2_Cl_2_, 0 °C, 18 % **46** 2 % **47** 15 % **48** 9 % **49** ; **b) 50** or **51**, Et_3_N, toluene, TFE, 16 °C; **c)** Zn dust, NH_4_Cl, MeOH, 60 °C, 80 % from **50**, *dr* 1.4 : 1, 59 % from **51**, *dr* 4 : 1; **d) 55**, toluene, 23 °C, 91 % *dr* 1 : 1 or **56**, toluene, −78 °C to 23 °C, 93 % *dr* 1 : 1; **e) 56**, toluene, 23 °C then 110 °C, 95 % combined yield of a 1 : 1 : 1 : 1 mixture of four isomers. Tf=trifluoromethanesulfonyl, TFE=2,2,2‐trifluoroethanol, Naph=2‐naphthyl, TES=triethylsilyl, TIPS=triisopropylsilyl.

We first elected to study (4+3) cycloaddition reactions of oxygen‐substituted oxyallyl cations to directly introduce the requisite C‐3 alcohol, as in our successful model study (Scheme 5). However, in contrast to the simpler furan **28**, no reaction between furan **43** and the benzyloxyallyl precursor **29** was observed at −78 °C, and while warming the reaction to ca. −40 °C led to consumption of material, a complex mixture was produced, with no evidence for formation of target cycloadduct **44** (Scheme 8). Use of 2‐(siloxy)acrolein **45** in a Sc(OTf)_3_‐catalysed cycloaddition reaction^[42]^ was more encouraging, leading to the formation of four cycloadducts **46**–**49**. The structures of **46**, **48**, and **49** were determined by 2D NMR analysis, and showed the desired *anti*‐relationship between the oxygen bridge and the adjacent silyloxy group, consistent with Harmata's results with 2,5‐dimethylfuran and furan.^[42]^ Cycloadduct **47** was not obtained in significant enough quantity to determine the absolute structure by NMR analysis, and was therefore inferred to be the last *anti* isomer possible. Despite the formation of cycloadducts, the modest combined yield (44 %) and lack of significant facial and regioselectivity made the siloxyacrolein chemistry unfeasible for total synthesis.

To remove the C‐3 alkoxy regio‐ and stereochemistry issue in the (4+3) cycloaddition reaction, we investigated the use of haloacetone oxyallyl cation precursors (Scheme 8).^[43]^ After dehalogenation, a maximum of two ketones can be formed, resulting from addition to either face of furan **43**. Treatment of **43** with 1,1,3‐trichloroacetone **50** and Et_3_N gave an inseparable 1.4 : 1 mixture of ketones **52** in 80 % yield following zinc‐mediated dehalogenation. Use of pentachloroacetone **51** as the coupling partner afforded a significantly more biased 4 : 1 mixture, albeit in lower overall yield. Although we were unable to unambiguously determine the stereochemistry of the major cycloadduct, we believe it is likely that reaction preferentially occurs from the lower face of furan **43** to deliver the desired oxygen‐bridge stereochemistry: the analogous (4+3) reaction of silyloxyacrolein **45** with **43** is ca. 75 % selective for addition to the lower face (**46**+**48** : **47**+**49**). Unfortunately, selective α‐oxygenation of the mixture of ketones **52** could not be achieved using either an asymmetric deprotonation‐silyl enol ether oxidation approach,^[33]^ or through proline‐catalysed α‐aminoxylation.^[44]^ The poor reactivity of **52** is also reflected in its failure to form a simple enamine with pyrrolidine.

Tetrahalocyclopropenes undergo Diels‐Alder reactions with furans followed by in situ ring‐opening to form synthetically versatile oxygen‐bridged tetrahalocycloheptadienes.[^11^, ^12^, ^45^] Treatment of furan **43** with tetrachlorocyclopropene (**55**) at room temperature gave a 1 : 1 inseparable mixture of cyclopropanes **57** and **58**, derived from reaction with either face of the furan and assigned as *exo* adducts based on work by Wallerstein et al. (Scheme 8).^[46]^ Use of tetrabromocyclopropene (**56**) at lower temperature gave a similar yield, paralleling early findings that bromide, whilst sterically more demanding, is more electronically activating in these dienophiles. However, no improvement in selectivity is seen on changing from **55** to **56**. A tandem cycloaddition‐thermal ring‐expansion reaction sequence using **56** gave regioisomeric oxabicycles **59** and **60**, each as a 1 : 1 mixture of facial isomers, in 95 % combined yield. Although the poor selectivity precluded further application in the synthesis of dictyoxetane itself, the possibility that the isomers could be converged to a 1 : 1 mixture of diketones **61** may be of future utility towards the synthesis of regio‐ and epimeric dictyoxetane analogues.

**Scheme 9:**
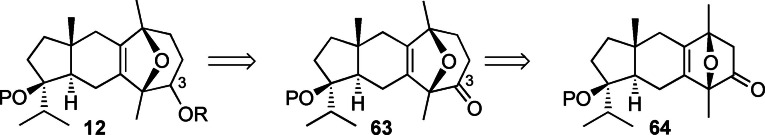
Alternative approach to intermediate **12**.

Our work on the formal (4+3) cycloaddition of furan **43** highlighted that, with the exception of pentachloroacetone **51**, facial bias was low, suggesting that the axially orientated methyl group at the *trans*‐hydrindane ring‐junction alone was not capable of delivering the levels of selectivity required. Attempts to use the chiral oxyallyl cation precursor **62** to override substrate bias and control regioselectivity were unsuccessful due to poor furan reactivity at low temperatures. We therefore considered an alternative approach to intermediate **12** based on reduction of ketone **63**, to be accessed through a regioselective one‐carbon ring‐expansion of oxanorbornenone **64** (Scheme 9).^[47]^ Ketone **64** is the formal Diels‐Alder reaction product of furan **43** with ketene, and hence offers the opportunity to influence facial and regioselectivity through reagent control, using an appropriate chiral ketene equivalent. Towards this goal, we herein report our preliminary investigations into the use of Aggarwal's C_2_‐symmetric chiral ketene equivalent **65** as the dienophile in a Diels‐Alder reaction with furan **43**. Alkene **65** shows high reactivity in a range of cycloadditions,^[48]^ and has recently been employed in natural product synthesis involving ring‐expansion of the resulting ketone.^[49]^

**Scheme 10:**
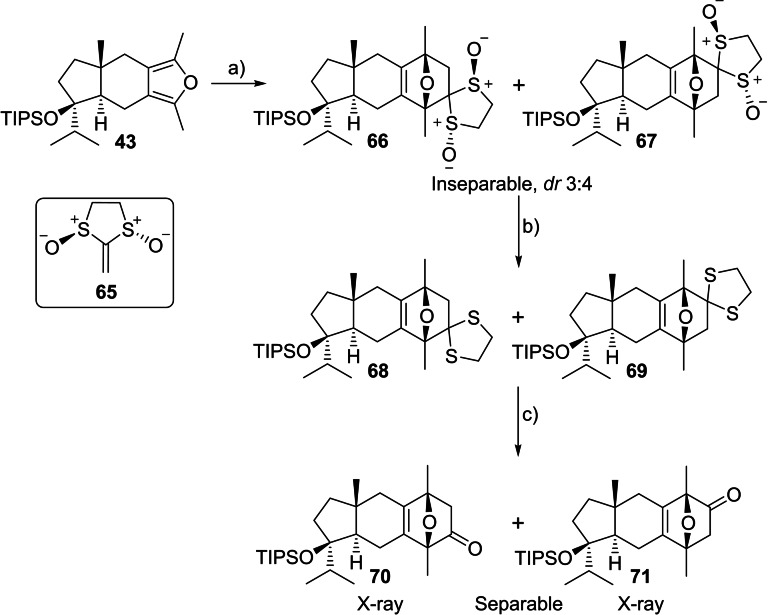
Diels‐Alder cycloaddition of furan **43** with chiral ketene equivalent **65**. Conditions: a) **65**, CH_2_Cl_2_, 0 °C, 82 % combined yield of **66** and **67**, 3 : 4 ratio of regioisomers; b) TFAA, NaI, acetone, −78 °C, 73 % combined yield; c) MeI, CaCO_3_, H_2_O, THF, 60 °C, 41 % **70**, 44 % **71**; TFAA=trifluoroacetic anhydride.

Treatment of furan **43** with enantiopure **65** at 0 °C gave predominantly two compounds as an inseparable mixture in a 4 : 3 ratio and in 82 % combined yield (Scheme 10). While the structures could not be elucidated fully by NMR analysis, data were consistent with Diels‐Alder adducts. Fortunately, X‐ray crystal structures obtained on subsequent derivatives showed that **66** and **67** were regioisomers, both derived from reaction with the lower face of furan **43**, producing the desired top‐facing oxygen bridge. The bissulfoxide moiety was reduced with TFAA/NaI^[48b]^ to give an inseparable mixture of dithiolanes **68** and **69**. Attempted thioacetal hydrolysis under the literature conditions (CuCl_2_/SiO_2_ with mild heating)[^48a^, ^48b^] resulted in the return of furan **43** as the exclusive product. Among several screened conditions (PIFA with and without NaHCO_3_,^[50]^ NBS/AgNO_3_/CaCO_3_,^[51]^ I_2_/CaCO_3_ ^[52]^), only MeI/CaCO_3_ ^[53]^ afforded the oxanorbornenones **70** and **71**, which were separable by column chromatography. X‐ray crystallography^[38]^ allowed for the unambiguous structural determination of both **70** and **71** and thus established that the Diels‐Alder cycloaddition of **43** with **65** resulted in a mixture of regioisomers **66** and **67**.

## Conclusion

In summary, studies towards the asymmetric synthesis of (+)‐dictyoxetane have been presented, based around the cycloaddition of a furan‐annelated *trans*‐hydrindane to assemble the carbocyclic core of the natural product. A high‐yielding and scalable synthesis of enantiopure *trans*‐hydrindane **24**, a known intermediate in the reported asymmetric total synthesis of dictyoxetane,^[8]^ was achieved through selective deoxygenation of the Hajos‐Parrish ketone. A 4‐step conversion of cyclohexanones to tetrasubstituted furans was developed as an alternative to a Garst‐Spencer annulation ‐ sequential dimethylation approach. Application of this methodology to a *trans*‐fused hydrindanone required protection of an embedded tertiary alcohol as a TIPS ether, the corresponding benzyl ether proving to be incompatible with the Vilsmeier‐Haack bromoformylation step. Tetrasubstituted furans were shown to participate in a range of (4+3) cycloadditions, but levels of regio‐ and stereoselectivity were modest using substrate control from the fused *trans*‐hydrindane ring system. In an alternative approach based on reagent control, a C_2_‐symmetric chiral ketene equivalent underwent (4+2) cycloaddition with excellent facial selectivity, but with low regioselectivity. Overall, we expect these studies to facilitate future research into the synthesis of dictyoxetane and simpler ring‐fused analogues, and tetrasubstituted furans in general.

## Conflict of interest

The authors declare no conflict of interest.

1

## Supporting information

As a service to our authors and readers, this journal provides supporting information supplied by the authors. Such materials are peer reviewed and may be re‐organized for online delivery, but are not copy‐edited or typeset. Technical support issues arising from supporting information (other than missing files) should be addressed to the authors.

Supporting Information

## Data Availability

The data that support the findings of this study are openly available in University of Birmingham eData Repository (UBIRA eData) at https://doi.org/10.25500/edata.bham.00000869, reference number 869.
