# Supplementary material for: Studies towards the Synthesis of (+)‐Dictyoxetane
Source: Chemistry. 2022 Oct 27;28(71):e202202429. doi: 10.1002/chem.202202429 (PMC10092742; doi:10.1002/chem.202202429)
Supplement: Supplementary file 1 — Supporting Information [file CHEM-28-0-s001.pdf]

Chemistry–A European Journal

Supporting Information

Studies towards the Synthesis of (+)-Dictyoxetane

Joseph Benford-Ward, Sanaz Ahmadipour, Aliya Sembayeva, Louise Male, and Richard S. Grainger*

Primary data files for this article have been deposited in the University of Birmingham data archive: https://doi.org/10.25500/edata.bham.00000869

Contents

Contents 1

General Experimental **5**

Reagents and Solvents 5

Analysis 5

Reaction Setup 7

Investigation of Conditions for the radical deoxygenation of Compounds 17, 19, 20, 21 and 22 9

Procedures **13**

Diol 5 13

Trans-hydrindane 6 14

Tertiary alcohol 7 15

Hajos-Parrish Ketone 15 16

Alcohol 16 17

Thionocarbamate 17 18

Thionocarbonate acetal 18 19

Xanthate 19 20

Xanthate acetal 20 21

O-Phenyl thionocarbonate 21 (and carbonate S2) 22

Thionocarbonate acetal 22 (and carbonate S3) 24

Acetal 23 25

Benzyl hydrindanone 24 26

Bromoenal 25 27

Enynal 26 28

Enynol 27 29

Furan 28 *via* enynol S5 30

Silyl enol ether 29 31

Oxabicyclic ketones 30 and 31 32

Chloroenal 33 33

Keto alcohol 35 34

Alkyne 1,3-diols 36–38 35

TIPS ketone 40 38

Bromoenal 41 39

Enynol 42 41

Furan 43 43

TIPS acrolein 45 44

Oxabicyclic ketones 46–49 45

Pentachloroacetone 51 48

Oxabicyclic ketone 52 49
Tetrabromocyclopropene 56 51
Tetrachlorocyclopropanes 57a and 58a 51
Tetrabromocyclopropanes 57b and 58b 52
Tetrabromides 59 and 60 54
Sulfoxides 66 and 67 55
Thioacetals 68 and 69 57
Oxanorbornenones 70 and 71 58
Triketone S1 60
Benzyl ether S4 61
Dibenzyl acetal S6 62
TIPS ether S7 63
Dimethylamine S10 64
Rac/(*S,S*)-bissulfoxide S11 64
Benzyl ether S12 66
1-Bromo-1-methoxypropan-2-one S14 67
1-Methoxy-1-((*R*)-1-(naphthalen-2-yl)ethoxy)propan-2-one S15 67
Triethyl((3-methoxy-3-((*R*)-1-(naphthalen-2-yl)ethoxy)prop-1-en-2-yl)oxy)silane S16 69
Tosylate S17 70
Tosylate acetal S18 71
NMR SPECTRA 72
Diol 5 73
Trans-hydrindane 6 75
Tertiary alcohol 7 77
Hajos-Parrish ketone 15 79
Alcohol 16 81
Thionocarbamate 17 83
Thionocarbonate acetal 18 85
Xanthate 19 87
Xanthate acetal 20 89
O-Phenyl thionocarbonate 21 91
Thionocarbonate acetal 22 93
Acetal 23 95
Benzyl hydrindanone 24 97
Bromoenal 25 99
Enynal 26 101
Enynol 27 103
Furan 28 105
Silyl enol ether 29 107
Oxabicyclic ketones 30 and 31 109
Chloroenal 33 111
Keto alcohol 35 113

Alkyne 1,3-diol 36 115
Alkyne 1,3-diol 37 117
Alkyne 1,3-diol 38 119
TIPS Ketone 40 121
Bromoenal 41 123
Enynol 42 – Isomer 1 125
Enynol 42 – Isomer 2 127
Furan 43 129
TIPS acrolein 45 131
Oxabicyclic ketones 46 and 47 133
Oxabicyclic ketone 48 138
Oxabicyclic ketone 49 143
Pentachloroacetone 51 148
Oxabicyclic ketone 52 150
Tetrabromocyclopropene 56 152
Tetrachlorocyclopropanes 57a and 58a 153
Tetrabromocyclopropanes 57b and 58b 155
Tetrabromides 59 and 60 157
Sulfoxides 66 and 67 159
Thioacetals 68 and 69 161
Oxanorbornenone 70 163
Oxanorbornenone 71 165
Triketone S1 167
Carbonate S2 169
Carbonate S3 170
Benzyl ether S4 172
Dibenzyl acetal S6 174
TIPS ether S7 176
Dimethylamine S10 179
(S,S)-Bissulfoxide S11 181
Benzyl ether S12 183
1-Bromo-1-methoxypropan-2-one S14 185
1-Methoxy-1-((R)-1-(naphthalen-2-yl)ethoxy)propan-2-one S15 187
Triethyl((3-methoxy-3-((R)-1-(naphthalen-2-yl)ethoxy)prop-1-en-2-yl)oxy)silane S16 189
Tosylate S17 191
Tosylate acetal S18 193
HPLC DATA **195**
Hajos-Parrish ketone 15 196
X-RAY DATA **198**
Alkyne 1,3-diol 36 201
Alkyne 1,3-diol 37 202
Alkyne 1,3-diol 38 203

Oxanorbornenone 70	204
Oxanorbornenone 71	206
Tosylate S17	208
Tosylate acetal S18	209
**REFERENCES**	**210**

General Experimental

Reagents and Solvents

All reagents were bought commercially from either Sigma-Aldrich (Merck), Alfa Aesar, Acros Organics, Fisher Scientific, VWR, or Fluorochem, and were used as received unless stated. ^{i}PrMgCl was bought as a 2 M solution in THF and titrated against a solution of menthol (2.5 mmol) and 1,10-phenanthroline; MeMgCl was bought as a 3 M in THF, and titrated the same way.[1] ^{n}BuLi was bought as a 2.5 M in hexanes and titrated with menthol and the indicator 'blue'.[2] PPh_3 was recrystallised via a two-step process: 20 g dissolved in 150 mL conc. HCl and precipitated using 150 mL H_2O, the filter cake was then recrystallised from 1:1 EtOH-Et_2O to give the purified material, stored in a dessicator cabinet with silica as the dessicant. *m*CPBA was purified by a phosphate buffer: *m*CPBA (5 g) was dissolved in Et_2O (25 mL) and washed with buffer (3 × 25 mL, [2.5 g NaOH, 27 g KH_2PO_4, 250 mL H_2O]), then dried over $MgSO_4$, filtered and concentrated under reduced pressure [**CAUTION! Detonation Risk**].[3] MeI, TESCl, TIPSCl, TMSCl, and AcBr were passed through either basic alumina or K_2CO_3 immediately prior use. All solvents were bought from one of the above suppliers and used without further drying or purification unless stated below. Any solvents that were dried were stored under argon in a round-bottom flask sealed with a rubber septum. Before and after every use the flask was flushed with argon gas. MeOH, toluene, THF, CH_2Cl_2, Et_2O, 1,4-dioxane, hexane and DMF were dried over 3 Å molecular sieves for at least 24 h before use.[4] MeCN was distilled from CaH_2 onto 3 Å molecular sieves and stored for at least 24 h before use. Et_3N, 2,6-lutidine and pyridine were distilled from CaH_2 and stored over KOH. Acetone and MVK were freshly distilled from K_2CO_3. Cyclohexanone was vacuum-distilled (30 mBar) from $MgSO_4$. H_2O was used in a deionised state as provided by a water deioniser. Petroleum ether refers to the fraction with boiling point 40-60 °C. All aqueous solutions are saturated unless stated otherwise.

Analysis

Silica gel on aluminium-backed TLC plates were used for reaction monitoring, supplied from Merck (60F254). The plates were visualised by UV (254 nm) and standard laboratory agents: $KMnO_4$, anisaldehyde, vanillin, iodine powder. Purification by flash column chromatography

was performed on Sigma-Aldrich/Fluorochem silica gel, pore size 60 Å, 230-400 mesh particle size, 40-63 µm particle size.[5] Infra-red spectra were recorded neat (oil), thin-film, or with the aid of an ATR-attachment (solid) on a Perkin Elmer Spectrum 100 FT-IR spectrometer, only selected absorbances ($\tilde{\nu}_{max}$, cm^{-1}) are reported. The following abbreviations are used when describing the data: w (weak), m (medium), st (strong), n (narrow), br (broad), sh (sharp). Melting points were recorded using open glass capillaries on a Gallenkamp melting point apparatus and are uncorrected, with three data points recorded and averaged. Samples for melting points were obtained by recrystallising a small portion of the bulk material from the solvent in brackets. Optical activities were recorded on polarimeter PolAAr 2001 and are calculated according to the following equation:

$$[\alpha]_D^{25} = \frac{[\alpha] \cdot 100}{c \cdot l}$$

Where c is concentration (g 100 mL^{-1}), l is path length (mm), and $[\alpha]$ is the measured optical rotation. MS data are reported as m/z (%) (relative intensity except in cases where only the parent ion is observed), from the following instruments: Bruker MicroTOF QII, Waters Xevo G2-XS, Waters GCT, and Waters LCT. ^{1}H and ^{13}C NMR spectra were recorded on a Bruker AVIII300, Bruker AVIII400, and Bruker NEO400 in the solvents indicated. The solvent signals were used as references: residual $CHCl_3$ (^{1}H, 7.26 ppm), $CDCl_3$ (^{13}C, 77.16 ppm), residual CH_2Cl_2 (^{1}H, 5.32 ppm), CD_2Cl_2 (^{13}C, 53.84 ppm), residual C_6H_6 (^{1}H, 7.16 ppm) and C_6D_6 (^{13}C, 128.06 ppm). Coupling constants (*J*) are reported in Hz, and are reported as observed, not averaged between the two environments that share them. The following abbreviations are used to describe multiplicity in ^{1}H-NMR: m (multiplet), st (stack), s (singlet), d (doublet), t (triplet), q (quartet), p (pentet), sept (septet), *ap.* (apparent) and in ^{13}C-NMR: C (quaternary), CH (tertiary), CH_2 (secondary) and CH_3 (primary). The distinction between multiplet and stack: a multiplet is a single environment that is too convoluted to establish its multiplicity correctly, a stack is where multiple environments overlap and their fidelity is lost. 1D ^{13}C NMR spectra were recorded using UDEFT or PENDANT pulse sequences from the Bruker standard pulse program library. 2D ^{1}H-^{13}C HSQC, ^{1}H-^{1}H COSY, ^{1}H-^{1}H NOESY and ^{1}H-^{13}C HMBC NMR spectra were recorded using the Bruker standard pulse program library. Spectra were processed using MestReNova version 10. Where the data are ambiguous and absolute assignment cannot be

made from the data, generic assignments are given (e.g. CH_2, 2 × 1H of CH_2), otherwise compound structures are numbered fully, not necessarily in accordance with their naming scheme, and this numbering is used to assign environments. In cases of diastereotopic protons H-#′ and H-#′′ are used when an absolute assignment cannot be made. Analytical chiral HPLC was performed by REACH separations in Nottingham, UK, using a Lux C4 (4.6 mm × 250 mm × 5 μm); chiral column-conditions will be stated where necessary.

For X-ray diffraction data, the datasets were measured on an Agilent SuperNova diffractometer using an Atlas detector. The data collections were driven and processed and absorption corrections were applied using CrysAlisPro.[S1] Using Olex2,[6] the structures of Tosylate **S17** and Tosylate acetal **S18** were solved using ShelXS[S2] and the remaining structures were solved using ShelXT,[7] and all structures were refined by a full-matrix least-squares procedure on F^2 in ShelXL.[8]

S1 CrysAlisPro, Agilent Technologies, Version 1.171.37.35, **2014** and Version 1.171.39.46, **2018**.

S2 G. M. Sheldrick, *Acta Cryst.* **2008**, *A64*, 112-122.

Reaction Setup

All reaction flasks were stored and dried in an oven (150 °C); reactions should be assumed to be carried out using dry glassware unless they contain or are removing H_2O. Flasks were cooled under vacuum and backfilled with argon for use. Argon gas was the chosen inert gas, lines were dried by passing the gas through drying silica, and needles were stored in a rubber septum over drying silica contained within a conical flask. Degassing refers to inserting a syringe needle into the middle of a solution and bubbling argon through at more than one bubble per second for the time stated. Reactions requiring heating were carried out using hotplates and an appropriately-sized heating block. A digital thermometer was used to establish the temperature of the hotplate and infer the temperature of the reaction flask. The following cooling baths were used: 0 °C (H_2O-ice), −40 °C (dry ice-MeCN), −78 °C (dry ice-acetone), −95 °C (N_2/MeOH). Typically, −10 to −20 °C was established by adding dry ice to acetone gently until the desired temperature was reached, monitored by thermometer, and careful maintenance of this state by further addition of dry ice as opposed to typical ice-salt-

water mixtures. All reactions were stirred with Teflon-coated magnetic stirrer bars of appropriate size for the reaction volume unless stated otherwise. Filtration of the drying agent ($MgSO_4$ or Na_2SO_4) was performed under suction through glass or cotton wool. In cases where the solid was required, Whatman filter paper of an appropriate size was placed over a sintered glass funnel and the filtrate was pulled through under suction. Solvent removal was achieved using a pressure-controlled rotary evaporator at the minimum pressure required to remove solvent until complete removal was achieved, using a typical temperature of 40–50 °C unless otherwise specified. Ultimate solvent removal was achieved under high vacuum ($<10^{-2}$ mbar, as measured by a digital manometer) using a rotary vane pump and glass manifold setup. Flash column chromatography on silica gel was performed using a standard glass column, (or in test-scale cases a syringe) either with a sinter or using sand as a levelling-agent. Silica gel was loaded as a slurry and compacted under pressure from either a set of bellows or a fish tank pump. Fractions were collected in appropriately-sized test tubes and interrogated by TLC analysis.

Investigation of Conditions for the radical deoxygenation of Compounds 17, 19, 20, 21 and 22

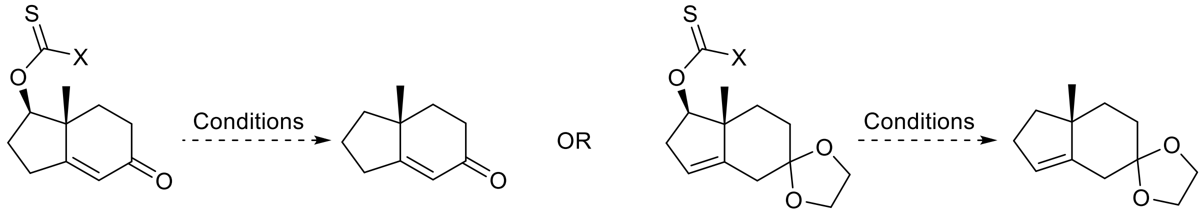

X = SMe **19**
X = OPh **21**
X = imid **17**

X = SMe **20**
X = OPh **22**

Entry	Substrate	Method	Start Mass (g)	Scale (mmol)	Yield (%)	Comment
1	**19**	A	0.021	0.082	0	Multiple products
2	**19**	B	0.011	0.043	0	Multiple products
3	**17**	A	0.099	0.358	11	Poor recovery
4	**17**	B	0.068	0.246	0	Poor starting material consumption
5	**17**	C	0.050	0.181	85	See discussion below
6	**17**	C	0.200	0.724	62	See discussion below
7	**17**	D	0.100	0.362	98	Impure
8	**21**	A	0.060	0.198	47	Multiple products
9	**21**	C	0.053	0.175	23	Silicon byproducts
10	**21**	C	0.052	0.172	58	See discussion below
11	**21**	C	0.053	0.175	61	See discussion below
12	**22**	A	0.088	0.254	0	Product formed by TLC
13	**22**	B	0.101	0.292	60	Poor starting material consumption
14	**22**	B	0.199	0.574	67	Impure
15	**22**	C	0.104	0.300	201	Silicon byproducts
16	**22**	C	0.030	0.087	71	Modified work up[a]
17	**22**	C	0.500	1.443	97	See discussion below
18	**22**	C	1.001	2.889	88	See discussion below
19	**22**	C	4.997	14.424	89	See discussion below
20	**22**	C	5.421	15.647	82	See discussion below
21	**22**	C	9.780	28.230	52	Purified in two batches
22	**22**	C	4.160	12.008	93	First vacuum distillation
23	**22**	D	0.153	0.442	97	Impure
24	**22**	E	0.051	0.147	42	Impure

Table S1: Key results in attempted deoxygenation of compounds **19**, **21**, **17** and the acetalised compounds **20** and **22**. The data show that method C with thionocarbonate **22** was most effective, particularly when purifying by vacuum distillation.

[a] P. Kraft, C. Weymuth, C. Nussbaumer, *Eur. J. Org. Chem*. **2006**, 1403–1412

Method A = $(Bu_4N)_2S_2O_8$, NaOOCH, DMF, 60 °C (H. S. Park, H. Y. Lee, Y. H. Kim, *Org. Lett.* **2005**, 7, 3187–3190)

Method B = H_3PO_2, Et_3N, ACCN, dioxane, 100 °C (D. H. R. Barton, D. O. Jang, J. C. Jaszberenyi, *Tetrahedron Lett*. **1992**, 33, 5709–5712)

Method C = $(TMS)_3SiH$, ACCN, toluene, 110 °C (C. Chatgilialoglu, D. Griller, M. Lesage, *J. Org. Chem.* **1988**, 53, 3641–3642)

Method D = $^nBu_3GeH$, ACCN, toluene, 110 °C (W. Russell Bowman, S. L. Krintel, M. B. Schilling, *Org. Biomol. Chem*. **2004**, *2*, 585–592)

Method E = $^nBu_3SnH$, ACCN, toluene, 110 °C (D. H. R. Barton, S. W. McCombie, *J. Chem. Soc., Perkin Trans. 1* **1975**, 1574–1585)

Discussion

Xanthate **19**: due to synthetic limitations only a few tests could be performed. Neither method A nor B delivered reasonable outcomes, both forming multiple products by TLC (Table S1, entries 1 and 2). In the small amounts of isolated material, spectroscopic analysis was unable to confirm the presence of enone **4**.

Thionocarbamate **17**: Method A did give enone **4** as a product, however the recovery was extremely poor and often there were many products (entry 3). In contrast, method B generated a single product by TLC, but this method failed to consume **17** and again little could be isolated after purification (entry 4). The first success utilised $(TMS)_3SiH$ (method C, entries 5 and 6). At first a yield of 85% was obtained, but upon increasing the scale from 50 mg to 200 mg the yield diminished to *ca.* 60% consistently despite little change in experimental conditions. Finally, use of $^nBu_3GeH$ (method D) effected complete consumption of **17**, however there was significant impurity from the germanium residues that could not be removed from the isolated oil, evidenced by ^1H NMR spectroscopy (entry 7).

Thionocarbonate **21**: produced similar observations to thionocarbamate **17** in many reactions. Method A was slightly more effective on **21**, but enone **4** was only obtained as a mixture with an unknown side-product, and the yield of this mixture was still poor (entry 8). Method C initially afforded **4** in poor yield, but this was quickly optimised to 61% (entries 9–11). However, the purity of enone **4** was an issue because silicon-containing byproducts co-eluted in column chromatography. A hexane-MeCN partition was effective in trapping the

byproducts in the hexane, where **4** had a high affinity for MeCN. Following column chromatography, most of the silicon residues had been removed. Frustratingly, the yield of **4** could not be advanced above 61% and mirrored thionocarbamate **17** in this respect.

Acetal **22**: Methods A and D offered no difference in the reaction profile from previous substrates (entries 12 and 23). Method E performed similarly to Method D, but the isolated yield was lower for the same level of impurity (entry 24). As a result, methods D and E were discarded from further trial. Method B appeared viable and delivered acetal **23** in 60–67% yield (entries 13 and 14). Unfortunately, acetal **22** was never fully consumed even with fresh reagents, changes to eqv.'s, concentration, and reaction time. Frequently a yellow residue crept up the reaction condenser and a pungent odour was noticed, especially if the reaction ran for more than 12 h; the identity of this was not established and rinsing it back into solution made no apparent difference. Deoxygenation with $(TMS)_3SiH$ (method C) gave the most consistent results, consuming all the starting material and generating a single product acetal **23**, albeit with similar impurity issues observed using $^n Bu_3GeH$ and $^n Bu_3SnH$. As such method C underwent substantial trialling (entries 15–22). One report from the literature addresses the impurity problem (P. Kraft, C. Weymuth, C. Nussbaumer, *Eur. J. Org. Chem*. **2006**, 1403–1412). They found that treating the crude reaction mixture with an acid wash, followed by TBAF in THF left the byproducts as a thick layer that would not pass through silica gel. Initially, this procedure worked well and provided a 97% yield of acetal **23** with minimal silicon residue (entries 16 and 17). Attempts to use KF as a cheaper alternative were fruitless, likely owing to the poor solubility of KF in organic solvents, and the poor solubility of the silicon byproducts in H_2O.

As the scale of the deoxygenation increased, the modified work-up procedure became less effective. Yields dropped below 90% and fell as low as 52% (entry 21). The problem with this purification method is that the silicon residues, having been treated with the TBAF solution, form a viscous layer which is insoluble under standard work-up conditions. When loaded onto silica gel it has a significant volume at 10 g scale and traps **23** within. This had two negative effects: recovery dropped, and as the product was slow to escape the residue layer the chromatographic band was wider, which in turn allowed other byproducts to co-elute and reduce the purity. At this point it was discovered that acetal **23** could be distilled under high vacuum, which significantly reduced the waste of silica, solvent, TBAF, and time spent on the purification and proved to be reliably better than the TBAF method at scale. Thus, as much as

20 g of acetal **22** was reacted in this procedure as part of a batch that went from alcohol **16** to diol **5** in 62% yield (84 mmol of **16**, 4 steps, averages to 89% per step).

Xanthate **20**: acetal **20**, produced at a later stage, did not readily undergo the same 3- or 4-step deoxygenation process. While pure **20** was deoxygenated with $(TMS)_3SiH$ to give acetal **23** in yields of 75–85%, when a 3-step procedure from alcohol **16** without purification was tested, the deoxygenation failed outright. The outcome suggested a conflict with the impurities being carried through; either the mineral oil from the NaH dispersion, or the remnants of CS_2 that are not volatile enough to be removed under reduced pressure. Since xanthate **19** also experiences mild silica sensitivity, purifying **19** and **20** to allow the deoxygenation to work would be less efficient in many ways and was deemed a worse route to acetal **23**.

To summarise the findings of the deoxygenation investigation: method A was completely incompatible with substrates **19**, **21**, **17**, and **22**. Method B only worked with acetal **22** but failed to completely consume starting material and produce yields above 61%. $^{n}Bu_3GeH$ (method D) and $^{n}Bu_3SnH$ (method E) produced byproducts that were inseparable even after chromatography; since these reagents are either more expensive or more toxic than $(TMS)_3SiH$ (method C), they were not optimised further. Method C produced the best yield for acetal **22** and was optimised to use vacuum distillation to purify **23**, providing excellent yields over multiple steps. Substrates prior to acetalisation could not be deoxygenated in high yield or purity. Despite the low cost of synthesising xanthate **19**, impurities from the reaction meant that extensive purification of both **19** and **20** before deoxygenation were necessary for success. Thus, acetal **22** had a higher multistep process efficiency and was the best substrate despite the relatively high cost of PhOC(S)Cl.

Procedures

Diol 5

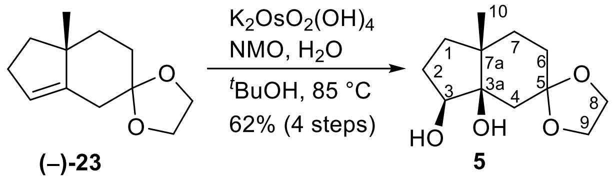

Procedure adapted from literature reports.[9]

Crude alkene (−)-**23** (assumed 60 mmol) was dissolved in ^{t}BuOH (90 mL) and H_2O (30 mL). NMO (14.8 mL, 72.0 mmol, 50 wt% aqueous solution) and $K_2OsO_2(OH)_4$ (221 mg, 0.600 mmol) were added, and the resulting solution was heated at 85 °C for 5 h. After 3 h, an additional portion of NMO (8.0 mL, 39.0 mmol) and $K_2OsO_2(OH)_4$ (50 mg, 0.14 mmol) were added. Upon completion, the solution was cooled to 23 °C, Na_2SO_3 (9.0 g, 71 mmol) and H_2O (15 mL) were added and the resulting mixture stirred for 10 min. The solution was extracted with EtOAc (150 mL, then 3 × 50 mL), saturating the aqueous phase with NaCl each time. The organic layers were dried over $MgSO_4$, filtered, and concentrated under reduced pressure to give a brown oil. A solid was precipitated by glass-scratching and was purified by a series of recrystallisations (Et_2O-hexane) to give diol **5** (11.86 g, 62% over 4 steps) as tan crystals.

TLC: 50% EtOAc-petroleum ether, R_f = 0.14 Vanillin

MP: 97−99 °C (Et_2O-hexane); *Lit.*[9] 70−72 °C

Optical Rotation: $[\alpha]_D^{25}$ = −4.6°, (c = 1.0, $CHCl_3$); *Lit.* [10] $[\alpha]_D^{20}$ = −61.8°, (c = 0.33, CH_2Cl_2)

IR (neat, ATR attachment): $\tilde{\nu}_{max}$ 3482 w br (O-H), 3344 br (O-H), 2940 w (C-H), 2888 w (C-H), 1067 m sh (C-O), 1013 m sh (C-O)

^{1}H NMR (400 MHz, C_6D_6): δ 4.33 (ddd, *J* 8.8, 5.2, 3.2, 1H, H-3), 3.55−3.34 (st, 4H, H-8 and 9), 3.02 (s, 1H, 3°-OH), 2.55 (d, *J* 3.6, 1H, 2°-OH), 1.92 (*ap.* dddd, *J* 14.1, 9.8, 8.5, 4.5, 1H, H-2'), 1.80−1.66 (st, 3H, H-2'' and H-4), 1.64−1.55 (st, 2H, H-1' and 1H of CH_2), 1.53−1.38 (st, 2H), 1.36−1.23 (st, 2H, H-1'' and 1H of CH_2), 1.07 (s, 3H, H-10)

^{13}C NMR (101 MHz, C_6D_6): δ 109.5 (C, C-5), 80.2 (C, C-3a), 76.5 (CH, C-3), 64.3 (CH_2, C-8 or 9), 64.0 (CH_2, C-8 or 9), 42.7 (C, C-7a), 40.5 (CH_2, C-4), 34.7 (CH_2, C-1), 32.9 (CH_2, C-6 or 7), 30.8 (CH_2, C-6 or 7), 29.2 (CH_2, C-2), 21.4 (CH_3, C-10)

LRMS [TOF-EI$^+$]: *m/z* 228 (15%, $[M]^+$), 170 (15), 99 (100)

These data are in agreement with literature reported values. [9]

Trans-hydrindane 6

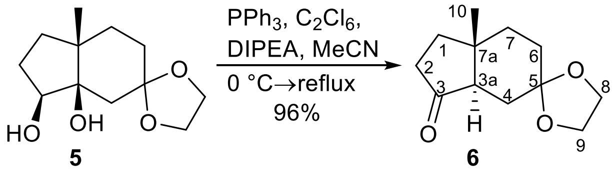

Procedure adapted from a literature report. [9]

A solution of PPh_3 (10.3 g, 39.4 mmol) and C_2Cl_6 (9.33 g, 39.4 mmol) in MeCN (40 mL) was stirred for 20 min. DIPEA (17.0 mL, 97.6 mmol) was added, and the resulting solution was cooled to 0 °C, followed by the addition of a solution of diol **5** (4.50 g, 19.7 mmol) in MeCN (30 mL) over 10 min. After 1 h, TLC (50% EtOAc-petroleum ether) indicated consumption of the starting diol (R_f = 0.14) and formation of the phosphorane intermediate (R_f = 0.55). The reaction mixture was heated at reflux until the intermediate spot had disappeared and a new spot (R_f = 0.50) was formed, at which time the reaction mixture was cooled to 23 °C. The solution was diluted with Et_2O (60 mL) and washed with H_2O (3 × 30 mL). The aqueous layers were further extracted with Et_2O (5 × 30 mL), and all organic extracts were combined and washed with brine (3 × 40 mL), dried over $MgSO_4$, filtered, and concentrated under reduced pressure. The crude material was purified by flash column chromatography (50% Et_2O-petroleum ether) to give ketone **6** (3.97 g, 96%) as a colourless oil that solidifies upon standing.

TLC: 50% EtOAc-petroleum ether, R_f = 0.50 Vanillin

MP: 61−63 °C (Et_2O); *Lit.* [9] 62−64 °C

Optical Rotation: $[\alpha]_D^{25}$ = −85° (c = 0.5, CH_2Cl_2), *Lit.* [10] $[\alpha]_D^{20}$ = −92.6° (c = 1.0, CH_2Cl_2)

IR (neat, ATR attachment): $\tilde{\nu}_{max}$ 2927 w (C-H), 2881 w (C-H), 2854 w (C-H), 1729 sh (C=O)

^{1}H NMR (400 MHz, C_6D_6): δ 3.54−3.38 (st, 4H, H-8 and 9), 2.21 (dd, *J* 12.5, 3.2, 1H, H-3a), 2.15 (*ap.* dt, *J* 13.1, 2.7, 1H, H-4’), 1.92 (*ap.* dt, *J* 9.6, 0.9, 1H, H-7’), 1.91 (*ap.* dd, *J* 9.8, 0.8, 1H, H-7’’), 1.76 (*ap.* td, *J* 13.7, 4.9, 1H, H-6’), 1.63−1.46 (st, 3H, H-2’, 4’’ and 6’’), 1.40−1.29 (st, 2H, H-1’ and 2’’), 1.14 (*ap.* dtd, *J* 12.2, 9.9, 1.0, 1H, H-1’’), 0.51 (s, 3H, H-10)

^{13}C NMR (101 MHz, C_6D_6): *δ* 213.5 (C, C-3), 109.7 (C, C-5), 64.5 (CH_2, C-8 or 9), 64.2 (CH_2, C-8 or 9), 57.0 (CH, C-3a), 38.4 (C, C-7a), 36.0 (CH_2, C-7), 35.6 (CH_2, C-2), 35.4 (CH_2, C-1), 32.2 (CH_2, C-6), 30.6 (CH_2, C-4), 16.6 (CH_3, C-10)

LRMS [TOF-EI$^+$]: *m/z* 210 (15%, $[M]^+$), 181 (15), 154 (15), 139 (10), 112 (15), 99 (100), 86 (90), 79 (40), 67 (65)

These data are in agreement with literature reported values. [9]

Tertiary alcohol 7

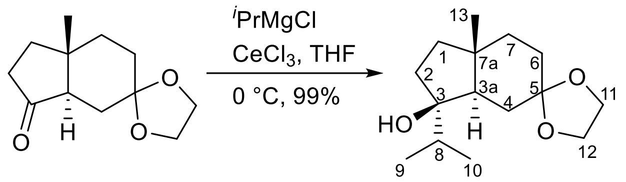

6 7

Procedure adapted from literature reports. [9][11]

$CeCl_3{\cdot}7H_2O$ (25.3 g, 67.8 mmol) was placed in a Schlenk tube with a stirrer bar and heated at 160 °C under vacuum (<10^{-1} mbar) for 2.5 h. **Note:** powdered $CeCl_3$ is easily sucked through tubing, as such the Schlenk tube was angled to reduce this. The tube was cooled to 23 °C, backfilled with argon, and THF (30 mL) was added. The milky suspension was stirred overnight to form a thick slurry. At this time, ketone **6** (8.40g, 39.9 mmol) in THF (40 mL) was added and stirred for a further 30 min, causing a yellowing of the solution. The reaction mixture was then cooled to 0 °C and ^{i}PrMgCl (35 mL, 63.4 mmol, 1.81 M solution in THF) was added over 40 min. Immediately after addition, the cooling bath was removed, and the reaction mixture stirred for a further 20 min. Upon completion, Et_2O (50 mL) and H_2O (50 mL) were added to quench the reaction, then AcOH was added until the phases became clear. The organic phase was washed with H_2O (3 × 30 mL) and the aqueous layers extracted with Et_2O (3 × 100 mL). The combined organic extracts were washed with brine (3 × 60 mL), dried over $MgSO_4$, filtered, and concentrated under reduced pressure to give alcohol **7** (10.0 g, 99%) as cream crystals without a need for further purification.

TLC: 50% EtOAc-petroleum ether, R_f = 0.50 Vanillin

MP: 81−82 °C (Et_2O), *Lit.* [9] 62−64 °C

Optical Rotation: $[\alpha]_D^{25}$ = −13° (c = 0.5, CH_2Cl_2); *Lit.* [10] $[\alpha]_D^{20}$ = −12.6° (c = 1.0, CH_2Cl_2)

IR (neat, ATR attachment): $\tilde{\nu}_{max}$ 3508 br (O-H), 2948 (C-H), 2876 (C-H), 1087 m (C-O)

^{1}H NMR (400 MHz, C_6D_6): δ 3.62–3.56 (m, 2H, H-11 or 12), 3.55–3.50 (m, 2H, H-11 or 12), 1.93 (ddd, *J* 14.2, 11.3, 7.4, 1H), 1.88–1.78 (st, 3H, H-3a and 2H of CH_2), 1.74–1.65 (st, 3H), 1.58–1.46 (st, 4H, H-14, and 3H of CH_2), 1.13 (s, 3H, H-13), 1.04 (*ap.* qd, 1H), 0.91 (d, *J* 6.8, 3H, H-9 or 10), 0.85 (d, *J* 6.8, 3H, H-9 or 10), 0.62 (s, 1H, OH)

^{13}C NMR (101 MHz, C_6D_6): δ 110.9 (C, C-5), 82.9 (C, C-3), 64.5 (CH_2, C-11 or 12), 64.2 (CH_2, C-11 or 12), 51.2 (CH, C-3a), 41.8 (C, C-7a), 39.8 (CH_2), 37.7 (CH_2), 37.6 (CH, C-8), 36.7 (CH_2), 32.3 (CH_2), 32.0 (CH_2), 18.5 (CH_3, C-9 or 10), 18.4 (CH_3, C-13), 17.7 (CH_3, C-9 or 10)

LRMS [TOF-ES$^+$]: *m/z* 237 (100%, $[M{-}OH]^+$), 193 (10, $[M{-}OH{-}^iPr]^+$)

These data are in agreement with literature reported values. [9]

Hajos-Parrish Ketone 15

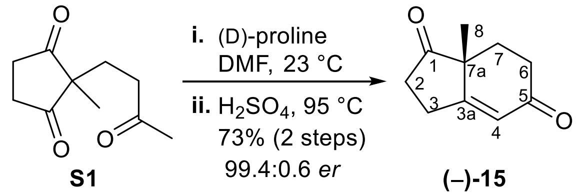

Procedure adapted from a literature report.[12]

(D)-Proline (3.13 g, 27.0 mmol) was dissolved in DMF (*ca.* 250 mL, transferred *via* cannula), and the resulting solution stirred for 1 h at 23 °C, having shielded the system from light with foil. A solution of crude triketone **S1** (assume 0.907 mol) in DMF (*ca.* 110 mL) was added and the resulting mixture stirred for 6 d. Upon consumption of the starting material (50% EtOAc-petroleum ether, R_f (triketone) = 0.26, R_f (ketol) = 0.13) the reaction was heated at 95 °C. Starting at 75 °C, an aliquot of an H_2SO_4-DMF solution (20 mL, 20 mL H_2SO_4 in 60 mL DMF, kept at −20 °C) was added. After 90 min, TLC indicated full conversion and the solvent was removed under reduced pressure. The residue was taken up in EtOAc (400 mL) and washed with $H_2SO_{4\,(aq.)}$ (2 M, 2 × 150 mL), and then $NaHCO_{3\,(aq.)}$ (2 × 180 mL). Furthermore, each aqueous wash was re-extracted with EtOAc (3 × 150 mL). All organic extracts were combined and dried over $MgSO_4$, filtered, and concentrated under reduced pressure to give the crude material as a dark brown oil. Purification by flash column chromatography (50% EtOAc-petroleum ether) gave *ca.* 160 g of an orange oil that solidified. The solid was then recrystallised from Et_2O in a single batch using a seed crystal, to give (−)-**15** (108.6 g, 73% over 2 steps) as pale orange plate crystals.

TLC: 50% EtOAc-petroleum ether, R_f = 0.21 UV / Vanillin

MP: 60−62 °C (Et_2O-hexane); *Lit.*[12] 66 °C (Et_2O-hexane)

Optical Rotation: $[\alpha]_D^{25}$ = −341°, (c = 1.0, toluene); Lit. [12] $[\alpha]_D^{25}$ = +347.5−349°, (c = 1.0, toluene, opposite enantiomer)

IR (neat, ATR attachment): $\tilde{\nu}_{max}$ 2957 w (C-H), 2878 w (C-H), 1741 st (C=O), 1699 w (C=C), 1650 st (C=O)

^{1}H NMR (400 MHz, $CDCl_3$): δ 5.93 (d, *J* 2.5, 1H, H-4), 2.93 (dddd, *J* 17.0, 11.0, 9.9, 2.4, 1H, H-3'), 2.83−2.66 (st, 2H, H-2' and 3''), 2.55−2.34 (st, 3H, H-2'', 6' and 6''), 2.07 (ddd, *J* 13.5, 5.2, 2.2, 1H, H-7'), 1.81 (*ap.* td, *J* 13.7, 5.4, 1H, H-7''), 1.32 (s, 3H, H-8)

^{13}C NMR (101 MHz, $CDCl_3$): δ 216.6 (C, C-1), 198.2 (C, C-5), 169.8 (C, C-3a), 123.9 (CH, C-4), 48.8 (C, C-7a), 35.9 (CH_2, C-2), 33.0 (CH_2, C-6), 29.3 (CH_2, C-7), 26.9 (CH_2, C-3), 20.6 (CH_3, C-8)

LRMS [TOF-ES$^+$]: m/z 165 (20%, $[M+H]^+$), 123 (100, $[M-H_2CCO]^+$)

HPLC: ratio of enantiomers = 99.4:0.6 (20% MeOH: 80% CO_2, 4 mL/min, 40 °C, T_r = 1.58 min (T_r (+)-isomer = 1.74 min)

These data are in agreement with literature reported values. [12]

Alcohol 16

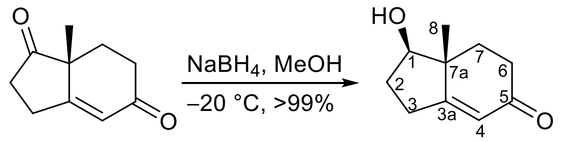

(−)-15 **16**

Procedure adapted from a literature report.[13]

Ketone (−)-**15** (10.0 g, 6.09 mmol) was dissolved in MeOH (61 mL) and the resulting solution cooled to −20 °C, as measured by an internal thermometer. $NaBH_4$ (693 mg, 18.3 mmol) was added portionwise (1 portion per 5 min) over 42 min such that the internal temperature did not exceed −20 °C. The solvent was removed under reduced pressure, and the residue taken up in EtOAc (40 mL) and H_2O (30 mL). The resulting solution was brought to neutral pH (universal indicator paper) by the addition of 1 M $HCl_{(aq.)}$, turning from orange to yellow. The aqueous phase was separated, saturated with NaCl, and further extracted with EtOAc (6 × 60 mL). The combined organic layers were dried with $MgSO_4$, filtered, and concentrated under

reduced pressure. The crude material was purified by flash column chromatography (70% → 100% EtOAc-petroleum ether) to give alcohol **16** (10.1 g, >99%) as a cream solid.

TLC: 80% EtOAc-petroleum ether, R_f = 0.22 UV / Vanillin

Optical Rotation: $[\alpha]_D^{25}$ = −73°, (c = 1.0, $CHCl_3$); *Lit.*[14] $[\alpha]_D^{25}$ = +90°, (c = 1.0, C_6H_6, opposite enantiomer)

IR (neat, ATR attachment): $\tilde{\nu}_{max}$ 3341 br (O-H), 2963 w (C-H), 2936 w (C-H), 1633 st (C=O), 1076 st (C-O)

^{1}H NMR (400 MHz, $CDCl_3$): δ 5.76 (br s, 1H, H-4), 3.83 (ddd, *J* 10.2, 7.4, 4.6, 1H, H-1), 2.69 (*ap.* ddtd, *J* 19.7, 11.6, 2.4, 0.7, 1H, H-3'), 2.58−2.28 (st, 4H, H-3'', 6', 6'' and O*H*), 2.16−2.05 (st, 2H, H-2' and 7'), 1.89−1.69 (st, 2H, H-2'' and 7''), 1.12 (d, *J* 0.7, 3H, H-8)

^{13}C NMR (101 MHz, $CDCl_3$): δ 199.6 (C, C-5), 175.6 (C, C-3a), 123.5 (CH, C-4), 80.7 (CH, C-1), 45.3 (C, C-7a), 34.2 (CH_2, C-7), 33.4 (CH_2, C-6), 29.2 (CH_2, C-2), 26.6 (CH_2, C-3), 15.2 (CH_3, C-8)

LRMS [TOF-EI$^+$]: *m/z* 166 (40%, $[M]^+$), 109 (100, $[M-HOCHCH_2CH_2]^+$)

The data are in agreement with previously recorded values.[15]

Thionocarbamate 17

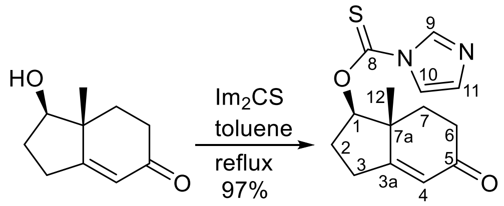

16 17

Procedure adapted from a literature report.[16]

Alcohol **16** (101 mg, 0.61 mmol) and thiocarbonyldiimidazole (129 mg, 0.72 mmol) were dissolved in toluene (6 mL) and the resulting mixture stirred for 5 min at 23 °C. The solution was then heated at reflux for 2 h, turning from yellow to orange. Upon completion, the reaction mixture was cooled to 23 °C and the solvent removed under reduced pressure. The crude material was purified directly by flash column chromatography (90% EtOAc-petroleum ether) to give thionocarbamate **17** (163 mg, 97%) as a yellow solid.

TLC: 80% EtOAc-petroleum ether, R_f = 0.08 UV / $KMnO_4$

MP: 86−88 °C (EtOAc-petroleum ether)

Optical Rotation: $[\alpha]_D^{25}$ = −48°, (c = 1.0, $CHCl_3$)

IR (neat, ATR attachment): $\tilde{\nu}_{max}$ 3142 w (C-H), 2962 w (C-H), 2927 w (C-H), 1760 w, 1667 st (C=O)

^{1}H NMR (400 MHz, $CDCl_3$): δ 8.32 (t, *J* 1.7, 0.9, 1H, H-9, 10 or 11), 7.61 (*ap.* t , *J* 1.4, 1H, H-9, 10 or 11), 7.09 (dd, *J* 1.6, 0.8, 1H, H-9, 10 or 11), 5.85 (br s, 1H, H-4), 5.52 (dd, *J* 10.0, 7.3, 1H, H-1), 2.96−2.78 (m, 1H), 2.64−2.46 (st, 3H, H-6' and 2H of CH_2), 2.40 (dddd, *J* 17.9, 5.0, 2.1, 0.8, 1H, H-6''), 2.13 (ddd, *J* 13.1, 5.3, 2.2, 1H, H-7'), 2.04−1.93 (st, 2H, H-7'' and 1H of CH_2), 1.35 (s, 3H, H-12)

^{13}C NMR (101 MHz, $CDCl_3$): δ 197.9 (C, C-5), 183.6 (C, C-8), 170.7 (C, C-3a), 136.8 (CH, C-9, 10 or 11), 131.2 (CH, C-9, 10 or 11), 124.2 (CH, C-4), 117.9 (CH, C-9, 10 or 11), 89.4 (CH, C-1), 45.4 (C, C-7a), 34.5 (CH_2, C-7), 33.1 (CH_2, C-6), 26.7 (CH_2, C-2 or 3), 25.8 (CH_2, C-2 or 3), 17.8 (CH_3, C-12)

HRMS [TOF-ES$^+$]: calculated for ($[M+H]^+$, $C_{14}H_{17}N_2O_2S$): 277.1011, found: 277.1019

Thionocarbonate acetal 18

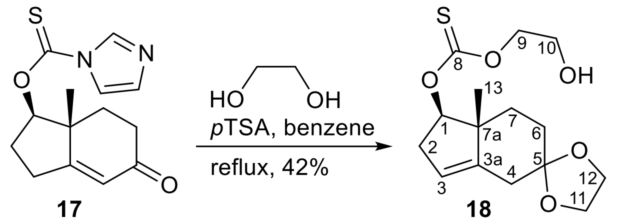

17 18

Procedure adapted from a literature report. [9]

Thionocarbamate **17** (52 mg, 0.18 mmol) was dissolved in benzene (10 mL), followed by addition of ethylene glycol (0.10 mL, 1.8 mmol,) and *p*TSA (42 mg, 0.22 mmol). A Dean-Stark trap and condenser were fitted, and the solution was heated at reflux for 3 h, resulting in a pale-yellow solution. Upon completion, the solution was cooled to 23 °C and $NaHCO_{3\,(aq.)}$ (15 mL) was added while stirring for 5 min. The organic phase was separated, and the aqueous phase further extracted with EtOAc (3 × 10 mL). The combined organic extracts were washed with brine (3 × 10 mL), dried over $MgSO_4$, filtered, and concentrated under reduced pressure. The crude material was purified by flash column chromatography (50% EtOAc-petroleum ether) to give thionocarbonate **18** (24 mg, 42%) as a cream solid.

TLC: 80% EtOAc-petroleum ether, R_f = 0.58 UV / $KMnO_4$

IR (neat, ATR attachment): $\tilde{\nu}_{max}$ 3391 br (O-H), 2935 n (C-H), 2887 n (C-H), 1806 w, 1775 w, 1746 w, 1454 w, 1231 m, 1088 st (C-O), 1062 m

^{1}H NMR (400 MHz, $CDCl_3$): δ 5.55 (*ap.* t, *J* 7.9, 1H, H-1), 5.27 (dd , *J* 2.9, 1.4, 1H, H-3), 4.55 (ddd, *J* 6.0, 3.0, 1.2, 2H, H-9), 3.99−3.91 (st, 6H, H-10, 11, and 12), 2.89 (ddt, *J* 10.0, 7.0, 2.3, 1H, H-2'), 2.49−2.29 (st, 3H, H-2'' and 2H of CH_2), 1.97 (s, 1H, OH), 1.92−1.59 (st, 4H), 1.11 (s, 3H, H-13)

^{13}C NMR (101 MHz, $CDCl_3$): δ 195.7 (C, C-8), 145.0 (C, C-3a), 119.0 (CH, C-3), 109.1 (C, C-5), 91.1 (CH, C-1), 74.2 (CH_2, C-9), 64.64** (CH_2, C-11 or 12), 64.58** (CH_2, C-11 or 12), 60.8 (CH_2, C-10), 47.1 (C, C-7a), 36.8 (CH_2), 36.1 (CH_2), 35.4 (CH_2, C-2), 31.3 (CH_2), 16.5 (CH_3, C-13)

***2 d.p. provided to distinguish peaks that would otherwise be the same at 1 d.p.*

Xanthate 19

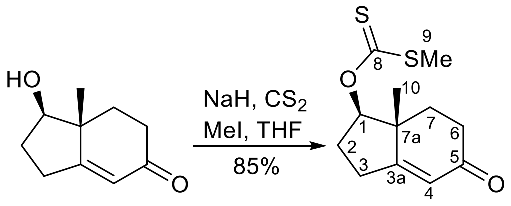

16 19

Procedure adapted from a literature report.[17]

A suspension of NaH (189 mg, nominally 3.91 mmol, 60% mineral oil dispersion) in THF (8 mL) was cooled to 0 °C, and a solution of alcohol **16** (501 mg, 3.01 mmol) in THF (7 mL) was added. The flask was raised from the cooling bath immediately after, followed by the addition of CS_2 (2.2 mL, 36.1 mmol). After 20 h, MeI (0.56 mL, 9.03 mmol) was added and the resulting solution was stirred until TLC indicated completion. Notably, the solution turns from a pale, cloudy yellow to bright red after the addition of CS_2, then to brown overnight, and back to red after the addition of MeI. Upon completion, the reaction mixture was quenched by the addition of H_2O (**Careful!**), then partitioned with Et_2O (25 mL). The organic phase was washed with H_2O (3 × 15 mL), then the combined aqueous layers were re-extracted with Et_2O (1 × 25 mL). The organic layers were then washed with brine (3 × 10 mL), dried over $MgSO_4$, filtered, and concentrated under reduced pressure. The crude material was purified by flash column chromatography (40% Et_2O-petroleum ether) to give xanthate **19** (659 mg, 85%) as an orange oil.

TLC: 50% EtOAc-petroleum ether, R_f = 0.56 UV / Vanillin

Optical Rotation: $[\alpha]_D^{25}$ = −4.1°, (c = 1.0, $CHCl_3$)

IR (neat): $\tilde{\nu}_{max}$ 2925 w (C-H), 1666 st (C=O), 1200 st, 1063 st

^{1}H NMR (400 MHz, $CDCl_3$): δ 5.81 (dt, *J* 2.1, 1.1, 1H, H-4), 5.63−5.57 (m, 1H, H-1), 2.87−2.75 (m, 1H, H-6'), 2.57 (s, 3H, H-9), 2.55−2.43 (st, 3H, H-2', H-3', and H-6''), 2.37 (dddd, *J* 17.9, 5.2, 2.1, 0.8, 1H, H-3''), 2.10 (ddd, *J* 13.1, 5.3, 2.1 , 1H, H-7'), 1.99−1.87 (st, 2H, H-2'' and H-7''), 1.29 (d, *J* 0.7, 3H, H-10)

^{13}C NMR (101 MHz, $CDCl_3$): δ 215.9 (C, C-8), 198.6 (C, C-5), 172.1 (C, C-3a), 123.9 (CH, C-4), 89.3 (CH, C-1), 45.6 (C, C-7a), 34.5 (CH_2, C-7), 33.3 (CH_2, C-6), 26.9 (CH_2, C-3), 26.0 (CH_2, C-2), 19.4 (CH_3, C-9), 17.5 (CH_3, C-10)

HRMS [TOF-ES$^+$]: calculated for ($[M+H]^+$, $C_{12}H_{17}O_2S_2$): 257.0670, found: 257.0674

Xanthate acetal 20

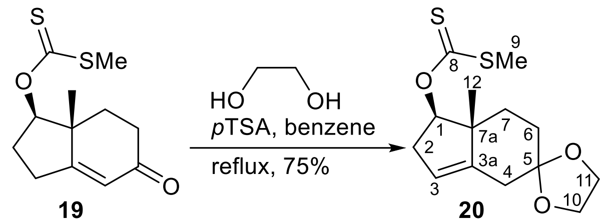

19 20

Procedure adapted from a literature report. [9]

Enone **19** (659 mg, 2.57 mmol) was dissolved in benzene (17 mL) with ethylene glycol (0.72 mL, 12.9 mmol) and *p*TSA (73 mg, 0.39 mmol). A Dean-Stark apparatus was fitted, and the trap filled with benzene. The reaction mixture was heated at reflux for 2 h at which point TLC indicated completion. The reaction mixture was cooled, neutralised with $NaHCO_{3\ (aq.)}$, and partitioned between Et_2O (20 mL) and H_2O (20 mL). The aqueous layer was re-extracted with Et_2O (1 × 20 mL), then the combined organic layers were washed with brine (3 × 50 mL), dried over $MgSO_4$, filtered, and concentrated under reduced pressure. The crude material was purified by flash column chromatography (10% EtOAc-petroleum ether) to give acetal **20** (578 mg, 75%) as a pale orange oil that solidifies on standing.

TLC: 25% EtOAc-petroleum ether, R_f = 0.50 UV / Vanillin

MP: 69−72 °C (EtOAc-petroleum ether)

Optical Rotation: $[\alpha]_D^{25}$ = −32°, (c = 1.0, $CHCl_3$)

IR (neat, ATR attachment): $\tilde{\nu}_{max}$ 2963 n (C-H), 2938 n (C-H), 2859 n (C-H), 2888 n (C-H), 1447 w, 1422 w, 1207 m, 1091 m (C-O), 1062 st (C-O), 1043 m (C-O), 798 m

^{1}H NMR (400 MHz, CDCl$_3$): δ 5.86 (*ap.* t, *J* 7.7, 1H, H-1), 5.29−5.26 (m, 1H, H-3), 3.99−3.90 (st, 4H, H-10 and H-11), 2.95−2.86 (m, 1H, H-2’), 2.56 (s, 3H, H-9), 2.45−2.35 (st, 3H, H-2’’ and 2H of CH$_2$), 1.91−1.78 (st, 2H, H-7’ and 1H of CH$_2$), 1.73−1.64 (st, 2H, H-7’’ and 1H of CH$_2$), 1.14 (d, *J* 0.6, 3H, H-12)

^{13}C NMR (101 MHz, CDCl$_3$): δ 215.8 (C, C-8), 145.0 (C, C-3a), 119.1 (CH, C-3), 109.1 (C, C-5), 91.3 (CH, C-1), 64.64** (CH$_2$, C-10 or 11), 64.59** (CH$_2$, C-10 or 11), 47.6 (C, C-7a), 36.8 (CH$_2$, C-4 or 6), 36.2 (CH$_2$, C-7), 35.7 (CH$_2$, C-2), 31.3 (CH$_2$, C-4 or 6), 19.1 (CH$_3$, C-9), 16.7 (CH$_3$, C-12)

***2 d.p. provided to distinguish peaks that would otherwise be the same at 1 d.p.*

HRMS [TOF-ES$^+$]: *m/z* calculated for ([M+H]$^+$, $C_{14}H_{20}O_3S_2$): 301.0932, found: 301.0938

O-Phenyl thionocarbonate 21 (and carbonate S2)

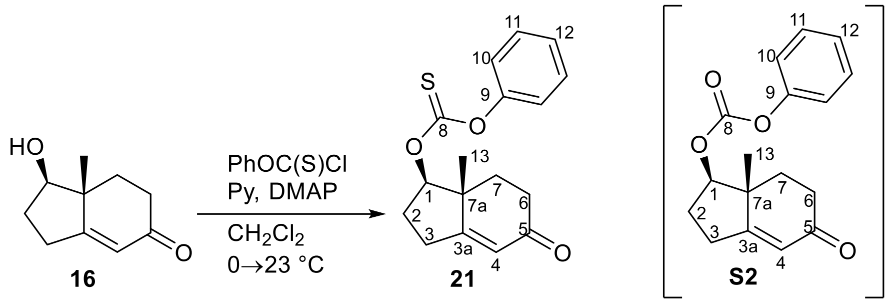

Procedure adapted from a literature report.[18]

Alcohol **16** (14.00 g, 84.23 mmol) was dissolved in CH$_2$Cl$_2$ (*ca*. 168 mL, transferred *via* cannula), followed by the addition of DMAP (1.00 g, 8.40 mmol) and pyridine (27 mL, 337 mmol). The solution was cooled to 0 °C and *O*-phenyl chlorothionoformate (14.0 mL, 101 mmol) was added *via* syringe pump over 1 h, causing the solution to turn yellow from colourless. The reaction mixture was allowed to warm in the cooling bath to 23 °C. After 4 h, the solvent was removed under reduced pressure, and the residue taken up in Et$_2$O (100 mL) then washed with H$_2$O (3 × 50 mL). The aqueous phases were re-extracted with Et$_2$O (4 × 50 mL) and the combined organic layers were then washed with brine (3 × 50 mL), dried over MgSO$_4$, filtered, and concentrated under reduced pressure to give an orange oil. The crude thionocarbonate was used without further purification.

An analytically pure sample was obtained by flash column chromatography (10 → 20% EtOAc-petroleum ether) to give thionocarbonate **21** as a viscous orange oil. In purifying with silica gel, carbonate **S2** was obtained as a mixture with thionocarbonate **21** (orange oil).

21:

TLC: 80% EtOAc-petroleum ether, R_f = 0.69 UV / Vanillin

Optical Rotation: $[\alpha]_D^{25}$ = +39°, (c = 1.0, $CHCl_3$)

IR (neat): $\tilde{\nu}_{max}$ 2934 w (C-H), 1667 sh (C=O), 1189 st

^{1}H NMR (400 MHz, $CDCl_3$): *δ* 7.45−7.39 (m, 2H, H-10), 7.33−7.27 (m, 1H, H-12), 7.14−7.08 (m, 2H, H-11), 5.83 (*ap.* t, *J* 1.6, 1H, H-4), 5.34−5.28 (m, 1H, H-1), 2.90−2.77 (m, 1H, H-3'), 2.62−2.46 (st, 3H, H-2',3'' and 6'), 2.41 (dddd, *J* 17.9, 5.3, 2.1, 0.9, 1H, H-6''), 2.17 (ddd, *J* 13.1, 5.3, 2.1, 1H, H-7'), 2.07−1.92 (st, 2H, H-2'' and 7''), 1.28 (d, *J* 0.7, 3H, H-13)

^{13}C NMR (101 MHz, $CDCl_3$): *δ* 198.3 (C, C-5), 194.7 (C, C-8), 171.7 (C, C-3a), 153.4 (C, C-9), 129.7 (CH, C-10), 126.8 (CH, C-12), 123.9 (CH, C-4), 121.9 (CH, C-11), 90.1 (CH, C-1), 45.2 (C, C-7a), 34.3 (CH_2, C-7), 33.2 (CH_2, C-6), 26.7 (CH_2, C-3), 25.6 (CH_2, C-2), 17.2 (CH_3, C-13)

LRMS [TOF-ES$^+$]: *m/z* 303 (85%, $[M+H]^+$), 209 (50, $[M−OPh]^+$), 149 (100, $[M−PhOCSO]^+$)

HRMS [TOF-ES$^+$]: calculated for ($[M+H]^+$, $C_{17}H_{19}O_3S$): 303.1055, found: 303.1046

S2:

***S2** was isolated as a mixture with **21**, but only peaks corresponding to the former are listed.*

TLC: 25% EtOAc-petroleum ether, R_f = 0.19 UV / Vanillin

^{1}H NMR (400 MHz, $CDCl_3$): *δ* 7.47–7.35 (m, 2H, H-10), 7.30–7.22 (m, 1H, H-12), 7.23–7.15 (m, 2H, H-11), 5.84 (*ap.* t, *J* 2.2, 1H, H-4), 4.80 (dd, *J* 9.6, 7.9, 1H, H-1), 2.82 (ddt, *J* 18.9, 11.3, 2.4, 1H), 2.63–2.34 (st, 4H), 2.17 (ddd, *J* 13.1, 5.3, 2.1, 1H), 2.11–1.91 (st, 2H), 1.28 (d, *J* 0.6, 3H, H-13)

^{13}C NMR (101 MHz, $CDCl_3$): *δ* 198.6 (C, C-5), 172.1 (C, C-3a), 153.5 (C, C-9), 151.1 (C, C-8), 129.7 (CH, C-10), 126.4 (CH, C-12), 124.0 (CH, C-4), 121.1 (CH, C-11), 85.4 (CH, C-1), 44.9 (C, C-7a), 34.4 (CH_2, C-7), 33.3 (CH_2, C-6), 26.6 (CH_2, C-3), 26.3 (CH_2, C-2), 16.7 (CH_3, C-13)

LRMS [TOF-EI$^+$]: *m/z* 286 (5%, $[M]^+$), 149 (80, $[M−PhOCSO]^+$), 94 (100)

Thionocarbonate acetal 22 (and carbonate S3)

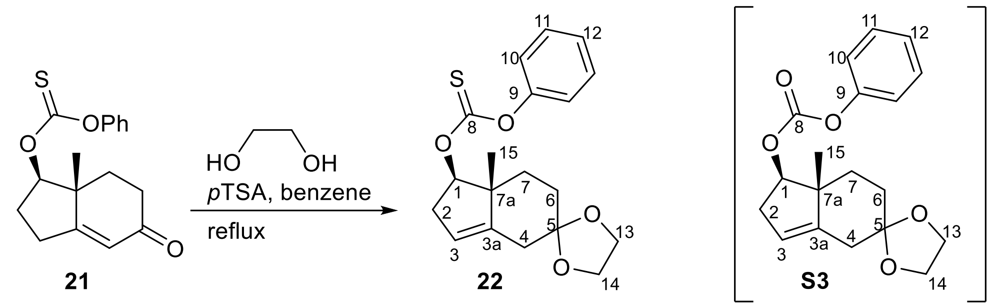

Procedure adapted from a literature report. [9]

Crude enone **21** (assumed 84.2 mmol) was dissolved in benzene (250 mL), followed by the addition of ethylene glycol (23.0 mL, 420 mmol) and *p*TSA (2.50 g, 13.2 mmol). A Dean-Stark trap and condenser were fitted, and the solution was heated at reflux for 3 d. After 1.5 d, an additional portion of ethylene glycol (6.0 mL, 108 mmol) and *p*TSA (0.50 g, 2.64 mmol) was added. Upon completion, the mixture was concentrated under reduced pressure and poured into a separating funnel with Et_2O (100 mL), then washed with $NaHCO_{3\,(aq.)}$ (3 × 50 mL). The aqueous phase was further extracted with Et_2O (1 × 50 mL) and the combined organic layers were washed with brine (3 × 50 mL), dried over $MgSO_4$, filtered, and concentrated under reduced pressure to give the crude acetal as an orange solid which was used without further purification.

An analytically pure sample was obtained by a series of recrystallisations (Et_2O-hexane) to give acetal **22** as cream crystals. When purified with silica gel, carbonate **S3** was also isolated as a white solid.

22:

TLC: 20% EtOAc-petroleum ether, R_f = 0.33 UV / Vanillin

MP: 93−94 °C (Et_2O)

Optical Rotation: $[\alpha]_D^{25}$ = −7.7°, (c = 1.0, $CHCl_3$)

IR (neat, ATR attachment): $\tilde{\nu}_{max}$ 2949 w (C-H), 2931 w (C-H), 2880 w (C-H), 1296 m, 1201 st

^{1}H NMR (400 MHz, $CDCl_3$): δ 7.44−7.38 (m, 2H, H-10), 7.31−7.26 (m, 1H, H-12), 7.14−7.09 (m, 2H, H-11), 5.57 (*ap.* t, *J* 8.0, 1H, H-1), 5.31−5.28 (m, 1H, H-3), 3.96 (*ap.* qd, *J* 4.9, 2.1, 4H, H-13 and 14), 3.02−2.93 (m, 1H, H-2’), 2.51−2.38 (st, 3H, H-2’’, 4’ and 4’’), 1.96−1.89 (m, 1H, H-7’), 1.84 (dd, *J* 14.8, 4.5, 1H, H-6’), 1.77−1.68 (st, 2H, H-6’’ and 7’’), 1.14 (s, 3H, H-15)

^{13}C NMR (101 MHz, $CDCl_3$): δ 195.1 (C, C-8), 153.6 (C, C-9), 145.0 (C, C-3a), 129.6 (CH, C-10), 126.6 (CH, C-12), 122.1 (CH, C-11), 118.9 (CH, C-3), 109.1 (C, C-5), 92.0 (CH, C-1), 64.7 (CH_2, C-13 or 14), 64.6 (CH_2, C-13 or 14), 47.2 (C, C-7a), 36.8 (CH_2, C-2 or 4), 36.2 (CH_2, C-7), 35.4 (CH_2, C-2 or 4), 31.3 (CH_2, C-6), 16.5 (CH_3, C-15)

LRMS [TOF-ES$^+$]: *m/z* 369 (80%, $[M+Na]^+$), 347 (100, $[M+H]^+$), 325 (20, $[M-OCH_2CH_2]^+$), 193 (80, $[M-PhOCSO+H]^+$)

HRMS [TOF-ES$^+$]: calculated for ($[M+Na]^+$, $C_{19}H_{22}O_4SNa$): 369.1137, found: 369.1145

S3:

TLC: 25% EtOAc-petroleum ether, R_f = 0.29 UV / Vanillin

IR (neat): $\tilde{\nu}_{max}$ 2947 w (C-H), 1756 st (C=O), 1738 m (C=O), 1593 w (C=C), 1492 w (C=C), 1247 st, 1206 st, 1091 st (C-O)

^{1}H NMR (400 MHz, $CDCl_3$): δ 7.46–7.32 (m, 2H, H-11), 7.24 (tt, *J* 7.5, 1.1, 1H, H-12), 7.21–7.14 (m, 2H, H-10), 5.28 (m, 1H, H-3), 5.05 (*ap.* t, *J* 8.2, 1H, H-1), 3.99–3.86 (st, 4H, H-13 and 14), 2.85–2.75 (m, 1H, H-2’), 2.49 (*ap.* dddt, *J* 15.4, 8.0, 3.4, 1.8, 1H, H-2’’), 2.40 (br s, 2H, H-4), 1.98–1.87 (m, 1H, H-7’), 1.83 (dd, *J* 14.0, 4.3, 1H, H-6’), 1.76–1.61 (st, 2H, H-6’’ and H-7’’), 1.13 (s, 3H, H-15)

^{13}C NMR (101 MHz, $CDCl_3$): δ 153.8 (C, C-8), 151.3 (C, C-9), 145.1 (C, C-3a), 129.6 (CH, C-11), 126.1 (CH, C-12), 121.2 (CH, C-10), 118.9 (CH, C-3), 109.1 (C, C-5), 87.3 (CH, C-1), 64.7 (CH_2, C-13 or 14), 64.6 (CH_2, C-13 or 14), 46.7 (C, C-7a), 36.8 (CH_2, C-4), 36.1 (CH_2, C-7), 35.9 (CH_2, C-2), 31.3 (CH_2, C-6), 16.0 (CH_3, C-15)

LRMS [TOF-ES$^+$]: *m/z* 353 (25%, $[M+Na]^+$), 331 (100, $[M+H]^+$), 193 (20, $[M-PhOCO_2]^+$)

Acetal 23

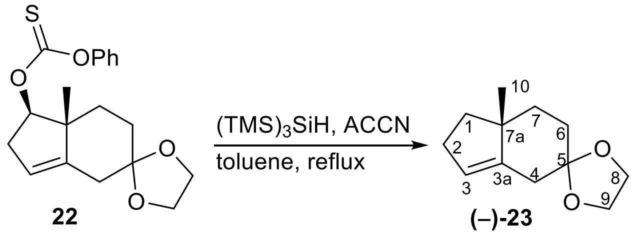

Procedure adapted from a literature report.[19]

Crude thionocarbonate **22** was divided between three flasks for practical reasons. A representative procedure for one of these batches follows:

Thionocarbonate **22** (*ca*. 28.9 mmol) was dissolved in wet toluene (290 mL), followed by the addition of $(TMS)_3SiH$ (10.5 mL, 34.7 mmol), and the resulting solution was degassed for 50 min. At this time ACCN (706 mg, 2.89 mmol) was added and the resulting mixture heated at reflux for 45 min. Upon cooling to 23 °C, the solvent was removed under reduced pressure and the residue taken up in Et_2O (80 mL) and washed with $HCl_{(aq.)}$ (1 M, 3 × 50 mL), then $NaOH_{(aq.)}$ (1 M, 3 × 50 mL). The aqueous phases were re-extracted with Et_2O (3 × 30 mL, 1 × 30 mL respectively) and then the combined organic extracts were washed with brine (3 × 80 mL), dried over $MgSO_4$, filtered, and concentrated under reduced pressure. The residue was distilled (60 °C, 10^{-1} mbar) to give acetal (−)-**23**, typically with minor silicon contaminants, as a yellowish oil (20.11 g total) which was used in the next step without further purification.

TLC: 20% EtOAc-petroleum ether, R_f = 0.33 UV / Vanillin
Optical Rotation: $[\alpha]_D^{25}$ = −27° (c = 1.0, CH_2Cl_2); *Lit.* [10] $[\alpha]_D^{20}$ = −18.7° (c = 0.83, CH_2Cl_2)
IR (neat): $\tilde{\nu}_{max}$ 2937 n (C-H), 1089 m (C-O)
^{1}H NMR (400 MHz, $CDCl_3$): δ 5.29 (d, *J* 2.2, 1H, H-3), 3.99−3.89 (st, 4H, H-8 and 9), 2.42 (dd, *J* 13.5, 2.5, 1H), 2.39−2.22 (st, 3H), 1.88−1.75 (st, 2H), 1.73−1.63 (st, 3H), 1.58−1.48 (m, 1H), 1.05 (s, 3H, H-10)
^{13}C NMR (101 MHz, $CDCl_3$): δ 146.4 (C, C-3a), 122.6 (CH, C-3), 109.9 (C, C-5), 64.6 (CH_2, C-8 or 9), 64.5 (CH_2, C-8 or 9), 45.1 (C, C-7a), 40.3 (CH_2), 37.7 (CH_2), 36.2 (CH_2), 31.9 (CH_2), 30.5 (CH_2), 22.3 (CH_3, C-10)
LRMS [TOF-EI$^+$]: *m/z* 194 (75%, $[M]^+$), 165 (40, $[M{-}CHO]^+$), 99 (100), 91 (85), 77 (80), 55 (35)
These data are in agreement with literature reported values. [9]

Benzyl hydrindanone 24

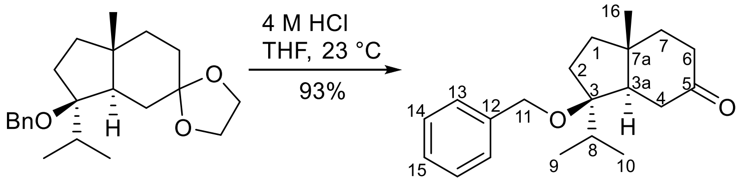

S4 24

Procedure adapted from a literature report. [9]
Acetal **S4** (10.27 g, 29.81 mmol) was dissolved in wet THF (15 mL) in air, and an aliquot of $HCl_{(aq.)}$ (15 mL of a freshly prepared 4 M solution) was added. The resulting solution was

stirred vigorously overnight, with some additional portions of $HCl_{(aq.)}$ (total volume added 6 mL) added. Upon completion, the reaction was diluted with Et_2O (40 mL) washed with $NaHCO_{3\,(aq.)}$ (3 × 15 mL). The aqueous phase was re-extracted with Et_2O (3 × 40 mL), then the combined organics were washed with brine (3 × 30 mL), then dried over $MgSO_4$, filtered, and concentrated under reduced pressure. A solid was precipitated from solution by the addition of hexane (2 mL) and scratching. This was then recrystallised from hexane with a seed crystal to give ketone **24** (8.357 g, 93%) as white needle crystals.

TLC: 25% Et_2O-petroleum ether, R_f = 0.32 Vanillin
MP: 75−78 °C (hexane)
Optical Rotation: $[\alpha]_D^{25}$ = +13° (c = 0.5, CH_2Cl_2); *Lit.* [10] $[\alpha]_D^{20}$ = +15.6° (c = 1.05, CH_2Cl_2)
IR (neat, ATR attachment): $\tilde{\nu}_{max}$ 2939 n (C-H), 2894 w (C-H), 2865 n (C-H), 1706 m (C=O), 1048 st (C-O)
^{1}H NMR (400 MHz, $CDCl_3$): δ 7.38−7.29 (st, 4H, H-13 and 14), 7.28−7.22 (m, 1H, H-15), 4.42 (s, 2H, H-11), 2.77 (dd, *J* 16.0, 14.5, 1H, H-4'), 2.53−2.21 (st, 5H, H-4'', H-8, and 3H of CH_2), 1.94−1.80 (st, 3H, H-3a, 2H of CH_2), 1.71 (dd, J 12.0, 7.7, 1H), 1.52 (*ap.* td, J 12.6, 5.6, 1H), 1.29−1.16 (m, 1H), 1.21 (s, 3H, H-16), 0.95 (d, J 6.8, 3H, H-9 or 10), 0.92 (d, J 6.9, 3H, H-9 or 10)
^{13}C NMR (101 MHz, $CDCl_3$): δ 213.7 (C, C-5), 139.9 (C, C-12), 128.3 (CH, C-13 or 14), 127.1 (CH, C-15), 126.8 (CH, C-13 or 14), 87.4 (C, C-3), 62.6 (CH_2, C-11), 49.6 (CH, C-3a), 41.7 (C, C-7a), 41.6 (CH_2, C-4), 39.9 (CH_2), 37.7 (CH_2), 37.3 (CH_2), 35.1 (CH_2), 33.0 (CH, C-8), 18.3 (CH_3, C-9 or 10), 18.1 (CH_3, C-16), 17.8 (CH_3, C-9 or 10)
LRMS [TOF-ES$^+$]: *m/z* 323 (100%, $[M+Na]^+$), 207 (5), 193 (30, $[M-OBn]^+$)
These data are in agreement with literature reported values. [10]

Bromoenal 25

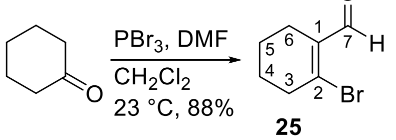

25

Procedure adapted from a literature report.[20]

PBr_3 (0.75 mL, 7.98 mmol) was added to a solution of DMF (0.75 mL, 9.69 mmol) in CH_2Cl_2 (25 mL) over 5 min at 0 °C. The solution was stirred for 30 min at 23 °C until a white suspension was formed, then a solution of cyclohexanone (0.3 mL, 2.9 mmol) in CH_2Cl_2 (5 mL) was added. The reaction was stirred for 24 h after which the mixture was poured into ice-water and extracted with CH_2Cl_2 (5 × 20 mL). The organic layers were washed with brine (3 × 30 mL), dried over anhydrous Na_2SO_4, filtered, and concentrated under reduced pressure. The crude material was purified by flash column chromatography (30% Et_2O-petroleum ether) gave bromoenal **25** (0.48 g, 88%) as a colourless oil.

IR (neat): $\tilde{\nu}_{max}$ 2938 (C-H), 1676 (C=O), 1616 (C=C)
^{1}H NMR (300 MHz, $CDCl_3$): δ 10.02 (s, 1H, H-7), 2.74 (tt, *J* 6.2, 2.3, 2H), 2.28 (tt, *J* 5.8, 2.3, 2H), 1.86–1.63 (st, 4H)
^{13}C NMR (101 MHz, $CDCl_3$): δ 193.9 (CH, C-7), 143.7 (C, C-2), 135.4 (C, C-1), 39.0 (CH_2), 25.1 (CH_2), 24.4 (CH_2), 21.2 (CH_2)
These data are in agreement with literature reported values.[20]

Enynal 26

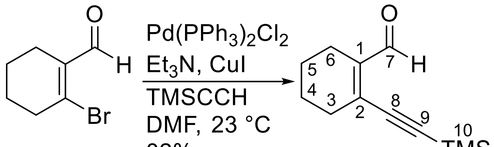

25 **26**

Procedure adapted from a literature report. [20]
A mixture of bromoenal **25** (0.44 g, 2.32 mmol), $Pd(PPh_3)_2Cl_2$ (0.16 g, 0.23 mmol) and CuI (0.09 g, 0.47 mmol) in DMF (8 mL) was stirred for 10 min. The flask was then cooled to 0 °C and TMS acetylene (0.65 mL, 4.57 mmol) and Et_3N (0.80 mL, 5.74 mmol) were added. The reaction was stirred for 1 h at 23 °C. Upon completion, the reaction mixture was filtered over celite with Et_2O (15 mL), then the filtrate was washed with brine (3 × 10 mL) and extracted with Et_2O (3 × 10 mL). The combined organic layers were dried over Na_2SO_4, filtered, and concentrated under reduced pressure. The crude material was purified by flash column chromatography (5% Et_2O-petroleum ether) to give enynal **26** (0.39 g, 82%) as a brown oil.

IR (neat): $\tilde{\nu}_{max}$ 2952 (C-H), 2068 (C≡C), 1677 (C=O), 836

^{1}H NMR (300 MHz, CDCl$_3$): δ 10.19 (s, 1H, H-7), 2.38 (dtd, J 6.0, 3.0, 1.7, 2H), 2.23 (dtd, J 4.2, 2.7, 1.4, 2H), 1.68–1.59 (st, 4H), 0.20 (s, 9H, H-10)

^{13}C NMR (101 MHz, CDCl$_3$): δ 193.1 (CH, C-7), 143.7 (C, C-2), 139.8 (C, C-1), 104.7 (C, C-8), 101.6 (C, C-9), 32.3 (CH$_2$), 22.1 (CH$_2$), 21.9 (CH$_2$), 21.1 (CH$_2$), -0.17 (CH$_3$, C-10)

These data are in agreement with literature reported values. [20]

Enynol 27

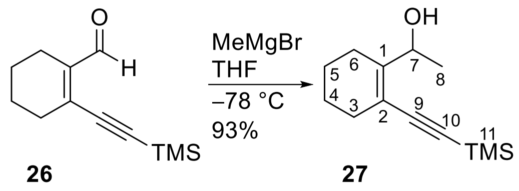

Procedure adapted from a literature report.[21]

Enynal **26** (0.25 g, 1.21 mmol) was dissolved in THF (6 mL), and the resulting mixture was cooled to −78 °C. MeMgBr (0.5 mL, 1.5 mmol, 3 M in THF) was added over 5 min, after which the reaction mixture was allowed to warm to 23 °C by removal of the cooling bath. Upon completion, the reaction was quenched by the addition of $NH_4Cl_{(aq.)}$ (5 mL) and extracted with EtOAc (3 × 10 mL). The combined organic layers were washed with brine (3 × 10 mL) and dried over $MgSO_4$, filtered, and concentrated under reduced pressure. The crude material was purified by flash column chromatography (20% Et_2O-petroleum ether) to give enynal **27** (0.25 g, 93%) as a brown oil.

IR (neat): $\tilde{\nu}_{max}$ 3333 (O-H), 2932 (C-H), 2137 (C≡C), 1250, 836

^{1}H NMR (400 MHz, CDCl$_3$): δ 4.98 (q, J 6.5, 1H, H-7), 2.32–1.98 (st, 2H), 1.86 (s, 1H, OH), 1.69–1.46 (st, 2H), 1.27 (d, J 6.5, 3H, H-8), 0.18 (s, 9H, H-11)

^{13}C NMR (101 MHz, CDCl$_3$): δ 149.6 (C, C-1), 115.4 (C, C-2), 104.7 (C, C-9), 98.0 (C, C-10), 69.6 (CH, C-7), 30.1 (CH$_2$), 23.3 (CH$_2$), 22.4 (CH$_2$), 22.2 (CH$_2$), 20.7 (CH$_3$, C-8), 0.19 (CH$_3$, C-11)

HRMS [TOF-ES$^+$]: calculated for ([M+Na]$^+$, $C_{13}H_{22}ONaSi$): 245.1338, found: 245.1337

Furan 28 *via* enynol S5

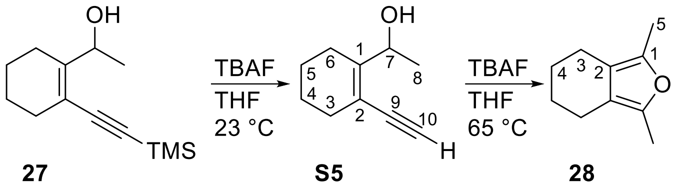

Procedure adapted from a literature report.[22]

TBAF (0.4 mL, 0.4 mmol, 1.0 M in THF) was added to a solution of enynol **27** (60 mg, 0.27 mmol) in THF (0.5 mL), and the resulting mixture was stirred at 23 °C. Upon completion, the reaction was diluted with Et_2O (10 mL) and washed with H_2O (3 × 10 mL). The aqueous layer was re-extracted with Et_2O (3 × 10 mL), then the combined organic layers were dried over $MgSO_4$, filtered, and concentrated under reduced pressure. The crude material was partially purified by flash column chromatography (50% Et_2O -petroleum ether) to give a mixture of enynol **27** and enynol **S5** (35 mg) as a brown oil.

TBAF (0.2 mL, 0.2 mmol, 1.0 M in THF) was added to a mixture of enynols **27** and **S5** (35 mg, 0.23 mmol) in THF (0.5 mL), and the resulting mixture was heated at 65 °C for 15 h. Upon completion, the reaction mixture was diluted with Et_2O (10 mL) and washed with H_2O (3 × 10 mL). The combined organic layers were dried over $MgSO_4$, filtered, and concentrated under reduced pressure to give crude furan **28** (34 mg) as a yellow oil, which was used in the next step without further purification.

Furan 28:

^{1}H NMR (400 MHz, $CDCl_3$): δ 2.44–2.29 (st, 4H, H-4), 2.13 (s, 6H, H-5), 1.64 (ddd, J 6.5, 4.0, 2.5, 4H, H-3)

^{13}C NMR (101 MHz, $CDCl_3$): δ 143.0 (C, C-1), 116.4 (C, C-2), 23.6 (CH_2, C-3), 20.6 (CH_2, C-4), 11.6 (CH_3, C-5)

Silyl enol ether 29

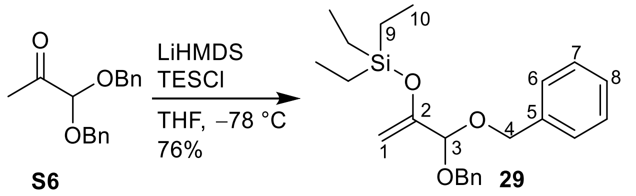

Procedure adapted from a literature report.[23]

Ketone **S6** (377 mg, 1.40 mmol) and TESCl (0.35 mL, 2.09 mmol) were dissolved in THF (7 mL) and the resulting solution was cooled to −78 °C. LiHMDS (1 M in THF, 1.70 mL, 1.70 mmol) was added to over 20 min, resulting in a yellow solution. After 45 min, the reaction mixture was quenched by the addition of $NaHCO_{3\ (aq.)}$ (3 mL), then diluted with H_2O (10 mL) and extracted with Et_2O (3 × 15 mL). The organic layers were washed with brine (3 × 15 mL), then dried over Na_2SO_4, filtered, and concentrated under reduced pressure. The crude material was purified by flash column chromatography (95:4:1 petroleum ether-Et_2O-Et_3N) to give silyl enol ether **29** (406 mg, 76%) as a colourless oil.

TLC: 25% Et_2O-petroleum ether, R_f = 0.32 Vanillin

IR (neat): $\tilde{\nu}_{max}$ 3031 w (C-H), 2955 w (C-H), 2912 w (C-H), 2877 w (C-H), 1640 w (C=C-O), 1455 w (C=C), 1055 m (C-O), 1018 m (C-O), 729 st, 695 st

^{1}H NMR (400 MHz, $CDCl_3$): *δ* 7.38−7.27 (st, 10H, H-8, 9 and 10), 4.93 (s, 1H, H-3), 4.73 (t, *J* 1.0, 1H, H-1'), 4.67 (d, *J* 11.9, 2H, H-6'), 4.59 (d, *J* 11.9, 2H, H-6''), 4.41 (d, *J* 1.2, 1H, H-1''), 0.98 (t, *J* 7.9, 9H, H-5), 0.76−0.68 (m, 6H, H-4)

^{13}C NMR (101 MHz, $CDCl_3$): *δ* 153.9 (C), 138.3 (C), 128.4 (CH, C-8), 127.9 (CH, C-9), 127.6 (CH, C-10), 99.2 (CH, C-3), 92.4 (CH_2, C-1), 67.7 (CH_2, C-6), 6.8 (CH_3, C-5), 5.0 (CH_2, C-4)

LRMS [TOF-ES$^+$]: *m/z* 407 (100%, $[M+Na]^+$)

These data are in agreement with literature reported values.[23]

Oxabicyclic ketones 30 and 31

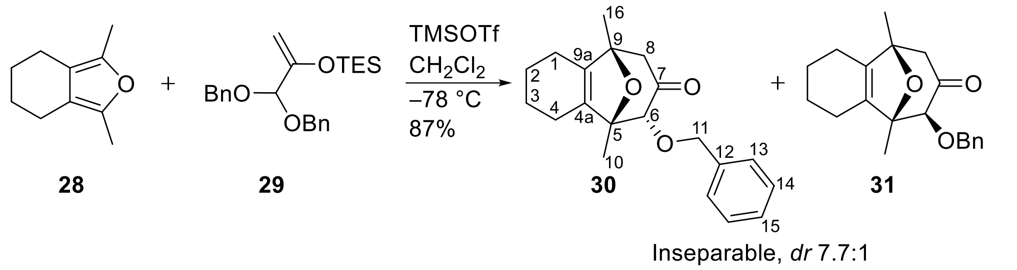

Procedure adapted from a literature report.[24]

A solution of crude furan **28** (66 mg, nominally 0.44 mmol) and silyl enol ether **29** (170 mg, 0.67 mmol) in CH_2Cl_2 (5 mL) was stirred at −78 °C for 15 min. At this time, TMSOTf (2 drops) was added and the resulting solution was stirred for a further 30 min, at which time the reaction was quenched by the addition of $NaHCO_{3\,(aq.)}$ (1 mL). The reaction mixture was allowed to warm to 23 °C and was then extracted with CH_2Cl_2 (3 × 10 mL). The combined organic layers were dried over Na_2SO_4, filtered, and concentrated under reduced pressure. The crude material was purified by flash column chromatography (25% Et_2O -petroleum ether) to give oxabicycles **30** and **31** (120 mg, 87%, *dr* 7.7:1) as a colourless oil.

IR (neat): $\tilde{\nu}_{max}$ 2931 (C-H), 1720 (C=O), 1103

^{1}H NMR (400 MHz, $CDCl_3$): δ **7.38–7.26** (st, 5H, Ar), **5.03**/4.73 (d, *J* 11.9/12.2, 1H, H-11’), **4.52**/4.43 (d, *J* 11.9/12.2, 1H, H-11’’), **3.80**/3.14 (s, 1H, H-6), 2.75/**2.55** (d, *J* 15.3/14.8, 1H, H-8’), **2.42**/2.24 (d, *J* 14.8/15.3, 1H, H-8’’), 2.17–1.94 (st, 3H), 1.85–1.72 (m, 1H), 1.69–1.49 (st, 5H), **1.40**/1.38 (s, 3H, H-10), **1.35**/1.34 (s, 3H, H-16)

**Where possible, sister signals are paired together with the frequency in bold denoting the major compound, this may alter the order of peaks.*

^{13}C NMR (101 MHz, $CDCl_3$): δ **206.8**/205.4 (C, C-7), 143.5/**139.9** (C, C-9a), **139.3**/137.1 (C, C-4a), **138.2**/137.5 (C, C-12), 128.6/128.43/**128.39**/**128.0**** (CH, C-13 or 14), **127.8**† (CH, C-15), **87.5**/80.2 (CH, C-6), **86.0**/85.5 (C, C-5), **84.4**/83.7 (C, C-9), 74.3/**72.5** (CH_2, C-11), **51.2**/47.2 (CH_2, C-8), **22.22**** (CH_2), **22.16**** (CH_2), **22.1** (CH_2), **21.53**/21.45** (CH_3, C-16), 21.20** (CH_2), 21.15** (CH_2), **21.0** (CH_2), **19.2**/17.2 (CH_3, C-10)

**Where possible, sister signals are paired together with the frequency in bold denoting the major compound, this may alter the order of peaks.*

***2 d.p. provided to distinguish peaks that would otherwise be the same at 1 d.p.*

†There are three missing signals for the minor isomer.

HRMS [TOF-ES$^+$]: calculated for ([M+Na]$^+$, $C_{20}H_{24}NaO_3$): 335.1632, found: 335.1631

Chloroenal 33

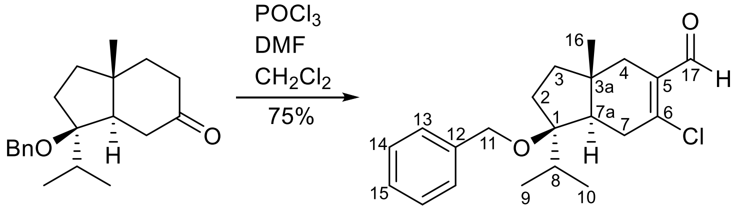

24 33

Procedure adapted from a literature report.[25]

DMF (0.12 mL, 1.5 mmol) and CH_2Cl_2 (1.2 mL) were cooled to 0 °C and $POCl_3$ (0.06 mL, 0.65 mmol) was added over 5 min. The cooling bath was removed upon complete addition, and the solution stirred for 20 min at 23 °C. At this time, ketone **24** (150 mg, 0.50 mmol) in CH_2Cl_2 (0.8 mL) was added over 5 min, causing a yellowing of the solution. After 24 h the reaction was quenched by addition of ice and solid $NaHCO_3$ until the effervescence ceased. The reaction mixture was partitioned with Et_2O (30 mL) and H_2O (20 mL). The aqueous phase was extracted with Et_2O (2 × 15 mL), and the organic layers were washed with brine (3 × 10 mL), then dried over $MgSO_4$, filtered, and concentrated under reduced pressure to a yellow oil. The crude material was purified by flash column chromatography (10% Et_2O-petroleum ether) to give chloroenal **33** (130 mg, 75%) as a pale-yellow oil.

Notes: Removal of solvent at room temperature. This compound was stored in a freezer under argon until needed.

TLC: 25% Et_2O-petroleum ether, R_f = 0.60 UV / Vanillin

IR (neat): $\tilde{\nu}_{max}$ 2949 w (C-H), 2889 w (C-H), 2861 w (C-H), 1671 st (C=O), 1608 sh m (C=C) 1062 m (C-O), 697 m

^{1}H NMR (400 MHz, CDCl$_3$): δ 10.23 (s, 1H, H-17), 7.38−7.25 (st, 5H, H-13, 14 and 15), 4.46−4.39 (st, 2H, H-11), 3.04 (dddd, J 19.6, 12.2, 4.2, 2.0, 1H, H-7’), 2.55 (ddd, J 19.6, 5.2, 2.4, 1H, H-7’’), 2.51 (dd, J 17.2, 2.0, 1H, H-4'), 2.32 (sept, J 6.8, 1H, H-8), 2.26−2.17 (m, 1H), 1.97 (d, J 16.9, 1H, H-4’’), 1.91 (dd, J 13.8, 7.9, 1H), 1.76 (*ap.* ddd, J 12.1, 6.3, 3.0, 2H, H-7a and 1H of CH_2), 1.31−1.22 (m, 1H), 0.95 (*ap.* t, J 7.4, 9H, H-9, 10 and 16)

^{13}C NMR (101 MHz, CDCl$_3$): *δ* 192.0 (CH, C-17), 153.1 (C, C-6), 139.7 (C, C-12), 132.7 (C, C-5), 128.4 (CH, C-14), 127.2 (CH, C-15), 127.1 (CH, C-13), 87.3 (C, C-1), 62.9 (CH$_2$, C-11), 46.9 (CH, C-3a), 40.0 (C, C-7a), 39.7 (CH$_2$, C-2 or 3), 38.9 (CH$_2$, C-4), 36.7 (CH$_2$, C-7), 34.8 (CH$_2$, C-2 or 3), 33.1 (CH, C-8), 18.6 (CH$_3$, C-16), 18.1 (CH$_3$, C-9 or 10), 17.7 (CH$_3$, C-9 or 10)

LRMS [TOF-ES+]: *m/z* 552 (5%), 536 (5), 426 (5), 406 (25), 404 (100), 371 (15, [M{^{37}Cl}+Na]$^+$), 369 (50, [M{^{35}Cl}+Na]$^+$), 274 (55), 251 (85), 206 (95)

Keto alcohol 35

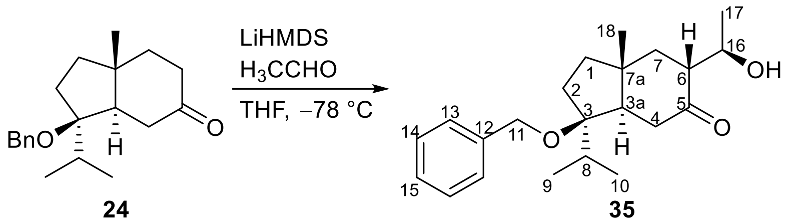

This procedure was adapted from reported literature.[10][26]

A solution of ketone **24** (401 mg, 1.33 mmol) in THF (4 mL) was cooled to −78 °C and LiHMDS (1 M in THF, 1.50 mL, 1.50 mmol) was added over 2 min. After 1 h, acetaldehyde (0.22 mL, 4.0 mmol) in THF (1.3 mL) was added in two portions. After a further 85 min, the reaction mixture was allowed to warm via removal from the cooling bath, and $NH_4Cl_{(aq.)}$ (5 mL) was added. The mixture was diluted in Et_2O (10 mL) and washed with H_2O (1 × 10 mL). The aqueous phase was re-extracted with Et_2O (3 × 10 mL), and the combined organics were washed with brine (3 × 10 mL), dried over $MgSO_4$, filtered, and concentrated under reduced pressure. The crude material was taken into the next step without further purification.

An analytically pure sample was obtained by flash column chromatography (40% Et_2O-petroleum ether) to give aldol product **35** as a pale-yellow oil.

Notes: In this instance, only a single isomer at C_{16} was detected. Later chemistry and the literature suggest there is an ca. 10:1 ratio here.

TLC: 50% Et_2O-petroleum ether, R_f = 0.26 Vanillin

IR (neat): $\tilde{\nu}_{max}$ 3459 br (OH), 2963 w (C-H), 2938 w (C-H), 2879 w (C-H), 1689 m (C=O), 1455 w (C=C), 1059 m (CO), 732 m, 697 m

^{1}H NMR (400 MHz, CDCl$_3$): *δ* 7.36−7.30 (st, 4H, H-13 and 14), 7.28−7.23 (m, 1H, H-15), 4.42 (s, 2H, H-11), 4.13 (s, 1H, OH), 3.96 (*ap.* p, *J* 6.4, 1H, H-16), 2.77 (*ap.* td, *J* 14.8, 1.0, 1H, H-4'), 2.48

(dd, *J* 15.2, 3.5, 1H, H-4''), 2.40–2.20 (st, 3H, H-2', 6 and 8), 1.93 (t, *J* 6.4, 1H, H-7'), 1.92–1.85 (m, 1H, H-2''), 1.76–1.68 (st, 2H, H-1' and 3a), 1.26 (s, 3H, H-18), 1.26–1.17 (st, 2H, H-1'' and 7''), 1.15 (d, *J* 6.4, 3H, H-17), 0.93 (*ap.* dd, *J* 6.8, 4.3, 6H, H-9 and 10)*

**Note: this is actually a pair of doublets J 6.8 but they overlap. As such the 4.3 Hz coupling in artificial.*

^{13}C NMR (400 MHz, $CDCl_3$): *δ* 217.5 (C, C-5), 139.7 (C, C-12), 128.4 (CH, C-13 or 14), 127.1 (CH, C-15), 126.9 (CH, C-13 or 14), 87.4 (C, C-3), 68.7 (CH, C-16), 62.6 (CH_2, C-11), 53.0 (CH, C-6), 50.4 (CH, C-3a), 42.3 (CH_2, C-4), 41.9 (C, C-7a), 41.4 (CH_2, C-7), 39.9 (CH_2, C-1), 34.9 (CH_2, C-2), 33.0 (CH, C-8), 20.2 (CH_3, C-17), 19.1 (CH_3, C-18), 18.3 (CH_3, C-9 or 10), 17.8 (CH_3, C-9 or 10)

LRMS [TOF-ES$^+$]: *m/z* 408 (100%, $[M+MeCN+Na]^+$), 367 (50, $[M+Na]^+$), 271 (50), 237 (30, $[M-OBn]^+$), 219 (30, $[M-OBn-H_2O]^+$)

HRMS [TOF-ES$^+$]: calculated for ($[M+Na]^+$, $C_{22}H_{32}O_3Na$): 367.2249, found: 367.2251

Although this compound has been reported in the literature, no data is reported for the free alcohol, only for the TBS ether. [10]

Alkyne 1,3-diols 36–38

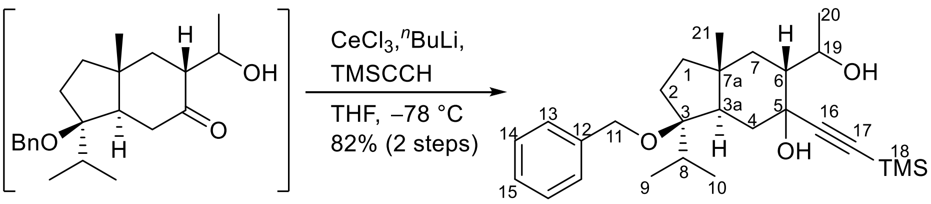

35 **36-38**

Procedure adapted from a literature report.[26]

A Schlenk tube containing $CeCl_3·7H_2O$ (1.49 g, 3.99 mmol) was heated at 140 °C for 2 h under vacuum (<1 mbar) whilst stirring. A sintered adapter was used to prevent powdered $CeCl_3$ from being pulled through the tubing. Upon cooling, the tube was backfilled with argon, and THF (2.5 mL) was added to form a white slurry. This was left to stir under argon overnight in which time the slurry thickens. A solution of TMS-acetylene (0.55 mL, 3.99 mmol) in THF (2.4 mL) was cooled to −78 °C and ^{n}BuLi (2.0 M in hexanes, 2.0 mL, 4.0 mmol) was added over 2 min. After 20 min, the Ce slurry was cooled to −78 °C, and the Li-acetylide solution was added over 5 min, resulting in a yellow slurry. After 1 h, a solution of the crude aldol product **35** (assumed 1.33 mmol) in THF (4 mL) was added over 5 min. After 1 h, the mixture was warmed to room temperature via removal of the cooling bath and diluted with Et_2O (10 mL) and

$NH_4Cl_{(aq.)}$ (3 mL). The organic layer was washed with H_2O (3 × 10 mL), the aqueous layers re-extracted with Et_2O (3 × 10 mL). Then the combined organic layers were washed with brine (2 × 10 mL), dried over $MgSO_4$, filtered, and the solvent removed under reduced pressure. The crude material was purified by flash column chromatography (25% → 50% Et_2O-petroleum ether) to give first, diol **38** (40 mg, 7%) as clear white crystals, then diol **37** (45 mg, 8%) as a colourless oil that forms plate crystals on standing, and finally diol **36** (252 mg, 43%) as a white crystalline solid. Furthermore, diols **36** and **37** co-eluted to give a white solid (146 mg, 25%).

38:

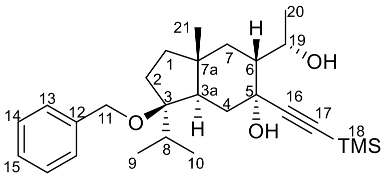

TLC: 50% Et_2O-petroleum ether, R_f = 0.52 Vanillin

IR (neat, ATR attachment): $\tilde{\nu}_{max}$ 3157 br (OH), 2958 n (C-H), 2903 w (C-H), 2874 w (C-H), 2169 n (C≡C), 1709 w, 1608 w (C=C), 840 st, 730 m

^{1}H NMR (400 MHz, $CDCl_3$): *δ* 7.37−7.31 (st, 4H, H-13 and 14), 7.29−7.20 (m, 1H, H-15), 4.84−4.74 (m, 1H, H-19), 4.42 (s, 2H, H-11), 3.38 (s, 1H, 3°-OH), 2.47 (d, *J* 3.4, 1H, 2°-OH), 2.28 (sept, *J* 6.7, 1H, H-8), 2.15 (ddd, *J* 14.0, 11.7, 7.9, 1H), 2.05−1.99 (st, 2H), 1.95 (dd, *J* 10.9, 4.4, 1H, H-3a), 1.81 (dd, *J* 13.9, 8.4, 1H), 1.68 (ddd, *J* 11.8, 5.4, 1.3, 1H), 1.62 (dd, *J* 11.7, 7.8, 1H), 1.58−1.51 (st, 2H), 1.28−1.20 (m, 1H), 1.20 (d, *J* 6.5, 3H, H-20), 1.06 (s, 3H, H-21), 0.99 (d, *J* 6.9, 3H, H-9 or 10), 0.97 (d, *J* 6.7, 3H, H-9 or 10), 0.17 (s, 9H, H-18)

^{13}C NMR (101 MHz, $CDCl_3$): *δ* 140.3 (C, C-12), 128.3 (CH, C-13 or 14), 127.1 (CH, C-13 or 14), 127.0 (CH, C-15), 110.5 (C, C-16), 95.0* (C, C-17), 88.3 (C, C-3), 73.0 (C, C-5), 68.8 (CH, C-19), 62.9 (CH_2, C-11), 46.1 (CH, C-6), 44.2 (CH, C-3a), 41.7 (C, C-7a), 40.8 (CH_2, C-1 or 7), 38.8 (CH_2, C-4), 33.9 (CH_2, C-2), 33.2 (CH_2, C-1 or 7), 33.1 (CH, C-8), 21.7 (CH_3, C-20), 19.1 (CH_3, C-21), 18.5 (CH_3, C-9 or 10), 18.3 (CH_3, C-9 or 10), 0.2 (CH_3, C-18)

**Appeared on a JMOD experiment, but not on the ^{13}C UDEFT and as such assignment is tentative, and only assigned by comparison with the other isomers.*

HRMS [TOF-ES$^+$]: calculated for ($[M+Na]^+$, $C_{27}H_{42}O_3SiNa$): 465.2801, found: 465.2800

37:

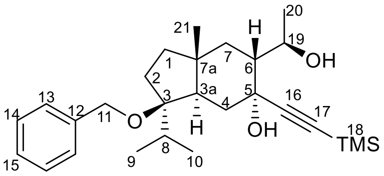

TLC: 50% Et_2O-petroleum ether, R_f = 0.49 Vanillin

MP: 125−128 °C (Et_2O-petroleum ether)

IR (neat, ATR attachment): $\tilde{\nu}_{max}$ 3347 br (OH), 2959 w (C-H), 2160 w (C≡C), 1455 w (C=C), 1249 n, 1056 m (C-O), 993 m, 840 st, 729 st

^{1}H NMR (400 MHz, $CDCl_3$): δ 7.38−7.31 (st, 4H, H-13 and 14), 7.28−7.23 (m, 1H, H-15), 4.41 (s, 2H, H-11), 4.00 (*ap.* p, *J* 6.4, 1H, H-11), 2.92 (br s, 1H, OH), 2.71 (br s, 1H, OH), 2.28 (sept, *J* 6.8, 1H, H-8), 2.20−2.00 (st, 3H, H-2' and 4), 1.93−1.76 (st, 3H, H-3a, 6 and H-2''), 1.59 (dd, *J* 11.7, 7.8, 1H, H-1'), 1.49 (dd, *J* 12.6, 4.5, 1H, H-7'), 1.39 (*ap.* d, *J* 12.7, 1H, H-7''), 1.33 (d, *J* 6.5, 3H, H-20), 1.20−1.11 (m, 1H, H-1''), 1.07 (s, 3H, H-21), 1.00 (d, *J* 6.9, 3H, H-9 or 10), 0.97 (d, *J* 6.7, 3H, H-9 or 10), 0.17 (s, 9H, H-18)

^{13}C NMR (101 MHz, $CDCl_3$): δ 140.2 (C, C-12), 128.3 (CH, C-13 or 14), 127.1 (CH, C-13 or 14), 127.0 (CH, C-15), 112.1 (C, C-16), 89.7 (C, C-17), 88.3 (C, C-3), 71.3 (CH, C-19), 69.2 (C, C-5), 62.9 (CH_2, C-11), 48.3 (CH, C-6), 44.0 (CH, C-3a), 42.0 (C, C-7a), 40.6 (CH_2, C-1), 40.5 (CH_2, C-7), 39.5 (CH_2, C-4), 34.0 (CH_2, C-2), 33.1 (CH, C-8), 22.8 (CH_3, C-20), 19.0 (CH_3, C-21), 18.5 (CH_3, C-9 or 10), 18.3 (CH_3, C-9 or 10), 0.0 (CH_3, C-18)

HRMS [TOF-ES$^+$]: calculated for ($[M+Na]^+$, $C_{27}H_{42}O_3SiNa$): 465.2801, found: 465.2802

36:

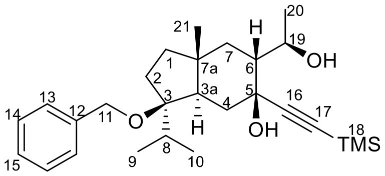

TLC: 50% Et_2O-petroleum ether, R_f = 0.37 Vanillin

MP: 100−103 °C (acetone)

IR (neat, ATR attachment): $\tilde{\nu}_{max}$ 3382 br (OH), 3285 br (OH), 2963 w (C-H), 2942 w (C-H), 2851 w (C-H), 2160 n (C≡C), 1453 n (C=C), 1250 m, 1090 m (CO), 842 st, 729 st

^{1}H NMR (400 MHz, CDCl$_3$): δ 7.35−7.27 (st, 4H, H-13 and 14), 7.25−7.19 (m, 1H, H-15), 4.87 (s, 1H, 3°-OH), 4.44 (d, *J* 12.0, 1H, 11′), 4.38 (d, *J* 12.0, 1H, H-11′′), 4.13 (dqd, *J* 9.3, 6.2, 3.6, 1H, H-19), 2.58 (d, *J* 3.6, 1H, 2°-OH), 2.28 (sept, *J* 6.8, 1H, H-8), 2.19−2.09 (st, 2H, H-2′ and 4′), 1.98 (*ap.* t, *J* 12.8, 1H, H-4′′), 1.86−1.75 (st, 3H, H-2′′, 3a, and 6), 1.59 (dd, *J* 11.5, 7.9, 1H, H-7′), 1.53 (dd, *J* 12.7, 4.1, 1H, H-1′), 1.23 (d, *J* 6.2, 3H, H-20), 1.15−1.09 (m, 1H, H-7′′), 1.08 (s, 3H, H-21), 1.00 (d, *J* 6.8, 3H, H-9 or 10), 0.98 (d, *J* 6.8, 3H, H-9 or 10), 0.94 (m, 1H, H-1′′), 0.19 (s, 9H, H-18)

^{13}C NMR (101 MHz, CDCl$_3$): δ 140.2 (C, C-12), 128.2 (CH, C-13 or 14), 126.8 (CH, C-15), 126.7 (CH, C-13 or 14), 108.2 (C, C-16), 90.1 (C, C-17), 87.6 (C, C-3), 75.2 (C, C-5), 72.6 (CH, C-19), 62.5 (CH$_2$, C-11), 49.5 (CH, C-6), 47.9 (CH, C-3a), 42.1 (C, C-7a), 40.8 (CH$_2$, C-1), 40.6 (CH$_2$, C-7), 38.1 (CH$_2$, C-4), 34.3 (CH$_2$, C-2), 32.9 (CH, C-8), 22.7 (CH$_3$, C-20), 19.8 (CH$_3$, C-21), 18.3 (CH$_3$, C-9 or 10), 18.0 (CH$_3$, C-9 or 10), 0.3 (CH$_3$, C-18)

HRMS [TOF-ES$^+$]: calculated for ([M+Na]$^+$, $C_{27}H_{42}O_3SiNa$): 465.2801, found: 465.2804

Single crystals of all three were obtained by slow evaporation of Et_2O. Crystal structures for **36**, **37**, and **38** can be found in the SI.

TIPS ketone 40

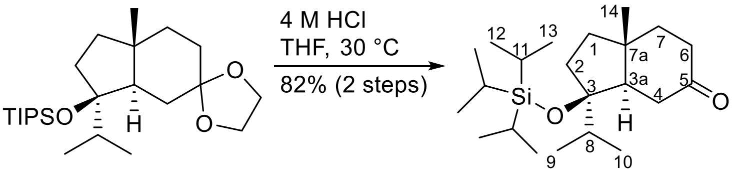

S7 **40**

Procedure adapted from a literature report. [9]

$HCl_{(aq.)}$ (4 M, 6.6 mL) was added to a solution of acetal **S7** (assumed 1.97 mmol) in wet THF (13 mL), in air, and the resulting solution was heated at 30 °C. After 3 h, the reaction mixture was extracted with petroleum-ether (5 × 20 mL). The combined organic layers were dried over $MgSO_4$, filtered, and the solvent removed under reduced pressure. The crude material was purified by flash column chromatography (5 → 10% Et_2O-petroleum ether) to give ketone **40** (590 mg, 82% over two steps) as a colourless oil that solidifies white over time.

TLC: 25% Et_2O-petroleum ether, R_f = 0.46 Vanillin

MP: 39−41 °C (acetone)

Optical Rotation: $[\alpha]_D^{25}$ = −3.6°, (c = 0.5, CH_2Cl_2)

IR (neat, ATR attachment): $\tilde{\nu}_{max}$ 2945 n (C-H), 2866 n (C-H), 1704 st (C=O), 1463 n, 1067 m (C-O), 882 m, 675 st, 623 m

^{1}H NMR (400 MHz, $CDCl_3$): δ 2.63 (dd, *J* 15.8, 14.4, 1H, H-4'), 2.52−2.30 (st, 3H, H-4'' and H-6), 2.22−2.07 (st, 2H, H-2), 1.93 (sept, *J* 6.8, 1H, H-8), 1.84 (ddd, *J* 12.8, 7.2, 1.8, 1H, H-7'), 1.71−1.61 (st, 2H, H-1' and 3a), 1.54 (*ap.* td, *J* 12.4, 6.3, 1H, H-7''), 1.21 (s, 3H, H-14), 1.09 (st, 22H, H-11, 12, 13 and 1''), 0.91* (dd, *J* 6.8, 1.6, 6H)

**This is a pair of doublets almost completely stacked (J 6.8) and as such the 1.6 Hz coupling is unreal.*

^{13}C NMR (101 MHz, $CDCl_3$): δ 213.8 (C, C-5), 86.1 (C, C-3), 50.0 (CH, C-3a), 42.3 (CH_2, C-4), 42.0 (CH_2, C-2), 41.3 (C, C-7a), 40.2 (CH_2, C-1), 39.8 (CH, C-8), 37.7 (CH_2, C-6), 37.3 (CH_2, C-7), 18.84** (CH_3, C-12 or 13), 18.79** (CH_3, C-12 or 13), 18.5 (CH_3, C-9 or 10), 18.3 (CH_3, C-14), 14.4 (CH, C-11)

***2 d.p. provided to distinguish peaks that would otherwise be the same at 1 d.p.*

Note: only one peak was observed for C-9 and 10 but 2D shows both ^{i}Pr correlate with this signal.

HRMS [TOF-ES$^+$]: calculated for ($[M+H]^+$, $C_{22}H_{42}O_2Si$): 367.3027, found: 367.3035

Bromoenal 41

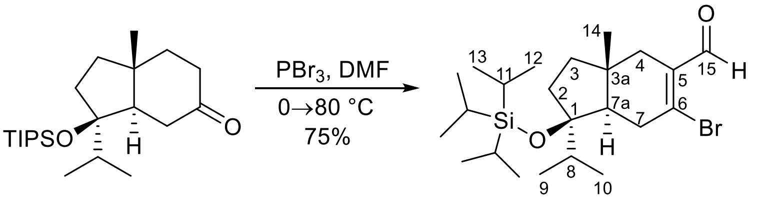

40 **41**

Procedure adapted from a literature report.[27]

PBr_3 (3.10 mL, 33.0 mmol) was added [**Carefully!**] to an ice-cooled solution of DMF (70 mL) over 10 min. After addition, the cooling bath was removed and the reaction mixture was stirred at 23 °C for 30 min, over which time a white slurry formed. At this time, a solution of ketone **40** (4.00 g, 10.9 mmol) in DMF (18 mL) was added and the resulting solution was heated at 80 °C for 35 min, at which time the vessel was cooled to 23 °C and $NaHCO_{3\ (aq.)}$ was added until the pH of the solution was 7, followed by H_2O (100 mL). The aqueous phase was extracted with CH_2Cl_2 (3 × 100 mL) and the combined organic extracts were washed with H_2O

(3 × 100 mL). Following a final re-extraction with CH_2Cl_2 (50 mL), the organic layers were dried over Na_2SO_4, filtered, and concentrated under reduced pressure. The crude material was purified by flash column chromatography (2.5% Et_2O-petroleum ether) to give bromoenal **41** (3.72 g, 75%) as a white solid.

Note: solvent removal was done at ambient temperature (ca. 23 °C).

TLC: 10% Et_2O-petroleum ether, R_f = 0.53 UV / Vanillin

Optical Rotation: $[\alpha]_D^{25}$ = −81.6°, (c = 1.0, CH_2Cl_2)

IR (neat, ATR attachment): $\tilde{\nu}_{max}$ 2925 n (C-H), 2866 n (C-H), 1678 st (C=O), 1602 m (C=C), 1463 m, 1077 m (C-O), 882 m, 670 st

^{1}H NMR (400 MHz, $CDCl_3$): δ 10.06 (s, 1H, H-15), 3.02 (dddd, *J* 19.7, 12.1, 4.2, 2.0, 1H), 2.69 (ddd, *J* 19.7, 4.9, 2.2, 1H), 2.49 (dd, *J* 16.8, 1.9, 1H), 2.14 (dd, *J* 13.7, 7.8, 1H), 2.06−1.87 (m, 3H, H-8 and 2 × 1H of CH_2), 1.68 (dd, *J* 12.1, 7.3, 1H, 1H of CH_2), 1.62 (dd, *J* 12.1, 4.9, 1H, H-7a), 1.19−1.07 (st, 22H, H-11, 12, 13, and 1H of CH_2), 0.96−0.91 (st, 6H, H-14 and either H-16 or 18), 0.88 (d, *J* 6.8, 3H, H-16 or 18)

^{13}C NMR (101 MHz, $CDCl_3$): δ 194.4 (CH, C-15), 145.1 (C, C-6), 134.6 (C, C-5), 85.9 (C, C-1), 48.4 (CH, C-7a), 41.4 (CH_2), 40.5 (CH_2), 40.4 (CH_2), 39.8 (CH, C-8), 39.7 (CH_2), 39.5 (C, C-3a), 18.84** (CH_3, C-12 or 13), 18.79†** (CH_3, C-12 or 13), 18.6 (CH_3, C-9, 10 or 14), 18.5 (CH_3, C-9, 10 or 14), 14.3 (CH, C-11)

***2 d.p. provided to distinguish peaks that would otherwise be the same at 1 d.p.*

†This is peak is slightly taller than the 18.84 ppm peak, and there is a ^{13}C environment missing. It has been seen in other compounds that this peak often merges into the TIPS-signals and it is likely that this is occurring here.

HRMS [TOF-ES$^+$]: calculated for ([M{^{79}Br}−OTIPS]$^+$, $C_{14}H_{20}O^{79}Br$): 283.0692, found: 283.0691

Enynol 42

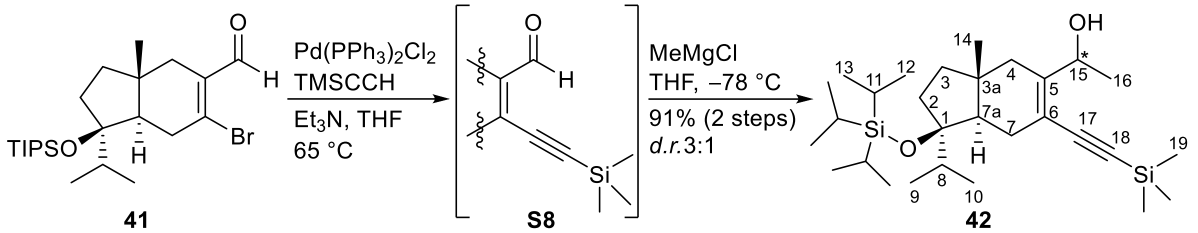

41 **S8** **42**

Procedure adapted from a literature report.[21]

Bromide **41** (3.72 g, 8.14 mmol) was dissolved in THF (24 mL) with Et_3N (3.40 mL, 24.3 mmol) and $Pd(PPh_3)_2Cl_2$ (284 mg, 0.41 mmol). The resulting solution was degassed for 25 min, then TMSCCH (1.20 mL, 8.91 mmol) was added and the resulting mixture was heated at 65 °C. Upon completion (^{1}H NMR*), the solution was allowed to cool to 23 °C and the solvent removed under reduced pressure. The residue was taken up in hexane (30 mL) and filtered through celite to remove all solids, washing with portions of hexane. The filtrate was poured into a separating funnel and washed with MeCN (50 + 25 mL). The MeCN-phase was re-extracted with hexane (3 × 40 mL) using TLC to monitor extraction, and the combined hexane layers were washed with H_2O (3 × 40 mL), then dried over $MgSO_4$, filtered, and the solvent removed under reduced pressure. The crude material was used in the next step without further purification.

TLC: 10% Et_2O-petroleum ether, R_f = 0.53 UV / Vanillin*

**Starting material and product have the same R_f and dip sensitivity-as such this reaction was monitored by ^{1}H NMR of reaction aliquots taken from solution and concentrated under reduced pressure, before being taken up in the NMR solvent: CHO_{sm} 10.06 ppm, CHO_{pr} 10.26 ppm ($CDCl_3$).*

Crude enynal **S8** (assumed 8.14 mmol) was dissolved in THF (18 mL) and the resulting solution cooled to −78 °C. After 10 min at this temperature, MeMgCl (5.60 mL, 12.2 mmol, 2.17 M solution in THF) was added. Upon completion, the reaction mixture was allowed to warm to 23 °C, then quenched with H_2O/NH_4Cl and extracted with Et_2O (3 × 40 mL). The combined organic layers were washed with brine (3 × 30 mL), dried over $MgSO_4$, filtered, and concentrated under reduced pressure. The crude material was purified by column chromatography (10 → 20% Et_2O-petroleum ether) to give an isomeric mix of enynols **42a** and **42b** (3.63 g, 91% over two steps, crude *d.r.* 1:3) as an orange solid.

The isomers can be partially separated under these conditions, and as such the data is given individually.

42a:

TLC: 25% Et_2O-petroleum ether, R_f = 0.42 UV / Vanillin

Optical Rotation: $[\alpha]_D^{25}$ = −46°, (c = 0.5, CH_2Cl_2)

IR (thin film, CH_2Cl_2): $\tilde{\nu}_{max}$ 3388 br (OH), 2944 n (C-H), 2867 n (C-H), 2138 w (C≡C), 1464 w, 1249 m, 1063 m (C-O), 840 m

^{1}H NMR (400 MHz, $CDCl_3$): *δ* 4.94 (qd, *J* 6.5, 4.5, 1H, H-15), 2.42 (dddd, *J* 16.8, 12.2, 4.3, 2.0, 1H), 2.25 (dd, *J* 17.4, 1.9, 1H), 2.16−2.05 (st, 2H), 2.02−1.86 (st, 4H, H-8, OH, and 2H of CH_2), 1.64 (dd, *J* 11.9, 7.3, 1H), 1.36 (dd, *J* 12.2, 4.9, 1H, H-7a), 1.27 (d, *J* 6.5, 3H, H-15), 1.14−1.05 (st, 22H, H-11, 12, 13, and 1H of CH_2), 0.95 (d, *J* 6.8, 3H, H-9 or 10), 0.93 (s, 3H, H-14), 0.87 (d, *J* 6.8, 3H, H-9 or 10), 0.20 (s, 9H, H-19)

^{13}C NMR (101 MHz, $CDCl_3$): *δ* 148.7 (C, C-5), 116.9 (C, C-6), 105.2 (C, C-17), 98.1 (C, C-18), 86.2 (C, C-1), 69.3 (CH, C-15), 46.5 (CH, C-7a), 41.3 (CH_2), 40.4 (CH_2), 40.04** (CH, C-8), 40.01** (CH_2), 39.8 (C, C-3a), 31.2 (CH_2), 20.2 (CH_3, C-16), 18.92** (CH_3, C-14), 18.87** (CH_3, C-12 or 13), 18.8 (CH_3, C-12 or 13), 18.7 (CH_3, C-9 or 10), 18.5 (CH_3, C-9 or 10), 14.4 (CH, C-11), 0.20 (CH_3, C-19)

***2 d.p. provided to distinguish peaks that would otherwise be the same at 1 d.p.*

HRMS [TOF-ES$^+$]: calculated for ($[M+H]^+$, $C_{29}H_{55}O_2Si_2$): 491.3735, found: 491.3725

42b:

TLC: 25% Et_2O-petroleum ether, R_f = 0.34 UV/Vanillin

Optical Rotation: $[\alpha]_D^{25}$ = −42°, (c = 0.5, CH_2Cl_2)

IR (thin film, CH_2Cl_2): $\tilde{\nu}_{max}$ 3326 br (OH), 2943 w (C-H), 2867 n (C-H), 2136 w (C≡C), 1464 w, 1249 n, 1060 m (C-O), 855 m, 840 sh

^{1}H NMR (400 MHz, $CDCl_3$): *δ* 5.10 (dd, *J* 6.7, 3.0, 1H, H-15), 2.37 (tdd, *J* 14.0, 5.2, 2.7, 1H, H-7'), 2.18−1.88 (st, 6H, H-4', 4'', 7'', 8, and either H-2 or 3), 1.68−1.58 (st, 2H, OH and H-2' or 3'), 1.40 (dd, *J* 12.1, 4.9, 1H, H-7a), 1.25 (d, *J* 6.5, 3H, H-16), 1.14−1.05 (st, 22H, H-2'' or 3'', and H-11, 12, 13), 0.94 (d, *J* 6.8, 3H, H-9 or 10), 0.90 (s, 3H, H-14), 0.86 (d, *J* 6.8, 3H, H-9 or 10), 0.20 (s, 9H, H-19)

^{13}C NMR (101 MHz, CDCl$_3$): δ 149.1 (C, C-5), 115.9 (C, C-6), 105.0 (C, C-17), 98.2 (C, C-18), 86.3 (C, C-1), 69.9 (CH, C-15), 46.5 (CH, C-7a), 41.2 (CH$_2$), 40.4 (CH$_2$), 40.1 (CH, C-8), 39.64** (CH$_2$), 39.59** (C, C-3a), 30.9 (CH$_2$, C-7), 21.1 (CH$_3$, C-16), 18.87** (CH$_3$, C-12 or 13), 18.85** (CH$_3$, C-14), 18.8 (CH$_3$, C-12 or 13), 18.7 (CH$_3$, C-9 or 10), 18.5 (CH$_3$, C-9 or 10), 14.4 (CH, C-11), 0.23 (CH$_3$, C-19)

***2 d.p. provided to distinguish peaks that would otherwise be the same at 1 d.p.*

HRMS [TOF-ES$^+$]: calculated for ([M+H]$^+$, $C_{29}H_{55}O_2Si_2$): 491.3735, found: 491.3739

Furan 43

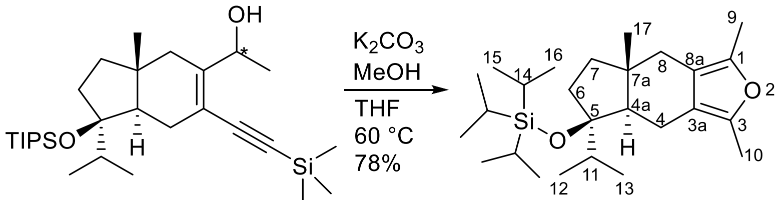

Enynol **42** (1.63 g, 3.31 mmol) and K_2CO_3 (4.58 g, 33.1 mmol) were dissolved in THF (5 mL) and MeOH (11 mL). The resulting solution was heated at 60 °C for 3.5 h. Upon completion, the reaction mixture was allowed to cool to 23 °C and was then filtered, partitioned with Et_2O (30 mL), H_2O (30 mL) and $NH_4Cl_{(aq.)}$ (5 mL). The aqueous layer was further extracted with Et_2O (3 × 20 mL) and the combined organic layers were washed with brine (3 × 30 mL), dried over Na_2SO_4, filtered, and concentrated under reduced pressure. The crude material was purified by column chromatography (3% Et_2O-petroleum ether) to give furan **43** (1.09 g, 78%) as a yellow oil.

TLC: 10% Et_2O-petroleum ether, R_f = 0.78 Vanillin

Optical Rotation: $[\alpha]_D^{25}$ = −23°, (c = 0.6, CH_2Cl_2)

IR (neat): $\tilde{\nu}_{max}$ 2939 n (C-H), 2867 n (C-H), 1603 w (C≡C), 1463 w, 1064 m (C-O), 881 m, 670 m

^{1}H NMR (400 MHz, CD$_2$Cl$_2$): δ 2.49 (dddd, *J* 14.3, 12.8, 2.9, 1.5, 1H, H-4'), 2.39 (d, *J* 14.2, 1H, H-8'), 2.35 (dd, *J* 15.6, 4.9, 1H, H-4''), 2.19−2.03 (st, 3H, H-8'' and either H-6' or 7'), 2.11 (s, 3H, H-9 or 10), 2.09 (s, 3H, H-9 or 10), 1.97 (*ap.* p, *J* 6.8, 1H, H-11), 1.64 (dd, *J* 12.0, 6.8, 1H, H-6' or 7'), 1.60 (dd, *J* 12.8, 5.0, 1H, H-4a), 1.21 (dd, *J* 12.2, 4.4, 1H, H-6'' or 7''), 1.14−1.10 (st, 21H, H-14, 15 and 16), 0.97 (d, *J* 6.8, 3H, H-12 or 13), 0.96 (s, 3H, H-17), 0.91 (d, *J* 6.8, 3H, H-12 or 13)

^{13}C NMR (101 MHz, CD_2Cl_2): δ 143.4 (C, C-1), 143.1 (C, C-3), 118.0 (C, C-3a), 117.6 (C, C-8a), 86.1 (C, C-5), 49.0 (CH, C-4a), 42.3 (C, C-7a), 42.1 (CH_2, C-6 or 7), 40.6 (CH, C-11), 40.2 (CH_2, C-6 or 7), 36.5 (CH_2, C-8), 20.9 (CH_2, C-4), 19.3 (CH_3, C-17), 18.93** (CH_3, C-15 or 16), 18.89** (CH_3, C-15 or 16), 18.7 (CH_3, C-12 or 13), 18.4 (CH_3, C-12 or 13), 14.7 (CH, C-14), 11.7 (CH_3, C-9 or 10), 11.6 (CH_3, C-9 or 10)

***2 d.p. provided to distinguish peaks that would otherwise be the same at 1 d.p.*

HRMS [TOF-ES$^+$]: calculated for ([M+H]$^+$, $C_{26}H_{47}O_2Si$): 419.3340, found: 419.3336

TIPS acrolein 45

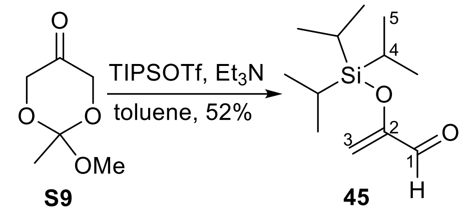

S9 **45**

Procedure adapted from a literature report.[28]

Dioxanone **S9** (256 mg, 1.75 mmol) was dissolved in toluene (3.4 mL) with Et_3N (0.48 mL, 3.42 mmol) and TIPSOTf (0.51 mL, 1.88 mmol), and the resulting solution was stirred for 1 d. At this time, the flask was heated at 40 °C for 1 h, at which point NMR analysis determined completion. H_2O (10 mL) was added, and the mixture was extracted with Et_2O (2 × 10 mL). The organic layers were washed with brine (2 × 10 mL), then dried over Na_2SO_4, filtered, and concentrated under reduced pressure. The crude material was purified by flash column chromatography (2% Et_2O-petroleum ether) to give acrylaldehyde **45** (208 mg, 52%) as a colourless oil.

TLC: 2% Et_2O-petroleum ether, R_f = 0.39 Vanillin

IR (neat): $\tilde{\nu}_{max}$ 2945 m (C-H), 2894 n (C-H), 2868 m (C-H), 1703 sh (C=O), 1615 sh (C=C), 1464 w, 1304, sh, 1036 sh, 881 st, 678 st

^{1}H NMR (400 MHz, $CDCl_3$): δ 9.32 (s, 1H, H-1), 5.49 (d, *J* 1.7, 1H, H-3'), 5.24 (d, *J* 1.7 Hz, 1H, H-3''), 1.28–1.18 (m, 3H, H-4), 1.09 (d, *J* 7.1, 18H, H-5)

^{13}C NMR (101 MHz, $CDCl_3$): δ 189.6 (CH, C-1), 156.4 (C, C-2), 112.2 (CH_2, C-3), 18.0 (CH_3, C-5), 12.8 (CH, C-4)

These data are in agreement with literature reported values.[28]

Oxabicyclic ketones 46–49

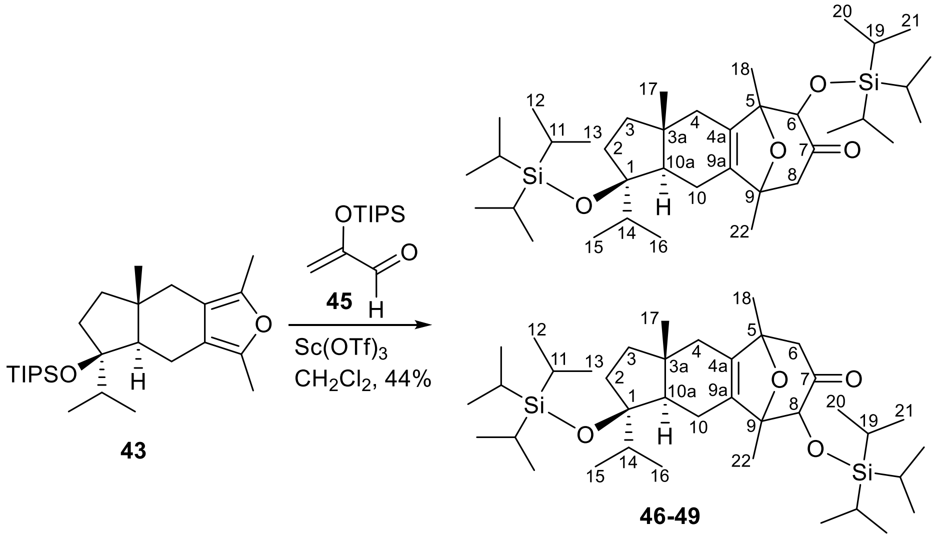

This procedure was adapted from a literature report. [28]

$Sc(OTf)_3$ (30 mg, 61 µmol) was placed in a flask with a stirrer bar and heated at 300 °C under vacuum (10^{-1} mbar) for *ca.* 2 h. Upon cooling to 23 °C, the flask was backfilled with argon, and a solution of furan **43** (257 mg, 0.61 mmol) in CH_2Cl_2 (0.6 mL) was added. The resulting mixture was stirred for 5 min, then a solution of **45** (135 mg, 0.59 mmol) in CH_2Cl_2 (0.6 mL) was added, turning the resulting mixture brown. After 22 h, the reaction mixture was washed with H_2O (15 mL) and re-extracted with Et_2O (3 × 20 mL). The combined organics were washed with brine (3 × 20 mL), dried over $MgSO_4$, filtered, and concentrated under reduced pressure. The crude residue was purified by flash column chromatography (2% → 25% Et_2O-petroleum ether) to give a mixture of ketones **46** and **47** (75 mg, 20%) as a yellow oil, followed by **48** (51 mg, 13%) as a yellow oil, and finally a mixture of **48** and **49** (43 mg, 11%) as a yellow oil.

46 and 47:

*Note: The pictured structure is for the major isomer **46**.*

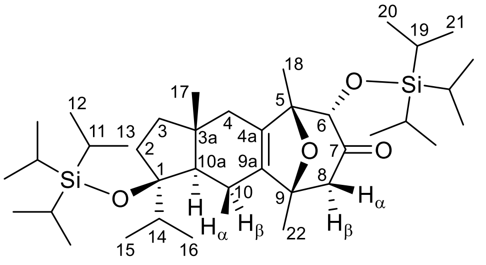

TLC: 5% Et_2O-petroleum ether, R_f = 0.35 Vanillin

IR (thin film, CH_2Cl_2): $\tilde{\nu}_{max}$ 2943 n (C-H), 2866 n (C-H), 1728 sh (C=O), 1464 w, 1150 n, 1113 n, 1065 n (C-O), 670 m

^{1}H NMR (500 MHz, $CDCl_3$): δ 4.29/**4.23** (s, 1H, H-6), 2.56/**2.54** (d, *J* 14.9, 1H, H-8$_\alpha$), 2.47/**2.41** (d, *J* 14.8, 1H, H-8$_\beta$), 2.26 (dddd, *J* 16.2, 11.8, 4.3, 2.8, 1H, H-10$_\alpha$), 2.11−1.94 (st, 4H, H-4, and either H-2 or 3), 1.88 (sept, *J* 6.8, 1H, H-14), 1.66 (ddd, *J* 17.4, 4.8, 2.1, 1H, H-10$_\beta$), 1.58 (dd, *J* 11.9, 7.2, 1H, H-2′ or 3′), 1.50/**1.48** (s, 3H, H-18), 1.37/**1.36** (s, 3H, H-22),1.33 (dd, *J* 11.6, 7.0, 1H, H-10a), 1.19 (*ap.* ddt, *J* 14.1, 8.3, 6.9, 3H, H-19), 1.09 (s, 21H, H-11, 12, and 13), 1.05 (d, *J* 5.6, 10H, H-20 or 21, and either H-2′′ or 3′′), 1.03 (d, *J* 6.2, 9H, H-20 or 21), 0.96 (s, 3H, H-17), 0.88 (d, *J* 6.8, 3H, H-15 or 16), 0.81 (d, *J* 6.8, 3H, H-15 or 16)

**Minor compound signals have been included where possible; in this case the chemical shift in bold indicates the major compound signal, and the integrals throughout reflect the number of major-compound protons per environment; this may alter the order of peaks.*

^{13}C NMR (126 MHz, $CDCl_3$): δ **206.2**/205.9 (C, C-7), 141.8/**140.6** (C, C-9a), **139.7**/139.2 (C, C-4a), **86.7**/85.8 (C, C-5), 85.2/**85.1** (C, C-1), 84.1/**83.9** (C, C-9), 84.0/**83.8** (CH, C-6), 51.4/**50.8** (CH_2, C-8), **48.7**/47.8 (CH, C-10a), **41.63**** (CH_2), 41.55/41.5** (C/CH_2), **41.1** (C, C-3a), 40.2/**40.1** (CH, C-14), 39.9/**39.8** (CH_2, C-2 or 3), **39.1**/38.2 (CH_2), **22.02**/21.97** (CH_2, C-10), **21.4**/21.2 (CH_3, C-22), **19.8**† (CH_3, C-18), 19.1/**19.0** (CH_3, C-17), 18.9/**18.83**/**18.78**** (CH_3, C-12 and 13), 18.7/**18.53**/18.47/**18.42****† (CH_3, C-20, 21 and either 15 or 16), **18.36**** (CH_3, C-15 or 16), **14.4**† (CH, C-11), **13.3**/13.0 (CH, C-19)

**Where possible, sister signals are paired together with the chemical shift in bold denoting the major compound, this may alter the order of peaks.*

***2 d.p. provided to distinguish peaks that would otherwise be the same at 1 d.p.*

†This peak has a distinct shoulder, or a higher amplitude, and likely contains one of the missing signals.

HRMS [TOF-ES$^+$]: calculated for ($[M+H]^+$, $C_{38}H_{71}O_4Si_2$): 647.4885, found: 647.4910

48+49:

Note: In initial experiments, isomers C and D were isolated together and as such the IR and MS data that follows refers to that mixture. However, in the quoted experimental C was partially isolated allowing the NMR data to be given for C and D specifically – this pertains to the suggested absolute structural assignment of both.

TLC: 5% Et_2O-petroleum ether, R_f = 0.30 Vanillin

IR (thin film, CH_2Cl_2): $\tilde{\nu}_{max}$ 2942 n (C-H), 2866 n (C-H), 1730 sh (C=O), 1464 w, 1065 n

HRMS [TOF-ES$^+$]: calculated for ($[M+Na]^+$, $C_{38}H_{70}O_4Si_2Na$): 669.4705, found: 669.4717

48:

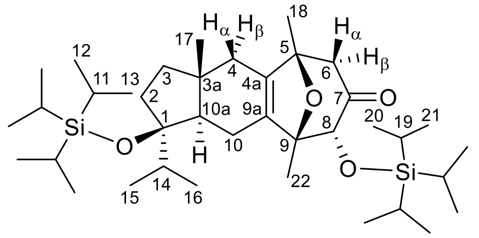

^{1}H NMR (400 MHz, CDCl$_3$): δ 4.28 (s, 1H, H-8), 2.57 (d, *J* 14.9, 1H, H-6$_\alpha$), 2.41 (d, *J* 14.9, 1H, H-6$_\beta$), 2.34–2.21 (m, 1H, H-10$_\alpha$), 2.17–2.10 (m, 1H, H-10$_\beta$), 2.09–1.83 (st, 4H, H-4$_\alpha$, 14, and either H-2 or 3), 1.76–1.67 (m, 1H, H-4$_\beta$), 1.58 (*ap.* q, *J* 6.7, 1H, H-2' or 3'), 1.50 (s, 3H, H-22), 1.43–1.36 (m, 1H, H-10a), 1.33 (s, 3H, H-18), 1.23–1.13 (m, 3H, H-19), 1.11–1.03 (st, 40H, H-11, 12, 13, 20, 21, and H-2'' or 3''), 0.96 (s, 3H, H-17), 0.87 (d, *J* 6.8, 3H, H-15 or 16), 0.82 (d, *J* 6.8, 3H, H-15 or 16)

^{13}C NMR (101 MHz, CDCl$_3$): δ 205.9 (C, C-7), 140.5 (C, C-9a), 139.7 (C, C-4a), 86.2 (C, C-9), 85.5 (C, C-1), 84.1 (CH, C-8), 83.9 (C, C-5), 51.7 (CH$_2$, C-6), 48.2 (CH, C-10a), 41.65** (CH$_2$, C-2 or 3), 40.9 (C, C-3a), 39.87** (CH, C-14), 39.7 (CH$_2$, C-2 or 3), 37.0 (CH$_2$, C-4), 23.2 (CH$_2$, C-10), 22.0 (CH$_3$, C-18), 19.5 (CH$_3$, C-22), 19.1 (CH$_3$, C-17), 18.8 (CH$_3$, C-12 and 13), 18.68/18.54** (CH$_3$, C-15 and 16), 18.46/18.35** (CH$_3$, C-20 and 21), 14.4 (CH, C-11), 12.90** (CH, C-19)

***2 d.p. provided to distinguish peaks that would otherwise be the same at 1 d.p.*

49:

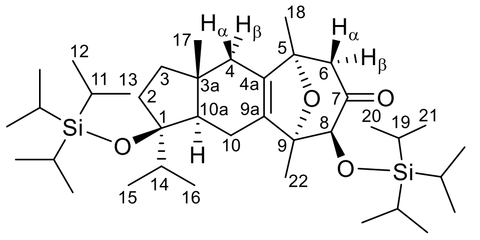

^{1}H NMR (500 MHz, CDCl$_3$): δ 4.24 (s, 1H, H-8), 2.53 (d, *J* 15.1, 1H, H-6$_\beta$), 2.39 (d, *J* 15.1, 1H, H-6$_\alpha$), 2.25–1.83 (st, 6H, H-4$_\beta$, 10, 14, and either H-2 or 3), 1.66 (dd, *J* 16.8, 2.3, 1H, H-4$_\alpha$), 1.61–1.58 (m, 1H, H-2' or 3'), 1.52 (s, 3H, H-22), 1.39 (dd, *J* 11.7, 4.3, 1H, H-10a), 1.34 (s, 3H, H-18), 1.17 (*ap.* td, *J* 8.3, 6.8, 3H, H-19), 1.14–0.99 (st, 40H, H-11, 12, 13, 20, 21, and either H-2'' or 3''), 0.93 (d, *J* 6.7, 3H, H-15 or 16), 0.87 (d, *J* 6.8, 3H, H-15 or 16), 0.79 (s, 3H, H-17)

^{13}C NMR (126 MHz, CDCl$_3$): δ 206.1 (C, C-7), 141.2 (C, C-9a), 140.2 (C, C-4a), 87.0 (C, C-9), 85.1 (C, C-1), 83.8 (CH, C-8), 83.3 (C, C-5), 50.6 (CH$_2$, C-6), 48.3 (CH, C-10a), 41.69** (CH$_2$), 41.2 (C, C-3a), 40.3 (CH, C-14), 39.9 (CH$_2$), 37.5 (CH$_2$, C-4), 23.6 (CH$_2$, C-10), 21.5 (CH$_3$, C-18), 20.1 (CH$_3$, C-22), 19.0 (CH$_3$, C-17), 18.88/18.87** (CH$_3$, C-12 and 13), 18.68** (CH$_3$, C-15 or 16), 18.47/18.42** (CH$_3$, C-20 and 21), 18.33** (CH$_3$, C-15 or 16), 14.2 (CH, C-11), 12.93** (CH, C-19)

**Note: these data were taken from a sample with an isomer ratio of 1 C : 5 D, but having partially isolated C (data above), only peaks corresponding to isomer D are reported here. **2 d.p. provided to distinguish peaks that would otherwise be the same at 1 d.p. In some cases, where isomer C has a peak within the 1 d.p. range.*

Pentachloroacetone 51

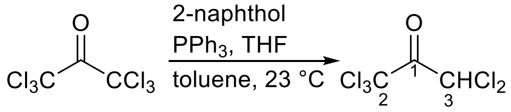

51

Procedure adapted from a literature report.[29]

A solution of hexachloroacetone (9.00 mL, 49.0 mmol) and 2-naphthol (3.60 g, 25.0 mmol) in THF (10 mL) and toluene (20 mL) was added to a solution of PPh$_3$ (6.56 g, 25.0 mmol) in toluene (19 mL) at 0°C over 5 min. A purple solid crashed out almost immediately, and the cooling bath was removed after addition. After 20 min, the solution was filtered and washed with toluene (30 mL total). The filtrate was concentrated under reduced pressure, and then distilled (160 °C, 80-100 mbar) to give a colourless oil (4.15 g, 80-100 mbar). Other fractions totalling 5.49 g were obtained, but deemed less pure by NMR.

Note: See results and discussion for details; the obtained oil was not pure and could not be made so on any occasion and as such a yield is not given. The mixture was used in any relevant cycloaddition.

IR (neat): $\tilde{\nu}_{max}$ 3016 w (C-H), 1780 sh (C=O), 894 sh, 831 s, 643 st

^{1}H NMR (400 MHz, CDCl$_3$): δ 6.74 (s, 1H)

Note: the 6.45 ppm impurity with an integral of 0.05 relative to the 6.74 ppm peak is likely trace sym-TCA, using integrals there is 1:0.025 or 2.4%.

^{13}C NMR (101 MHz, CDCl$_3$): δ 179.7 (C, C-1), 92.6 (C, C-2), 61.9 (CH, C-3)

Note: peaks for hexachloroacetone can be seen at 175.7 ppm and 90.1 ppm.

Oxabicyclic ketone 52

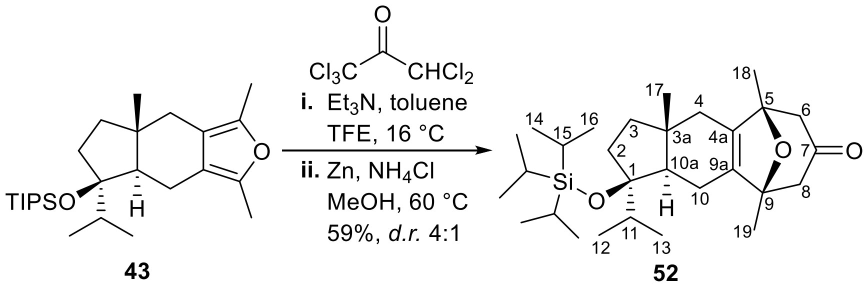

The stereochemistry of C_5 and C_9 are tentatively assigned as (S) and (R) respectively, see main text.

Procedure adapted from a literature report.[30]

Furan **43** (220 mg, 0.53 mmol) was dissolved in toluene (0.26 mL) and 2,2,2-trifluoroethanol (1.0 mL) and the resulting solution was stirred vigorously. Solutions of pentachloroacetone (444 mg, 1.93 mmol) in TFE (3 mL), and Et_3N (total 0.27 mL, 1.93 mmol) in TFE (3 mL) were added simultaneously *via* a syringe pump over 9 h, and the resulting mixture was stirred overnight. Note: lab temperature began at 14 °C and warmed to 18 °C across addition time. The reaction mixture was poured into Et_2O (20 mL), washed with H_2O (3 × 15 mL), and then re-extracted with Et_2O (2 × 20 mL). The combined organic layers were washed with brine (3 × 20 mL), dried over $MgSO_4$, filtered, and finally concentrated under reduced pressure to give a cream solid. This material was used without further purification.

Note: The intermediate chlorides can be purified, but not separated, by flash column chromatography (2.5% Et_2O-petroleum ether) to give a mixture as a white solid.

The crude chlorides were dissolved in MeOH (2.7 mL) with zinc dust (2.07 g, 31.6 mmol) and NH_4Cl (422 mg, 7.89 mmol) and the resulting suspension was heated at 60 °C for 2.5 h. At this time, the reaction mixture was cooled to ambient temperature and filtered through celite to remove the excess solid, eluting with Et_2O (20 mL). The filtrate was washed with a solution of EDTA (0.5 M, pH 8, 3 × 15 mL), re-extracted with Et_2O (15 mL), and the combined organic extracts were washed with brine (3 × 20 mL), dried over $MgSO_4$, filtered, and concentrated under reduced pressure. The crude material was purified by flash column chromatography (25% Et_2O-petroleum ether) to give oxabicyclic ketone **52** (147 mg, 59%, *ca.* 4:1 isomer ratio) as a yellow oil.

TLC: Intermediate chlorides, 10% Et_2O-petroleum ether, R_f = 0.61 Vanillin

Ketone, 25% Et_2O-petroleum ether, R_f = 0.30 Vanillin

IR (neat): $\tilde{\nu}_{max}$ 2928 n (C-H), 2892 n (C-H), 2867 n (C-H), 1715 sh (C=O), 1464 w, 1375 w, 1295 w, 1064 m, 882 m, 670 m

^{1}H NMR (500 MHz, $CDCl_3$): *δ* 2.39 (d, *J* 15.7, 1H, H-6′ or 8′), 2.37 (d, J 16.0, 1H, H-6′ or 8′), 2.35−2.24 (st, 3H, H6′′, 8′′, H-10′), 2.11 (dd, *J* 13.7, 7.7, 1H), 2.03 (dd, *J* 13.3, 7.3, 1H), 2.00−1.87 (st, 2H, H-11 and 1H of CH_2), 1.71−1.64 (st, 2H, H-10′′ and 1H of CH_2), 1.64−1.57 (dd, *J* 12.0, 7.1, 1H), 1.40/**1.37**/**1.36** (s, 2 × 3H, H-18 and 19), 1.56−1.48/**1.31** (m/dd, *J* 11.6, 4.6, 1H, H-10a), **1.09**/1.07 (s/s, 22H, H-14, 15, 16, and 1H of CH_2), **0.99**/0.74 (s, 3H, H-17), 0.93/0.90/**0.88**/**0.83** (d, *J* 6.9, 2 × 3H, H-12 and 13)

**Minor compound signals have been included where possible; in this case the bold frequency indicates the major compound signal, and the integrals throughout reflect the number of major-compound protons per environment; this may alter the order of peaks.*

^{13}C NMR (126 MHz, $CDCl_3$): *δ* 207.9/**207.8** (C, C-7), 141.5/**140.7** (C, C-9a), **139.7**/139.4 (C, C-4a), 85.2/**85.1** (C, C-1), 82.98/**82.97****/**82.9/**82.4 (C, C-5 and 9), **49.8**/**49.5**/49.2 (CH_2, C-6 and 8), **48.9**/47.8 (CH, C-10a), **41.5**/41.38** (CH_2), **41.36**/41.0** (C, C-3a), 40.1/**40.0** (CH, C-11), **39.8**/39.6 (CH_2), **37.0**/36.9 (CH_2), **22.3**/22.2 (CH_3, C-18 or 19), 21.79/**21.77**** (CH_2, C-10), 21.7/**21.6** (CH_3, C-18 or 19), **19.2**/18.9 (CH_3, C-17), **18.82**†/18.79/**18.78**** (CH_3, C-14 and 16), 18.67/**18.65**** (CH_3, C-12 or 13), **18.50**/18.48** (CH_3, C-12 or 13), **14.4**/14.3 (CH, C-15)

**Where possible, sister signals are paired together with the frequency in bold denoting the major compound, this may alter the order of peaks.*

***2 d.p. provided to distinguish peaks that would otherwise be the same at 1 d.p.*

†There are two missing signals for the minor isomer. This peak has a distinct shoulder and likely contains one of the missing signals.

HRMS [TOF-ES$^+$]: calculated for ($[M+H]^+$, $C_{29}H_{51}O_3Si$): 475.3607, found: 475.3619

Tetrabromocyclopropene 56

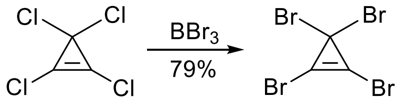

55 **56**

Procedure adapted from a literature report.[31]

Tetrachlorocyclopropene (0.74 mL, 6.00 mmol) was treated with BBr_3 (0.86 mL, 9.01 mmol) dropwise over 5 min. **CAUTION!** A potent exotherm ensued, joined by vigorous effervescence. The crude material was subjected to vacuum distillation (115 °C, 0.1 mbar) to give tetrabromocyclopropene (1.68 g, 79%) as a colourless oil.

Note: the oil freezes to a white solid when stored at/below −25 °C.

IR (neat): $\tilde{\nu}_{max}$ 1761 sh (C=C), 1117 st, 992 st, 651 st, 581 m

^{13}C NMR (101 MHz, $CDCl_3$): δ 121.1 (C, C=C), 24.1 (C, CBr_2)

MS [GCMS-EI$^+$]: *m/z* 356 [$C_3Br^{79}{}_2$ $Br^{81}{}_2$], 275 [$C_3Br^{79}{}_2$ $Br^{81}{}_1$], 196 [$C_3Br^{79}{}_1$ $Br^{81}{}_1$], 115 [$C_3Br^{79}{}_1$]

These data are in agreement with literature reported values.[31]

Tetrachlorocyclopropanes 57a and 58a

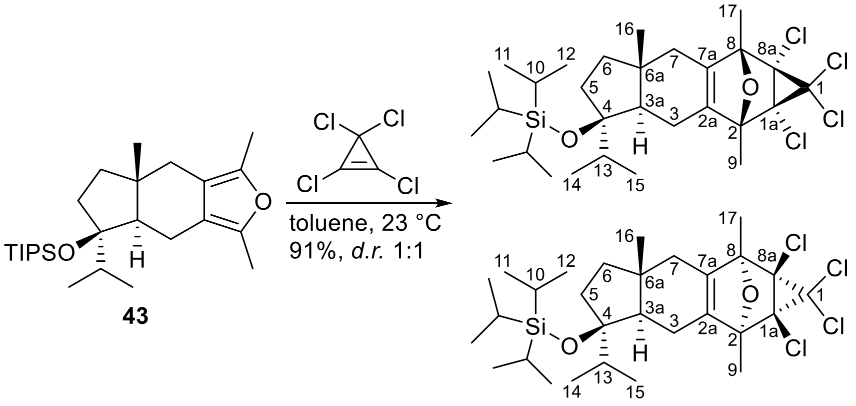

43

57a / 58a

Procedure adapted from a literature report.[32]

A solution of furan **43** (27 mg, 65 µmol) in toluene (0.65 mL) was treated with tetrachlorocyclopropene (0.01 mL, 85 µmol) and the resulting solution was stirred at 23 °C for 3 h. At this time, the solvent was removed under reduced pressure and the crude residue purified by flash column chromatography (1% Et_2O-petroleum ether) to give a mixture of chlorocyclopropanes **57a/58a** (35 mg, 91%, isolated *d.r.* 1:1) as a white solid.

TLC: 5% Et_2O-petroleum ether, R_f = 0.52, 0.50 Vanillin

IR (neat, ATR attachment): $\tilde{\nu}_{max}$ 2940 n (C-H), 2867 n (C-H), 1464 w, 1382 n, 1247 w, 1152 n, 1113 n, 1064 m (C-O), 882 m, 735 m, 671 m, 644 m

^{1}H NMR (400 MHz, $CDCl_3$): δ 2.45−2.26 (st, 2H), 2.24−1.85 (st, 12H), 1.66/1.65/1.63/1.61 (s, 4 × 3H, H-9 and 17), 1.67−1.60 (m, 2 × 1H), 1.51/1.41 (dd, *J* 11.3/11.7, 4.9/4.8, 2 × 1H, H-3a), 1.12−1.05 (st, 44H, H-10, 11, 12, and 2 × 1H of CH_2), 0.97−0.83 (st, 18H, H-14, 15, and 16)

**Where possible, sister signals are paired together, this may alter the order of peaks. Note: Integral values sum to 92H (the sum of both isomers protons).*

^{13}C NMR (101 MHz, $CDCl_3$): δ 150.0/149.0/148.2/148.0 (C, C2a and 7a), 90.7/90.5/90.1/90.0 (C, C-2 and 8), 85.3/84.9 (C, C-4), 67.4/67.14/67.09/67.05** (C, C1a and 8a), 48.7/47.6 (CH, C-3a), 41.9/41.3 (C, C-6a), 41.6/41.5 (CH_2), 40.2/40.1 (CH, C-13), 39.8/39.5/39.0/38.1 (CH_2), 23.8/23.5 (CH_2, C-3), 19.3/19.1 (CH_3, C-16), 18.84/18.80/18.76** (CH_3, C-11 and 12), 18.71/18.65/18.5/18.3** (CH_3, C-14 and 15), 14.4 (CH, C-10), 12.9/12.8/12.7/12.4 (CH_3, C-9 and 17)

**Where possible, sister signals are paired together, this may alter the order of peaks.*

***2 d.p. provided to distinguish peaks that would otherwise be the same at 1 d.p.*

Note: C-1 of both isomers was not observed.

HRMS [TOF-ES$^+$]: calculated for ($[M+H]^+$, $C_{29}H_{47}O_2Si^{35}Cl_4$): 595.2094, found: 595.2074

Tetrabromocyclopropanes 57b and 58b

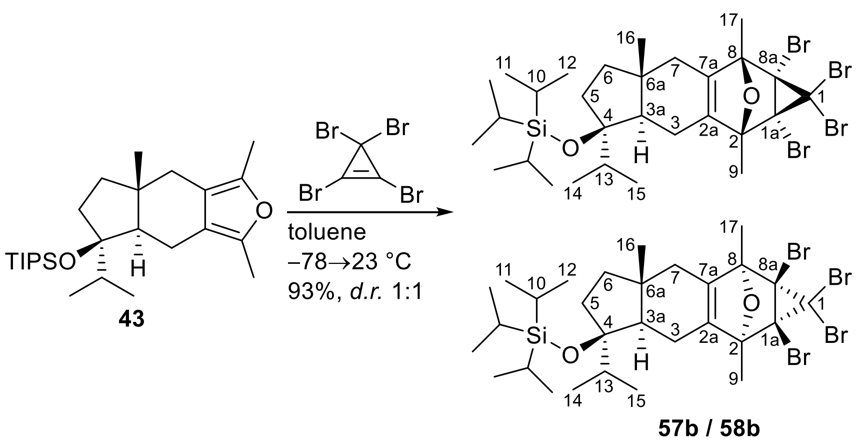

Procedure adapted from a literature report.[32]

A solution of furan **43** (115 mg, 0.28 mmol) in toluene (0.7 mL) was cooled to −78 °C and a solution of tetrabromocyclopropene (109 mg, 0.31 mmol) in toluene (0.7 mL) was added

dropwise over 5 min. The reaction mixture was allowed to warm over several hours to ambient temperature. After 6 h, TBCP (2 drops, neat) was added and the reaction mixture was stirred for 15 h. The solvent was removed under reduced pressure and the crude residue purified by flash column chromatography (2% Et_2O-petroleum ether) to give a mixture of bromocyclopropanes **57b/58b** (197 mg, 93%, isolated *d.r.* 1:1) as a white solid.

TLC: 5% Et_2O-petroleum ether, R_f = 0.56, 0.50 Vanillin

IR (neat, ATR attachment): $\tilde{\nu}_{max}$ 2934 n (C-H), 2866 n (C-H), 1464 w, 1381 n, 1064 n (C-O), 880 n, 672 n, 654 n

^{1}H NMR (400 MHz, $CDCl_3$): δ 2.42−2.25 (m, 1H, H-10'), 2.21−1.97 (st, 5H, H-10'' and 4H of CH_2), 1.69/1.68/1.67/1.65 (s, 4 x 3H, H-9 and 17), 1.61 (*ap.* dd, *J* 7.1, 4.4, 1H), 1.49/1.40 (dd, *J* 11.6/11.4, 4.6/4.8, 1H, H-3a), 1.96−1.88 (m, 1H, H-13), 1.22−0.98 (st, 22H, H-10, 11, 12, and 1H of CH_2), 0.98−0.84 (st, 9H, H14, 15, and 16)

**Where possible, sister signals are paired together, this may alter the order of peaks.*

^{13}C NMR (101 MHz, $CDCl_3$): δ 150.8/149.9/149.0/148.8 (C, C-2a and 7a), 91.5/91.3/90.82/90.79** (C, C-2 and 8), 85.3/84.9 (C, C-4), 61.7/61.3/61.1/61.0 (C, C-1a and 8a), 48.4/47.7 (CH, C-3a), 48.3/48.2 (C, C-1), 42.0/41.3 (C, C-6a), 41.6/41.5 (CH_2), 40.18/40.15** (CH, C-13), 39.8/39.5/39.2/38.2 (CH_2), 24.0/23.7 (CH_2, C-3), 19.4/19.3 (CH_3, C-16), 18.9/18.80** (CH_3, C-11 and 12), 18.75/18.7/18.5/18.4** (CH_3, C-14 and 15), 14.39/14.36** (CH, C-10), 13.2/13.1/12.9/12.7 (CH_3, C-9 and 17)

**Where possible, sister signals are paired together, this may alter the order of peaks.*

***2 d.p. provided to distinguish peaks that would otherwise be the same at 1 d.p.*

HRMS [TOF-ASAP$^+$]: calculated for ($[M+H]^+$, $C_{29}H_{47}O_2{}^{79}Br_3{}^{81}BrSi$): 773.0058, found: 773.0038

Tetrabromides 59 and 60

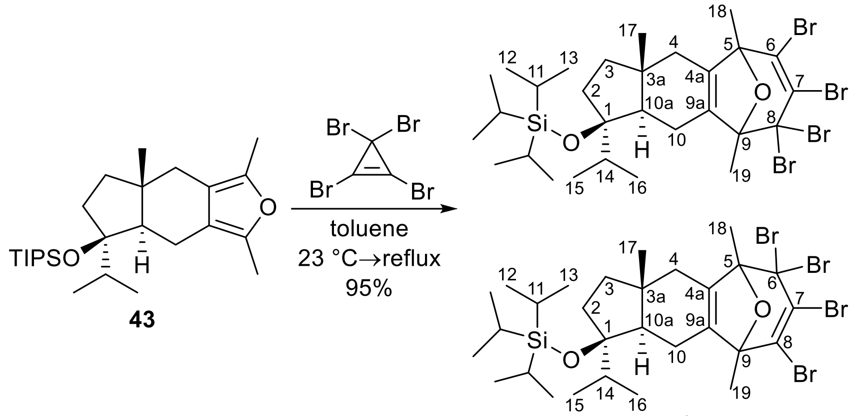

*Note: Both **59** and **60** exist as a pair of facial isomers.*

Procedure adapted from a literature report. [32]

A solution of tetrabromocyclopropene (273 mg, 0.77 mmol) in toluene (1 mL) was added to a solution of furan **43** (281 mg, 0.67 mmol) in toluene (2.4 mL) and the resulting mixture was stirred at ambient temperature for 1 h (until starting material consumption by TLC), then heated at reflux for 5 h*. The solvent was removed under reduced pressure and the crude residue purified by flash column chromatography (2% Et_2O-petroleum ether) to give a mixture of oxabicycles **59** and **60** (492 mg, 95%, isolated *d.r.* 1:1:1:1) as a white solid.

**Note: there is no R_f change from the cyclopropanes to the oxabicycles.*

TLC: 5% Et_2O-petroleum ether, R_f = 0.56, 0.50 Vanillin

IR (neat, ATR attachment): $\tilde{\nu}_{max}$ 2940 n (C-H), 2866 n (C-H), 1566 w, 1464 w, 1376 n, 1063 m (C-O), 741 m, 671 m, 652 m

^{1}H NMR (400 MHz, CDCl$_3$): δ 2.74 (dddd, *J* 17.6, 11.6, 4.6, 2.7, 1H), 2.66−2.56 (st, 2H), 2.50-2.26 (st, 4H), 2.20−1.89 (st, 21H), 1.87/1.86/1.82/1.79 (s, 12H, H-18 or 19), 1.69−1.59 (st, 4H), 1.58/1.56/1.55/1.53 (s, 12H, H-18 or 19), 1.56−1.41 (m, 4H, H-10a), 1.16−1.02 (st, 88H, H-11, 12, 13, and 1H of CH_2), 0.98−0.81 (st, 36H, H-15, 16, and 17)

**Where possible, sister signals are paired together, this may alter the order of peaks.*

^{13}C NMR (101 MHz, CDCl$_3$): δ 150.9/150.7/149.1/148.8 (C, C-4a or 9a), 137.2/137.1/136.0/135.75/135.73/135.6/135.4/135.2** (C, C-4a or 9a, and either C-6/8 or 7), 127.6†/127.5/126.8 (C, C-6/8 or 7), 92.8/92.7/91.5/91.4 and 88.8/88.6/88.1/88.0 (C, C-5 and 9), 85.3/85.2† (C, C-1), 71.9/71.6/71.1/71.0 (C, *C*Br$_2$, C-8/6), 48.24/48.19/47.9/47.6** (CH, C-10a), 42.0/41.5/41.33/40.8** (C, C-3a), 41.7/41.4/41.28/40.7** (CH_2),

40.2/40.1/40.0† (CH, C-14), 39.6/39.4/39.33/39.29/39.0/38.0/37.4** (CH_2), 25.7/24.9/23.13/23.09** (CH_2), 23.2/23.0/22.3†† (CH_3, C-18 or 19), 21.2/20.7/20.4/20.2 (CH_3, C-18 or 19), 19.4/18.72/18.68/18.65/18.6/18.5/18.4** (CH_3, C-15, 16, and 17), 18.83/18.79** (CH_3, C-12 and 13), 14.4†/14.3 (CH, C-11)

**Where possible, sister signals are paired together, this may alter the order of peaks.*

***2 d.p. provided to distinguish peaks that would otherwise be the same at 1 d.p.*

†This peak is suspected of containing more than one signal owing to either a visible shoulder or an enhanced amplitude.

††2D data shows that this peak correlates with 2-CH_3 ^1H environments.

HRMS [TOF-ASAP$^+$]: calculated for ($[M+H]^+$, $C_{29}H_{47}O_2{}^{79}Br_3{}^{81}BrSi$): 773.0058, found: 773.0040

Sulfoxides 66 and 67

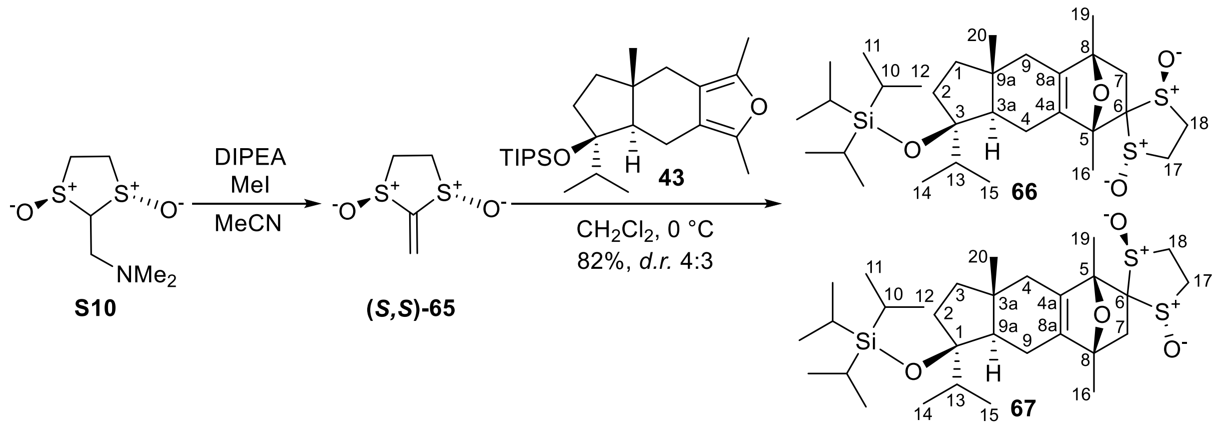

Procedure adapted from a literature report. [3]

DIPEA (0.88 mL, 5.08 mmol) and MeI (0.79 mL, 12.7 mmol) were added to a solution of amine **S10** (495 mg, 2.53 mmol) in MeCN (5 mL). The reaction vessel was shielded from light and stirred overnight. In the morning, an aliquot of the reaction mixture was taken to determine completion by ^1H NMR spectroscopy. Upon completion, the reaction mixture was concentrated to dryness to give a white solid. This material was used without further purification.

Crude sulfoxide (*S,S*)-**65** (1.28 g, *nominally* 8.66 mmol) was dissolved in CH_2Cl_2 (2.5 mL) and the resulting suspension was cooled to 0 °C, then a solution of furan **43** (358 mg, 0.856 mmol) in CH_2Cl_2 (2 mL) was added. An additional portion of (*S,S*)-**65** (64 mg, 0.43 mmol) was added after 1.3 h, and following a further 15 min the reaction was considered complete by TLC. The

reaction mixture was filtered to remove solids, eluting with EtOAc, and the filtrate concentrated under reduced pressure. The crude residue was purified by flash column chromatography (EtOAc → 3% MeOH-EtOAc) to give cycloadducts **66** and **67** (400 mg, 82%, isolated *d.r.* 4:3) as a white solid.

Notes: All solvent removal was done at ca. 25 °C. When the purified cycloadducts had been dissolved in a chlorinated solvent for several hours the solution turned green.

TLC: EtOAc, R_f = 0.30 and 0.23 UV/Vanillin

IR (neat, ATR attachment): $\tilde{\nu}_{max}$ 2933 n (C-H), 2867 n (C-H), 1463 w, 1383 w, 1151 n, 1064 m (C-O), 1038 m (C-O), 671 n

^{1}H NMR (400 MHz, CD_2Cl_2): δ 4.19−3.96/3.56−3.20 (st, 2H/6H, H-17 and 18), 2.56/2.36 (d, *J* 13.0/12.4, 1H, H-7'), 2.51−2.38 (st, 2H), 2.27−2.19 (m, 1H), 2.16−1.84 (st, 10H, H-13 and 8H of CH_2), 1.78-1.72 (m, 1H), 1.76/1.61/1.50/1.47 (s, 3H, H-16 and 19), 1.68−1.57 (st, 3H, H-3a/9a, and 2H of CH_2), 1.49−1.46 (m, 1H, H-3a/9a), 1.37/1.28 (d, *J* 13.0/12.5, 1H, H-7''), 1.20−1.08 (st, 44H, H-10, 11, 12 and 2H of CH_2), 1.01−0.97 (st, 9H, H-20 and either 14 or 15), 0.93 (d, *J* 6.7, 3H, H-14 or 15), 0.88 (*ap.* dd, *J* 7.8, 6.8, 6H, 2 × H-14 or 15)

**Where possible, sister signals are paired together, this may alter the order of peaks.*

^{13}C NMR (101 MHz, CD_2Cl_2): δ 149.7/147.6/142.3/141.7 (C, C-4a and 8a), 101.1/97.1 (C, C-6), 91.3/90.1/87.3/86.7 (C, C-5 and 8), 85.52/85.45** (C, C-3/1), 52.5/52.4/51.1/50.1 (CH_2, C-17 and 18), 49.0/47.9 (CH, C-3a/9a), 41.9/41.8 (CH_2), 41.7/41.22** (C, C-9a/3a), 40.6/40.3 (CH, C-13), 40.0 (CH_2), 39.9 (CH_2), 38.3 (CH_2), 36.7 (CH_2), 36.4/36.1 (CH_2, C-7), 23.1/22.2 (CH_2), 19.7/19.2 (CH_3, C-20), 18.89/18.85**† (CH_3, C-11 and 12), 18.7/18.54/18.46** (CH_3, C-14 and 15), 17.1/16.4/15.8/15.4 (CH_3, C-16 and 19), 14.6 (CH, C-13)

**Where possible, sister signals are paired together, this may alter the order of peaks.*
***2 d.p. provided to distinguish peaks that would otherwise be the same at 1 d.p.*
†2D data shows that this peak/s correlates with more ^{1}H environments than expected.

HRMS [TOF-ES$^+$]: calculated for ($[M+H]^+$, $C_{30}H_{53}O_4SiS_2$): 569.3155, found: 569.3156

Thioacetals 68 and 69

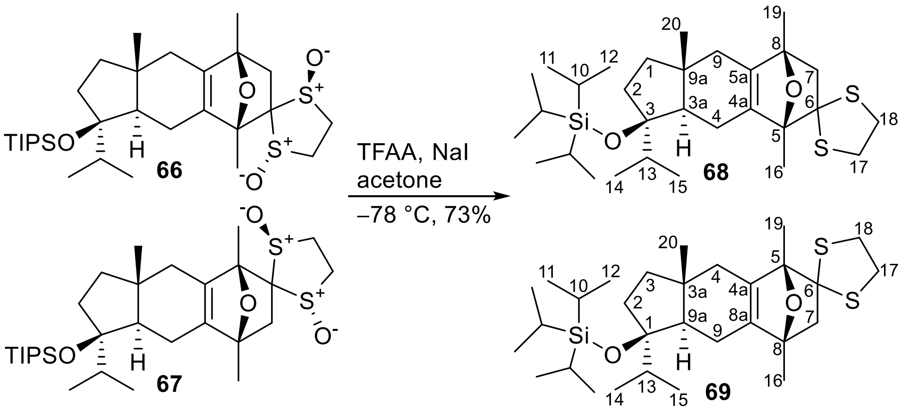

Procedure adapted from a literature report.[3]

A solution of sulfoxides **66** and **67** (131 mg, 0.230 mmol) in acetone (2.3 mL) was cooled to −78 °C. NaI (172 mg, 1.15 mmol) was added to the reaction mixture, followed by the addition of TFAA (0.19 mL, 1.38 mmol) over 10 min. An additional portion of NaI (89 mg, 0.59 mmol) and TFAA (0.10 mL, 0.71 mmol) after 80 min was required for full starting material consumption. After a further 10 min, $Na_2S_2O_3$ (aq.) and $NaHCO_3$ (aq.) were added and the solution allowed to warm to 23 °C. The amount of $Na_2S_2O_3$ added should be sufficient to turn the solution colourless. The reaction mixture was extracted with EtOAc (3 × 15 mL) and the combined organic extracts were washed with brine (2 × 20 mL), then dried over $MgSO_4$, filtered, and finally concentrated under reduced pressure. The crude material was purified by flash column chromatography (5% Et_2O-petroleum ether) to give a mixture of thioacetals **68** and **69** (90 mg, 73%) as a colourless oil.

TLC: 10% Et_2O-petroleum ether, R_f = 0.47 UV/Vanillin

IR (thin film, CH_2Cl_2): $\tilde{\nu}_{max}$ 2927 n (C-H), 2866 n (C-H), 1464 w, 1375 w, 1151 n, 1063 m (C-O), 998 n, 880 n, 670 m

^{1}H NMR (400 MHz, $CDCl_3$): δ 3.32−3.17/3.09−2.99 (st, 6H/2H, H-17 and 18), 2.44−2.24 (st, 6H, H-7 and 2 × 1H of CH_2), 2.22−1.97 (st, 8H), 1.95−1.86 (st, 2H, H-13), 1.80−1.67 (st, 2H), 1.64−1.57 (st, 2H, H-1/3′ or 2′), 1.56/1.53/1.41/1.38 (s, 4 × 3H, H-16 and 19), 1.51−1.43 (st, 2H, H-3a/9a), 1.11−1.04 (st, 44H, H-10, 11, 12, and either H-1/3′′ or 2′′), 0.99/0.96 (s, 2 × 3H, H-20), 0.96 (d, *J* 6.6, 3H, H-14 or 15)/0.92−0.85 (st, 9H, H-14 and 15)

**Where possible, sister signals are paired together, this may alter the order of peaks*

^{13}C NMR (101 MHz, $CDCl_3$): δ 146.0/144.6/143.2/142.5 (C, C-4a and 5a/8a),

91.7/90.7/85.0/84.9 (C, C-5 and 8),85.2/85.1 (C, C-3/1), 75.3/75.2 (C, C-6), 56.21/56.17** (CH_2, C-7), 48.81/48.75** (CH, C-3a/9a), 41.59/41.5** (CH_2), 41.55/41.1** (C, C-9a/3a), 40.84/40.77** (CH_2, C-17 or 18), 40.4 (CH_2), 40.3/40.18** (CH, C-13), 40.24/40.1** (CH_2, C-17 or 18), 40.0/39.9 (CH_2), 36.9 (CH_2), 24.2/21.8 (CH_2, C-9/4), 19.8/19.2 (CH_3, C-20), 18.80/18.77/18.75/18.7**† (CH_3, C-11, 12, and either C-14 or 15), 18.52/18.47** (CH_3, C-14 or 15), 17.4/16.7 (CH_3, C-16 or 19), 14.4/14.3 (CH, C-10), 12.4/11.9 (CH_3, C-16 or 19)

**Where possible, sister signals are paired together, this may alter the order of peaks.*

***2 d.p. provided to distinguish peaks that would otherwise be the same at 1 d.p.*

†2D data shows that this peak/s correlates with more ^1H environments than expected.

HRMS [TOF-ES$^+$]: calculated for ($[M+H]^+$, $C_{30}H_{53}O_2S_2Si$): 537.3256, found: 537.3270

Oxanorbornenones 70 and 71

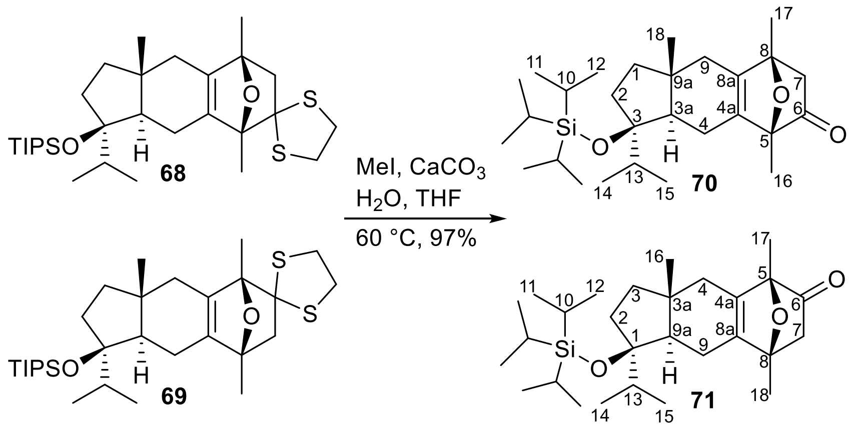

Procedure adapted from a literature report.[33]

Thioacetals **68** and **69** (620 mg, 1.15 mmol) were dissolved in THF (8.6 mL) with H_2O (2.9 mL), $CaCO_3$ (1.15 g, 11.5 mmol) and MeI (7.25 mL, 115 mmol). The resulting solution was heated at 60 °C for 4 d. Upon completion, the reaction mixture was cooled to ambient temperature and filtered through celite, eluting with Et_2O, and washed with H_2O (3 × 30 mL). The aqueous layers were re-extracted with Et_2O (3 × 30 mL), and the combined organic layers were washed with brine (3 × 40 mL), dried over $MgSO_4$, filtered, and concentrated under reduced pressure. The crude material was then purified by flash column chromatography (10% Et_2O-petroleum ether) to give first **71** (233 mg, 44%) as a cream solid, then **70** (219 mg, 41%) as a cream solid. An additional 62 mg (12%) of isomerically-mixed fractions were also obtained.

71:

TLC: 10% Et_2O-petroleum ether, R_f = 0.28 UV/Vanillin

MP: 91−94 °C (Et_2O-petroleum ether)

Optical Rotation: $[\alpha]_D^{25}$ = +223°, (c = 0.5, CH_2Cl_2)

IR (neat, ATR attachment): $\tilde{\nu}_{max}$ 2960 n (C-H), 2941 n (C-H), 2888 n (C-H), 2864 n (C-H), 1755 sh (C=O), 1659 w (C=C), 1463 w, 1380 n, 1077 m (C-O), 1064 m (C-O), 882 m, 672 st

^{1}H NMR (400 MHz, CD_2Cl_2): δ 2.50−2.40 (m, 1H, H-9'), 2.17−2.00 (st, 3H, H-4' and 2H of H-2 or 3), 1.99 (br s, 2H, H-7' and 7''), 1.93 (*ap.* p, *J* 6.8, 1H, H-13), 1.82−1.68 (st, 2H, H-4'' and H-9''), 1.62 (dd, *J* 12.1, 6.9, 1H, either H-2' or 3'), 1.51 (s, 3H, H-18), 1.37−1.33 (m, 1H, H-9a), 1.32 (s, 3H, H-17), 1.11 (s, 22H, H-10, 11, 12, and either H-2'' or 3''), 1.02 (s, 3H, H-16), 0.89 (d, *J* 6.8 Hz, 3H, H-14 or 15), 0.84 (d, *J* 6.8 Hz, 3H, H-14 or 15)

^{13}C NMR (101 MHz, CD_2Cl_2): δ 210.9 (C, C-6), 151.3 (C, C-8a), 138.0 (C, C-4a), 89.3 (C, C-5), 85.3 (C, C-1), 84.9 (C, C-8), 49.3 (CH, C-9a), 41.9 (C, C-3a), 41.8 (CH_2, C-2 or 3), 40.9 (CH_2, C-7), 40.3 (CH, C-13), 39.8 (CH_2, C-2 or 3), 36.8 (CH_2, C-4), 23.2 (CH_2, C-9), 19.3 (CH_3, C-16), 18.87* (CH_3, C-11 or 12), 18.83* (CH_3, C-11 or 12), 18.6 (CH_3, *both* C-14 and 15**), 17.4 (CH_3, C-18), 14.6 (CH, C-10), 11.0 (CH_3, C-17)

**2 d.p. provided to distinguish peaks that would otherwise be the same at 1 d.p.*

***in 2-D NMR experiments, both H-14 and H-15 correlate with this peak, and the peak itself is noticeably broad.*

HRMS [TOF-ES$^+$]: calculated for ($[M+OH]^+$, $C_{28}H_{49}O_4Si$): 477.3400, found: 477.3386

Note: The molecular ion was not observed under mass spectrometry conditions; however, a mass corresponding to the ionised hydrate was and the ketone is inferred from this.

70:

TLC: 10% Et_2O-petroleum ether, R_f = 0.23 UV/Vanillin

MP: 90−92 °C (Et_2O-petroleum ether)

Optical Rotation: $[\alpha]_D^{25}$ = −267°, (c = 0.33, CH_2Cl_2)

IR (neat, ATR attachment): $\tilde{\nu}_{max}$ 2929 n (C-H), 2866, n (C-H), 1753 sh (C=O), 1649 w (C=C), 1391 n, 1062 m (C-O), 676 m

^{1}H NMR (400 MHz, CD_2Cl_2): δ 2.36 (dddd, *J* 17.2, 11.6, 4.9, 2.7, 1H, H-4'), 2.17−2.10 (st, 2H, H-9', and either H-1' or 2'), 2.06 (dd, *J* 13.1, 7.2, 1H, H-1'' or H-2''), 2.01 (br. s, 2H, H-7' and 7''), 1.92 (sept, *J* 6.8, 1H, H-13), 1.79 (ddd, *J* 17.6, 4.9, 2.6, 1H, H-4''), 1.69−1.61 (st, 2H, H-9'', and

either H-1′ or 2′), 1.49 (s, 3H, H-17), 1.35 (s, 3H, H-16), 1.31 (dd, *J* 11.6, 4.9, 1H, H-3a), 1.10 (s, 22H, H-10, 11, 12, and either H-1′′ or 2′′), 1.02 (s, 3H, H-18), 0.87 (d, *J* 6.8, 3H, H-14 or 15), 0.84 (d, *J* 6.8, 3H, H-14 or 15)

^{13}C NMR (101 MHz, CD_2Cl_2): δ 210.2 (C, C-6), 150.5 (C, C-8a), 138.5 (C, C-4a), 89.2 (C, C-5), 85.4 (C, C-3), 84.8 (C, C-8), 49.4 (CH, C-3a), 41.8 (CH_2, C-1 or 2), 41.5 (C, C-9a), 41.1 (CH_2, C-7), 40.2 (CH, C-13), 39.8 (CH_2, C-1 or 2), 38.2 (CH_2, C-9), 21.6 (CH_2, C-4), 19.5 (CH_3, C-18), 18.9 (CH_3, C-11 or 12), 18.8 (CH_3, C-11 or 12), 18.6 (CH_3, C-14 or 15), 18.5 (CH_3, C-14 or 15), 17.9 (CH_3, C-17), 14.6 (CH, C-10), 10.5 (CH_3, C-16)

HRMS [TOF-ES$^+$]: calculated for ($[M+OH]^+$, $C_{28}H_{49}O_4Si$): 477.3400, found: 477.3403

Note: The molecular ion was not observed under mass spectrometry conditions; however, a mass corresponding to the ionised hydrate was and the ketone is inferred from this.

Single crystals of both **215a** and **215b** were obtained by the slow evaporation of acetone. The crystal structures of both compounds can be found in the SI.

Triketone S1

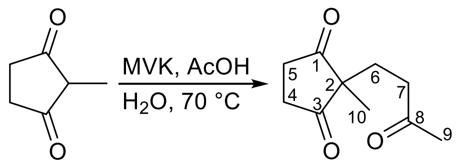

S1

Procedure adapted from a literature report. [12]

2-Methyl-1,3-cyclopentanedione (101.7 g, 0.907 mol), MVK (102 mL, 1.26 mol), and glacial AcOH (2.60 mL, 45.4 mmol) were dissolved in deionised H_2O (202 mL). The reaction vessel was shielded from light and the mixture heated at 70 °C, over which time the solution turns a reddish brown. Upon completion, the reaction mixture was cooled to 23 °C and extracted with CH_2Cl_2 (6 extracts, total volume *ca.* 1 L). The combined extracts were dried over $MgSO_4$, filtered, and the solvent removed under reduced pressure to give a dark brown oil. The crude material was taken forward without further purification.

An analytically pure sample was obtained by vacuum distillation (<1 mbar, 120 °C) to give triketone **S1** as a yellow oil.

TLC: EtOAc, R_f = 0.53 UV / $KMnO_4$

IR (neat): $\tilde{\nu}_{max}$ 2930 w (C-H), 1764 w (C=O), 1712 st (C=O)

^{1}H NMR (400 MHz, CDCl$_3$): δ 2.89−2.66 (st, 4H, H-4 and 5), 2.43 (t, *J* 7.2, 2H, H-7), 2.07 (s, 3H, H-9), 1.86 (t, *J* 7.2, 2H, H-6), 1.08 (s, 3H, H-10)

^{13}C NMR (101 MHz, CDCl$_3$): δ 215.9 (C, C-1 and 3), 207.9 (C, C-8), 55.2 (C, C-2), 37.5 (CH_2, C-7), 34.8 (CH_2, C-4 and 5), 30.1 (CH_3, C-9), 27.9 (CH_2, C-6), 19.2 (CH_3, C-10)

LRMS [TOF-EI$^+$]: *m/z* 182 (35%, $[M]^+$), 164 (15, $[M−H_2O]^+$), 154 (30), 139 (20, $[M−OCCH_3]^+$), 125 (100, $[M−H_3CCOCH_2]^+$), 97 (100, $[C_5H_5O_2]^+$), 69 (80), 55 (80)

These data are in agreement with literature reported values. [12]

Benzyl ether S4

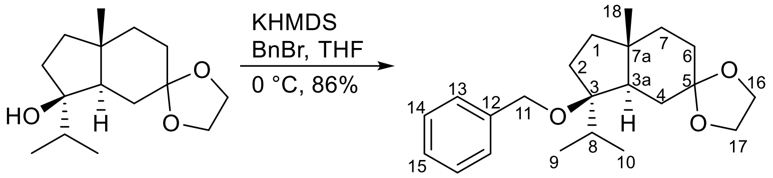

Procedure adapted from a literature report. [10]

KHMDS (1 M in THF, 10 mL, 10 mmol) and BnBr (0.63 mL, 5.30 mmol) were added to a solution of alcohol **7** (1.12 g, 4.42 mmol) in THF (5.4 mL) at −78 °C. After 4 h, a further portion of BnBr (0.15 mL, 1.32 mmol) was added. Upon completion, the reaction mixture was quenched by the addition of $NH_4Cl_{(aq.)}$ (15 mL), diluted with Et_2O (30 mL) and washed with H_2O (3 × 15 mL), then brine (3 × 15 mL). The organic layer was dried over $MgSO_4$, filtered, and concentrated under reduced pressure to give a yellow oil. The crude material was purified by flash column chromatography (10% Et_2O-petroleum ether) to give benzyl ether **S4** (1.31 g, 86%) as a white solid.

TLC: 25% EtOAc-petroleum ether, R_f = 0.58 UV / Vanillin

MP: 91−92 °C (MTBE-acetone)

Optical Rotation: $[\alpha]_D^{25}$ = +67° (c = 0.5, CH_2Cl_2); *Lit.* [10] $[\alpha]_D^{20}$ = +32.2° (c = 1.06, CH_2Cl_2)

IR (neat, ATR attachment): $\tilde{\nu}_{max}$ 2953 (C-H), 2876 (C-H), 1084 m (C-O)

^{1}H NMR (400 MHz, CDCl$_3$): δ 7.37−7.29 (st, 4H, H-13 and 14), 7.26−7.21 (m, 1H, H-15), 4.42 (d, *J* 1.4, 2H, H-11), 4.00−3.91 (st, 4H, H-16 and 17), 2.27 (*ap.* p, *J* 6.8, 1H, H-8), 2.18 (ddd, *J* 14.1, 11.8, 7.9, 1H), 1.97−1.78 (st, 5H, H-3a and 4H of CH_2), 1.67−1.55 (st, 3H), 1.42 (td, *J* 13.4,

4.5, 1H), 1.23−1.11 (m, 1H), 1.09 (s, 3H, H-18), 0.99 (d, *J* 6.8, 3H, H-9 or 10), 0.97 (d, *J* 6.9, 3H, H-9 or 10)

^{13}C NMR (101 MHz, CDCl$_3$): δ 140.3 (C, C-12), 128.2 (CH, C-13 or 14), 126.8 (CH, C-15), 126.7 (CH, C-13 or 14), 110.0 (C, C-5), 87.6 (C, C-3) 64.4 (CH$_2$, C-16 or 17), 64.3 (CH$_2$, C-16 or 17), 62.5 (CH$_2$, C-11), 48.3 (CH, C-3a), 41.8 (C, C-7a), 40.2 (CH$_2$), 36.9 (CH$_2$), 34.4 (CH$_2$), 33.8 (CH$_2$), 33.0 (CH, C-8), 31.7 (CH$_2$), 18.5 (CH$_3$, C-9 or 10), 18.2 (CH$_3$, C-9 or 10), 18.1 (CH$_3$, C-18)

LRMS [TOF-ES$^+$]: *m/z* 367 (100%, [M+Na]$^+$), 237 (50, [M−OBn]$^+$)

These data are in agreement with literature reported values. [10]

Dibenzyl acetal S6

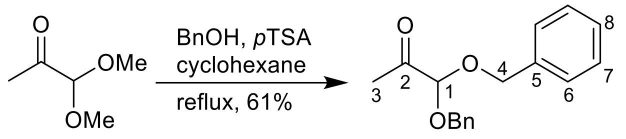

S6

Procedure adapted from a literature report. [23]

Methylglyoxal-1,1-dimethyl acetal (0.59 mL, 5.0 mmol) was dissolved in cyclohexane (12.5 mL) with BnOH (1.1 mL, 11 mmol), and *p*TSA (47 mg, 0.25 mmol), then the resulting mixture was heated at reflux for 90 min. At this time, the solution was cooled and neutralised by the addition of $NaHCO_{3\,(aq.)}$ (20 mL). The organic layer was further washed with $NaHCO_{3\,(aq.)}$ (2 × 20 mL) and brine (3 × 20 mL), dried over $MgSO_4$, filtered, and concentrated under reduced pressure to give a brown oil. The crude material was purified by flash column chromatography (15% Et_2O-petroleum ether) to give dibenzyl acetal **S6** (820 mg, 61%) as a colourless oil.

TLC: 25% Et_2O-petroleum ether, R_f = 0.42 Vanillin

IR (neat): $\tilde{\nu}_{max}$ 3033 w (C-H), 2876 w (C-H), 1728 sh (C=O), 1455 n (C=C), 1048 m (C-O), 1025 m (C-O)

^{1}H NMR (400 MHz, CDCl$_3$): δ 7.39−7.29 (st, 10H, H-6, 7 and 8), 4.73 (s, 1H, H-3), 4.68 (d, *J* 11.8, 2H, H-4’), 4.59 (d, *J* 11.8, 2H, H-4’’), 2.25 (s, 3H, H-1)

^{13}C NMR (101 MHz, CDCl$_3$): δ 204.0 (C, C-2), 137.0 (C, C-5), 128.7 (CH), 128.2 (CH), 101.1 (CH, C-3), 69.4 (CH$_2$, C-4), 25.2 (CH$_3$, C-1)

These data are in agreement with literature reported values, with the exception of one carbon environment being unobserved *ca.* 127 ppm. [23]

TIPS ether S7

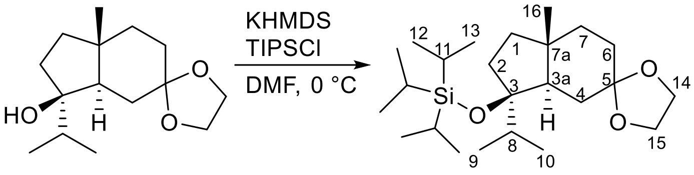

Procedure adapted from the benzylation protocol detailed previously.

KHMDS (1 M in THF, 3.00 mL, 3.00 mmol) was added to a solution of alcohol **7** (500 mg, 1.97 mmol) in DMF (5 mL) and the resulting solution was cooled to 0 °C. TIPSCl (0.46 mL, 2.2 mmol) was added and the flask was raised from the cooling bath. Upon completion, the reaction mixture was diluted with Et_2O (20 mL) and washed with HCl (1 M, 3 × 15 mL). The aqueous layer was re-extracted with Et_2O (3 × 15 mL), and the combined organic layers were washed with brine (3 × 15 mL), then dried over $MgSO_4$, filtered, and concentrated under reduced pressure. The crude material was used without further purification.

An analytically pure sample was obtained by flash column chromatography (5% Et_2O-petroleum ether) to give silyl ether **S7** as a colourless oil.

TLC: 50% Et_2O-petroleum ether, R_f = 0.68 Vanillin

Optical Rotation: $[\alpha]_D^{25}$ = +28°, (c = 0.55, CH_2Cl_2)

IR (neat): $\tilde{\nu}_{max}$ 2944 n (C-H), 2866 n (C-H), 1462 w, 1079 m (C-O), 882 n, 670 m

^{1}H NMR (400 MHz, $CDCl_3$): δ 3.99−3.89 (st, 4H, H-14 and 15), 2.12−1.95 (st, 2H), 1.91 (*ap.* p, *J* 6.8, 1H, H-8), 1.87−1.71 (st, 3H), 1.68−1.47 (st, 5H, H-3a and 4H of CH_2), 1.35 (*ap.* td, *J* 13.1, 4.4, 1H), 1.12−1.01 (st, 24H, H-11, 12, 13 and 16), 0.95 (d, *J* 6.8, 3H, H-9 or 10), 0.93 (d, *J* 6.7, 3H, H-9 or 10)

**There is an impurity at ca. 0 ppm that is difficult to remove at this stage*

^{13}C NMR (101 MHz, $CDCl_3$): δ 111.3 (C, C-5), 86.1 (C, C-3), 64.4 (CH_2, C-14 or 15), 64.3 (CH_2, C-14 or 15), 48.7 (CH, C-3a), 41.6 (CH_2), 41.5 (C, C-7a), 40.5 (CH_2), 40.1 (CH, C-8), 36.8 (CH_2), 34.3 (CH_2), 31.8 (CH_2), 19.0 (CH_3, C-16), 18.9 (CH_3, C-12 or 13), 18.8* (CH_3, C-12 or 13), 18.3 (CH_3, C-9 or 10), 14.5 (CH, C-11)

**Note: Either C-9 or 10 was not observed most likely because it co-incides with the signals for the TIPS group and in fact a shoulder can be seen at 18.8 ppm.*

HRMS [TOF-ES$^+$]: calculated for ($[M+H]^+$, $C_{24}H_{47}O_3Si$): 411.3289, found: 411.3262

Dimethylamine S10

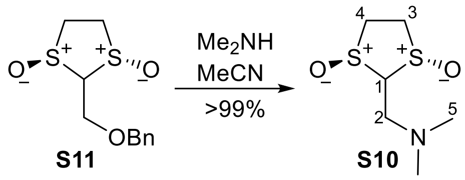

Procedure adapted from a literature report. [3][34]

Me_2NH (18.0 mL, 36.0 mmol, 2 M in THF) was added to a solution of benzyl ether **S11** (1.86 g, 7.20 mmol) in MeCN (28 mL) and the flask was protected from light. After 16 h, a further portion of Me_2NH (18.0 mL, 36.0 mmol) was added. After stirring for 4 d, the solvent and excess reagent were removed under reduced pressure, and the resulting residue was purified by flash column chromatography (EtOAc → 10% MeOH-EtOAc, only 6 cm of silica gel) to give amine **S10** (1.41 g, >99%) as a white solid.

TLC: 10% MeOH-EtOAc, R_f = 0.08 UV / $KMnO_4$

^{1}H NMR (400 MHz, $CDCl_3$): δ 3.94 (t, J 8.8, 1H, H-1), 3.81–3.59 (st, 4H, H-3 and 4), 2.95 (d, J 8.6, 2H, H-2), 2.41 (s, 6H, H-5)

^{13}C NMR (101 MHz, $CDCl_3$): δ 90.7 (CH, C-1), 52.3 (CH_2, C-2), 51.5 (CH_2, C-3 or 4), 51.0 (CH_2, C-3 or 4), 45.7 (CH_3, C-5)

HRMS [TOF-ES$^+$]: calculated for ($[M+H]^+$, $C_6H_{14}O_2S_2N$): 196.0466, found: 196.0462

Rac/(*S,S*)-bissulfoxide S11

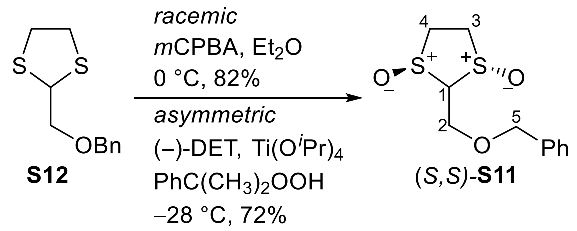

**stereochemistry drawn for the asymmetric reaction product*

Procedure adapted from a literature report. [3][34]

Racemic Synthesis:

A solution of *m*CPBA (3.94 g, 22.8 mmol) in Et_2O (57 mL) was added to a solution of thioacetal

S12 (2.05 g, 9.06 mmol) in Et_2O (24 mL) over 90 min *via* a syringe pump. Approximately 20 min into addition, a white solid began to precipitate from solution. Upon completion, the solid was obtained by filtration and purified by recrystallisation from EtOAc, being careful to cool from reflux to −28 °C (freezer) over 4 h. The racemic sulfoxide **S11** (1.86 g, 82%) was obtained as white plate crystals.

Asymmetric Synthesis of the (S,S) or (−)-Isomer:

$Ti(O^iPr)_4$ (1.30 mL, 4.42 mmol) was added to a solution of (−)-DET (3.00 mL, 17.7 mmol) in CH_2Cl_2 (20 mL) and the resulting yellow solution was stirred for a further 30 min. At this point, thioacetal **S12** (2.02 g, 8.92 mmol) was added with the aid of CH_2Cl_2 (15 mL) and the resulting solution was cooled to −40 °C using a carefully managed dry ice-acetone bath. After stirring at this temperature for 1 h, cumyl hydroperoxide (3.30 mL, 17.7 mmol, 80% technical grade) was added over 5 min, and following 20 min of further stirring, the reaction vessel was transferred to a freezer (−28 °C, no stirring) for 3 d. Upon completion, H_2O (1.50 mL) was added and the reaction mixture was stirred vigorously as it warmed to 23 °C over 2 h. After this period, the reaction mixture was filtered through celite (4 cm by 3.5^2 cm) washing with CH_2Cl_2. It should be noted that the initial gel was reluctant to filter until it was mechanically stirred into the celite, at which point fluid rushed through. The filtrate was concentrated under reduced pressure to give *ca.* 9 g of crude material, that purified by flash column chromatography (5% → 10% MeOH-EtOAc) to give the (*S,S*)-bissulfoxide **S11** (1.65 g, 72%) as a white crystalline solid.

TLC: 10% MeOH-EtOAc, R_f = 0.62 UV / $KMnO_4$

Optical Rotation: $[\alpha]_D^{25}$ = −116°, (c = 1.0, $CHCl_3$); *Lit.* [3] $[\alpha]_D^{25}$ = +125.4°, (c = 1.0, $CHCl_3$) for (*R,R*)-isomer

IR (neat, ATR attachment): $\tilde{\nu}_{max}$ 3059 w (C-H), 2976 n (C-H), 2861 w (C-H), 1453 n (C=C), 1015 st, 732 st, 696 st

^{1}H NMR (400 MHz, $CDCl_3$): δ 7.41−7.29 (st, 5H, Ar), 4.66 (d, *J* 12.0, 1H, H-5’), 4.60 (d, *J* 11.9, 1H, H-5’’), 4.14−4.05 (st, 2H, H-2), 4.04−3.96 (m, 1H, H-1), 3.83−3.61 (st, 4H, H-3 and 4)

^{13}C NMR (101 MHz, $CDCl_3$): δ 137.0 (C, Ar), 128.7 (C, Ar), 128.3 (C, Ar), 128.1 (C, Ar), 90.1 (CH, C-1), 74.1 (CH_2, C-2 or 5) , 62.0 (CH_2, C-2 or 5), 51.9 (CH_2, C-3 or 4), 51.5 (CH_2, C-3 or 4)

HRMS [TOF-ES$^+$]: calculated for ($[M+Na]^+$, $C_{11}H_{14}O_3S_2Na$): 281.0282, found: 281.0287

These data are in agreement with literature reported values. [3][34]

Benzyl ether S12

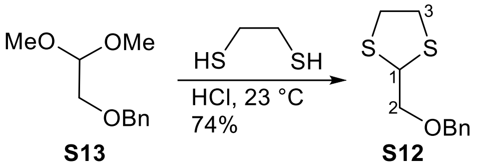

Procedure adapted from a literature report. [3][34]

Ethanedithiol (4.80 mL, 57.0 mmol) and HCl (37.5%, 3.60 mL) were cooled to 0 °C and acetal **S13** (10.92 g, 55.6 mmol) was added dropwise over 2 h *via* a syringe pump, causing the solution to cloud. The cooling bath was removed after addition, and after a further 45 min TLC indicated completion. The reaction mixture was poured (**Careful!**) into H_2O (25 mL) and extracted with CH_2Cl_2 (3 × 25 mL). The organic extracts were washed with H_2O (2 × 25 mL), $NaHCO_3$ (aq.) (2 × 25 mL), and brine (1 × 20 mL), then were dried over $MgSO_4$, filtered and concentrated under reduced pressure. The crude material was purified by flash column chromatography (8% Et_2O-petroleum ether) to give dithiolane **S12** (9.37 g, 74%) as a colourless oil.

TLC: 10% Et_2O-petroleum ether, R_f = 0.33 UV / $KMnO_4$

IR (neat): $\tilde{\nu}_{max}$ 3028 w (C-H), 2923 w (C-H), 2852 w (C-H), 1495 w (C=C), 1096 st (C-O), 734 st, 696 st

^{1}H NMR (400 MHz, CDCl$_3$): δ 7.37−7.33 (st, 4H, Ar), 7.33−7.27 (m, 1H, Ar), 4.63 (t, *J* 7.0, 1H, H-1), 4.60 (s, 2H, OBn), 3.57 (d, *J* 7.0, 2H, H-2), 3.20 (s, 4H, H-3)

^{13}C NMR (101 MHz, CDCl$_3$): δ 138.1 (C, Ar), 128.6 (C, Ar), 127.9 (C, Ar), 127.8 (C, Ar), 75.3 (CH_2, C-2), 73.4 (CH_2, OBn), 51.9 (CH, C-1), 38.2 (CH_2, C-3)

These data are in agreement with literature reported values. [3][34]

1-Bromo-1-methoxypropan-2-one S14

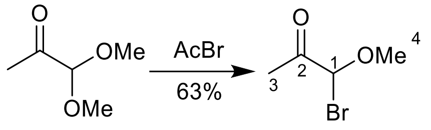

Procedure adapted from a literature report.[35]

AcBr (0.81 mL, 11.0 mmol) was added to methylglyoxal 1,1-dimethyl acetal (1.20 mL, 10.0 mmol) at 0 °C and the resulting solution stirred at 23 °C for 1 h. At this time, the mixture was distilled (*ca.* 170 mbar, 50 °C) to give bromide **S14** (1.06 g, 63%) as a pale-yellow oil.

Note: It was found that the oil fumes in air and turns yellow rapidly at room temperature. It was stored in a freezer under argon when not in use, but subsequent reactions work better if it is used immediately after distillation.

IR (neat): $\tilde{\nu}_{max}$ 2941 w (C-H), 2843 w (C-H), 1727 st (C=O), 1356 m, 1224 m, 1090 st (C-O), 626 st

^{1}H NMR (400 MHz, $CDCl_3$): δ 5.95 (s, 1H, H-1), 3.59 (s, 3H, H-4), 2.37 (s, 3H, H-3)

^{13}C NMR (101 MHz, $CDCl_3$): δ 197.4 (C, C-2), 92.4 (CH, C-1), 59.0 (CH_3, C-4), 23.0 (CH_3, C-3)

These data are in agreement with literature reported values.[35]

1-Methoxy-1-((*R*)-1-(naphthalen-2-yl)ethoxy)propan-2-one S15

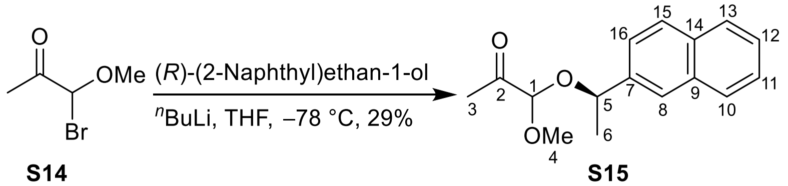

Procedure adapted from a literature report.[36]

(*R*)-(2-Naphthyl)ethan-1-ol (491 mg, 2.85 mmol) in THF (6 mL) was cooled to 0 °C, at which point ^{n}BuLi (2.39 M in hexanes, 1.43 mL, 3.42 mmol) was added resulting in a yellow solution. After 15 min, bromide **S14** (520 mg, 3.13 mmol, prepared earlier that day) in THF (5 mL) was added over 5 min and the cooling bath was removed. After 3 h, the reaction mixture was quenched by the addition of $NaHCO_{3\,(aq.)}$ (2 mL), then diluted with H_2O (20 mL) and extracted with EtOAc (3 × 30 mL). The organic layers were washed with brine (3 × 25 mL), then dried

over $MgSO_4$, filtered, and concentrated under reduced pressure. The crude material was purified by flash column chromatography (15% Et_2O-petroleum ether) to give a 1:1 mixture of diastereomers of acetal **S15** (213 mg, 29%) as a yellow oil.

Note: 128 mg of naphthylethanol was returned, the adjusted yield of the mixed acetal was therefore 39%.

TLC: 25% Et_2O-petroleum ether, R_f = 0.32 UV / Vanillin

IR (neat): $\tilde{\nu}_{max}$ 3056 w (C-H), 2976 w (C-H), 2930 w (C-H), 2834 w (C-H), 1728 m (C=O), 1602 w (C=C), 1102 m, 1059 st (C-O), 1032 st (C-O)

^{1}H NMR (400 MHz, $CDCl_3$): δ 7.90−7.74 (st, 8H, Ar), 7.57−7.45 (st, 6H, Ar), 4.99/4.80 (q, *J* 6.6, 1H, H-5), 4.46/4.41 (s, 1H, H-1), 3.36/3.21 (s, 3H, H-4), 2.23/2.18 (s, 3H, H-3), 1.63/1.58 (d, *J* 6.6, H-5)

^{13}C NMR (101 MHz, $CDCl_3$): δ 204.4/204.0 (C, C-2), 140.0/139.4 (C, C-7), 133.4 (C, C-9 or 14), 133.29** (C, C-9 or 14), 133.26**† (C, C-9 or 14), 128.9 (CH, Ar), 128.6 (CH, Ar), 128.1 (CH, Ar), 128.0 (CH, Ar), 127.9 (CH, Ar), 127.8 (CH, Ar), 126.5 (CH, Ar), 126.4 (CH, Ar), 126.3 (CH, Ar), 126.2 (CH, Ar), 126.1 (CH, Ar), 125.7 (CH, Ar), 124.3 (CH, Ar), 124.2 (CH, Ar), 102.5/100.9 (CH, C-1), 76.0/75.5 (CH, C-5), 55.5/54.0 (CH_3, C-4), 25.2 (CH_3), 24.3 (CH_3), 23.8 (CH_3), 23.4 (CH_3)

**Compound exists as a pair of diastereomers, as such sister signals have been paired where possible.*

***2 d.p. provided to distinguish peaks that would otherwise be the same at 1 d.p. †Peak is twice as high and close inspection shows a shoulder, it is likely that the missing C-9/14 environment is here.*

LRMS [TOF-ES$^+$]: *m/z* 313 (90%, $[M+Na+MeOH]^+$), 281 (100, $[M+Na]^+$), 155 (25, $[NaphCH_2CH_3]^+$)

These data are in agreement with literature reported values.[36]

Triethyl((3-methoxy-3-((*R*)-1-(naphthalen-2-yl)ethoxy)prop-1-en-2-yl)oxy)silane S16

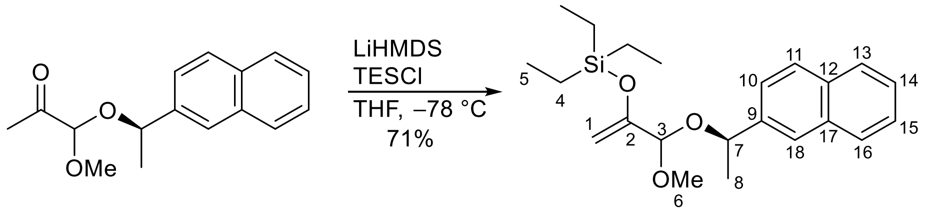

Procedure adapted from a literature report. [36]

Ketone **S15** (52 mg, 0.20 mmol) was dissolved in THF (0.95 mL) and cooled to −78 °C. TESCl (0.05 mL, 0.29 mmol), then LiHMDS (1 M in THF, 0.23 mL, 0.23 mmol) were added. After 45 min, the reaction mixture was quenched by the addition of $NaHCO_{3\,(aq.)}$ (2 mL), then diluted with H_2O (10 mL) and extracted with Et_2O (3 × 10 mL). The organic layers were washed with brine (2 × 10 mL), then dried over Na_2SO_4, filtered, and concentrated under reduced pressure. The crude material was purified by flash column chromatography (95:4:1 petroleum ether-Et_2O-Et_3N) to give a diastereomeric mixture of silyl enol ethers **S16** (53 mg, 71%) as a colourless oil.

TLC: 25% Et_2O-petroleum ether, R_f = 0.65 UV / Vanillin

IR (neat): $\tilde{\nu}_{max}$ 3057 w (C-H), 2955 n (C-H), 2911 n (C-H), 2877 n (C-H), 1640 sh (C=O), 1510 w (C=C), 1050 m (C-O), 1018 m (C-O), 747 st

^{1}H NMR (400 MHz, CD_2Cl_2): δ 7.87−7.81 (st, 6H, Ar), 7.79 (dd, *J* 1.8, 0.7, 1H, Ar), 7.77 (*ap.* dt, *J* 1.8, 0.7 Hz, 1H, Ar), 7.56 (dd, *J* 8.5, 1.7, 1H, Ar), 7.52−7.43 (st, 5H, Ar), 4.99/4.83 (q, *J* 6.6, 1H, H-7), 4.61/4.49 (d, *J* 0.7, 1H, H-3), 4.59/4.58 (dd, *J* 1.2, 0.6, 1H, H-1), 4.35/4.33 (d, *J* 1.2, 1H, H-1'), 3.26/3.16 (s, 3H, H-6), 1.55/1.53 (d, *J* 2.3, 3H, H-8), 1.01−0.96/0.95−0.90 (st, 9H, H-5), 0.75−0.63 (st, 12H, H-4)

^{13}C NMR (101 MHz, CD_2Cl_2): δ 154.9/154.6 (C, C-2), 141.8/141.2 (C, C-9), 133.69/133.65** (C, C-12 or 17), 133.5/133.4 (C-12 or 17), 128.6 (CH, Ar), 128.4 (CH, Ar), 128.24** (CH, Ar), 128.20** (CH, Ar), 127.99** (CH, Ar), 127.98** (CH, Ar), 126.42** (CH, Ar), 126.41** (CH, Ar), 126.13* (CH, Ar), 126.11** (CH, Ar), 125.7 (CH, Ar), 125.4 (CH, Ar), 125.0 (CH, Ar), 124.9 (CH, Ar), 101.0/99.2 (CH, C-3), 92.2/92.0 (CH_2, C-1), 75.0/74.4 (CH, C-7), 54.1†/52.5 (CH_3, C-6), 24.3/23.4 (CH_3, C-8), 6.80/6.75** (CH_3, C-5), 5.16/5.13** (CH_2, C-4)

***2 d.p. provided to distinguish peaks that would otherwise be the same at 1 d.p.*

†signal merges with solvent in UDEFT, but JMOD shows a signal here, as such assignment is tentative

LRMS [TOF-ES$^+$]: *m/z* 767 (70%, $[2M+Na]^+$), 395 (100, $[M+Na]^+$)

These data are in agreement with literature reported values. [36]

Tosylate S17

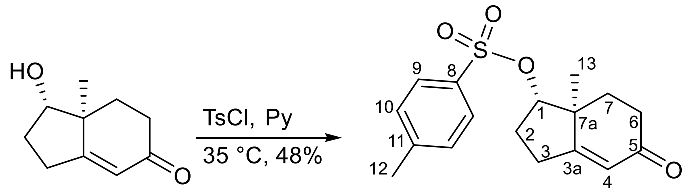

Procedure adapted from a literature report.[37]

TsCl (7.9 g, 41.4 mmol) was added to a solution of alcohol (+)-**15** (4.15 g, 25.0 mmol) in pyridine (35 mL) at 23 °C. The solution was then heated at 35 °C for 4 h. Upon completion, the reaction mixture was cooled to 23 °C and the solvent removed under reduced pressure. The residue was taken up in Et_2O (75 mL) and washed with $HCl_{(aq.)}$ (20 mL, 2 M), then $NaHCO_3$ $_{(aq.)}$ (15 mL), and finally brine (15 mL). The combined organic layers were dried over $MgSO_4$, filtered, and concentrated under reduced pressure. The crude material was purified by flash column chromatography (20% EtOAc-hexane) to give tosylate **S17** (3.81 g, 48%) as a white solid.

TLC: 50% EtOAc-hexane, R_f = 0.26 UV

MP: 102−107 °C (hexane-EtOAc)

IR (neat, ATR attachment): $\tilde{\nu}_{max}$ 2958 w (C-H), 2945 w (C-H), 1659 st (C=O), 1171 st, 984 st

^{1}H NMR (400 MHz, $CDCl_3$): δ 7.82–7.76 (m, 2H, H-9), 7.39–7.32 (m, 2H, H-10), 5.75 (*ap.* t, *J* 2.1, 1H, H-4), 4.39 (dd, *J* 9.9, 8.0, 1H, H-1), 2.72 (ddt, *J* 19.6, 11.6, 2.7, 1H, H-3’), 2.45 (s, 3H, H-12), 2.47–2.25 (st, 3H, H-6 and H-3’’), 2.12–1.97 (st, 2H, H-2), 1.91 (ddd, *J* 13.2, 5.4, 2.1, 1H, H-7’), 1.58 (td, *J* 13.7, 5.1, 1H, H-7’’), 1.18 (d, *J* 0.7, 3H, H-13)

^{13}C NMR (101 MHz, $CDCl_3$): δ 198.2 (C, C-5), 170.7 (C, C-3a), 145.2 (C, C-11), 133.7 (C, C-8), 130.0 (CH, C-10), 128.0 (CH, C-9), 124.2 (CH, C-4), 87.5 (CH, C-1), 44.9 (C, C-7a), 33.6 (CH_2, C-7), 33.0 (CH_2, C-6), 26.8 (CH_2, C-2), 26.3 (CH_2, C-3), 21.8 (CH_3, C-12), 16.5 (CH_3, C-

13)

These data are in agreement with literature reported values.[37]

Tosylate acetal S18

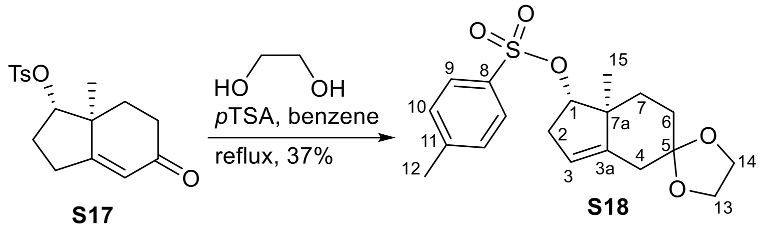

Procedure adapted from a literature report. [9]

Ethylene glycol (0.61 mL, 10.9 mmol) and *p*TSA (40 mg, 0.23 mmol) were added to a solution of enone **S17** (0.67 g, 2.10 mmol) in benzene (25 mL) and the resulting mixture was heated at reflux under a Dean-Stark apparatus for 18 h. Upon completion, the reaction mixture was cooled to 23 °C and the solvent removed under reduced pressure. The residue was taken up in Et_2O (10 mL) and washed with H_2O (10 mL). The aqueous layer was re-extracted with Et_2O (2 × 5 mL) and the combined organic layers were dried over Na_2SO_4, filtered, and concentrated under reduced pressure. The crude material was purified by flash column chromatography (40% Et_2O-petroleum ether) to give acetal **S18** (0.28 g, 37%) as a white solid.

TLC: 50% Et_2O-petroleum ether, R_f = 0.39 UV

MP: 106–110 °C (Et_2O-petroleum ether)

IR (neat, ATR attachment): $\tilde{\nu}_{max}$ 2958 w (C-H), 2945 w (C-H), 1357 m, 1093 st, 983 st

^{1}H NMR (400 MHz, CDCl$_3$): *δ* 7.82–7.75 (m, 2H, H-9), 7.35–7.30 (m, 2H, H-10), 5.21–5.05 (m, 1H, H-3), 4.61 (t, *J* 8.3, 1H, H-1), 3.98–3.84 (st, 4H, H-13 and 14), 2.44 (s, 3H, H-12), 2.40 (*ap.* dt, *J* 8.4, 2.1, 2H, H-2), 2.32 (*ap.* t, *J* 1.6, 2H, H-4), 1.76 (td, *J* 14.1, 13.4, 4.5, 1H, H-6’), 1.69–1.59 (m, 2H, H-6’’ and H-7’), 1.36 (td, *J* 14.5, 13.9, 4.5, 1H, H-7’’), 1.07 (s, 3H, H-15)

^{13}C NMR (101 MHz, CDCl$_3$): *δ* 144.9 (C, C-3a or 11), 144.7 (C, C-3a or 11), 134.0 (C, C-8), 129.9 (CH, C-10), 128.1 (CH, C-9), 118.6 (CH, C-3), 108.9 (C, C-5), 89.6 (CH, C-1), 64.62** (CH_2, C-13 or 14), 64.57** (CH_2, C-13 or 14), 46.4 (C, C-7a), 36.7 (CH_2, C-4), 36.0 (CH_2, C-2), 35.2 (CH_2, C-7), 31.1 (CH_2, C-6), 21.8 (CH_3, C-12), 16.0 (CH_3, C-15)

***2 d.p. provided to distinguish peaks that would otherwise be the same at 1 d.p.*

HRMS [TOF-ES$^+$]: calculated for ([M+H]$^+$, $C_{19}H_{25}O_5S$): 365.1423, found: 365.1427

NMR Spectra

Diol 5

^{1}H NMR Spectrum, C_6D_6, 400 MHz

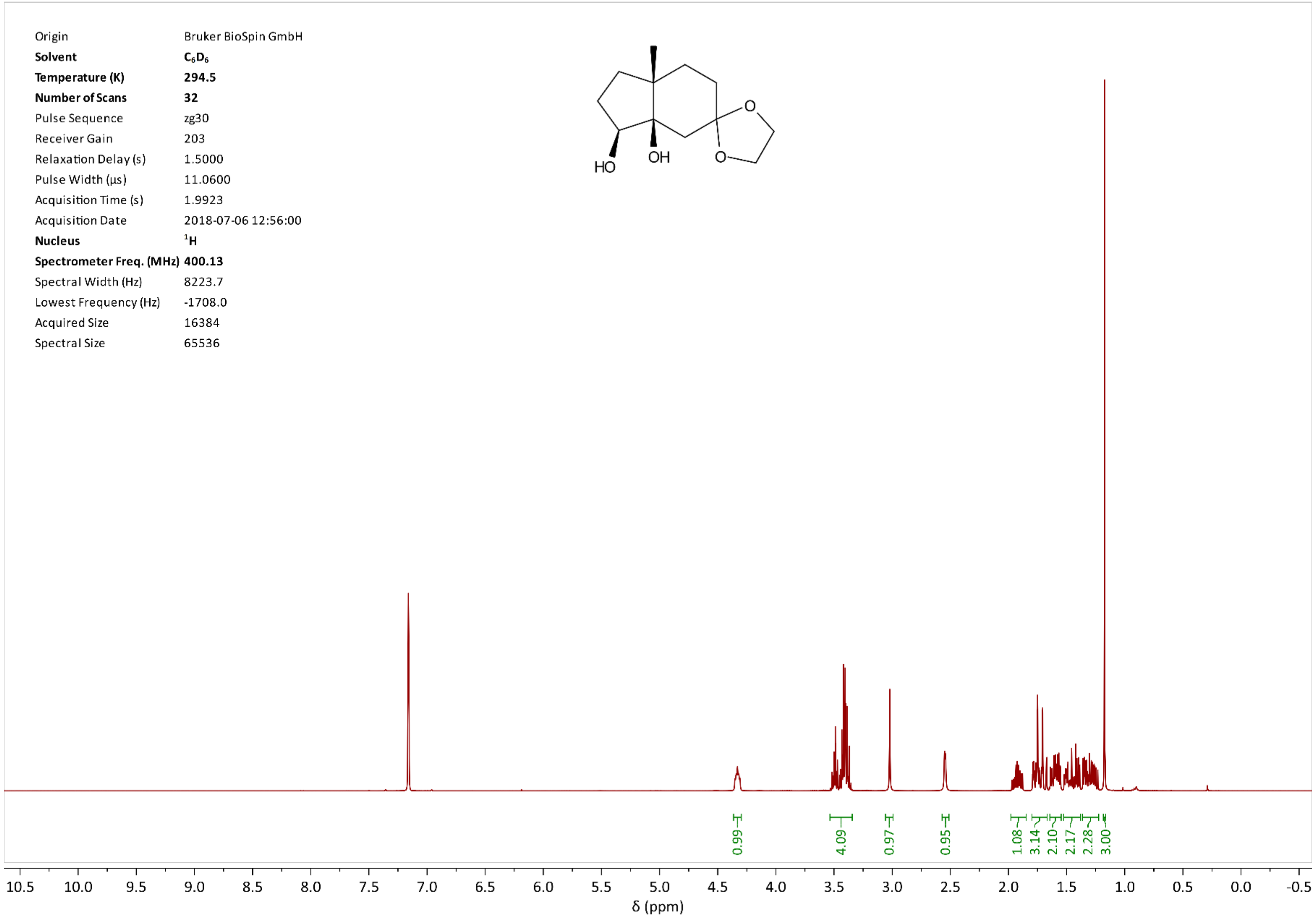

Diol 5

^{13}C NMR Spectrum, C_6D_6, 101 MHz

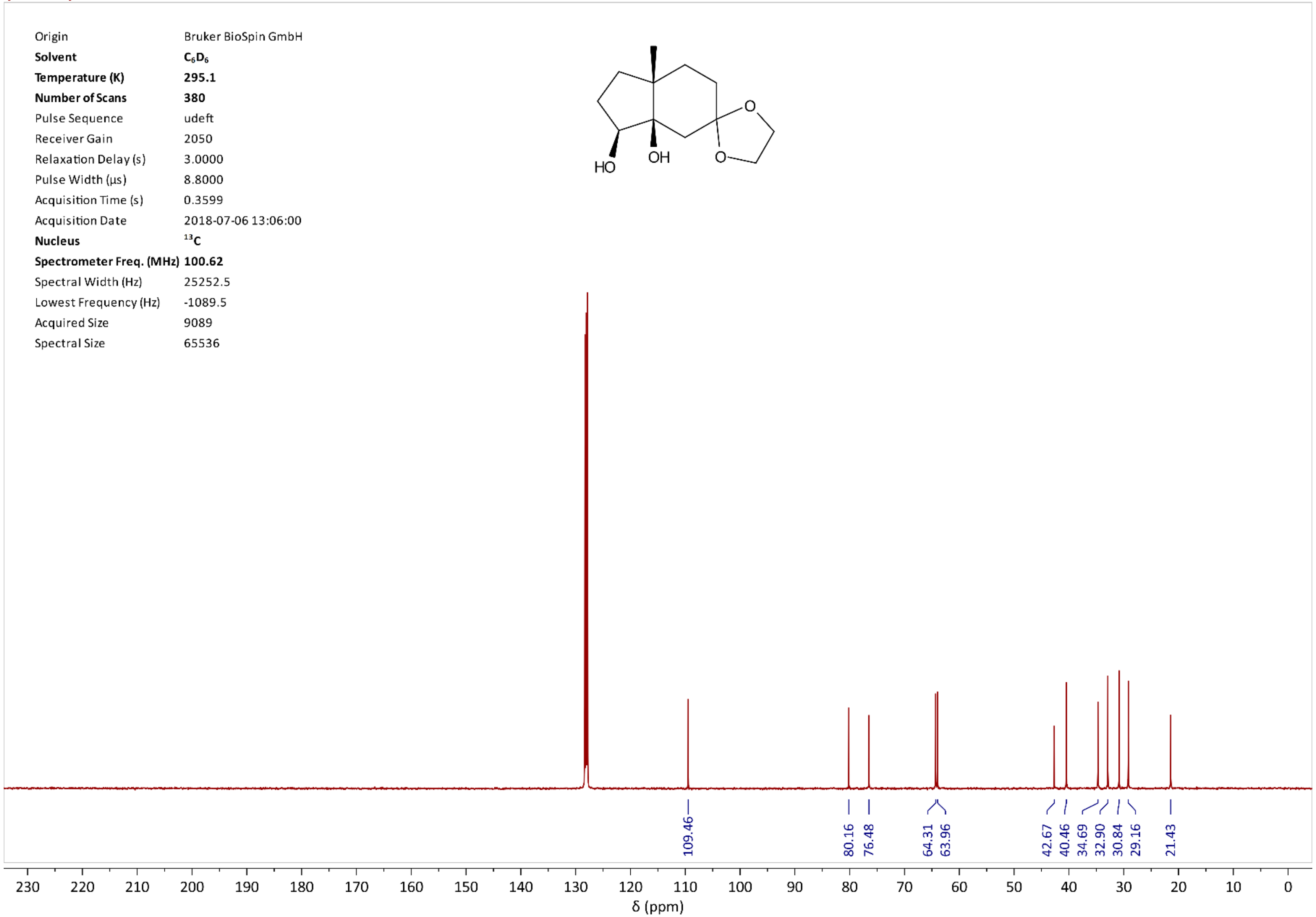

Trans-hydrindane 6

^1H NMR Spectrum, C_6D_6, 400 MHz

Origin	Bruker BioSpin GmbH
**Solvent**	**C_6D_6**
**Temperature (K)**	**297.9**
**Number of Scans**	**2**
Pulse Sequence	zg30
Receiver Gain	60
Relaxation Delay (s)	2.0000
Pulse Width (μs)	10.0000
Acquisition Time (s)	4.5875
Acquisition Date	2019-02-07 23:07:54
**Nucleus**	**^1H**
**Spectrometer Freq. (MHz)**	**400.13**
Spectral Width (Hz)	7142.9
Lowest Frequency (Hz)	-1167.2
Acquired Size	32768
Spectral Size	65536

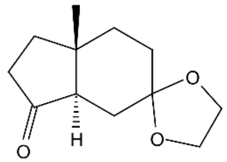

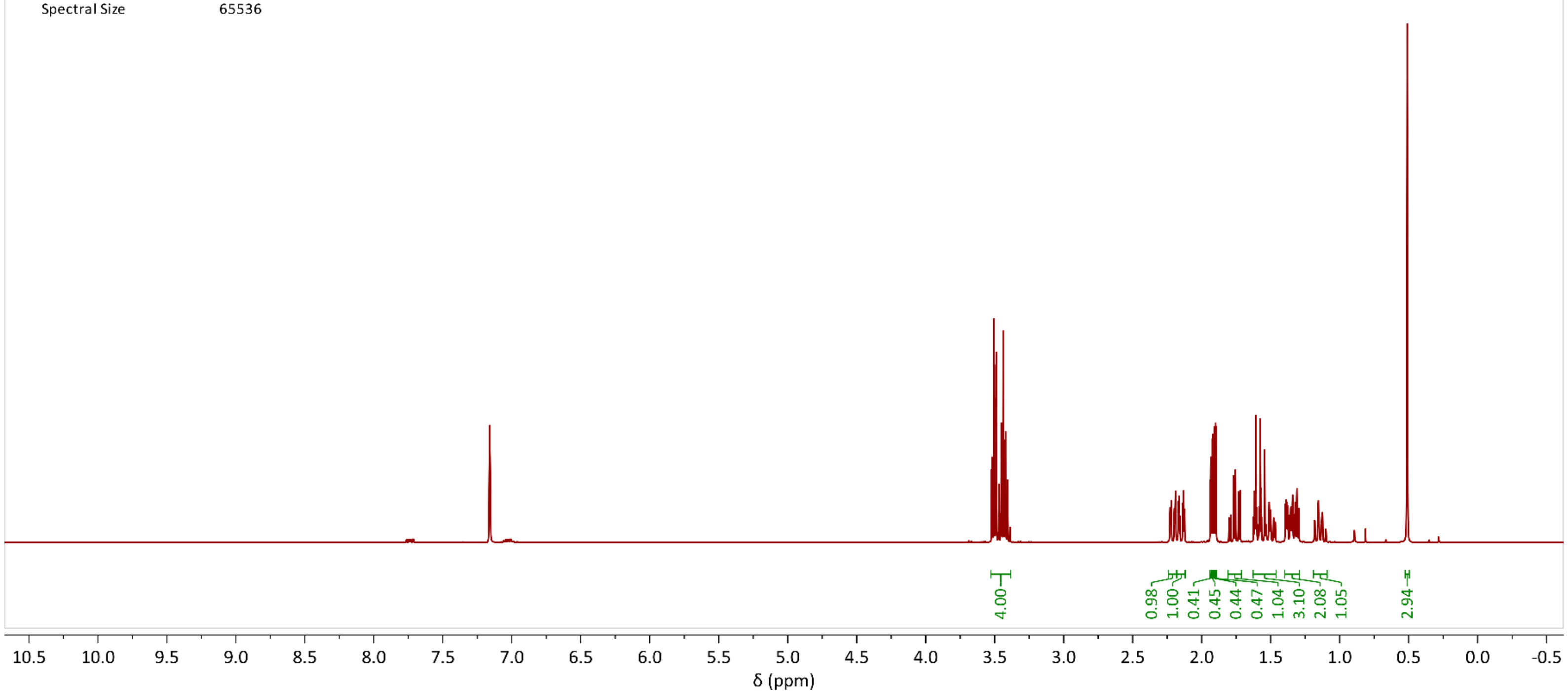

Trans-hydrindane 6

^{13}C NMR Spectrum, C_6D_6, 101 MHz

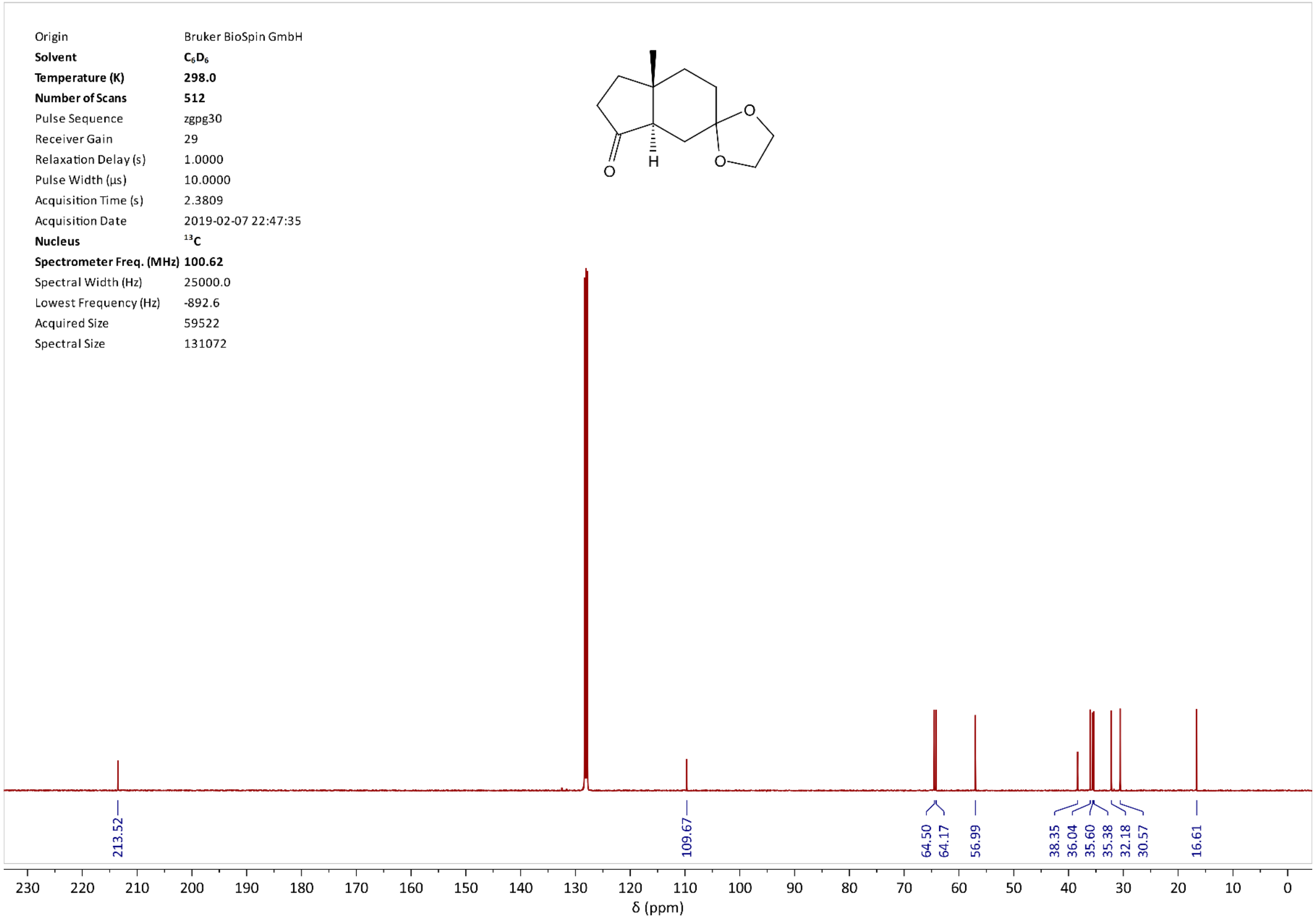

Tertiary alcohol 7

^{1}H NMR Spectrum, C_6D_6, 400 MHz

Origin	Bruker BioSpin GmbH
**Solvent**	**C_6D_6**
**Temperature (K)**	**294.6**
**Number of Scans**	**32**
Pulse Sequence	zg30
Receiver Gain	203
Relaxation Delay (s)	1.5000
Pulse Width (μs)	11.0600
Acquisition Time (s)	1.9923
Acquisition Date	2018-06-30 16:25:00
**Nucleus**	**^{1}H**
**Spectrometer Freq. (MHz)**	**400.13**
Spectral Width (Hz)	8223.7
Lowest Frequency (Hz)	-1740.0
Acquired Size	16384
Spectral Size	65536

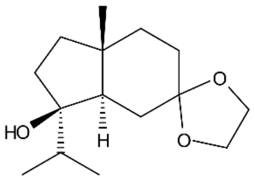

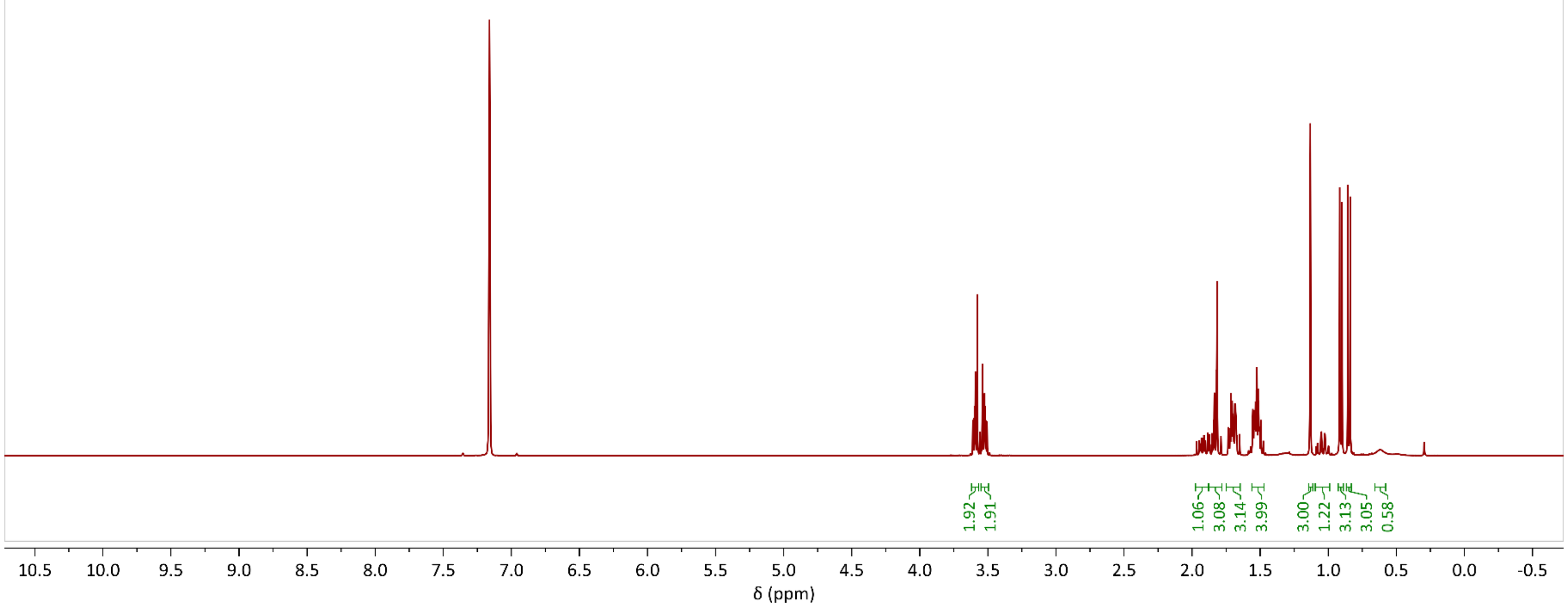

Tertiary alcohol 7

^{13}C NMR Spectrum, C_6D_6, 101 MHz

Origin	Bruker BioSpin GmbH
**Solvent**	**C_6D_6**
**Temperature (K)**	**294.6**
**Number of Scans**	**380**
Pulse Sequence	udeft
Receiver Gain	2050
Relaxation Delay (s)	3.0000
Pulse Width (µs)	8.8000
Acquisition Time (s)	0.3599
Acquisition Date	2018-06-30 16:35:00
**Nucleus**	**^{13}C**
**Spectrometer Freq. (MHz)**	**100.62**
Spectral Width (Hz)	25252.5
Lowest Frequency (Hz)	-1097.5
Acquired Size	9089
Spectral Size	65536

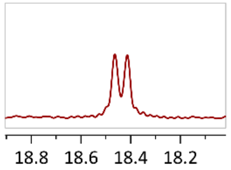

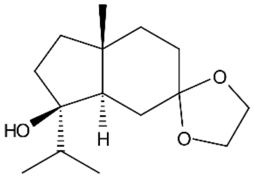

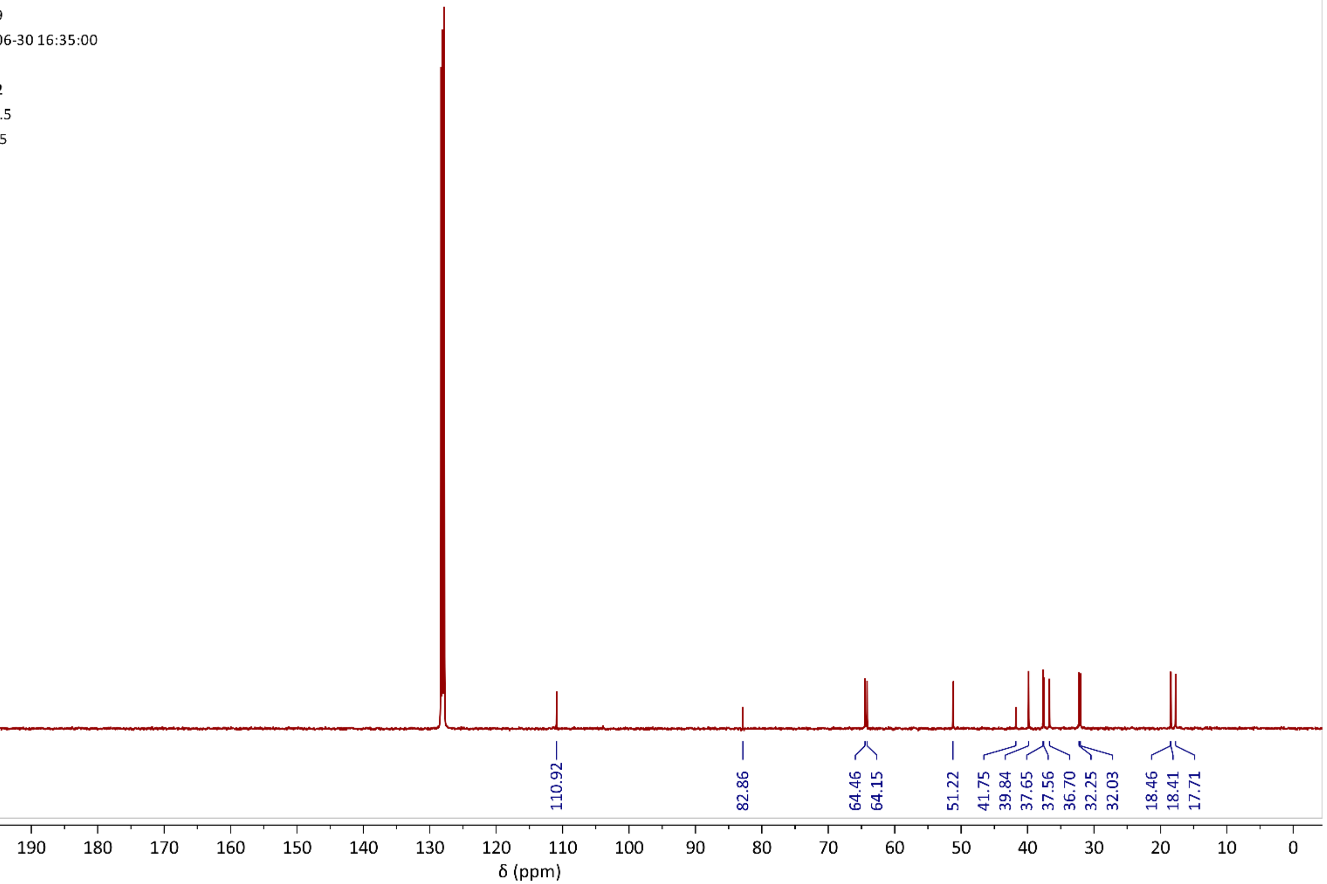

Hajos-Parrish ketone 15

^{1}H NMR Spectrum, $CDCl_3$, 400 MHz

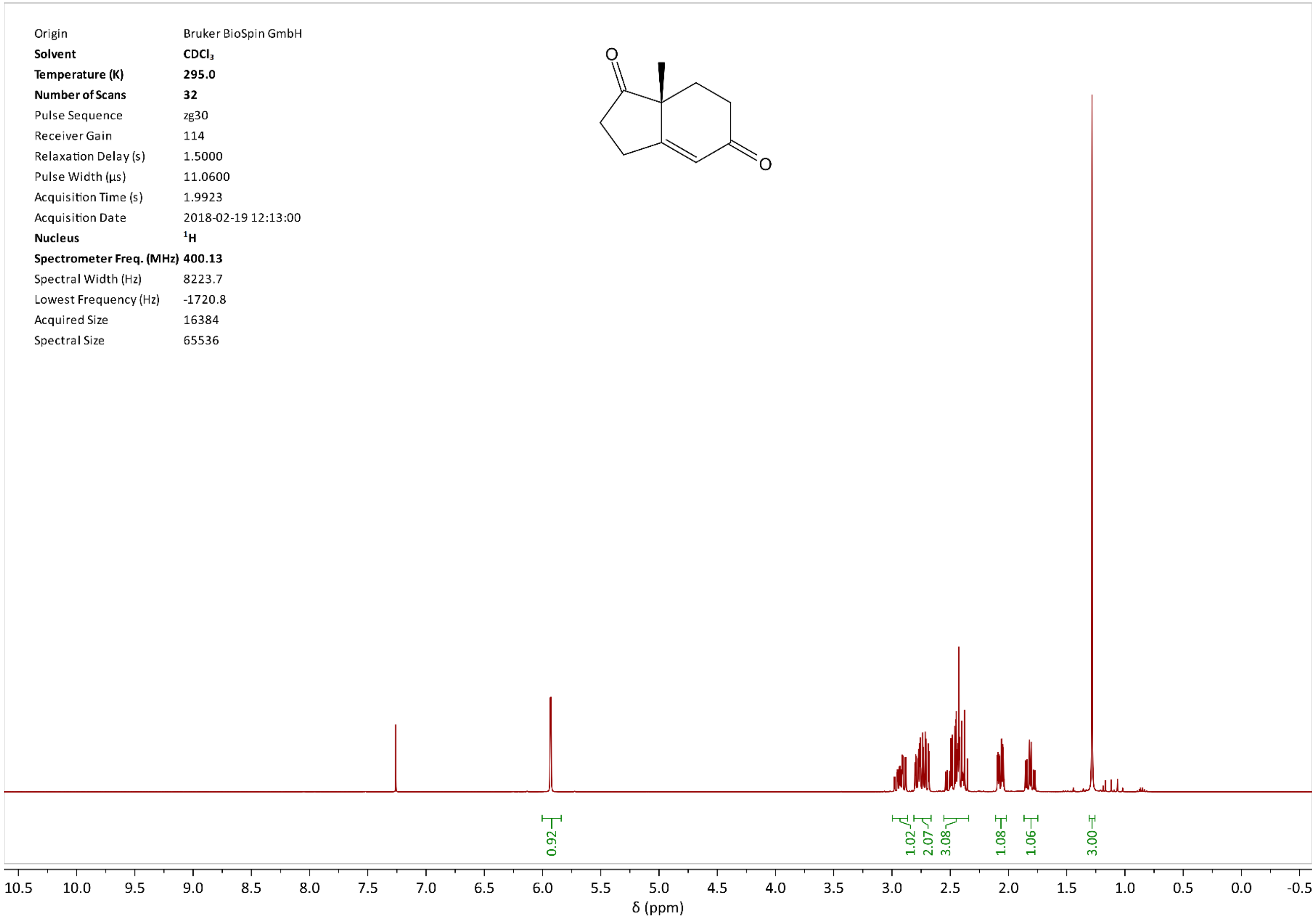

Hajos-Parrish ketone 15

^{13}C NMR Spectrum, $CDCl_3$, 101 MHz

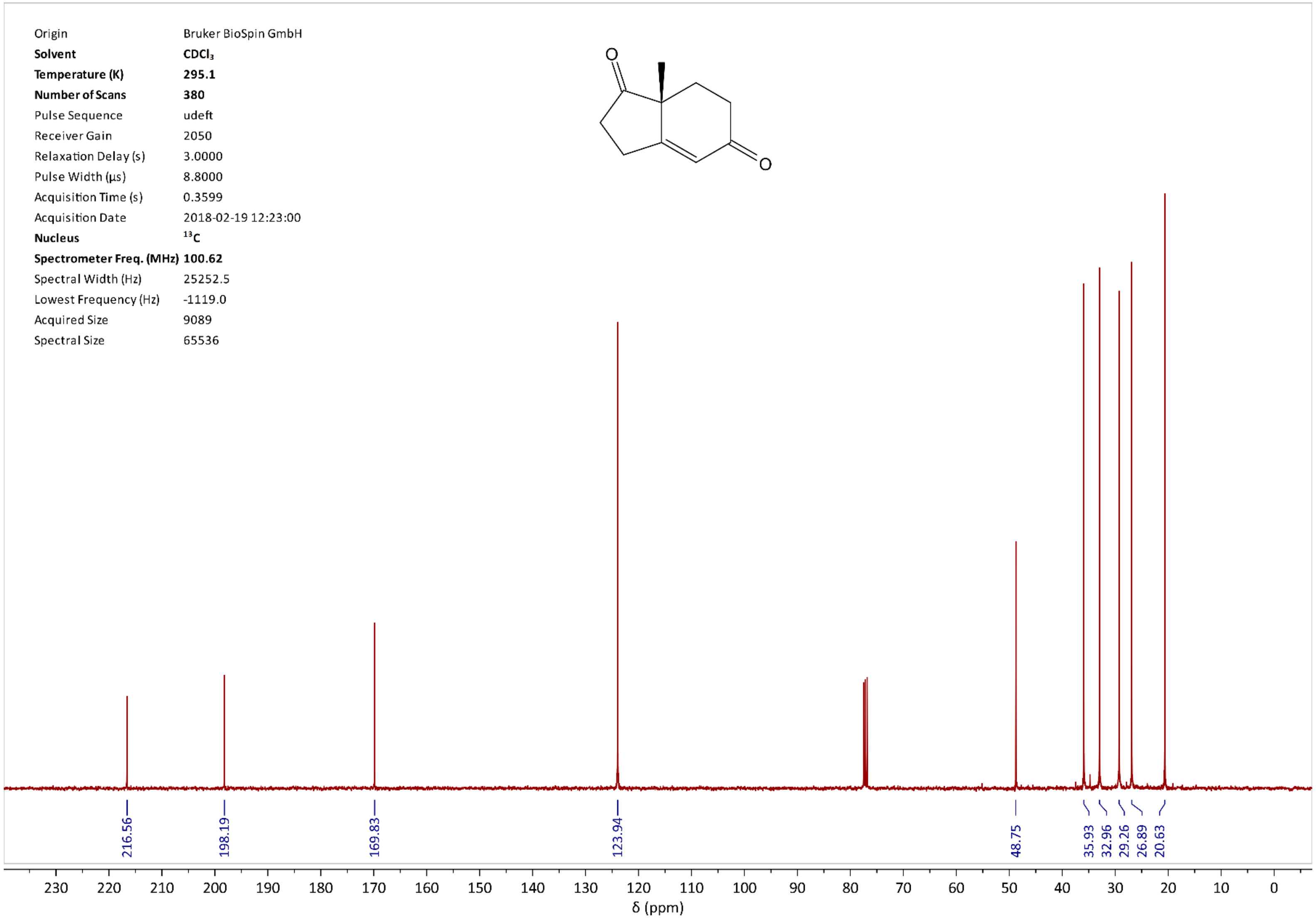

Alcohol 16

^{1}H NMR Spectrum, $CDCl_3$, 400 MHz

Origin	Bruker BioSpin GmbH
**Solvent**	**$CDCl_3$**
**Temperature (K)**	**295.3**
**Number of Scans**	**32**
Pulse Sequence	zg30
Receiver Gain	114
Relaxation Delay (s)	1.5000
Pulse Width (μs)	11.0600
Acquisition Time (s)	1.9923
Acquisition Date	2018-02-20 02:56:00
**Nucleus**	**^{1}H**
**Spectrometer Freq. (MHz)**	**400.13**
Spectral Width (Hz)	8223.7
Lowest Frequency (Hz)	-1720.9
Acquired Size	16384
Spectral Size	65536

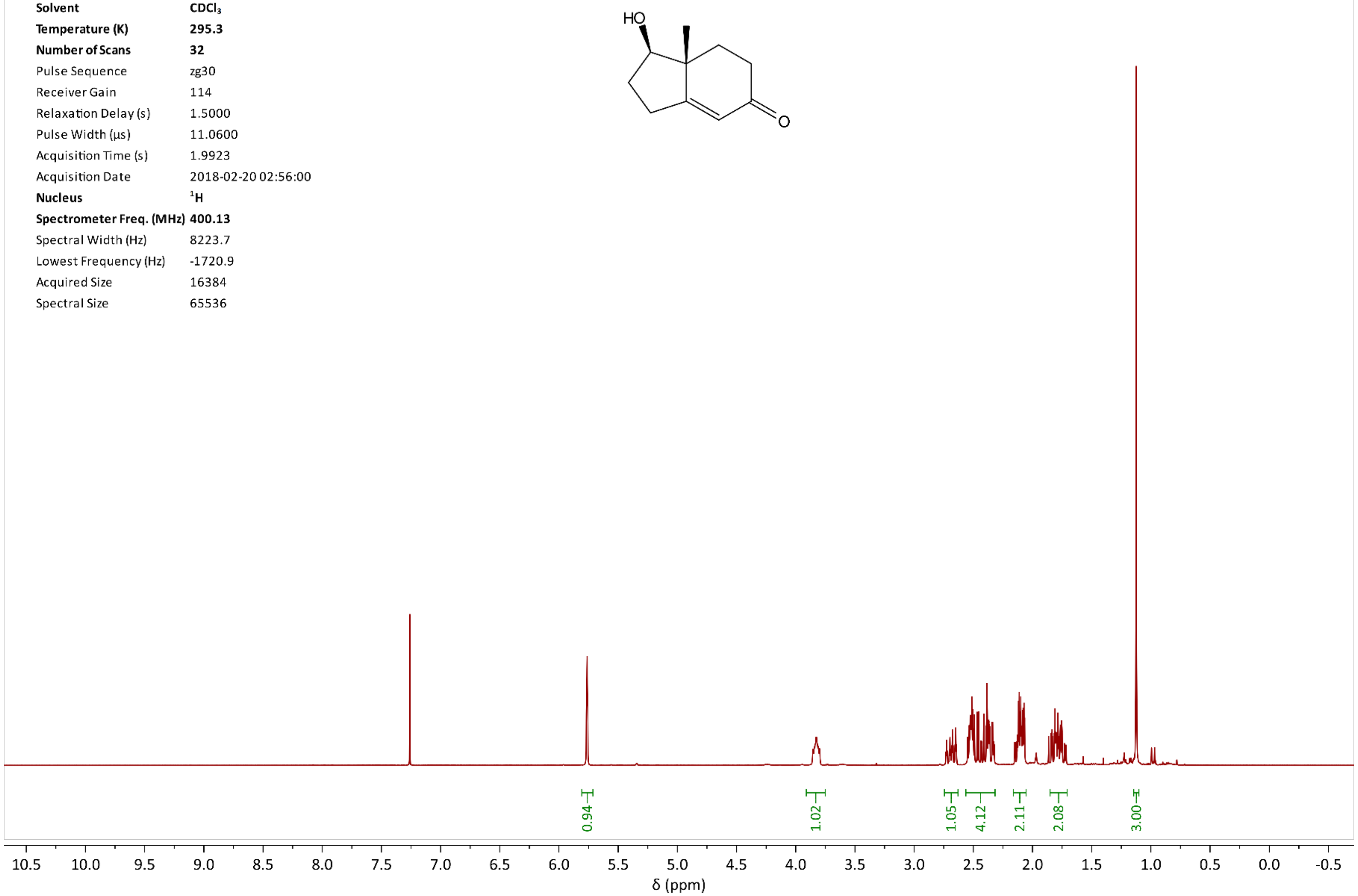

Alcohol 16

^{13}C NMR Spectrum, $CDCl_3$, 101 MHz

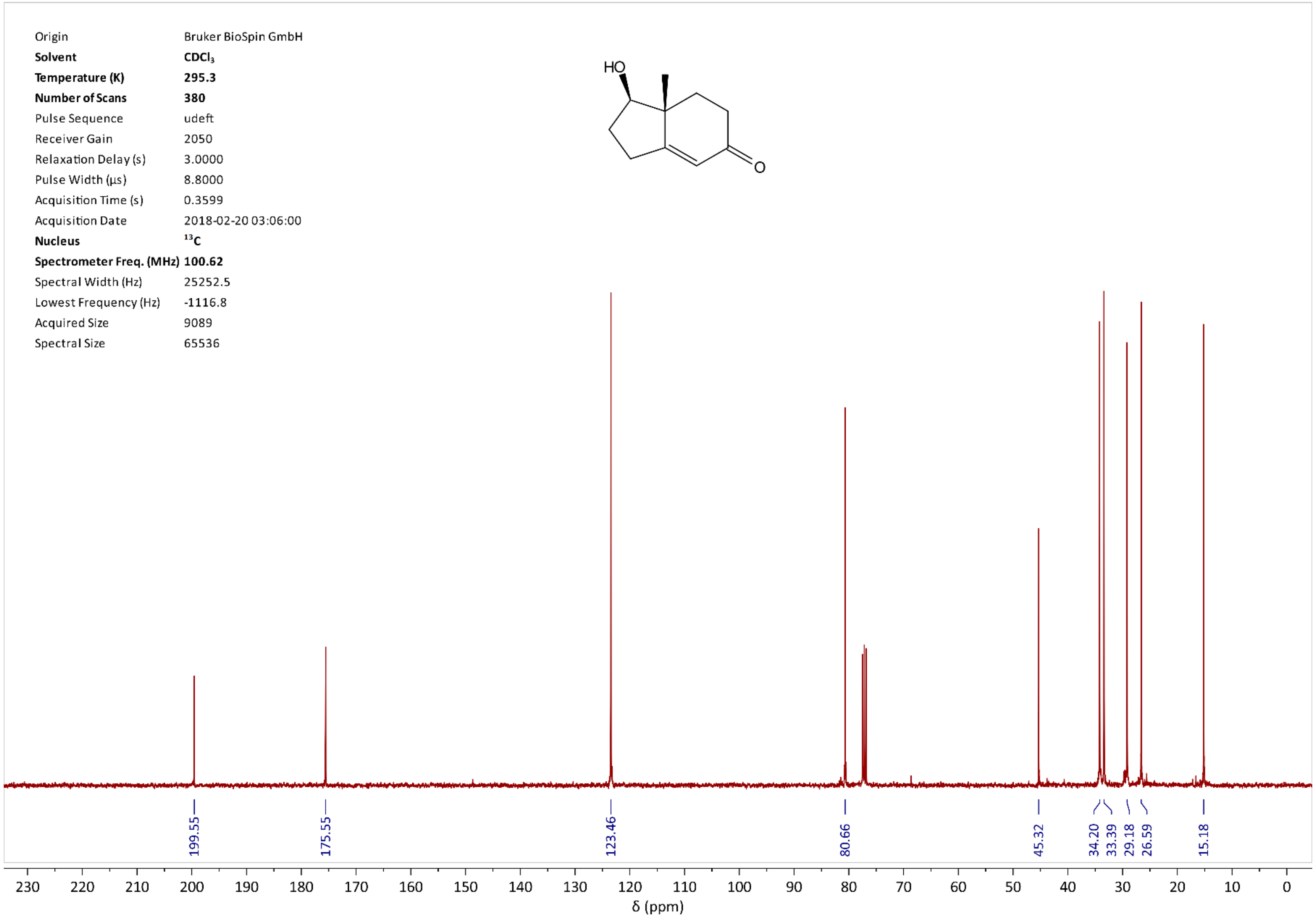

Thionocarbamate 17

^{1}H NMR Spectrum, $CDCl_3$, 400 MHz

Origin	Bruker BioSpin GmbH
**Solvent**	**$CDCl_3$**
**Temperature (K)**	**295.0**
**Number of Scans**	**32**
Pulse Sequence	zg30
Receiver Gain	128
Relaxation Delay (s)	1.5000
Pulse Width (µs)	11.0600
Acquisition Time (s)	1.9923
Acquisition Date	2018-02-16 17:21:00
**Nucleus**	**^{1}H**
**Spectrometer Freq. (MHz)**	**400.13**
Spectral Width (Hz)	8223.7
Lowest Frequency (Hz)	-1721.1
Acquired Size	16384
Spectral Size	65536

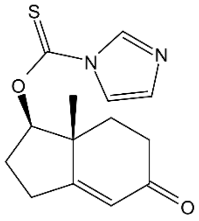

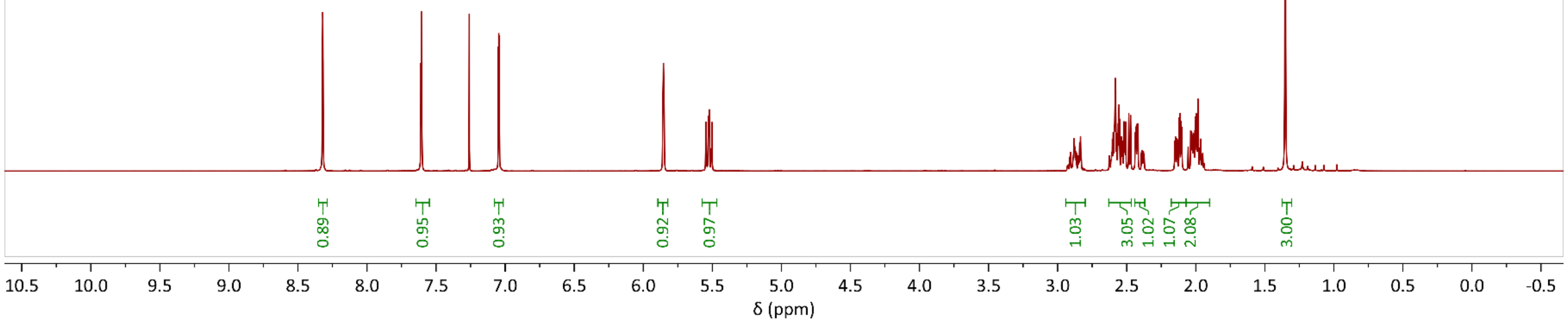

Thionocarbamate 17

^{13}C NMR Spectrum, $CDCl_3$, 101 MHz

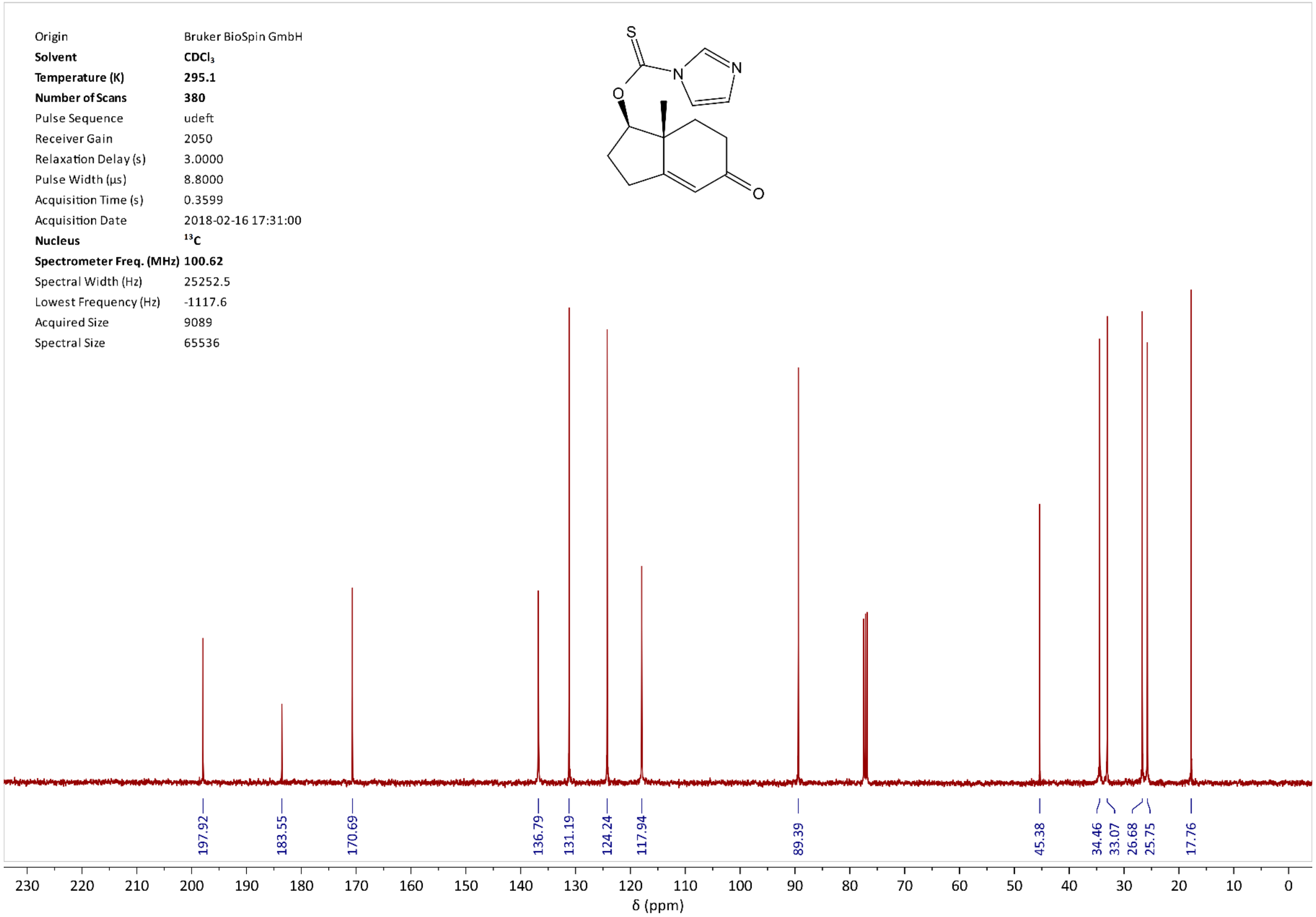

Thionocarbonate acetal 18

^{1}H NMR Spectrum, $CDCl_3$, 400 MHz

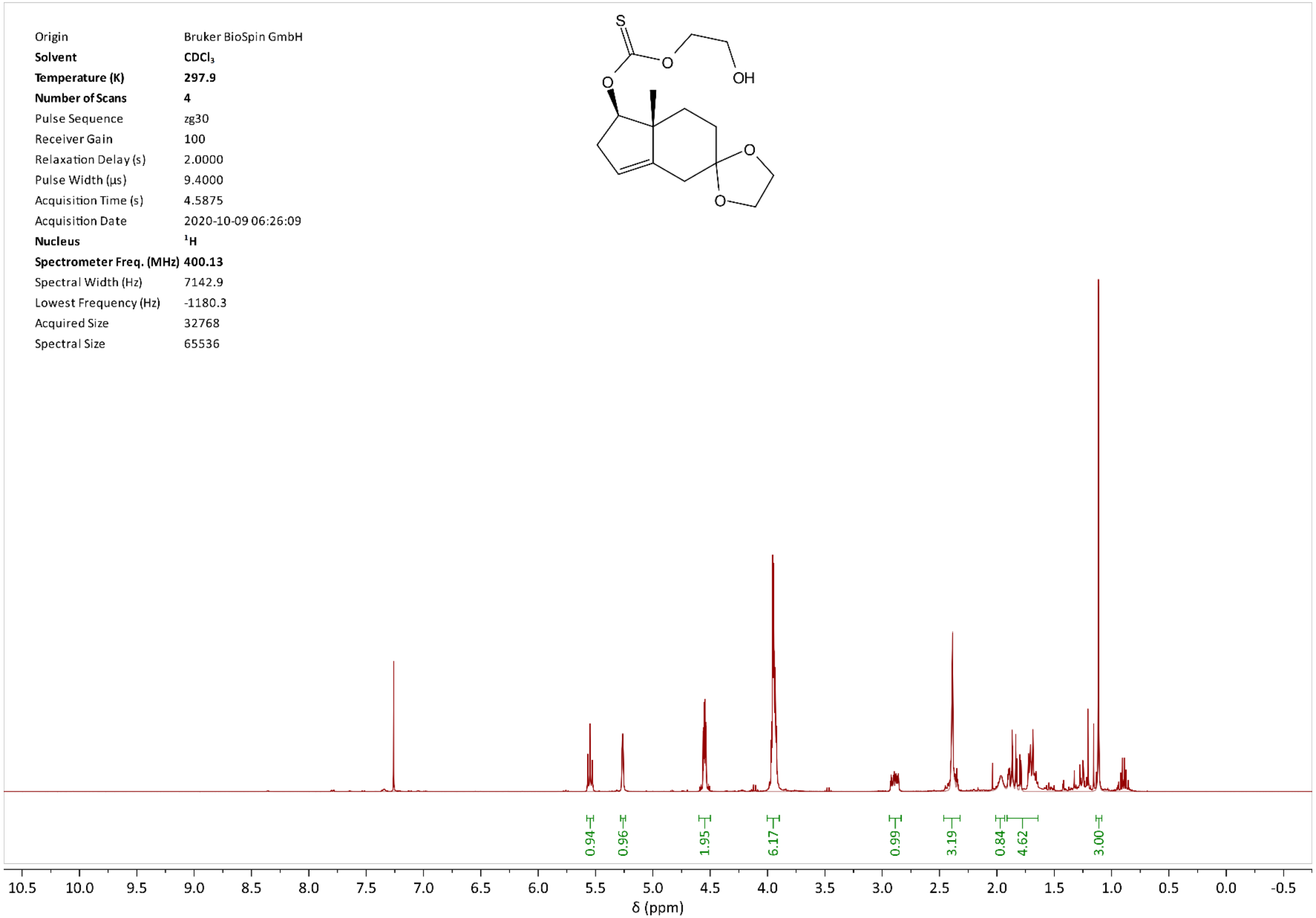

Thionocarbonate acetal 19

^{13}C NMR Spectrum, $CDCl_3$, 101 MHz

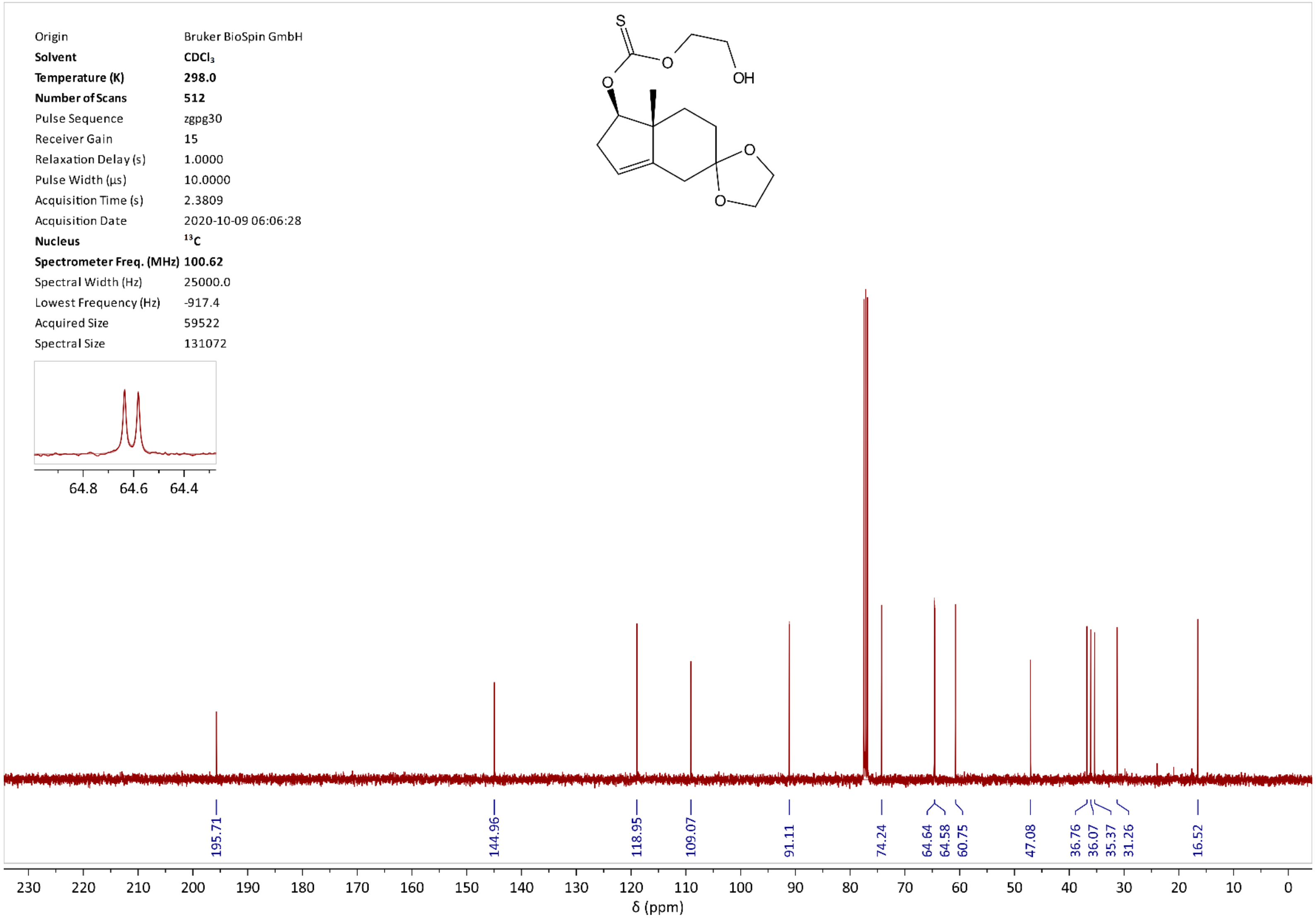

Xanthate 19

^{1}H NMR Spectrum, $CDCl_3$, 400 MHz

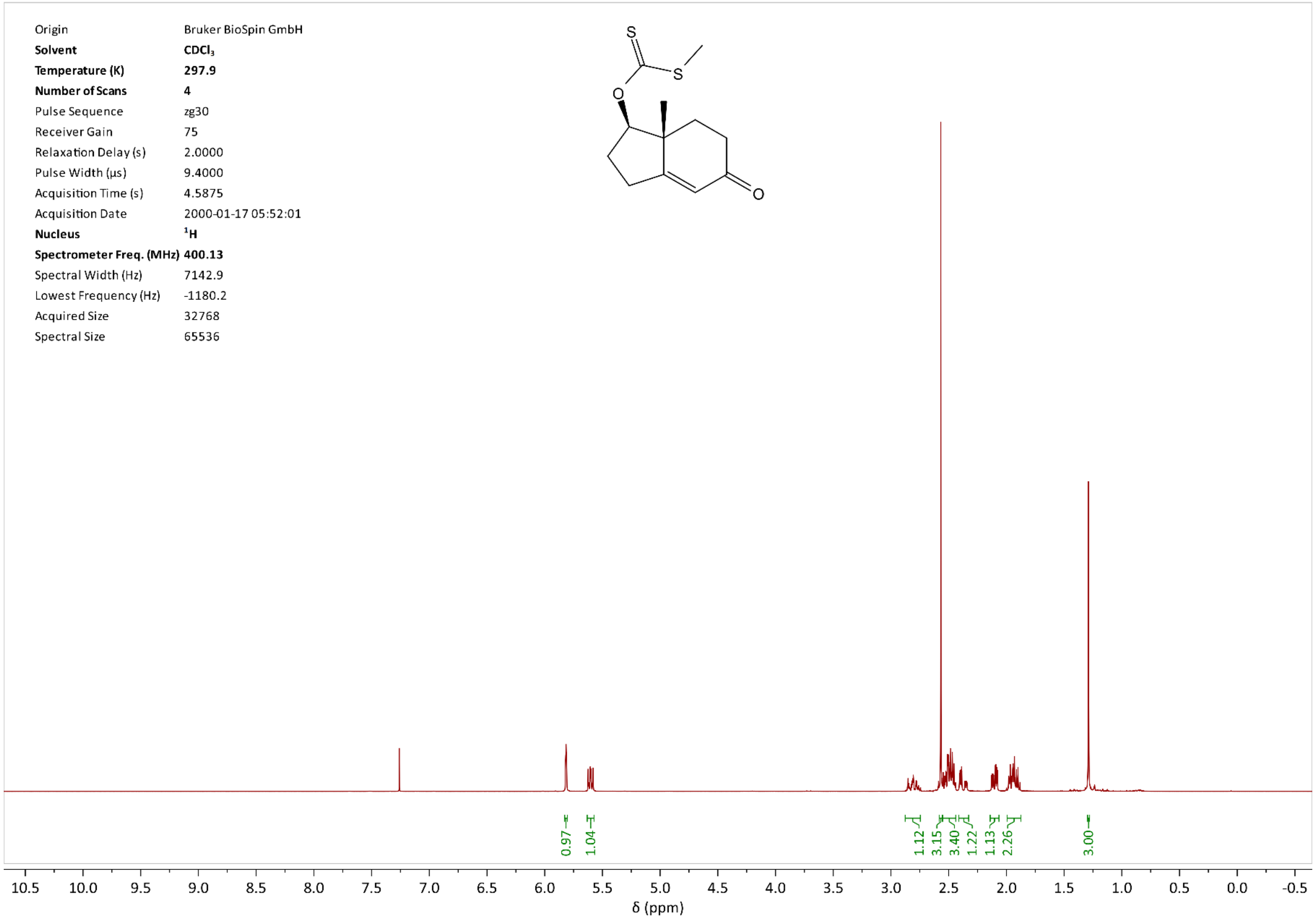

Xanthate 19

^{13}C NMR Spectrum, $CDCl_3$, 101 MHz

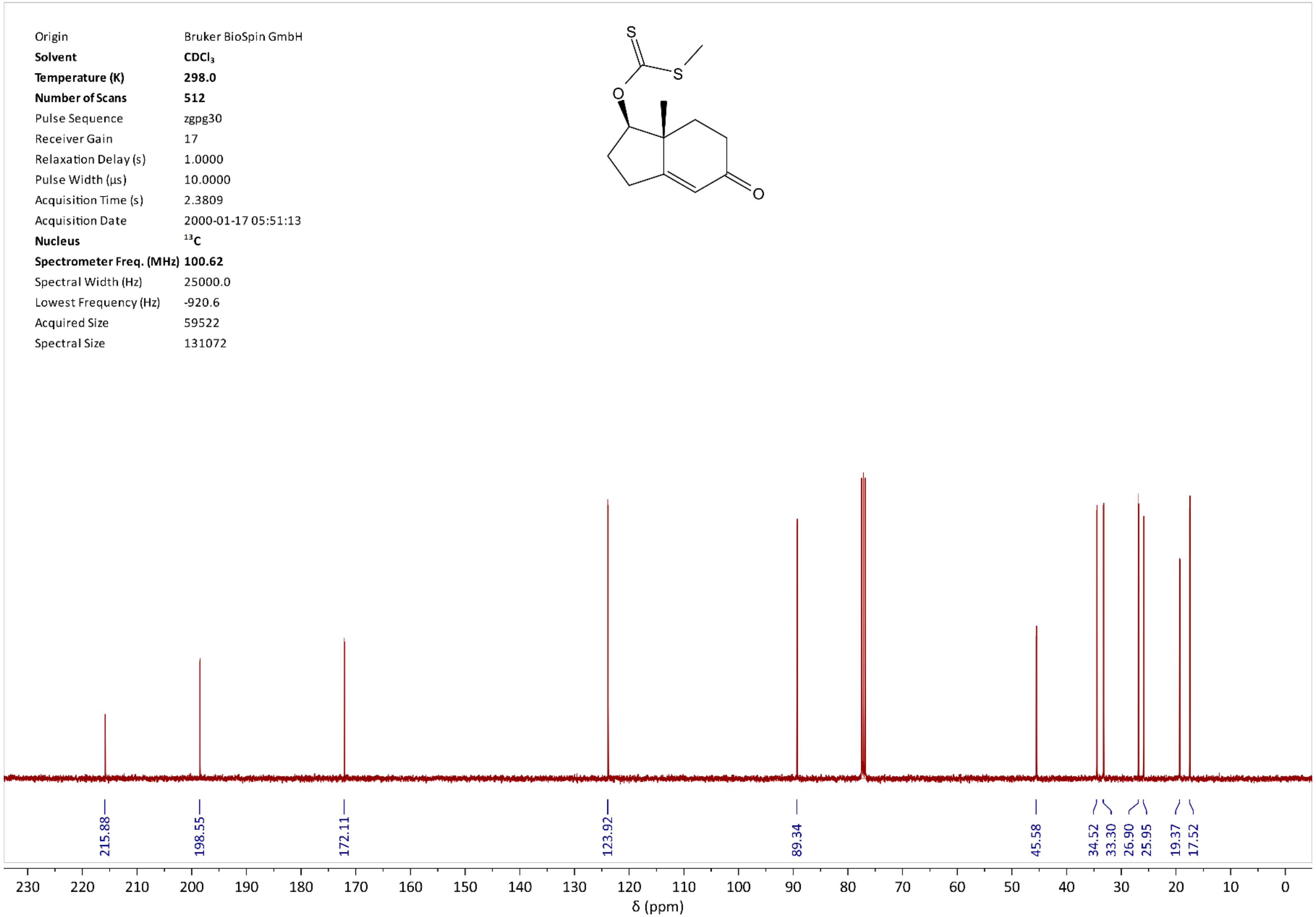

Xanthate acetal 20

^{1}H NMR Spectrum, $CDCl_3$, 400 MHz

Origin	Bruker BioSpin GmbH
**Solvent**	**$CDCl_3$**
**Temperature (K)**	**297.9**
**Number of Scans**	**4**
Pulse Sequence	zg30
Receiver Gain	101
Relaxation Delay (s)	2.0000
Pulse Width (µs)	9.4000
Acquisition Time (s)	4.5875
Acquisition Date	2000-01-22 13:31:53
**Nucleus**	**^1H**
**Spectrometer Freq. (MHz)**	**400.13**
Spectral Width (Hz)	7142.9
Lowest Frequency (Hz)	-1180.3
Acquired Size	32768
Spectral Size	65536

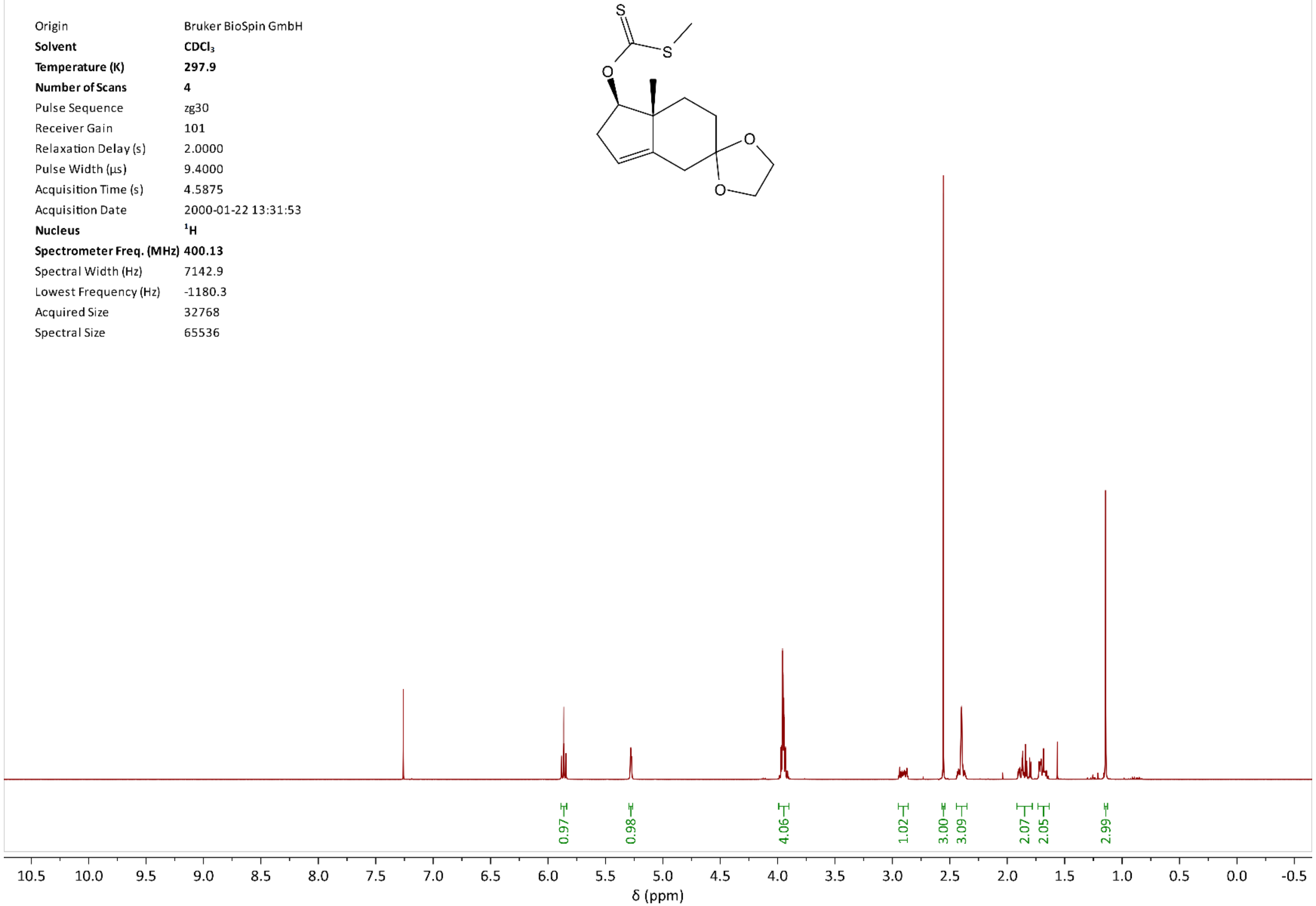

Xanthate acetal 20

^{13}C NMR Spectrum, $CDCl_3$, 101 MHz

Origin	Bruker BioSpin GmbH
**Solvent**	**$CDCl_3$**
**Temperature (K)**	**298.0**
**Number of Scans**	**512**
Pulse Sequence	zgpg30
Receiver Gain	16
Relaxation Delay (s)	1.0000
Pulse Width (μs)	10.0000
Acquisition Time (s)	2.3809
Acquisition Date	2000-01-22 13:31:04
**Nucleus**	**^{13}C**
**Spectrometer Freq. (MHz)**	**100.62**
Spectral Width (Hz)	25000.0
Lowest Frequency (Hz)	-917.1
Acquired Size	59522
Spectral Size	131072

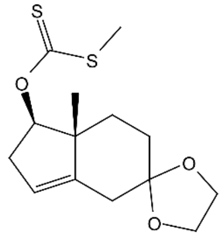

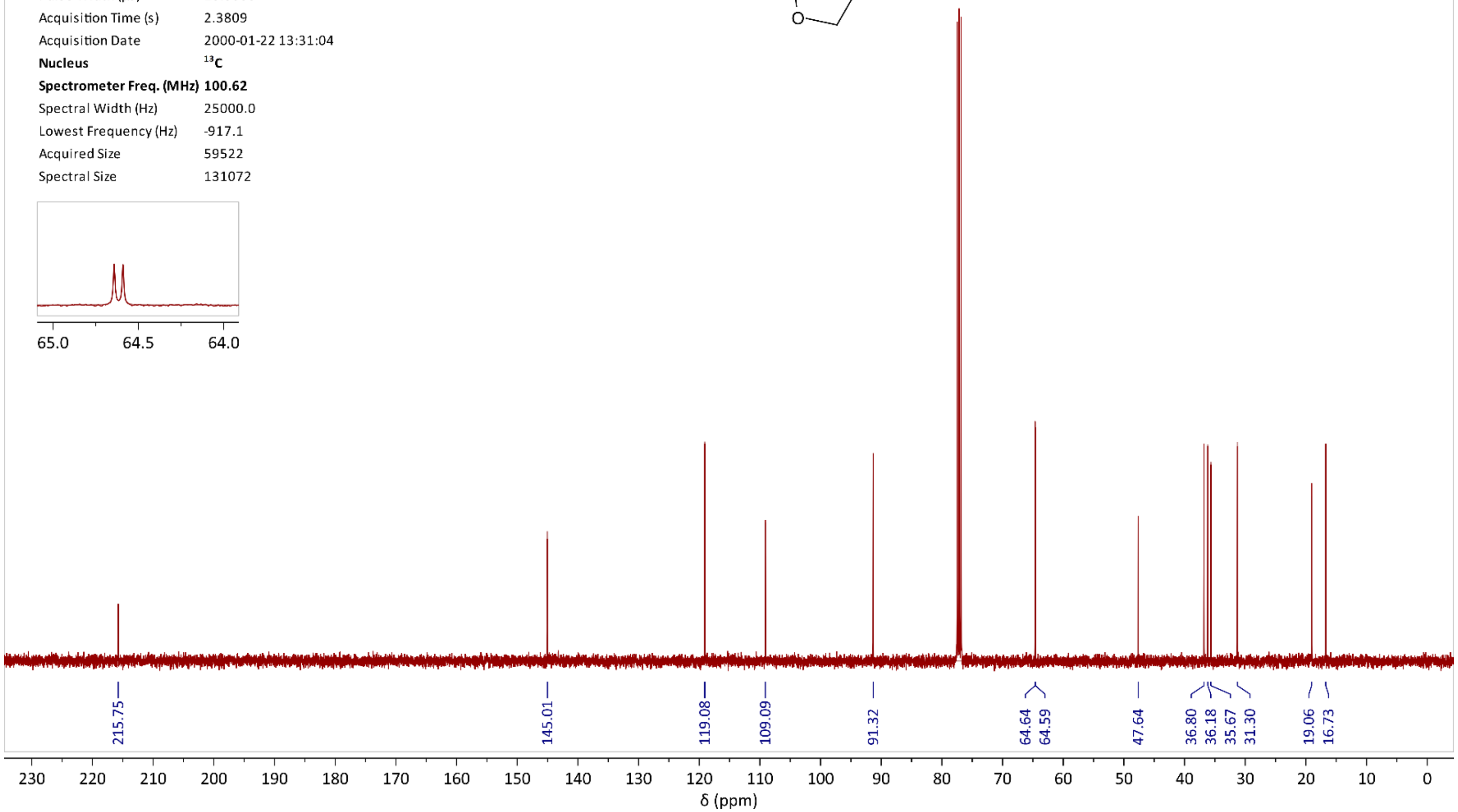

O-Phenyl thionocarbonate 21
^{1}H NMR Spectrum, $CDCl_3$, 400 MHz

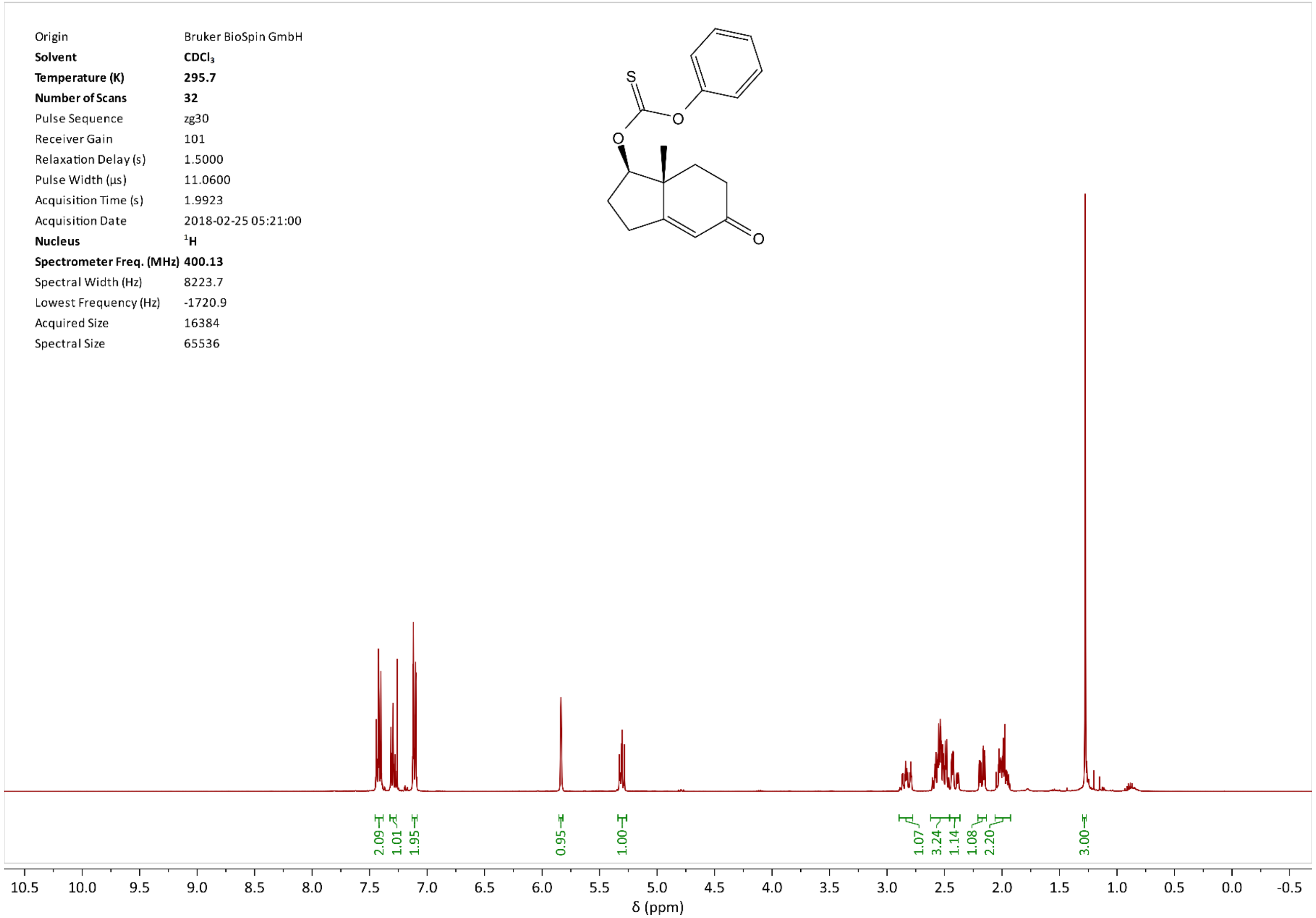

O-Phenyl thionocarbonate 21

^{13}C NMR Spectrum, $CDCl_3$, 101 MHz

Origin	Bruker BioSpin GmbH
**Solvent**	**$CDCl_3$**
**Temperature (K)**	**295.7**
**Number of Scans**	**380**
Pulse Sequence	udeft
Receiver Gain	2050
Relaxation Delay (s)	3.0000
Pulse Width (µs)	8.8000
Acquisition Time (s)	0.3599
Acquisition Date	2018-02-25 05:31:00
**Nucleus**	**^{13}C**
**Spectrometer Freq. (MHz)**	**100.62**
Spectral Width (Hz)	25252.5
Lowest Frequency (Hz)	-1119.7
Acquired Size	9089
Spectral Size	65536

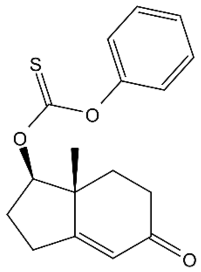

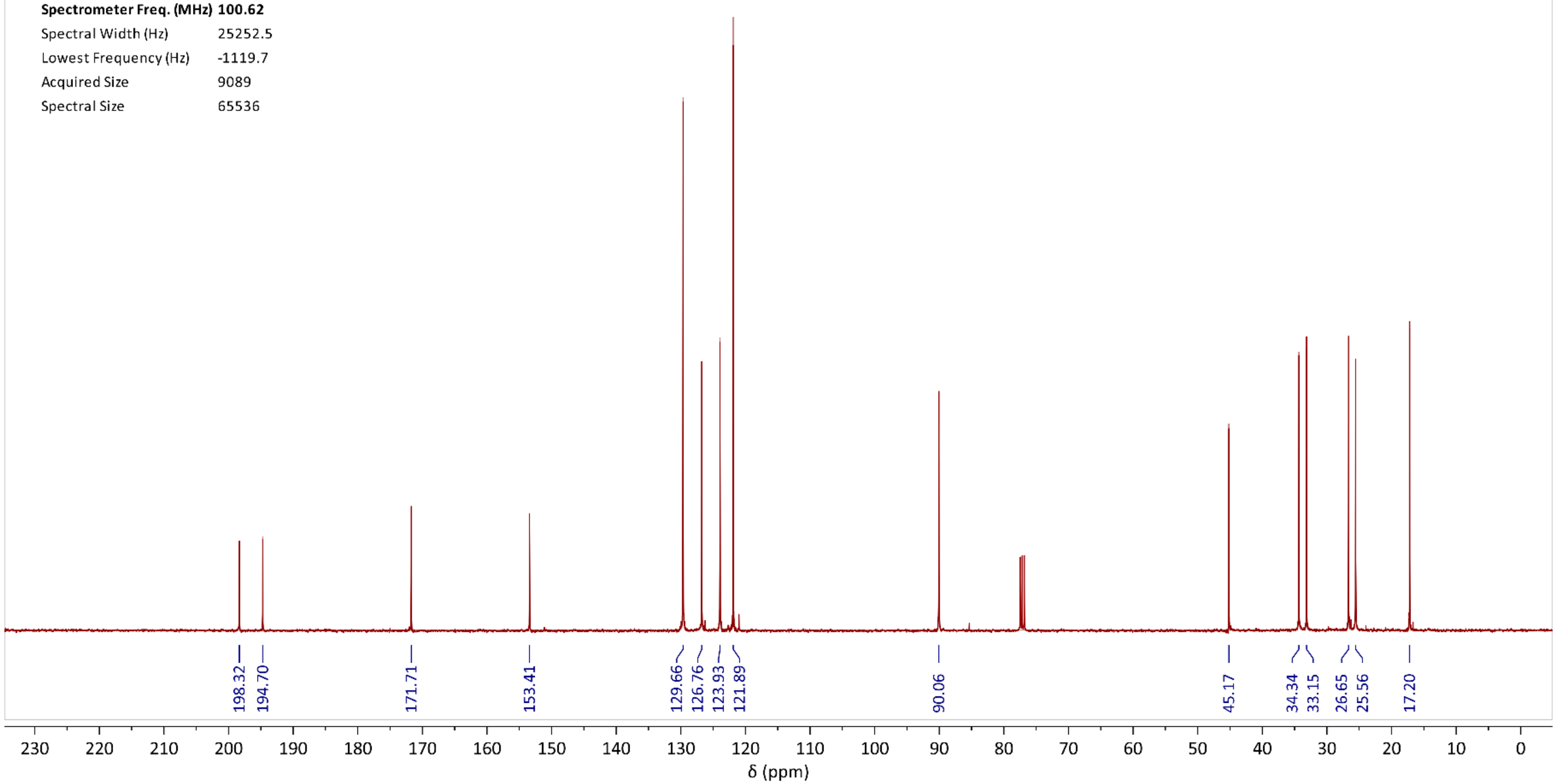

Thionocarbonate acetal 22

^{1}H NMR Spectrum, $CDCl_3$, 400 MHz

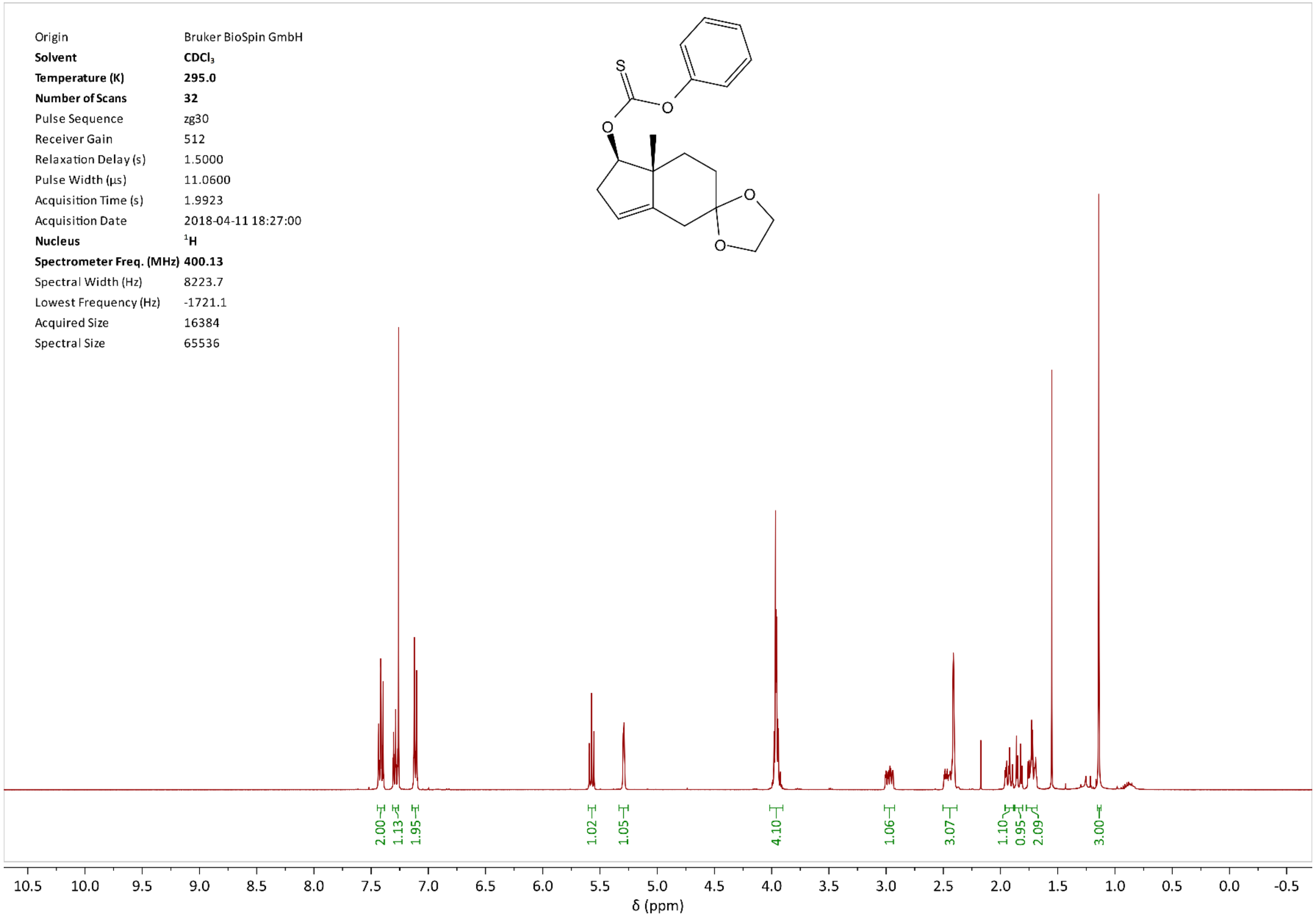

Thionocarbonate acetal 22

^{13}C NMR Spectrum, $CDCl_3$, 101 MHz

Origin	Bruker BioSpin GmbH
**Solvent**	**$CDCl_3$**
**Temperature (K)**	**295.2**
**Number of Scans**	**380**
Pulse Sequence	udeft
Receiver Gain	2050
Relaxation Delay (s)	3.0000
Pulse Width (µs)	8.8000
Acquisition Time (s)	0.3599
Acquisition Date	2018-04-11 18:38:00
**Nucleus**	**^{13}C**
**Spectrometer Freq. (MHz)**	**100.62**
Spectral Width (Hz)	25252.5
Lowest Frequency (Hz)	-1112.0
Acquired Size	9089
Spectral Size	65536

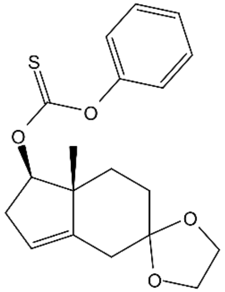

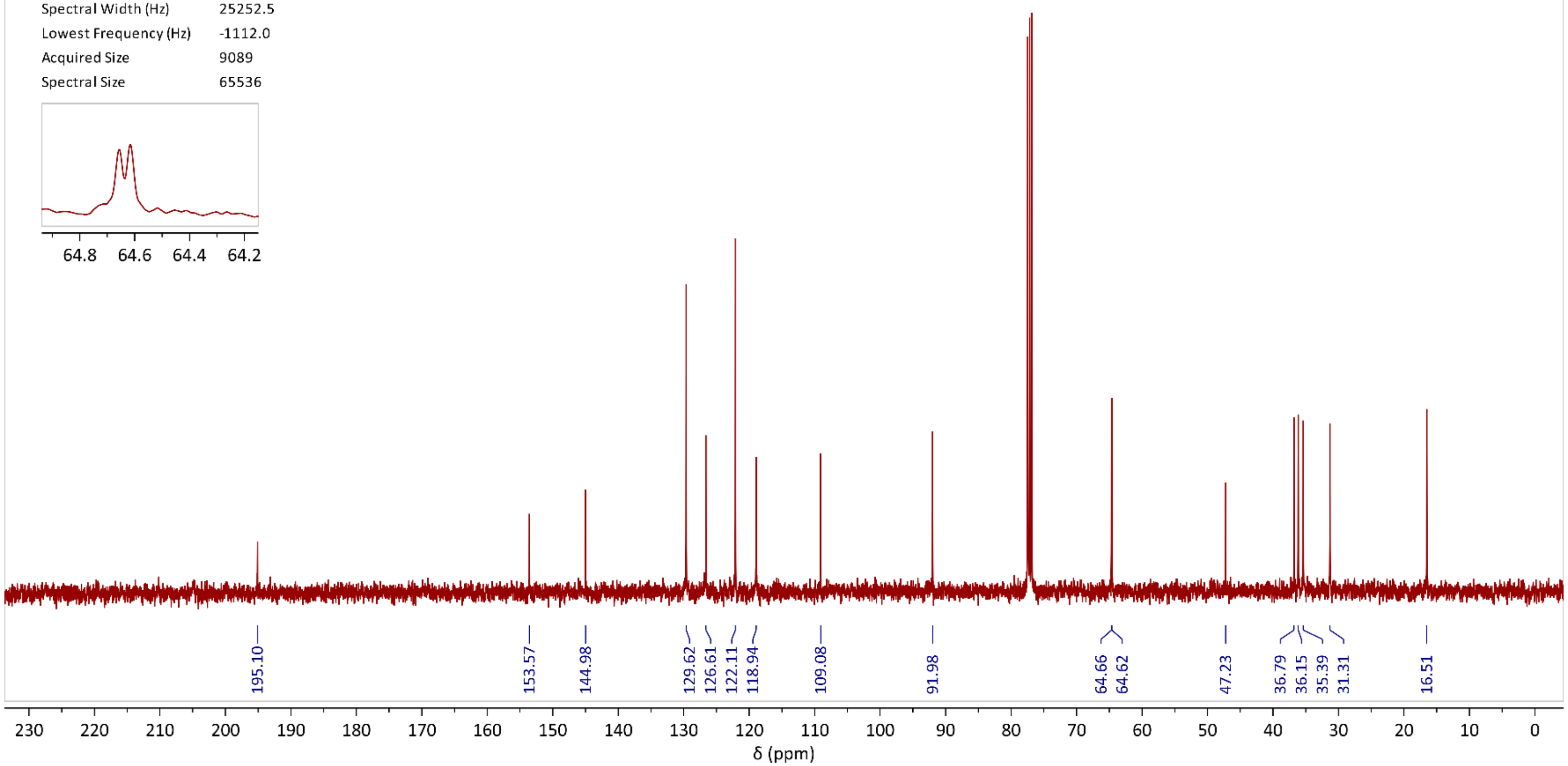

Acetal 23

^{1}H NMR Spectrum, $CDCl_3$, 400 MHz

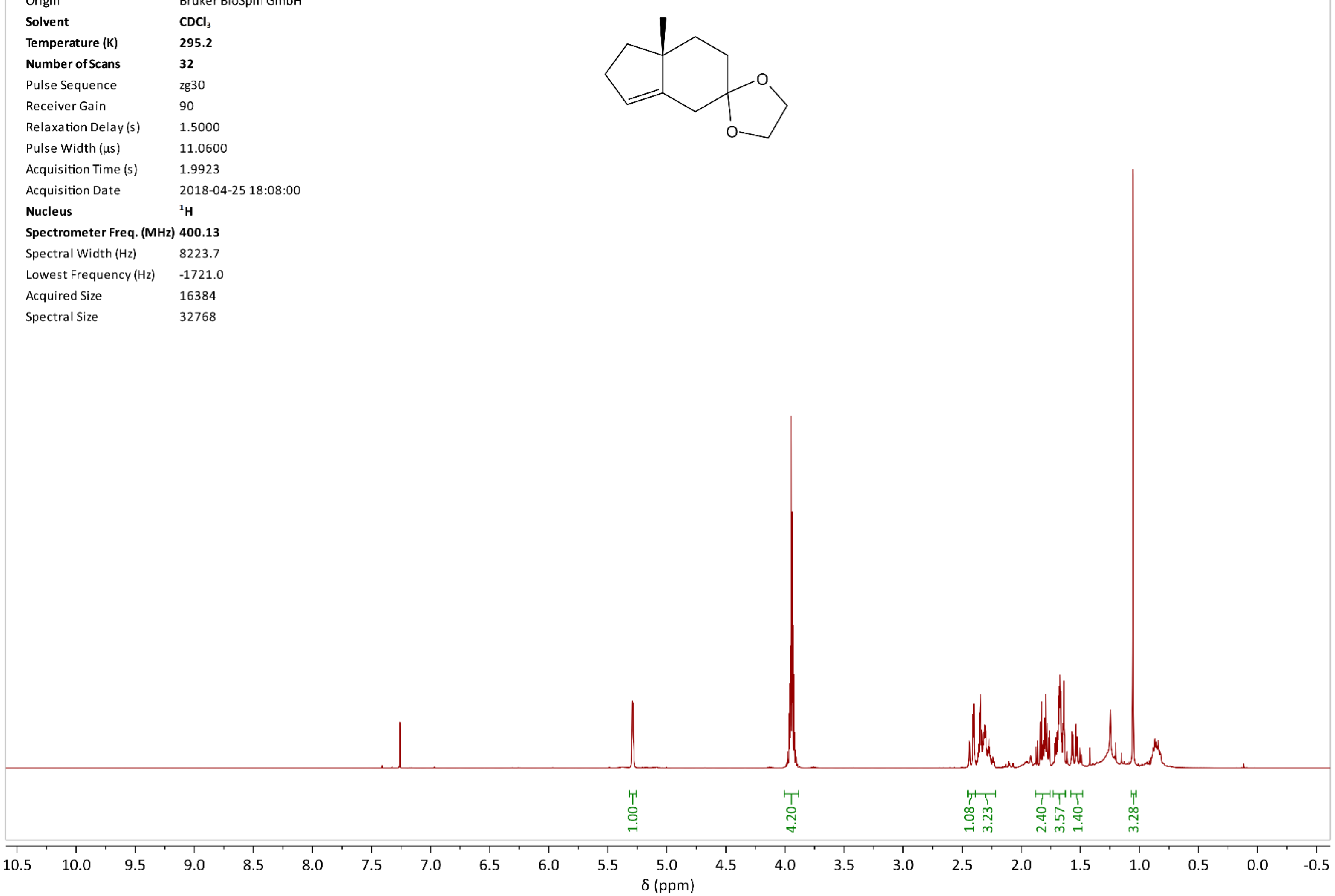

Acetal 23

^{13}C NMR Spectrum, $CDCl_3$, 101 MHz

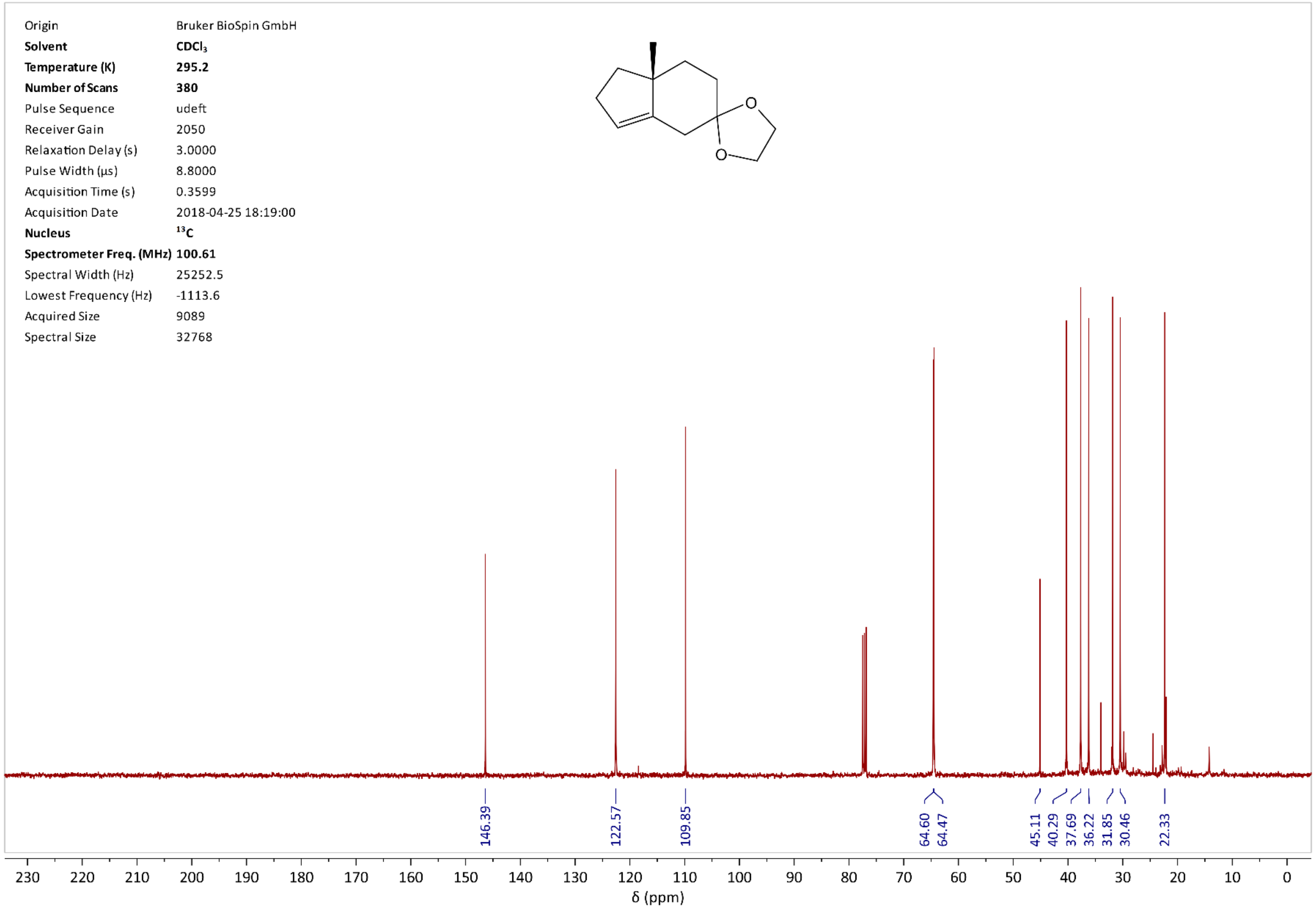

Benzyl hydrindanone 24

^{1}H NMR Spectrum, $CDCl_3$, 400 MHz

Origin	Bruker BioSpin GmbH
**Solvent**	**$CDCl_3$**
**Temperature (K)**	**294.3**
**Number of Scans**	**32**
Pulse Sequence	zg30
Receiver Gain	203
Relaxation Delay (s)	1.5000
Pulse Width (μs)	11.0600
Acquisition Time (s)	1.9923
Acquisition Date	2018-08-20 10:48:00
**Nucleus**	**^{1}H**
**Spectrometer Freq. (MHz)**	**400.13**
Spectral Width (Hz)	8223.7
Lowest Frequency (Hz)	-1721.0
Acquired Size	16384
Spectral Size	65536

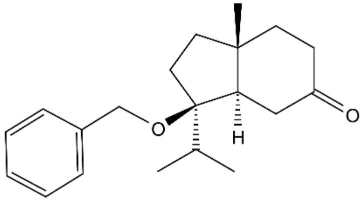

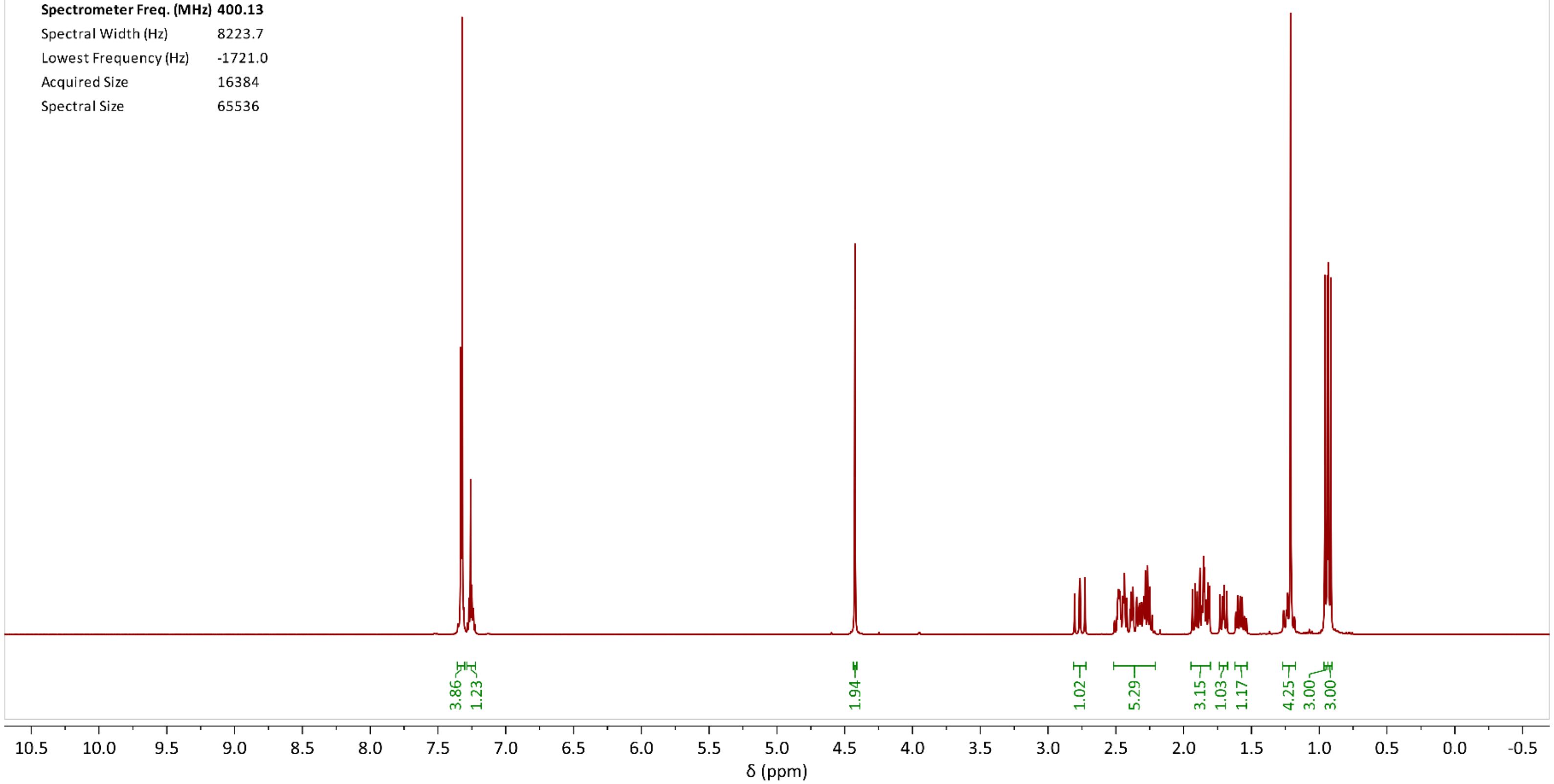

Benzyl hydrindanone 24

^{13}C NMR Spectrum, $CDCl_3$, 101 MHz

Origin	Bruker BioSpin GmbH
**Solvent**	**$CDCl_3$**
**Temperature (K)**	**294.8**
**Number of Scans**	**380**
Pulse Sequence	udeft
Receiver Gain	2050
Relaxation Delay (s)	3.0000
Pulse Width (μs)	8.8000
Acquisition Time (s)	0.3599
Acquisition Date	2018-08-20 18:55:00
**Nucleus**	**^{13}C**
**Spectrometer Freq. (MHz)**	**100.62**
Spectral Width (Hz)	25252.5
Lowest Frequency (Hz)	-1114.8
Acquired Size	9089
Spectral Size	65536

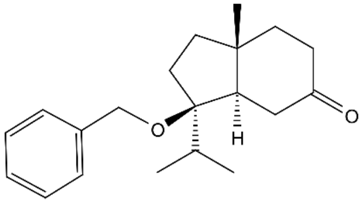

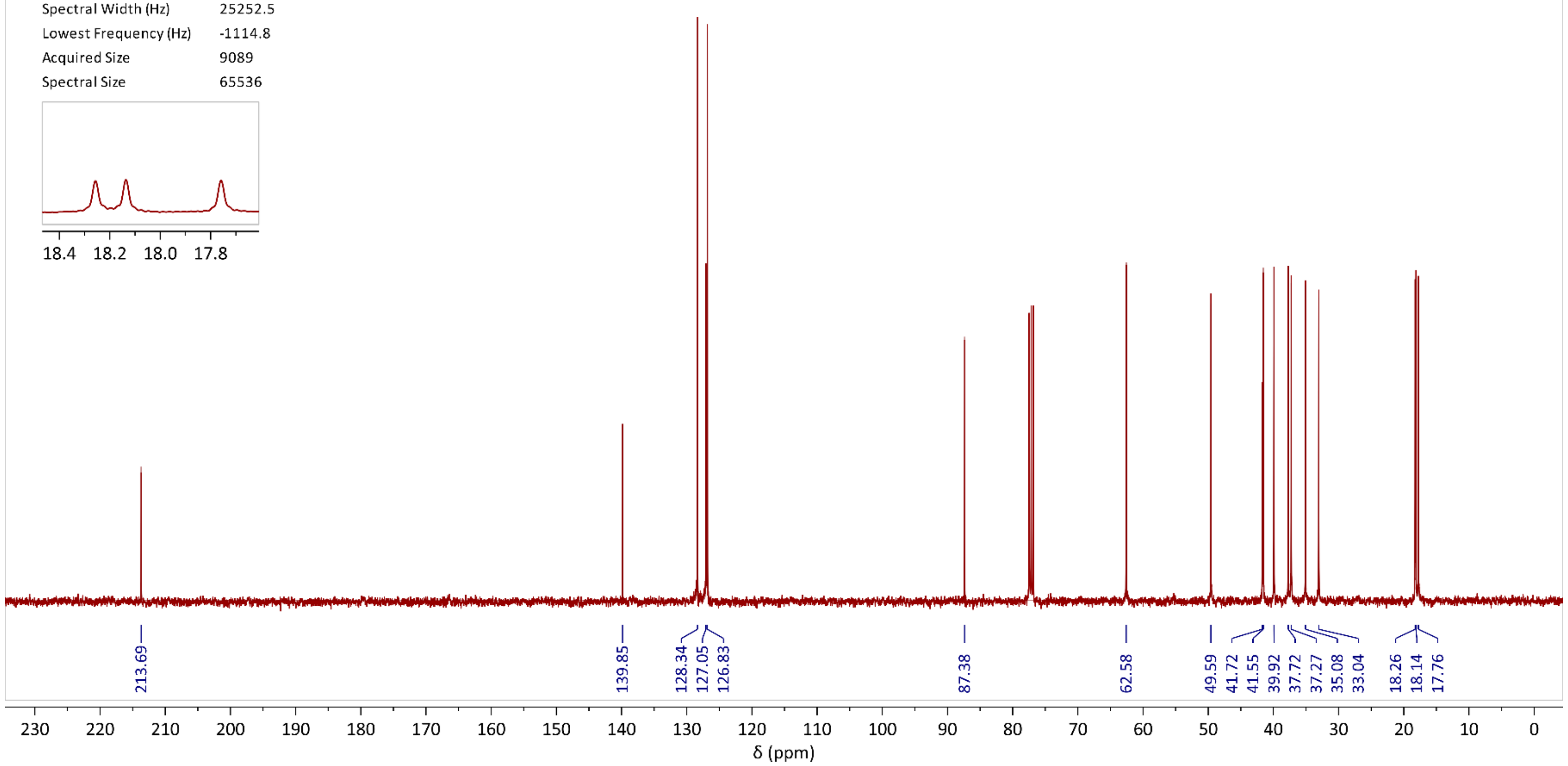

Bromoenal 25

^{1}H NMR Spectrum, $CDCl_3$, 300 MHz

Origin	Bruker BioSpin GmbH
**Solvent**	**$CDCl_3$**
**Temperature (K)**	**294.2**
**Number of Scans**	**32**
Pulse Sequence	zg30
Receiver Gain	406
Relaxation Delay (s)	1.0000
Pulse Width (µs)	12.8000
Acquisition Time (s)	2.7263
Acquisition Date	2015-10-15 11:52:00
**Nucleus**	**^{1}H**
**Spectrometer Freq. (MHz)**	**300.13**
Spectral Width (Hz)	6009.6
Lowest Frequency (Hz)	-1158.9
Acquired Size	16384
Spectral Size	65536

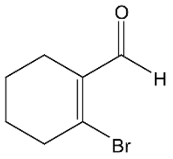

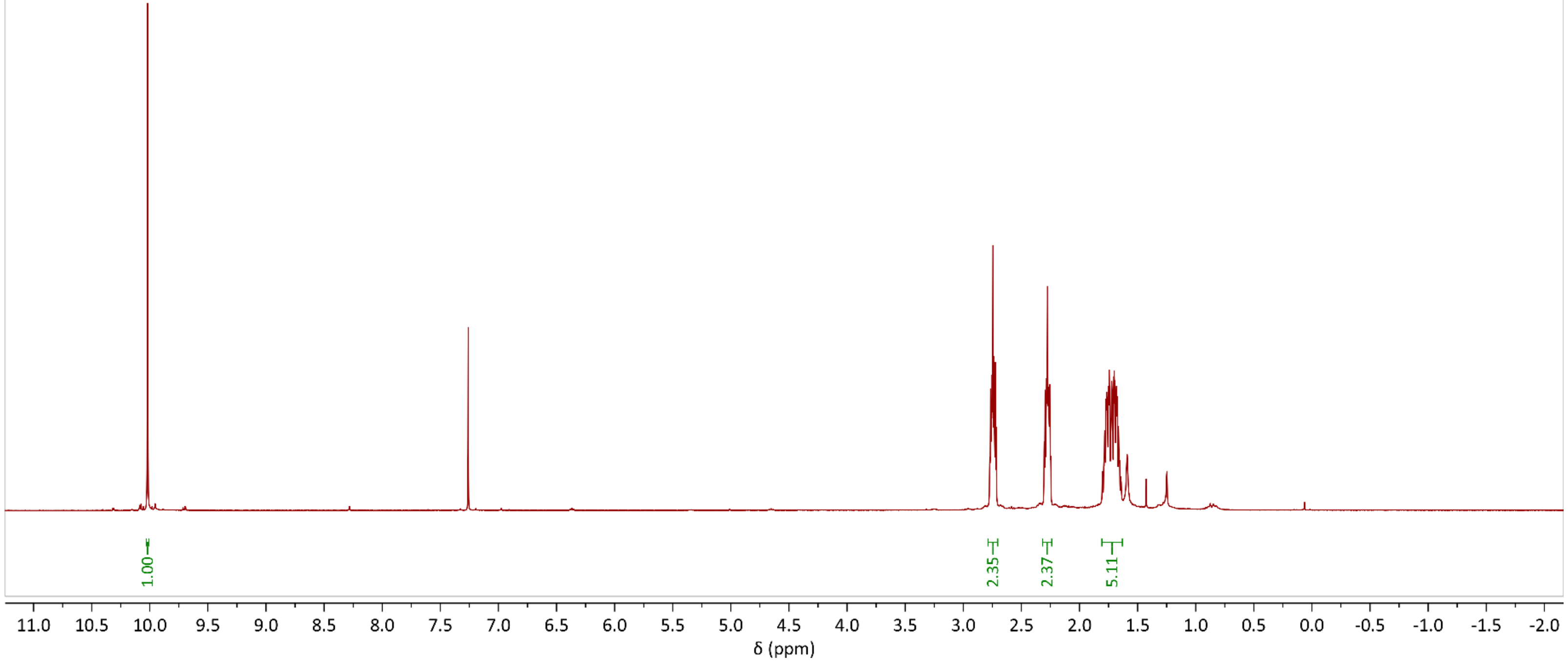

Bromoenal 25

^{13}C NMR Spectrum, $CDCl_3$, 101 MHz

Origin	Bruker BioSpin GmbH
**Solvent**	**$CDCl_3$**
**Temperature (K)**	**294.2**
**Number of Scans**	**380**
Pulse Sequence	udeft
Receiver Gain	2050
Relaxation Delay (s)	3.0000
Pulse Width (µs)	8.8000
Acquisition Time (s)	0.3599
Acquisition Date	2015-10-15 17:14:00
**Nucleus**	**^{13}C**
**Spectrometer Freq. (MHz)**	**100.62**
Spectral Width (Hz)	25252.5
Lowest Frequency (Hz)	-1112.3
Acquired Size	9089
Spectral Size	65536

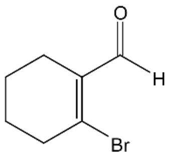

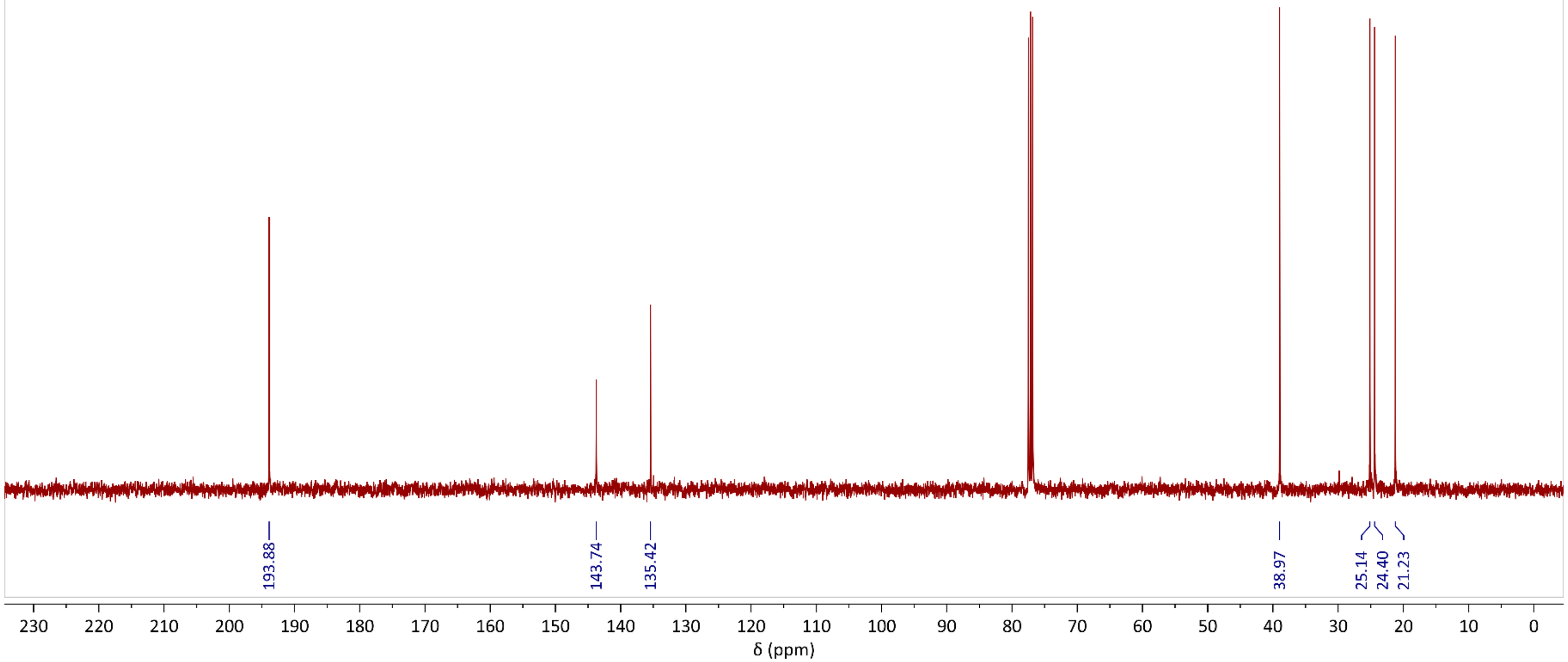

Enynal 26

^{1}H NMR Spectrum, $CDCl_3$, 300 MHz

Origin	Bruker BioSpin GmbH
**Solvent**	**$CDCl_3$**
**Temperature (K)**	**294.2**
**Number of Scans**	**32**
Pulse Sequence	zg30
Receiver Gain	72
Relaxation Delay (s)	1.0000
Pulse Width (μs)	12.8000
Acquisition Time (s)	2.7263
Acquisition Date	2015-10-20 11:33:00
**Nucleus**	**^{1}H**
**Spectrometer Freq. (MHz)**	**300.13**
Spectral Width (Hz)	6009.6
Lowest Frequency (Hz)	-1158.9
Acquired Size	16384
Spectral Size	65536

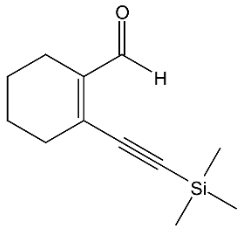

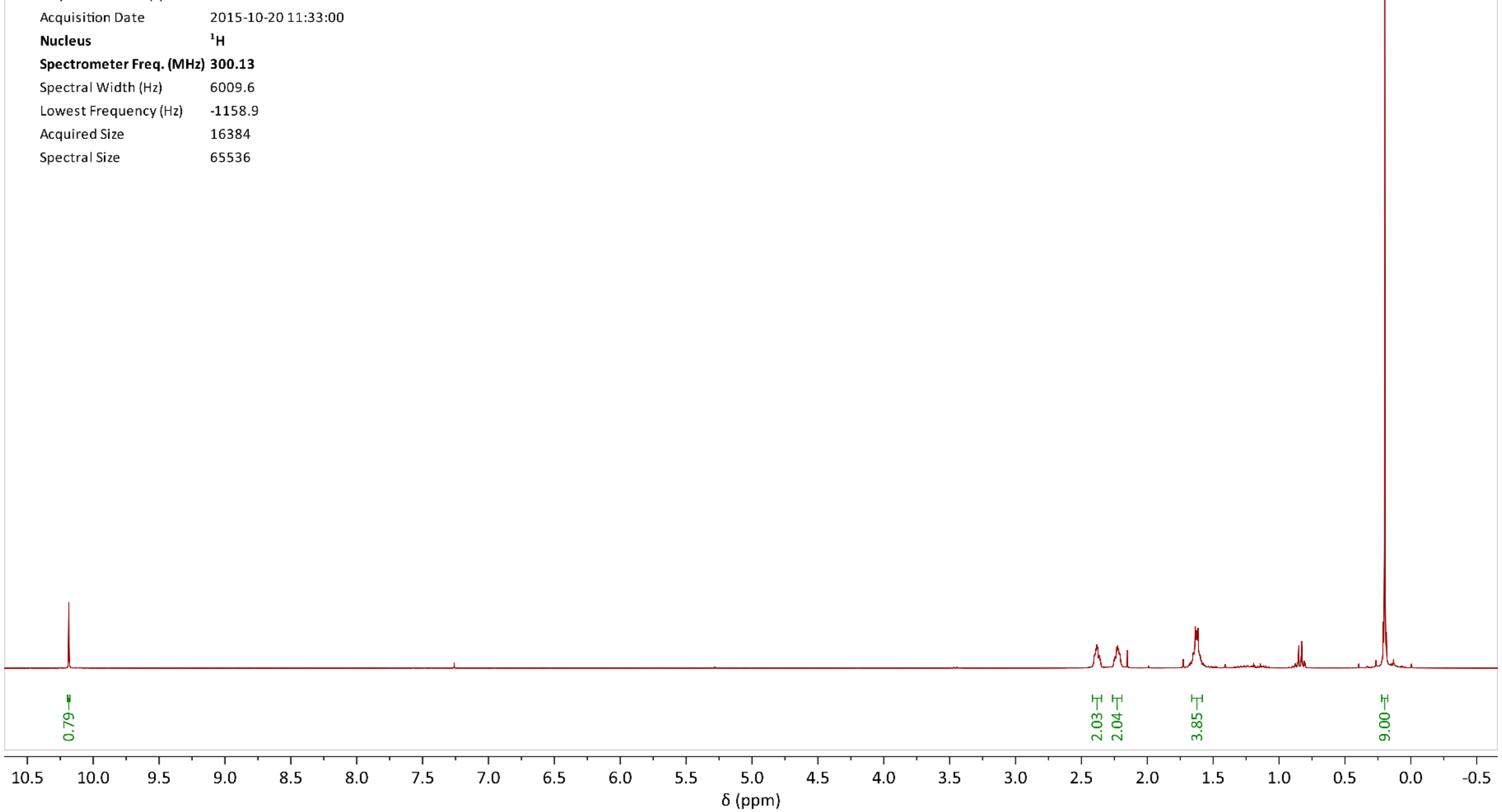

Enynal 26

^{13}C NMR Spectrum, $CDCl_3$, 101 MHz

Origin	Bruker BioSpin GmbH
**Solvent**	**$CDCl_3$**
**Temperature (K)**	**294.4**
**Number of Scans**	**380**
Pulse Sequence	udeft
Receiver Gain	2050
Relaxation Delay (s)	3.0000
Pulse Width (µs)	8.8000
Acquisition Time (s)	0.3599
Acquisition Date	2015-10-20 23:41:00
**Nucleus**	**^{13}C**
**Spectrometer Freq. (MHz)**	**100.62**
Spectral Width (Hz)	25252.5
Lowest Frequency (Hz)	-1113.7
Acquired Size	9089
Spectral Size	65536

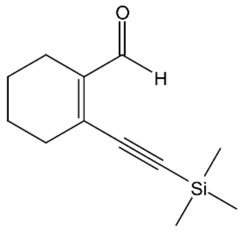

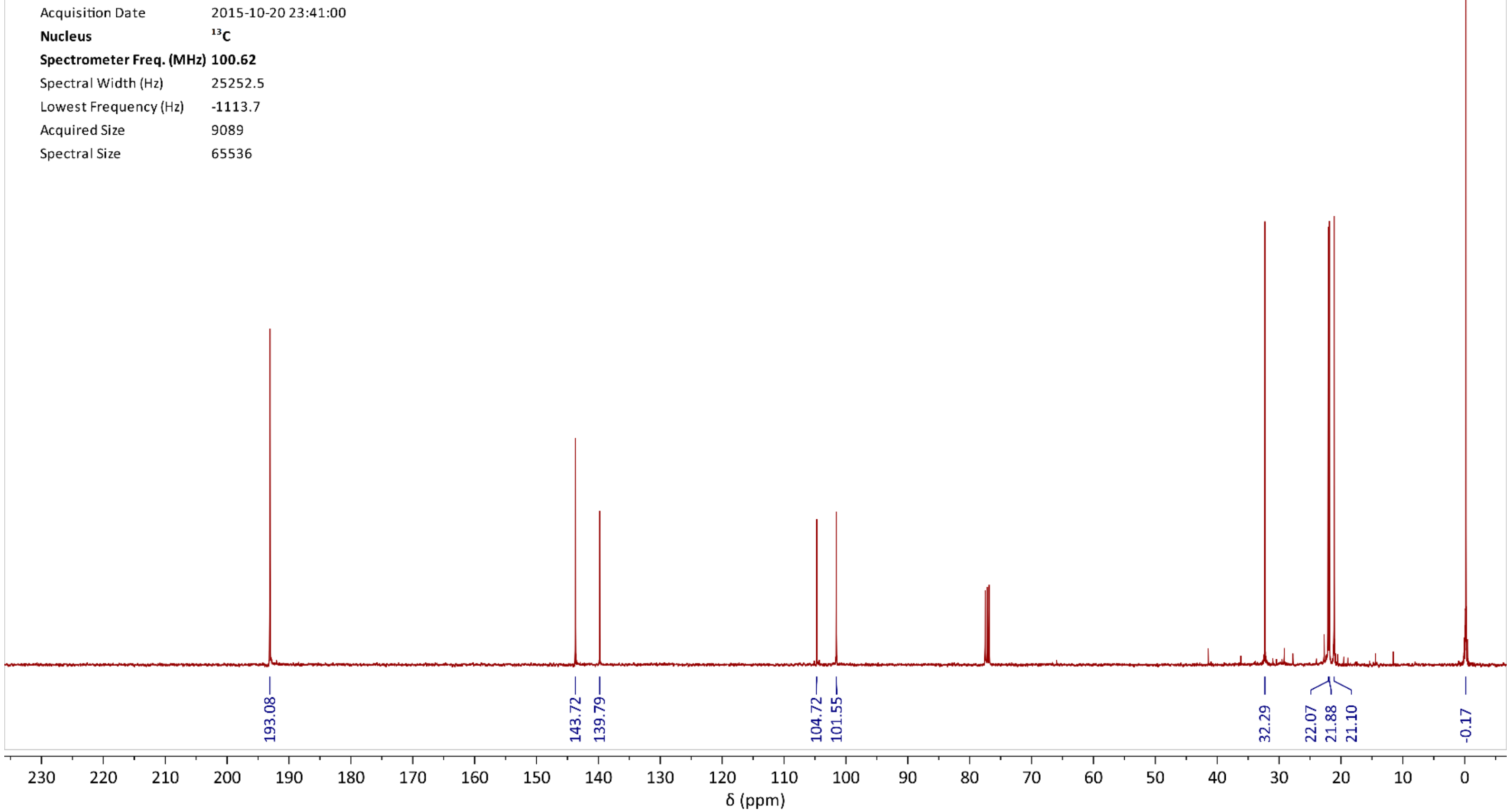

Enynol 27

^{1}H NMR Spectrum, $CDCl_3$, 400 MHz

Origin	Bruker BioSpin GmbH
**Solvent**	**$CDCl_3$**
**Temperature (K)**	**294.3**
**Number of Scans**	**32**
Pulse Sequence	zg30
Receiver Gain	144
Relaxation Delay (s)	1.5000
Pulse Width (μs)	9.5000
Acquisition Time (s)	1.9923
Acquisition Date	2015-11-02 11:06:00
**Nucleus**	**^{1}H**
**Spectrometer Freq. (MHz)**	**400.13**
Spectral Width (Hz)	8223.7
Lowest Frequency (Hz)	-1721.0
Acquired Size	16384
Spectral Size	65536

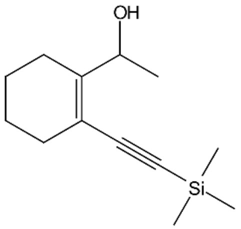

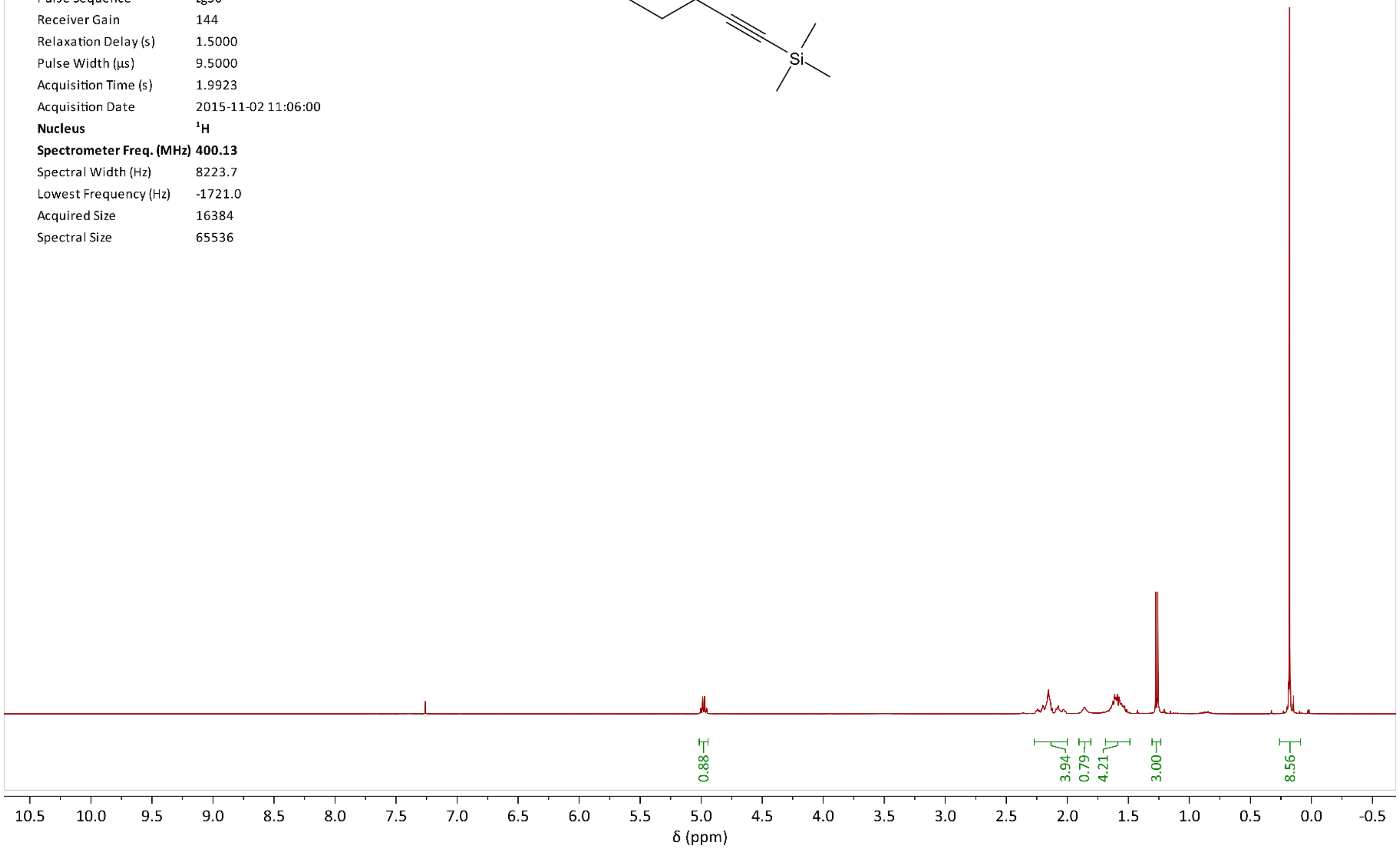

Enynol 27

^{13}C NMR Spectrum, $CDCl_3$, 101 MHz

Origin	Bruker BioSpin GmbH
**Solvent**	**$CDCl_3$**
**Temperature (K)**	**294.4**
**Number of Scans**	**380**
Pulse Sequence	udeft
Receiver Gain	2050
Relaxation Delay (s)	3.0000
Pulse Width (μs)	8.8000
Acquisition Time (s)	0.3599
Acquisition Date	2015-11-02 12:43:00
**Nucleus**	**^{13}C**
**Spectrometer Freq. (MHz)**	**100.62**
Spectral Width (Hz)	25252.5
Lowest Frequency (Hz)	-1131.0
Acquired Size	9089
Spectral Size	65536

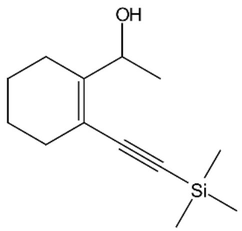

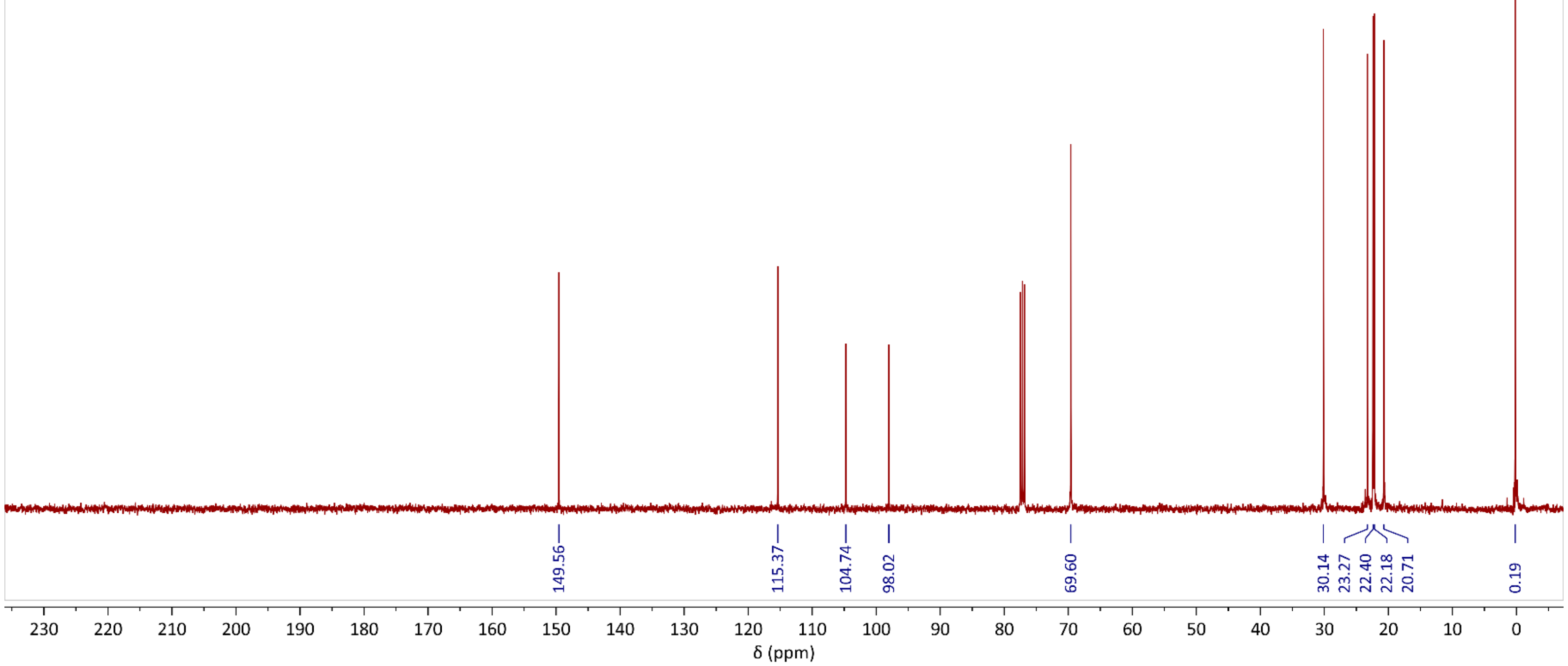

Furan 28

^1H NMR Spectrum, $CDCl_3$, 400 MHz

Origin	Bruker BioSpin GmbH
**Solvent**	**$CDCl_3$**
**Temperature (K)**	**294.9**
**Number of Scans**	**32**
Pulse Sequence	zg30
Receiver Gain	512
Relaxation Delay (s)	1.5000
Pulse Width (µs)	9.5000
Acquisition Time (s)	1.9923
Acquisition Date	2015-11-23 13:12:00
**Nucleus**	**^1H**
**Spectrometer Freq. (MHz)**	**400.13**
Spectral Width (Hz)	8223.7
Lowest Frequency (Hz)	-1720.9
Acquired Size	16384
Spectral Size	65536

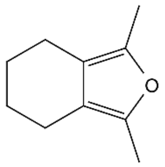

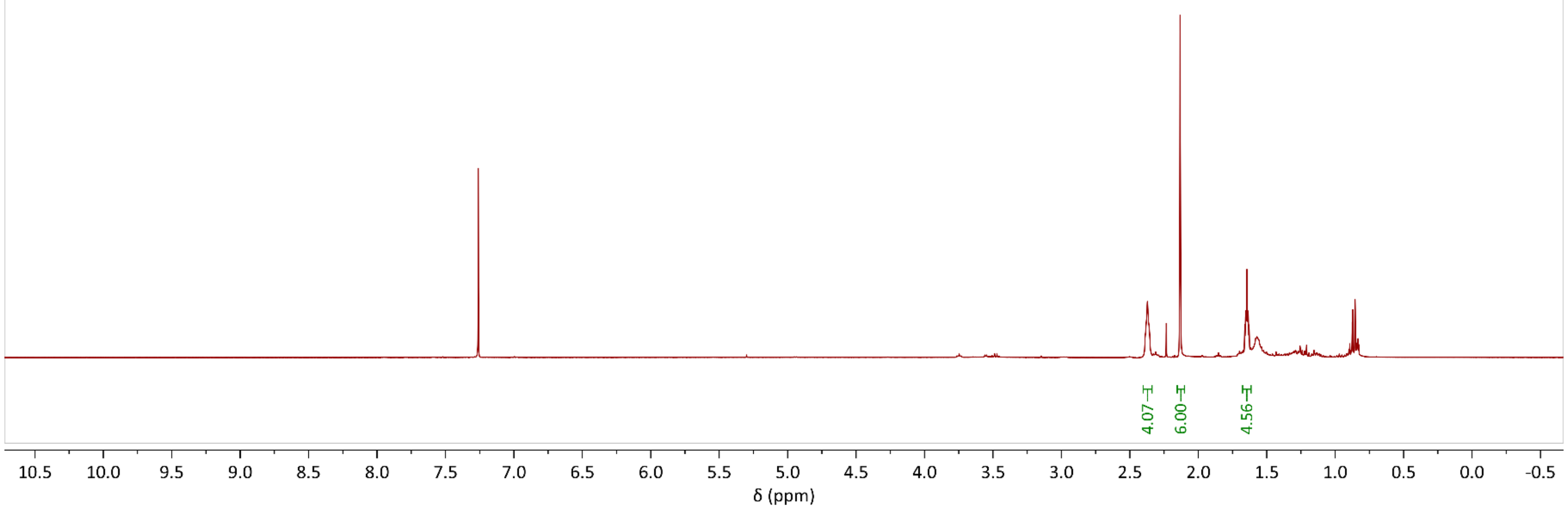

Furan 28

^{13}C NMR Spectrum, $CDCl_3$, 101 MHz

Origin	Bruker BioSpin GmbH
**Solvent**	**$CDCl_3$**
**Temperature (K)**	**294.5**
**Number of Scans**	**380**
Pulse Sequence	udeft
Receiver Gain	2050
Relaxation Delay (s)	3.0000
Pulse Width (µs)	8.8000
Acquisition Time (s)	0.3599
Acquisition Date	2015-11-23 12:44:00
**Nucleus**	**^{13}C**
**Spectrometer Freq. (MHz)**	**100.62**
Spectral Width (Hz)	25252.5
Lowest Frequency (Hz)	-1111.5
Acquired Size	9089
Spectral Size	65536

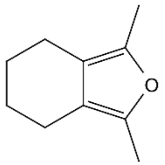

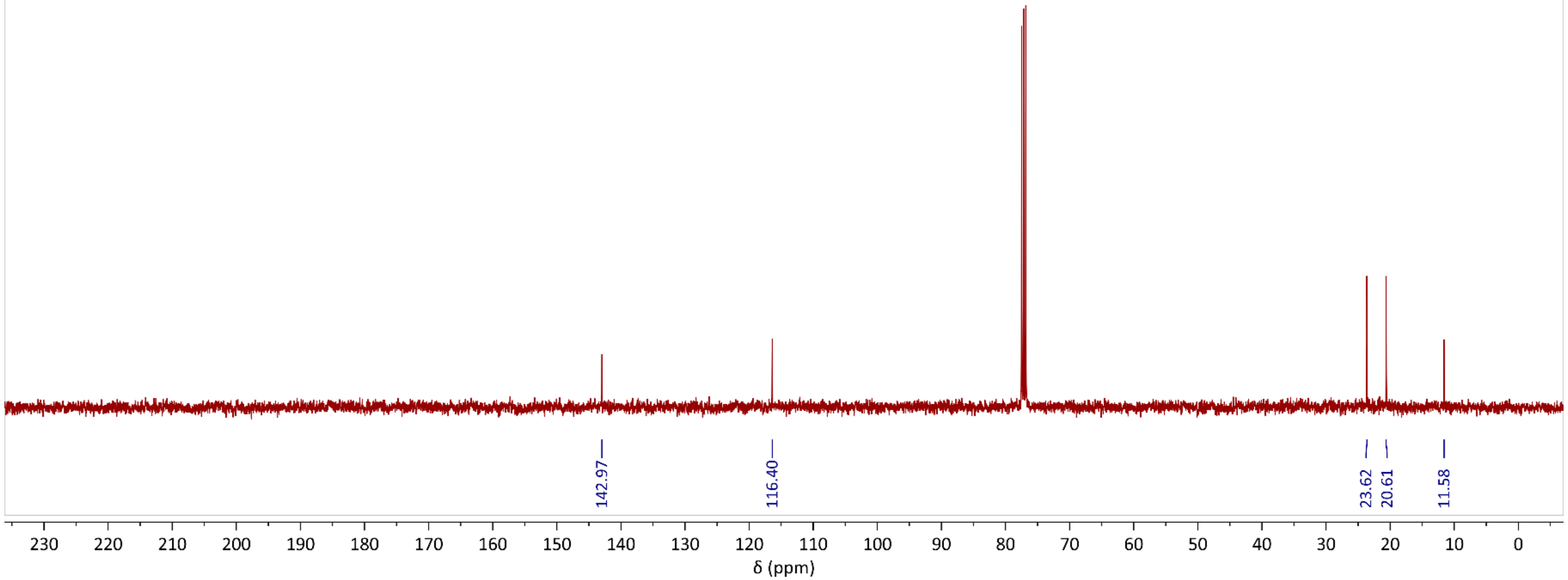

Silyl enol ether 29

^{1}H NMR Spectrum, $CDCl_3$, 400 MHz

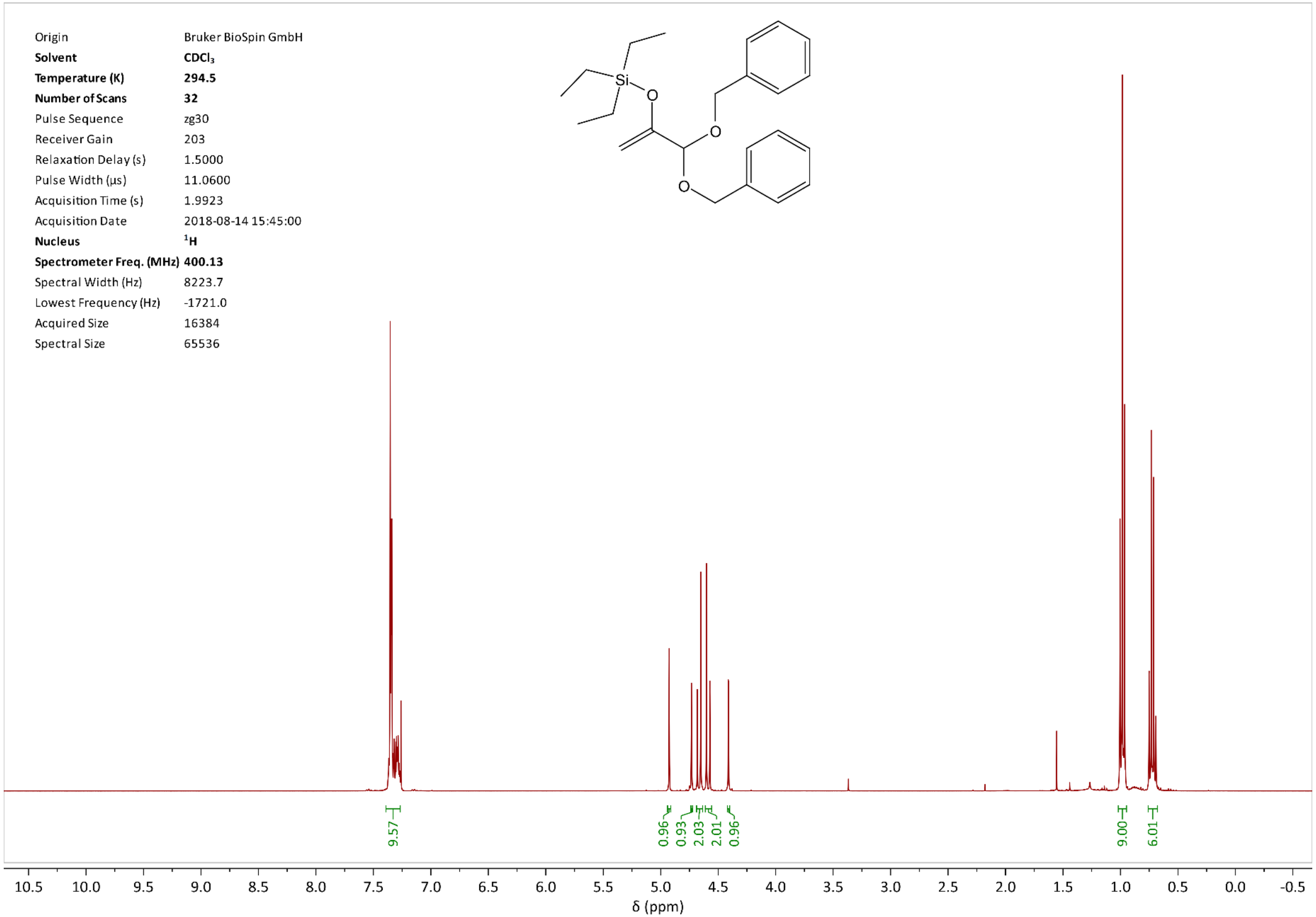

Silyl enol ether 29

^{13}C NMR Spectrum, $CDCl_3$, 101 MHz

Origin	Bruker BioSpin GmbH
**Solvent**	**$CDCl_3$**
**Temperature (K)**	**294.9**
**Number of Scans**	**380**
Pulse Sequence	udeft
Receiver Gain	2050
Relaxation Delay (s)	3.0000
Pulse Width (μs)	8.8000
Acquisition Time (s)	0.3599
Acquisition Date	2018-08-14 15:55:00
**Nucleus**	**^{13}C**
**Spectrometer Freq. (MHz)**	**100.62**
Spectral Width (Hz)	25252.5
Lowest Frequency (Hz)	-1114.2
Acquired Size	9089
Spectral Size	65536

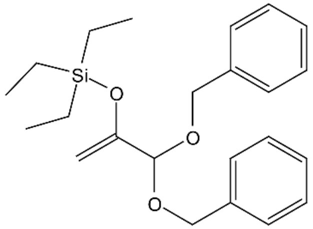

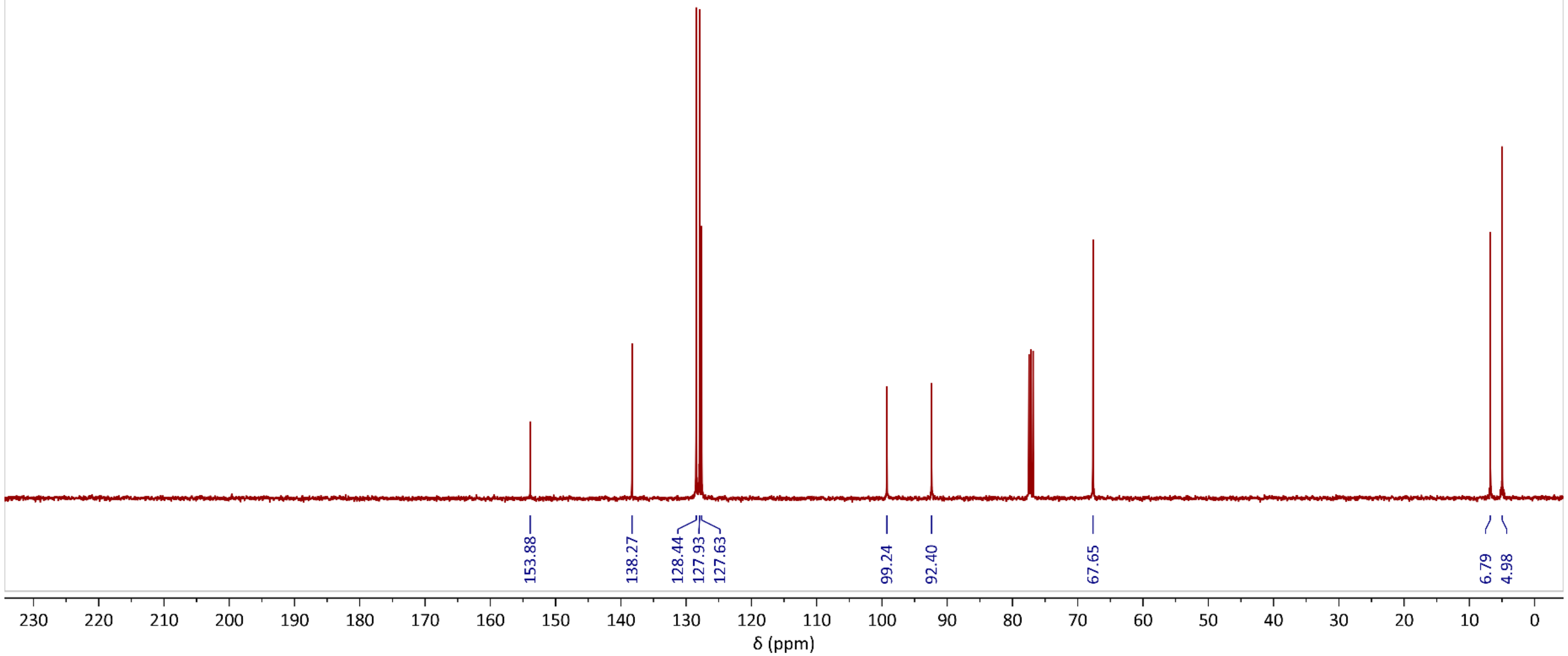

Oxabicyclic ketones 30 and 31

^{1}H NMR Spectrum, $CDCl_3$, 400 MHz

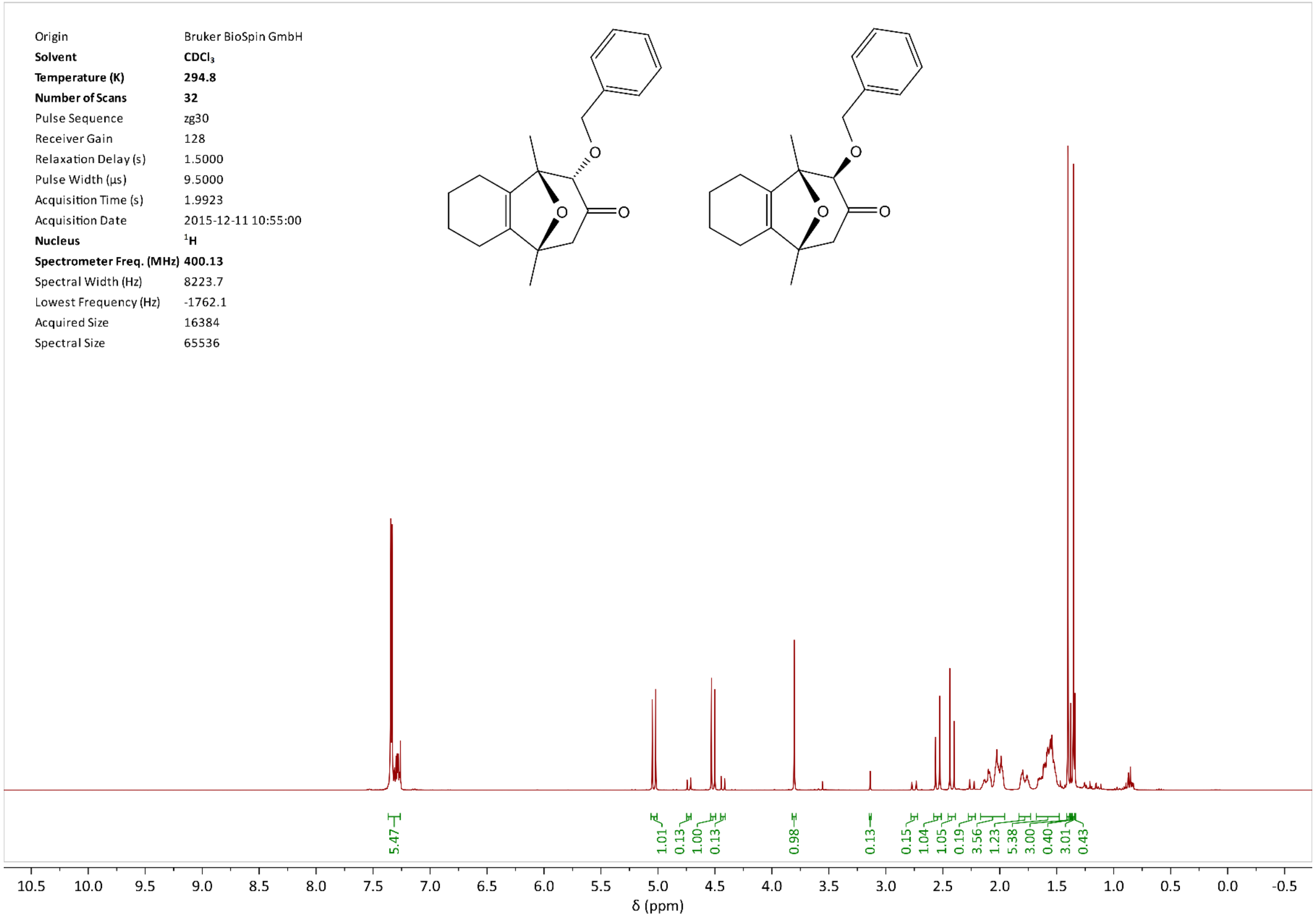

Oxabicyclic ketones 30 and 31

^{13}C NMR Spectrum, $CDCl_3$, 101 MHz

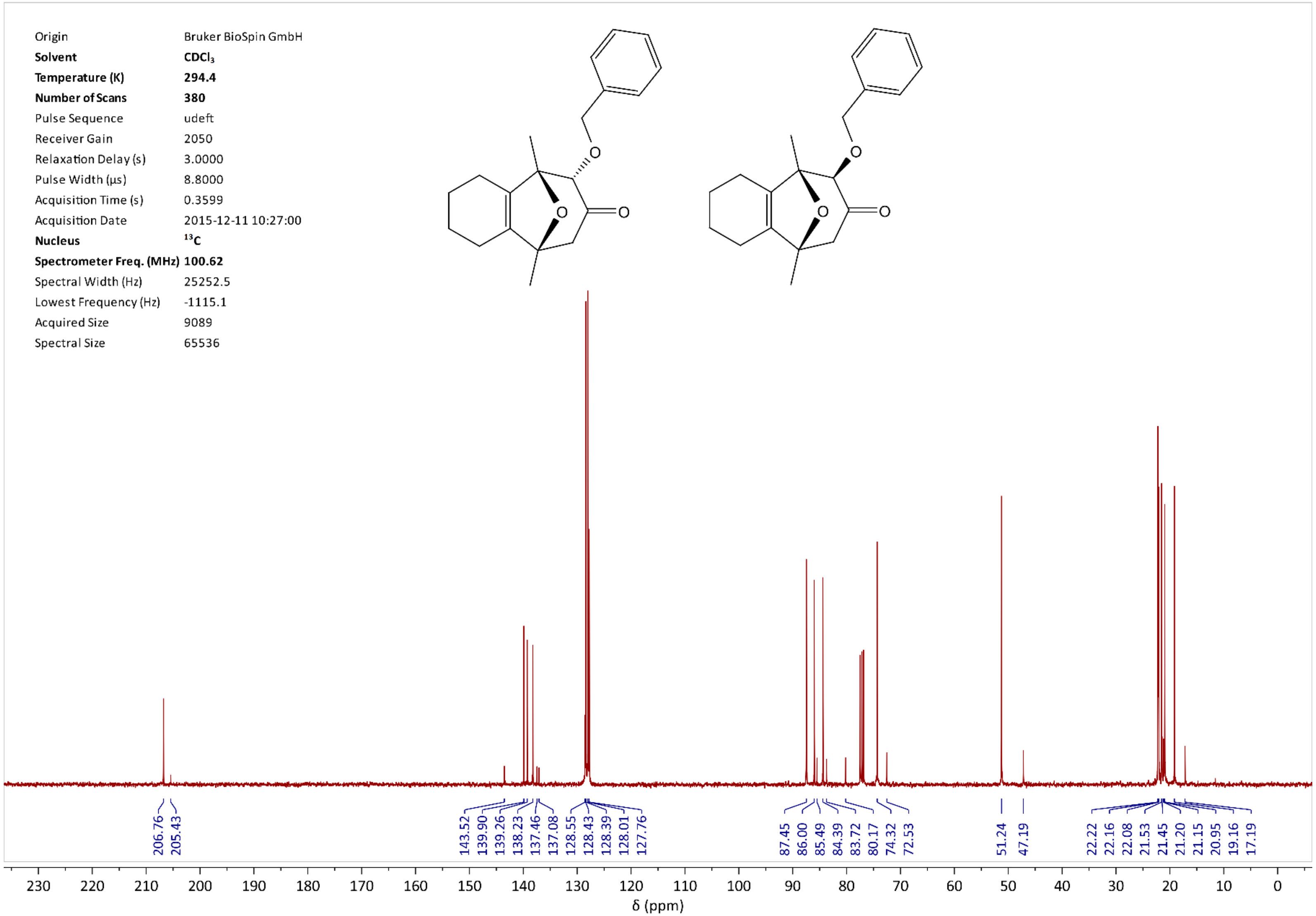

Chloroenal 33

^1H NMR Spectrum, $CDCl_3$, 400 MHz

Origin	Bruker BioSpin GmbH
**Solvent**	**$CDCl_3$**
**Temperature (K)**	**294.6**
**Number of Scans**	**32**
Pulse Sequence	zg30
Receiver Gain	203
Relaxation Delay (s)	1.5000
Pulse Width (μs)	11.0600
Acquisition Time (s)	1.9923
Acquisition Date	2018-07-19 09:09:00
**Nucleus**	**^1H**
**Spectrometer Freq. (MHz)**	**400.13**
Spectral Width (Hz)	8223.7
Lowest Frequency (Hz)	-1721.0
Acquired Size	16384
Spectral Size	65536

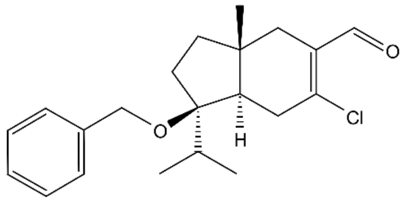

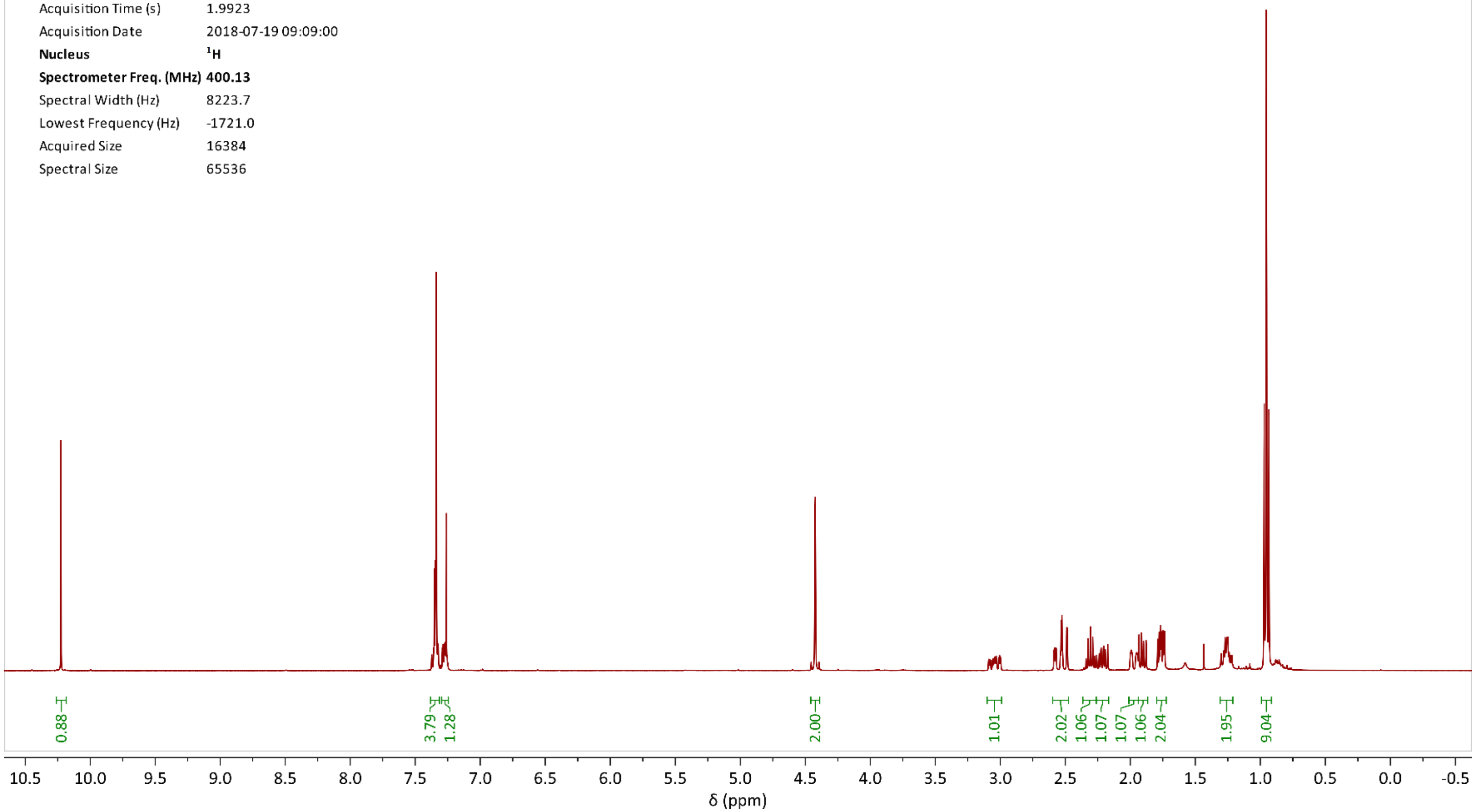

Chloroenal 33

^{13}C NMR Spectrum, $CDCl_3$, 101 MHz

Origin	Bruker BioSpin GmbH
**Solvent**	**$CDCl_3$**
**Temperature (K)**	**295.1**
**Number of Scans**	**380**
Pulse Sequence	udeft
Receiver Gain	2050
Relaxation Delay (s)	3.0000
Pulse Width (μs)	8.8000
Acquisition Time (s)	0.3599
Acquisition Date	2018-07-19 09:19:00
**Nucleus**	**^{13}C**
**Spectrometer Freq. (MHz)**	**100.62**
Spectral Width (Hz)	25252.5
Lowest Frequency (Hz)	-1113.7
Acquired Size	9089
Spectral Size	65536

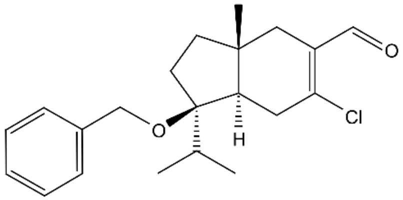

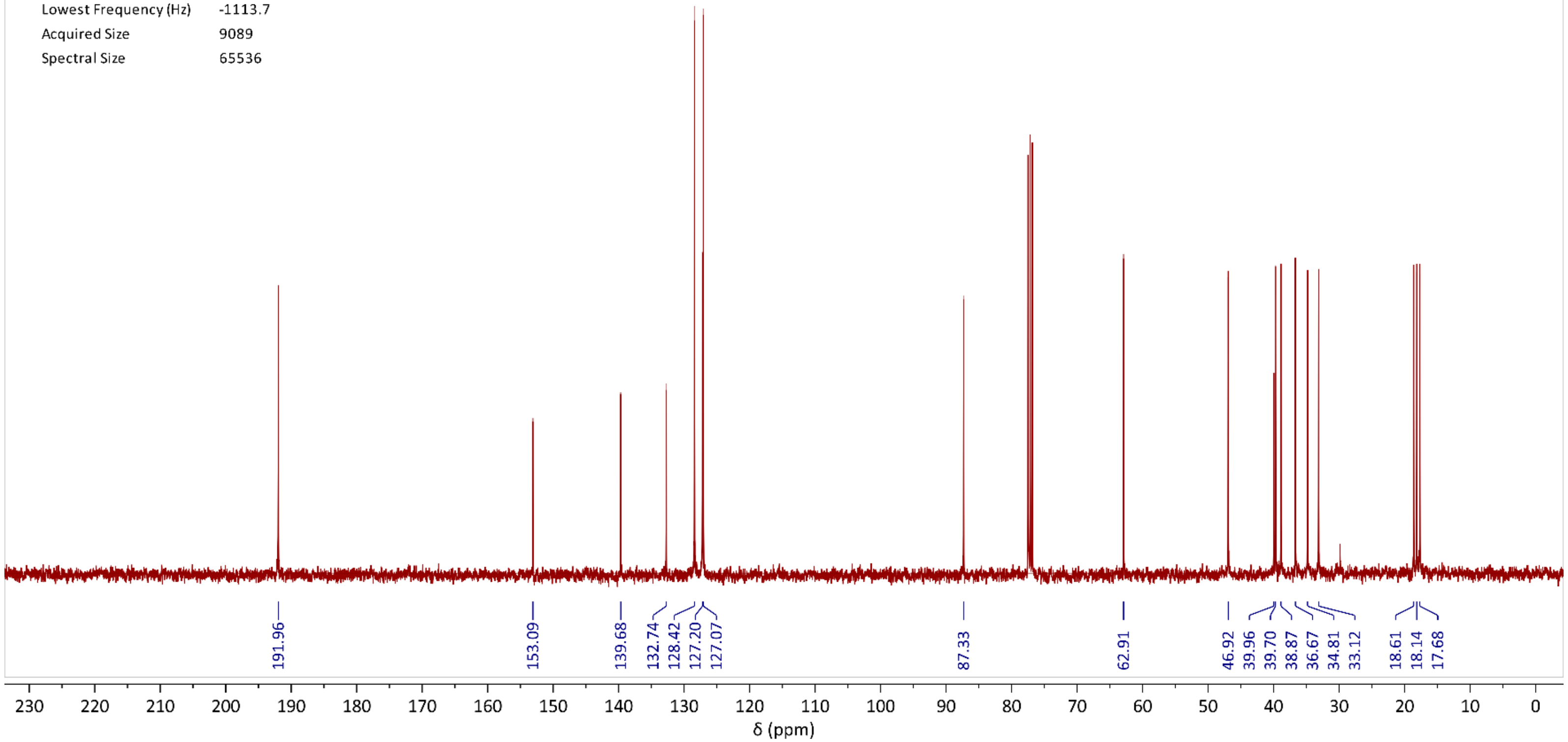

Keto alcohol 35

^{1}H NMR Spectrum, $CDCl_3$, 400 MHz

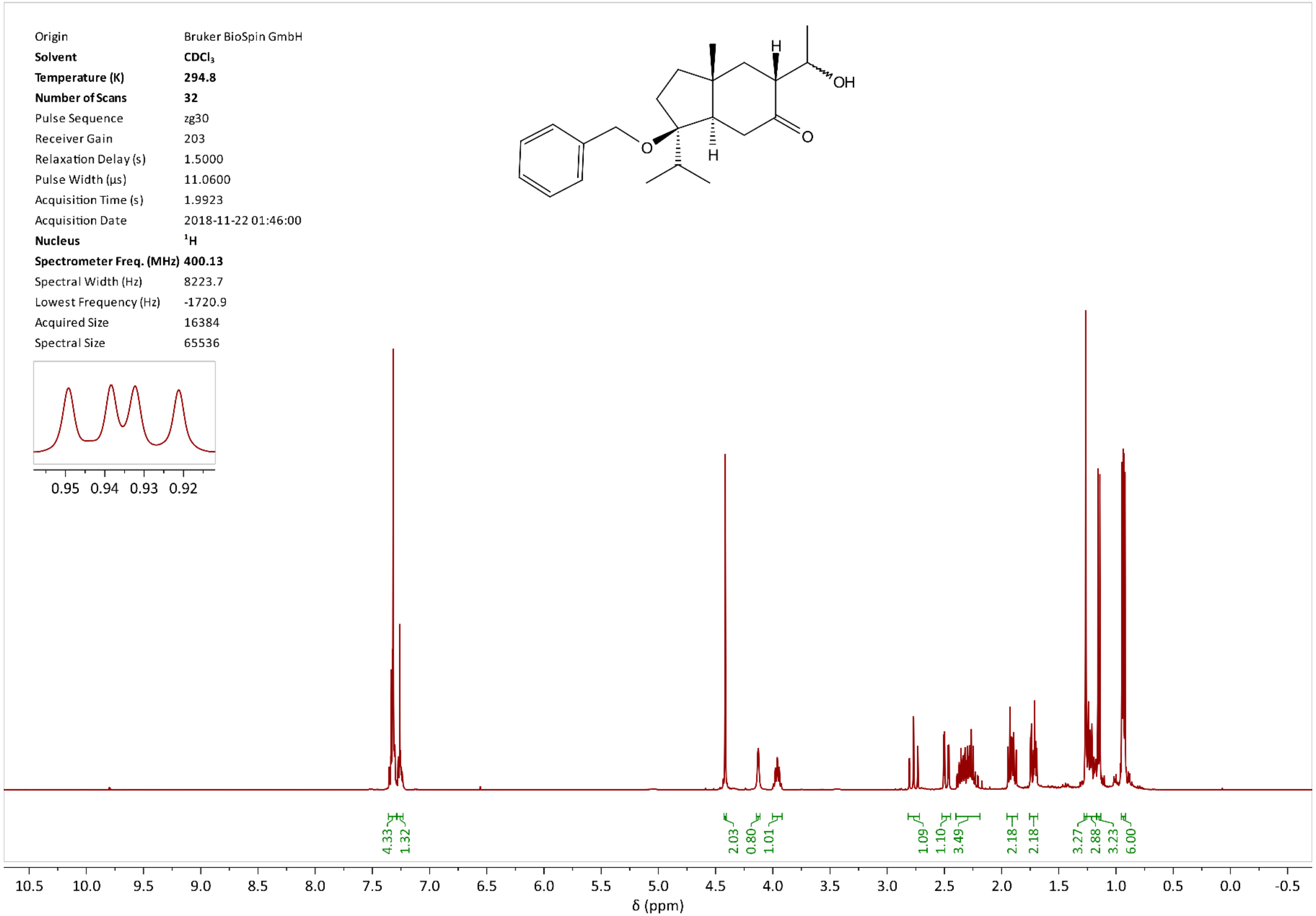

Keto alcohol 35

^{13}C NMR Spectrum, $CDCl_3$, 101 MHz

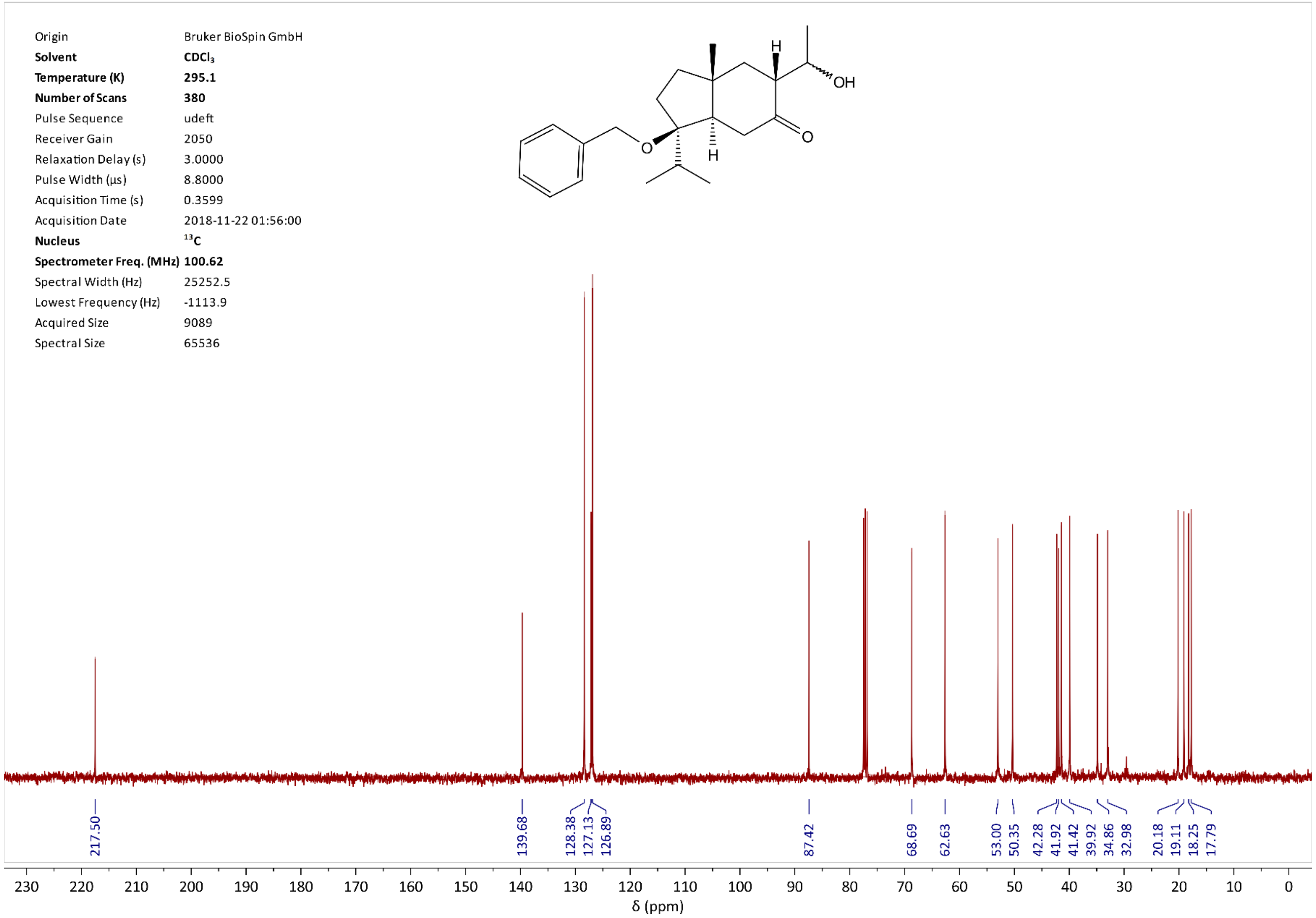

Alkyne 1,3-diol 36

^{1}H NMR Spectrum, $CDCl_3$, 400 MHz

Origin	Bruker BioSpin GmbH
**Solvent**	**$CDCl_3$**
**Temperature (K)**	**297.9**
**Number of Scans**	**2**
Pulse Sequence	zg30
Receiver Gain	89
Relaxation Delay (s)	2.0000
Pulse Width (µs)	10.0000
Acquisition Time (s)	4.5875
Acquisition Date	2018-12-21 00:49:35
**Nucleus**	**^{1}H**
**Spectrometer Freq. (MHz)**	**400.13**
Spectral Width (Hz)	7142.9
Lowest Frequency (Hz)	-1180.4
Acquired Size	32768
Spectral Size	65536

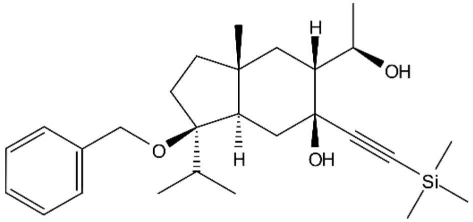

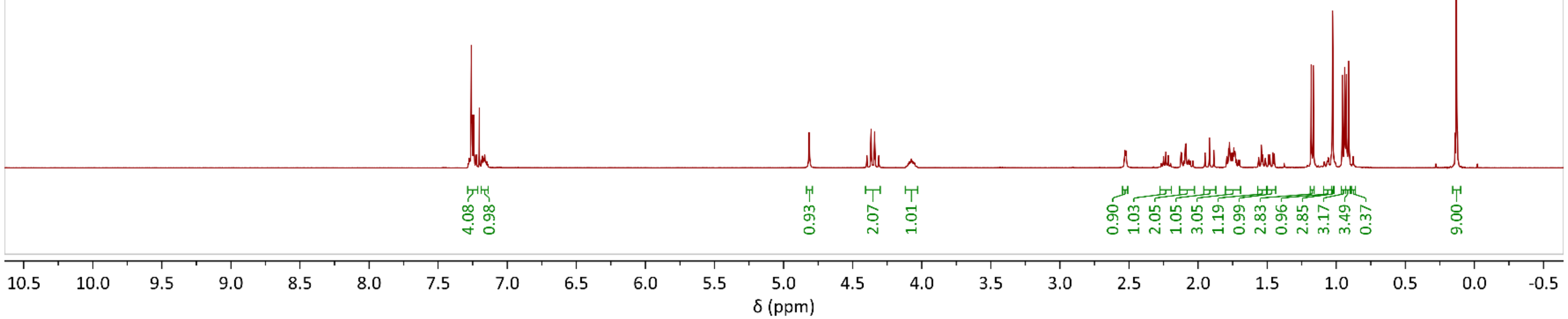

Alkyne 1,3-diol 36

^{13}C NMR Spectrum, $CDCl_3$, 101 MHz

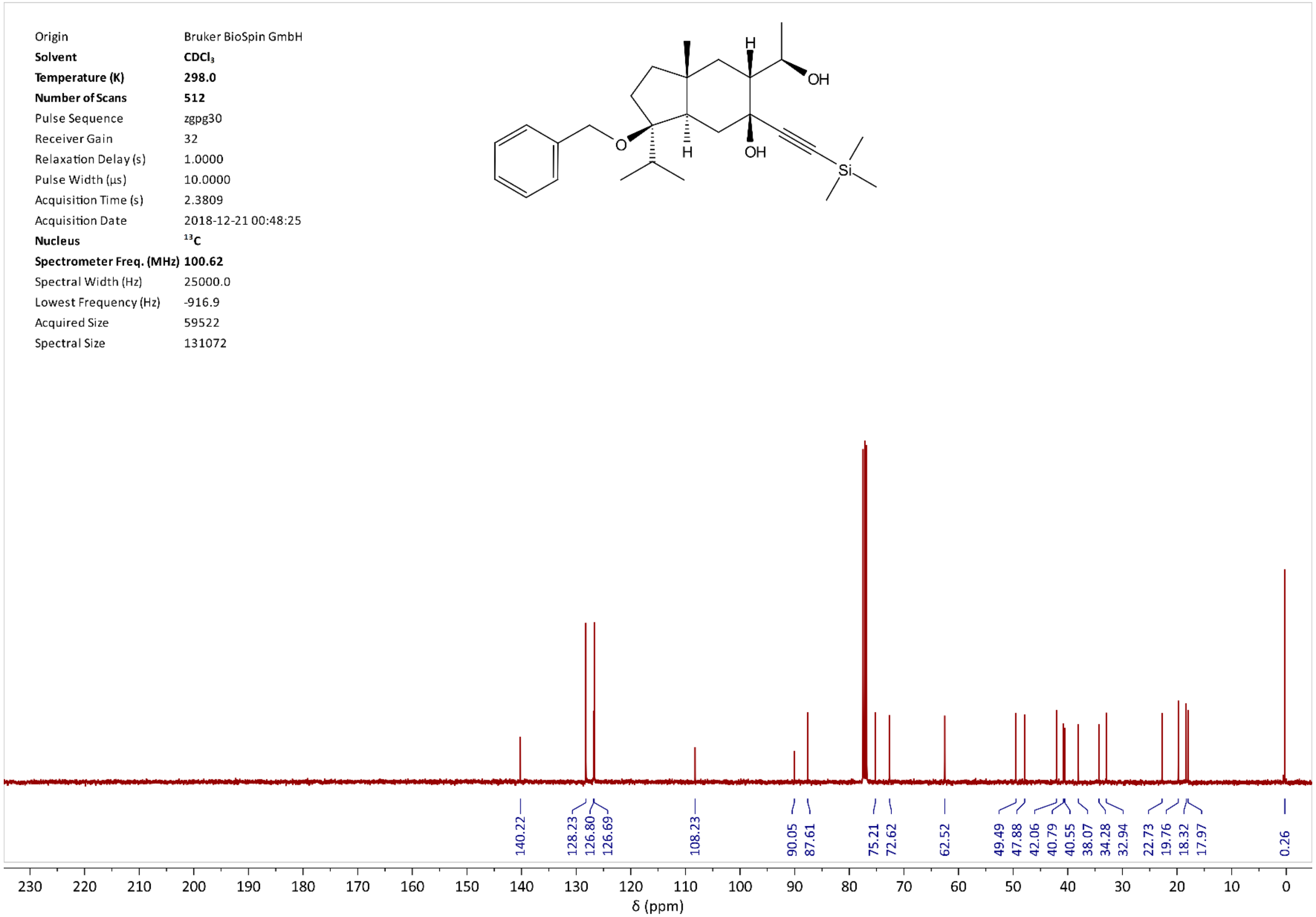

Alkyne 1,3-diol 37

^{1}H NMR Spectrum, $CDCl_3$, 400 MHz

Origin	Bruker BioSpin GmbH
**Solvent**	**$CDCl_3$**
**Temperature (K)**	**297.9**
**Number of Scans**	**2**
Pulse Sequence	zg30
Receiver Gain	101
Relaxation Delay (s)	2.0000
Pulse Width (µs)	10.0000
Acquisition Time (s)	4.5875
Acquisition Date	2018-12-20 22:29:08
**Nucleus**	**^{1}H**
**Spectrometer Freq. (MHz)**	**400.13**
Spectral Width (Hz)	7142.9
Lowest Frequency (Hz)	-1180.4
Acquired Size	32768
Spectral Size	65536

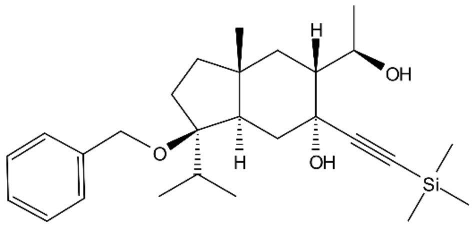

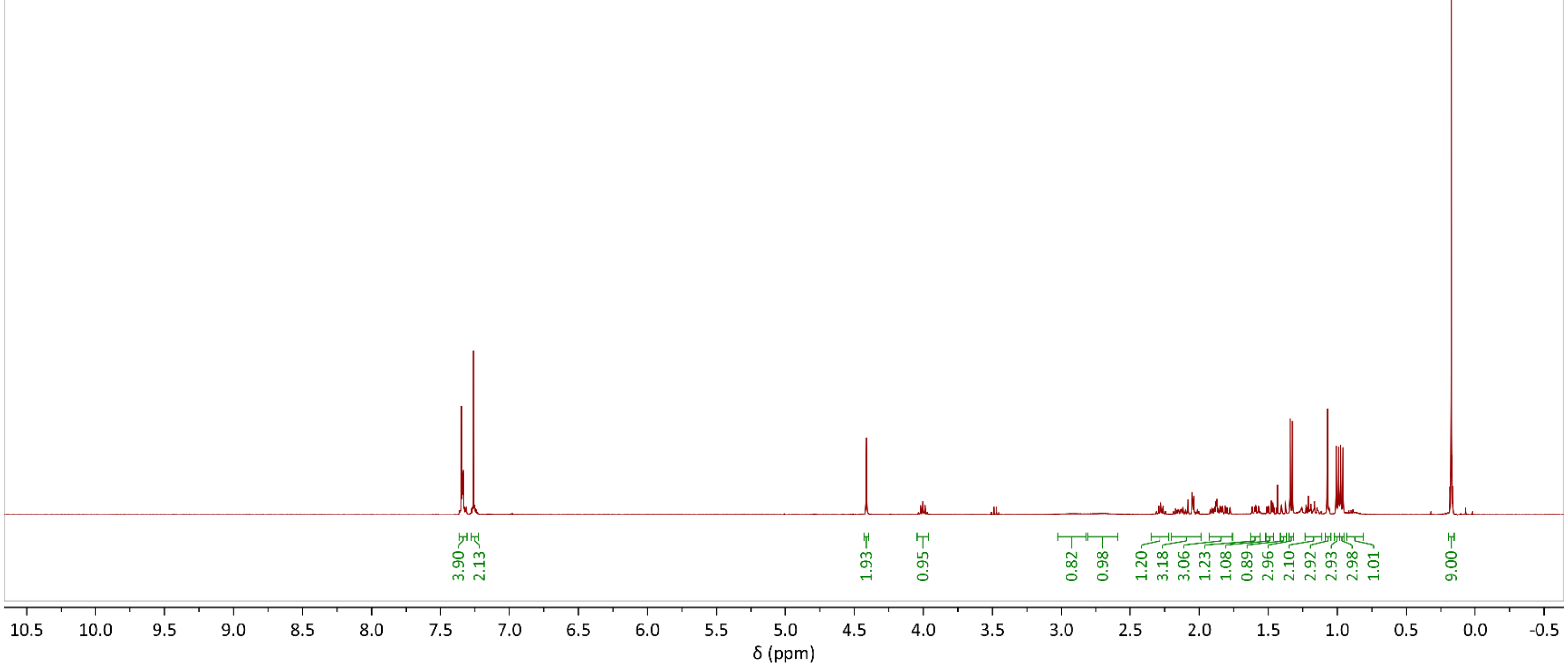

Alkyne 1,3-diol 37

^{13}C NMR Spectrum, $CDCl_3$, 101 MHz

Origin	Bruker BioSpin GmbH
**Solvent**	**$CDCl_3$**
**Temperature (K)**	**298.0**
**Number of Scans**	**512**
Pulse Sequence	zgpg30
Receiver Gain	32
Relaxation Delay (s)	1.0000
Pulse Width (µs)	10.0000
Acquisition Time (s)	2.3809
Acquisition Date	2018-12-20 22:27:58
**Nucleus**	**^{13}C**
**Spectrometer Freq. (MHz)**	**100.62**
Spectral Width (Hz)	25000.0
Lowest Frequency (Hz)	-915.8
Acquired Size	59522
Spectral Size	131072

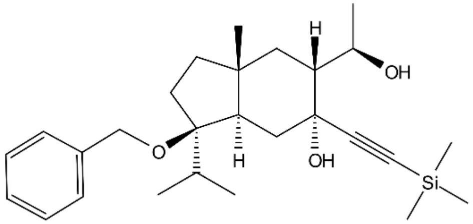

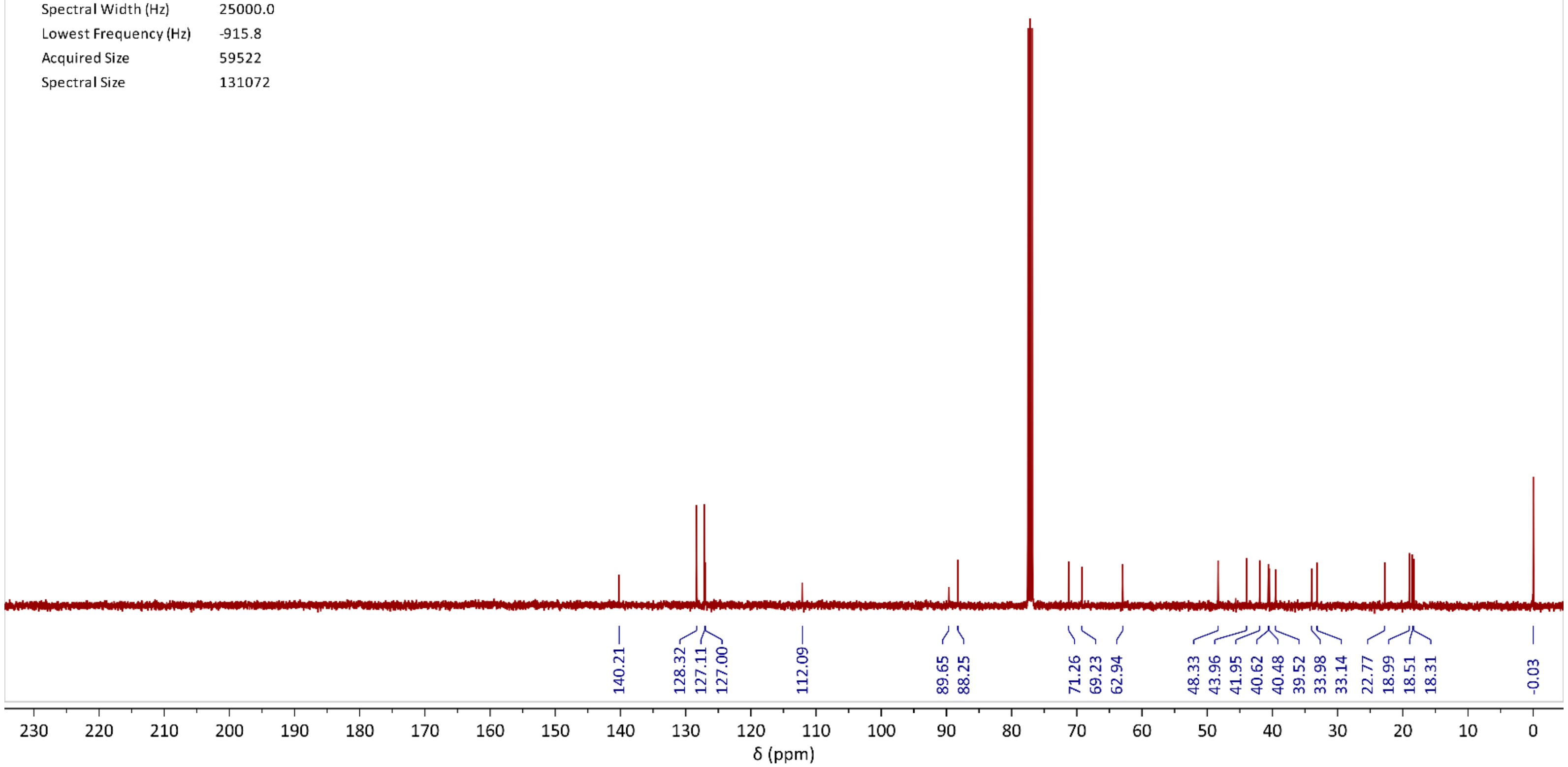

Alkyne 1,3-diol 38

^{1}H NMR Spectrum, $CDCl_3$, 400 MHz

Origin	Bruker BioSpin GmbH
**Solvent**	**$CDCl_3$**
**Temperature (K)**	**297.9**
**Number of Scans**	**2**
Pulse Sequence	zg30
Receiver Gain	101
Relaxation Delay (s)	2.0000
Pulse Width (μs)	10.0000
Acquisition Time (s)	4.5875
Acquisition Date	2018-12-20 20:11:23
**Nucleus**	**^{1}H**
**Spectrometer Freq. (MHz)**	**400.13**
Spectral Width (Hz)	7142.9
Lowest Frequency (Hz)	-1180.2
Acquired Size	32768
Spectral Size	65536

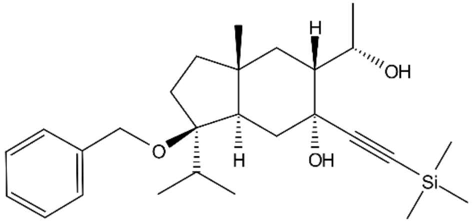

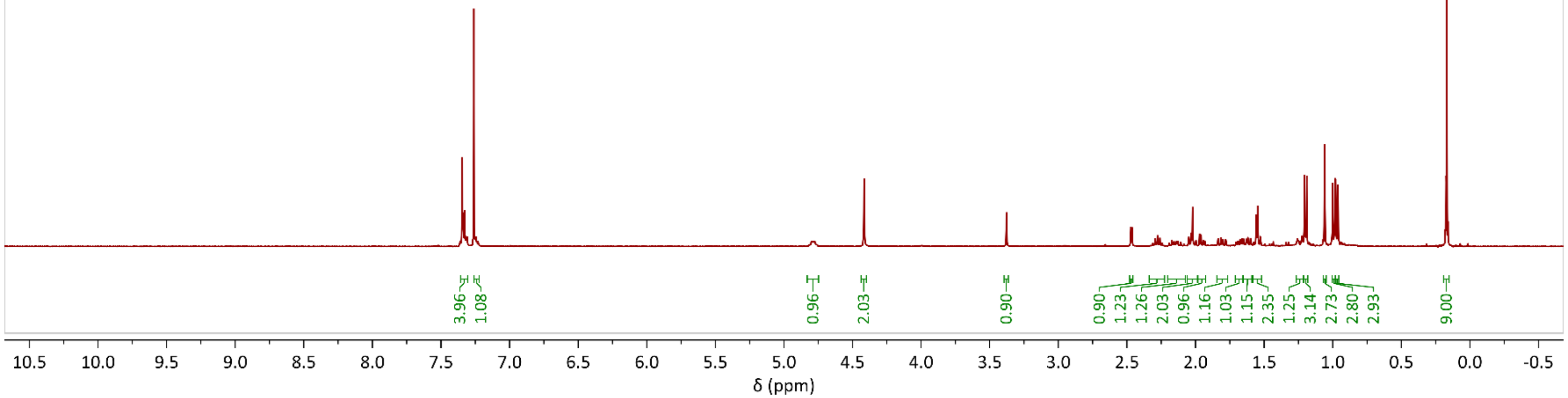

Alkyne 1,3-diol 38

^{13}C NMR Spectrum, $CDCl_3$, 101 MHz

Origin	Bruker BioSpin GmbH
**Solvent**	**$CDCl_3$**
**Temperature (K)**	**298.0**
**Number of Scans**	**512**
Pulse Sequence	zgpg30
Receiver Gain	32
Relaxation Delay (s)	1.0000
Pulse Width (µs)	10.0000
Acquisition Time (s)	2.3809
Acquisition Date	2018-12-20 20:10:13
**Nucleus**	**^{13}C**
**Spectrometer Freq. (MHz)**	**100.62**
Spectral Width (Hz)	25000.0
Lowest Frequency (Hz)	-915.6
Acquired Size	59522
Spectral Size	131072

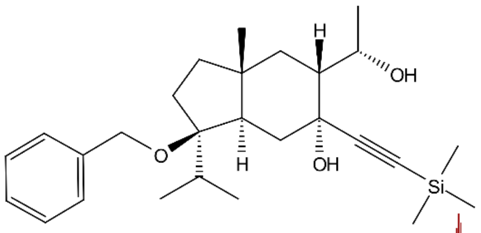

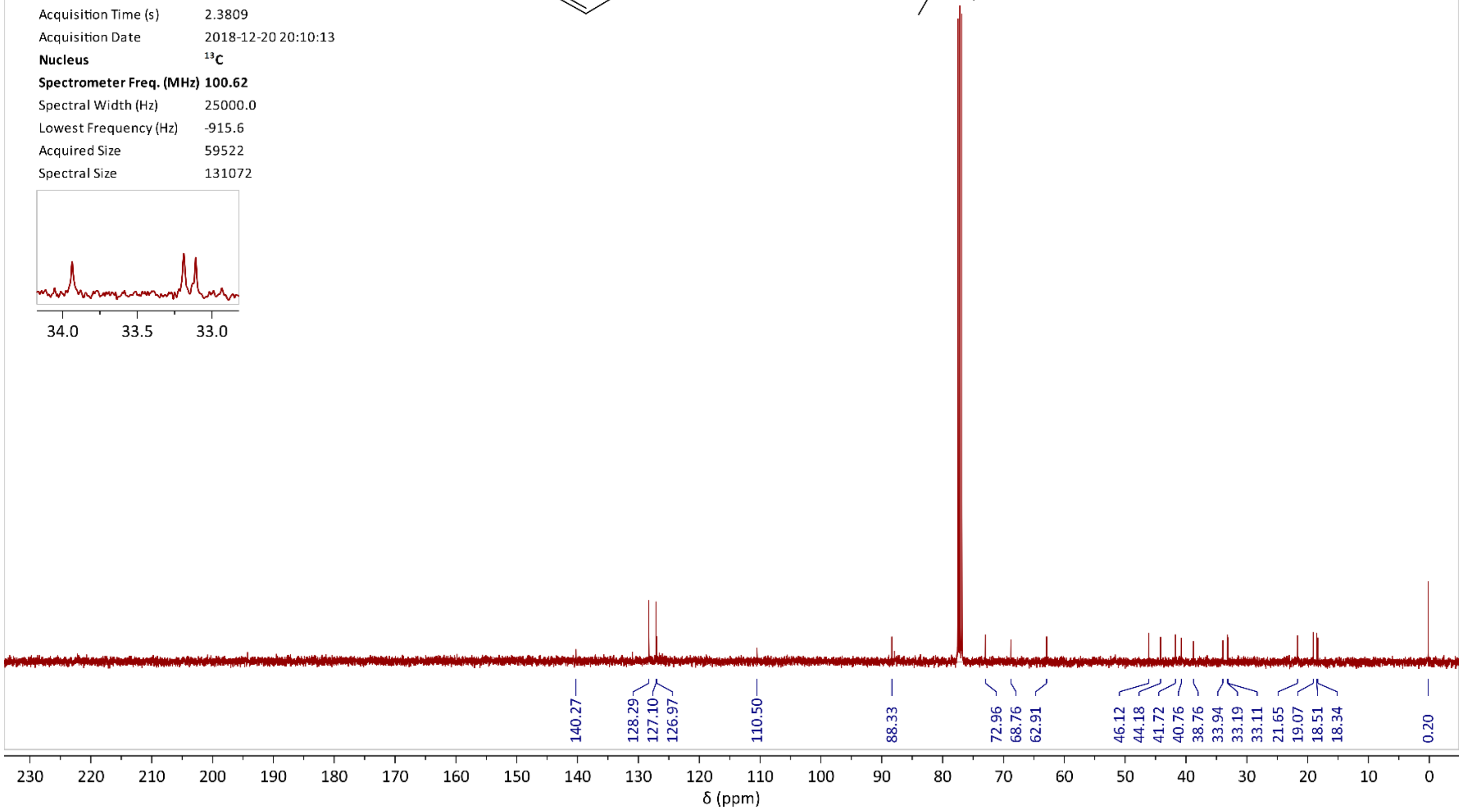

TIPS Ketone 40

^{1}H NMR Spectrum, $CDCl_3$, 400 MHz

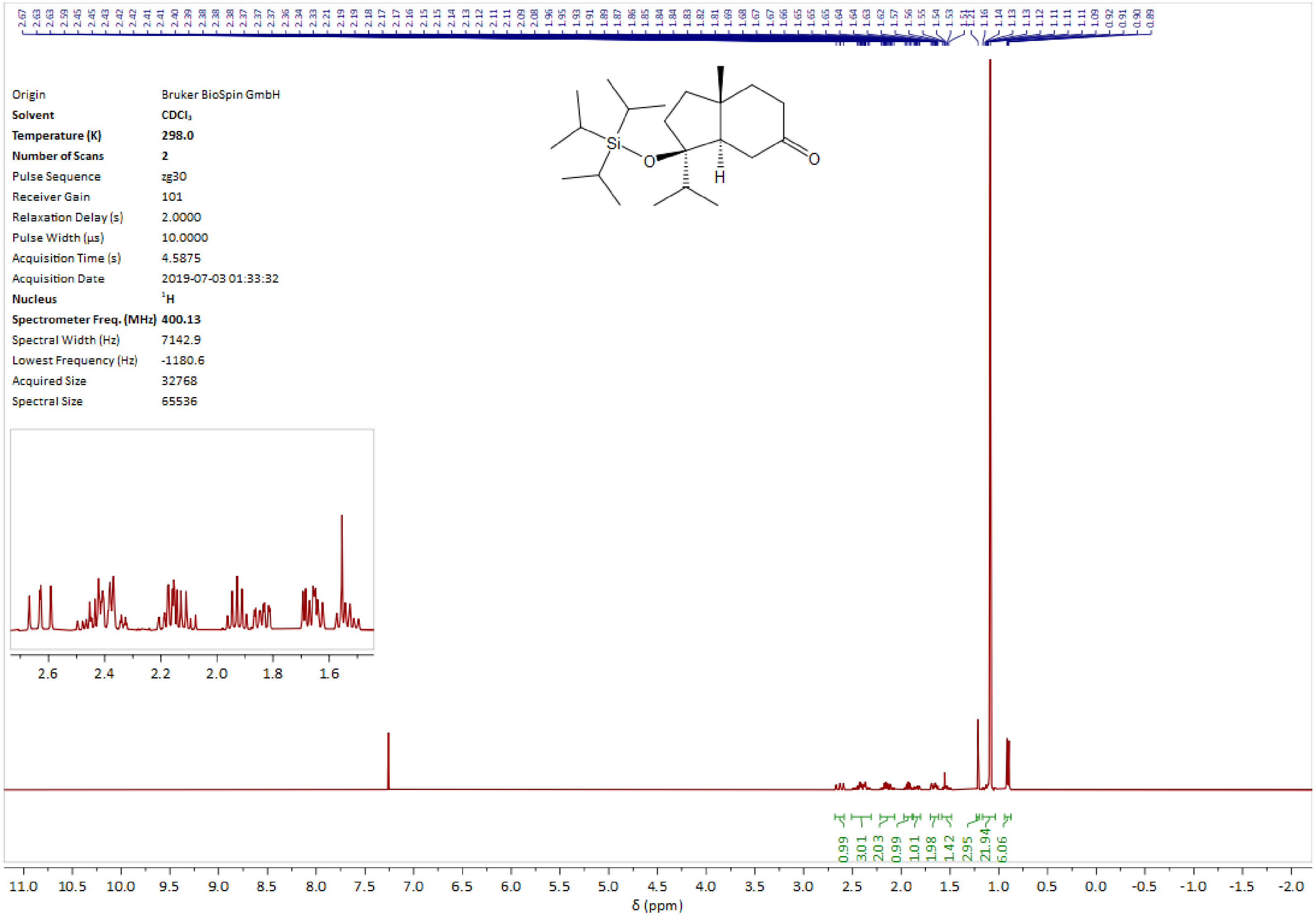

TIPS ketone 40

^{13}C NMR Spectrum, $CDCl_3$, 101 MHz

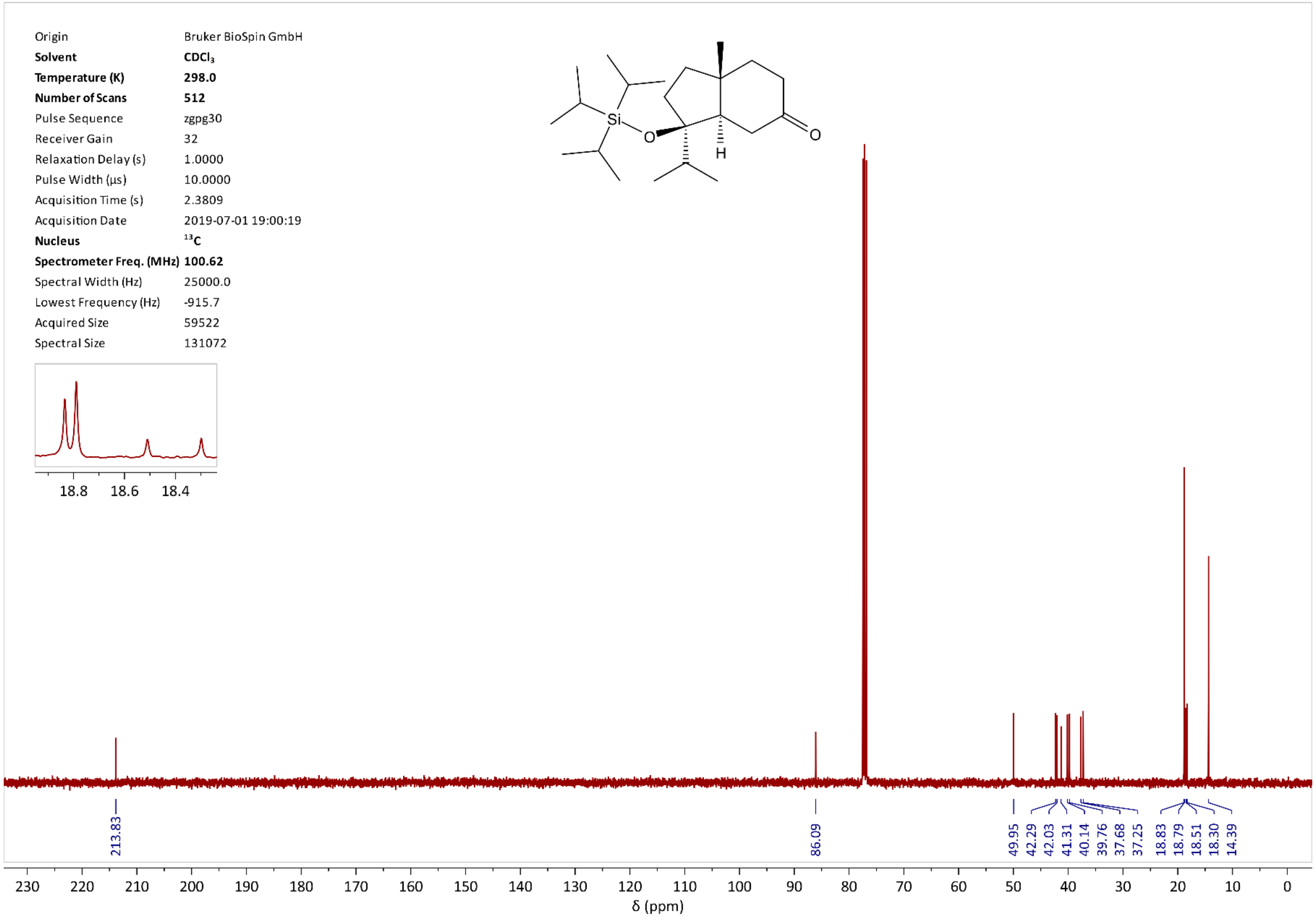

Bromoenal 41

^{1}H NMR Spectrum, $CDCl_3$, 400 MHz

Origin	Bruker BioSpin GmbH
**Solvent**	**$CDCl_3$**
**Temperature (K)**	**294.4**
**Number of Scans**	**32**
Pulse Sequence	zg30
Receiver Gain	90
Relaxation Delay (s)	1.5000
Pulse Width (µs)	11.0600
Acquisition Time (s)	1.9923
Acquisition Date	2020-01-24 09:32:00
**Nucleus**	**^{1}H**
**Spectrometer Freq. (MHz)**	**400.13**
Spectral Width (Hz)	8223.7
Lowest Frequency (Hz)	-1720.9
Acquired Size	16384
Spectral Size	65536

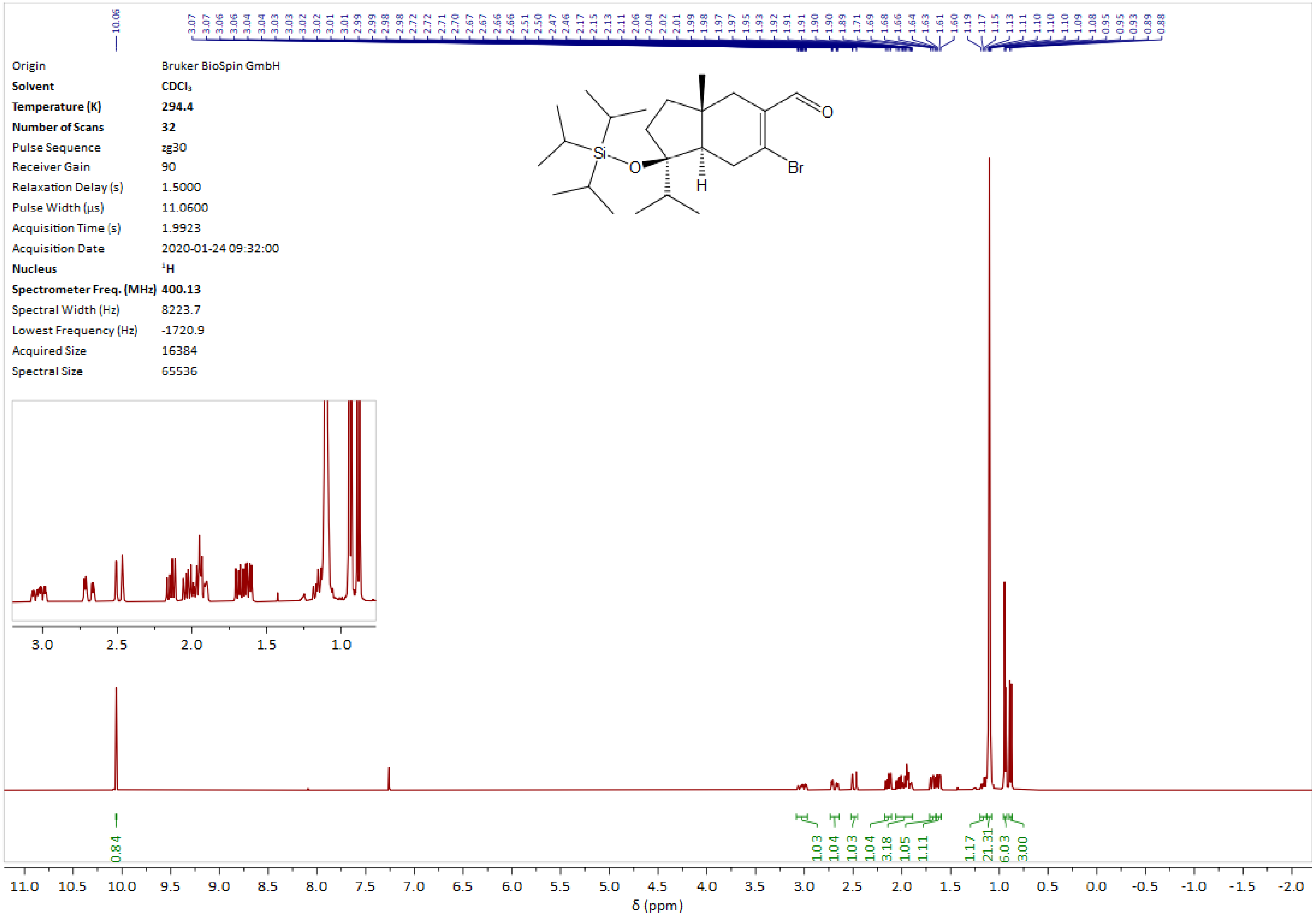

Bromoenal 41

^{13}C NMR Spectrum, $CDCl_3$, 101 MHz

Origin	Bruker BioSpin GmbH
**Solvent**	**$CDCl_3$**
**Temperature (K)**	**294.9**
**Number of Scans**	**380**
Pulse Sequence	udeft
Receiver Gain	2050
Relaxation Delay (s)	3.0000
Pulse Width (μs)	8.8000
Acquisition Time (s)	0.3599
Acquisition Date	2020-01-24 08:38:00
**Nucleus**	**^{13}C**
**Spectrometer Freq. (MHz)**	**100.62**
Spectral Width (Hz)	25252.5
Lowest Frequency (Hz)	-1128.3
Acquired Size	9089
Spectral Size	65536

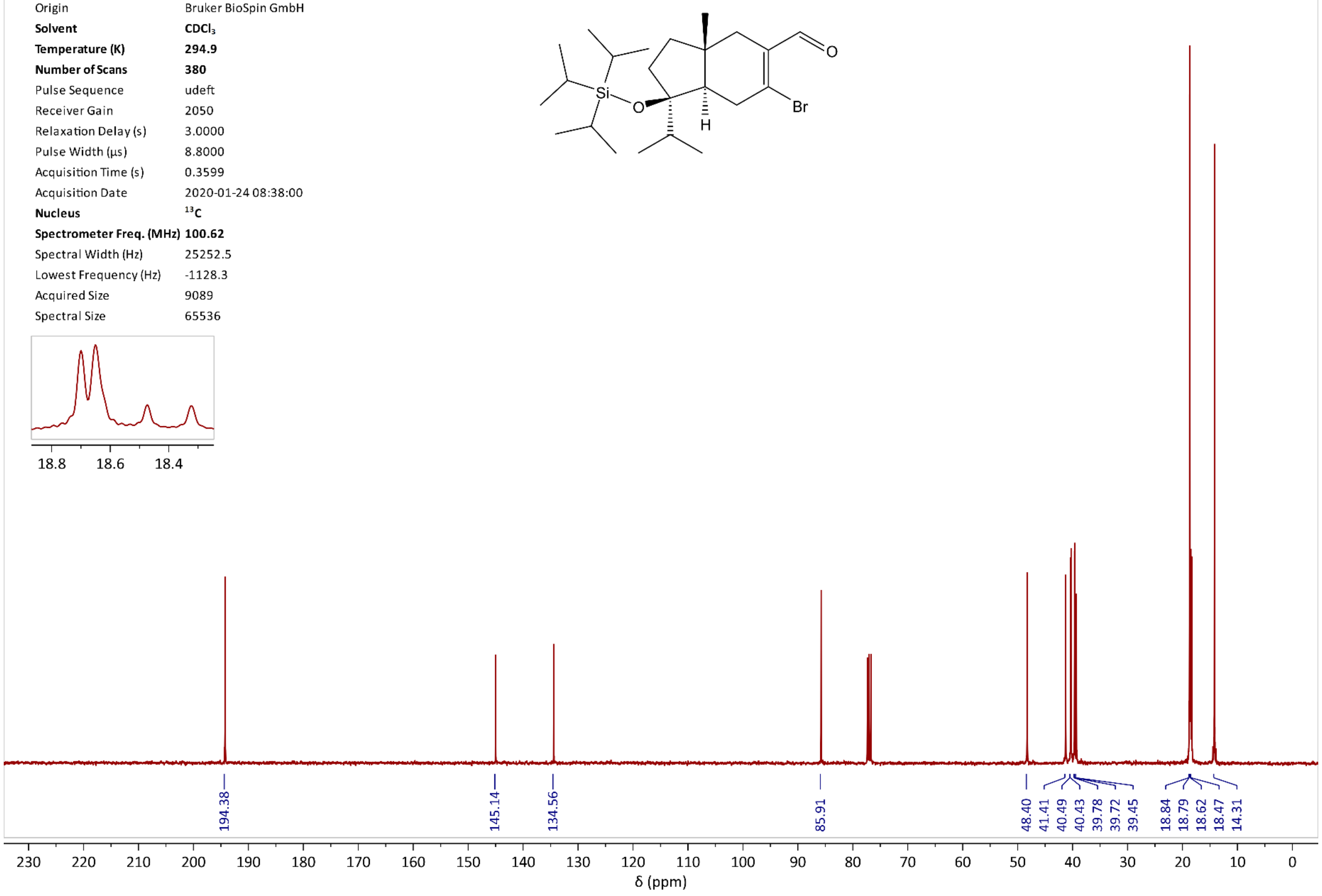

Enynol 42 – Isomer 1

^{1}H NMR Spectrum, $CDCl_3$, 400 MHz

Origin	Bruker BioSpin GmbH
**Solvent**	**$CDCl_3$**
**Temperature (K)**	**297.9**
**Number of Scans**	**4**
Pulse Sequence	zg30
Receiver Gain	86
Relaxation Delay (s)	2.0000
Pulse Width (µs)	9.4000
Acquisition Time (s)	4.5875
Acquisition Date	2020-10-14 20:40:54
**Nucleus**	**^{1}H**
**Spectrometer Freq. (MHz)**	**400.13**
Spectral Width (Hz)	7142.9
Lowest Frequency (Hz)	-1180.8
Acquired Size	32768
Spectral Size	65536

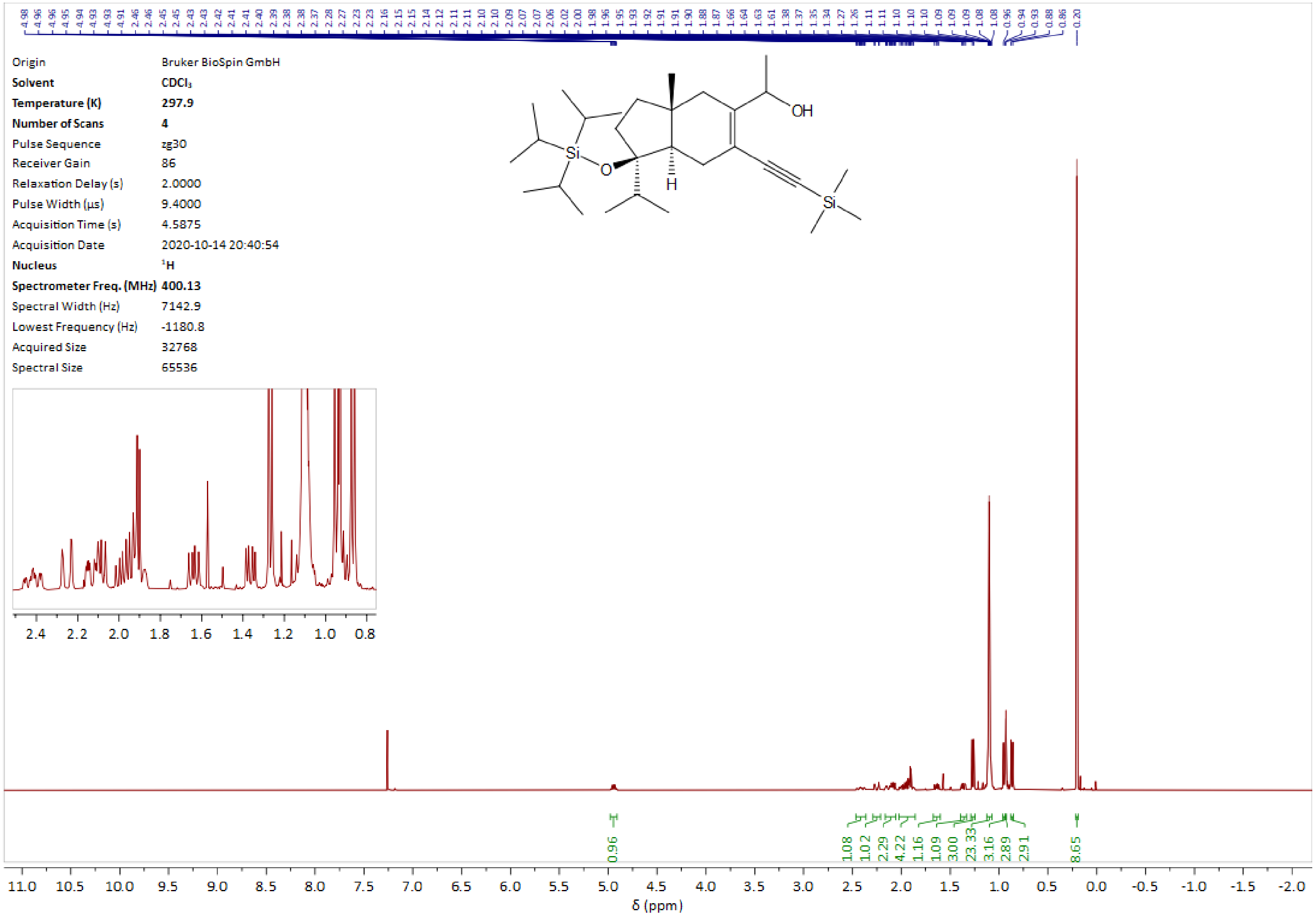

Enynol 42 – Isomer 1

^{13}C NMR Spectrum, $CDCl_3$, 101 MHz

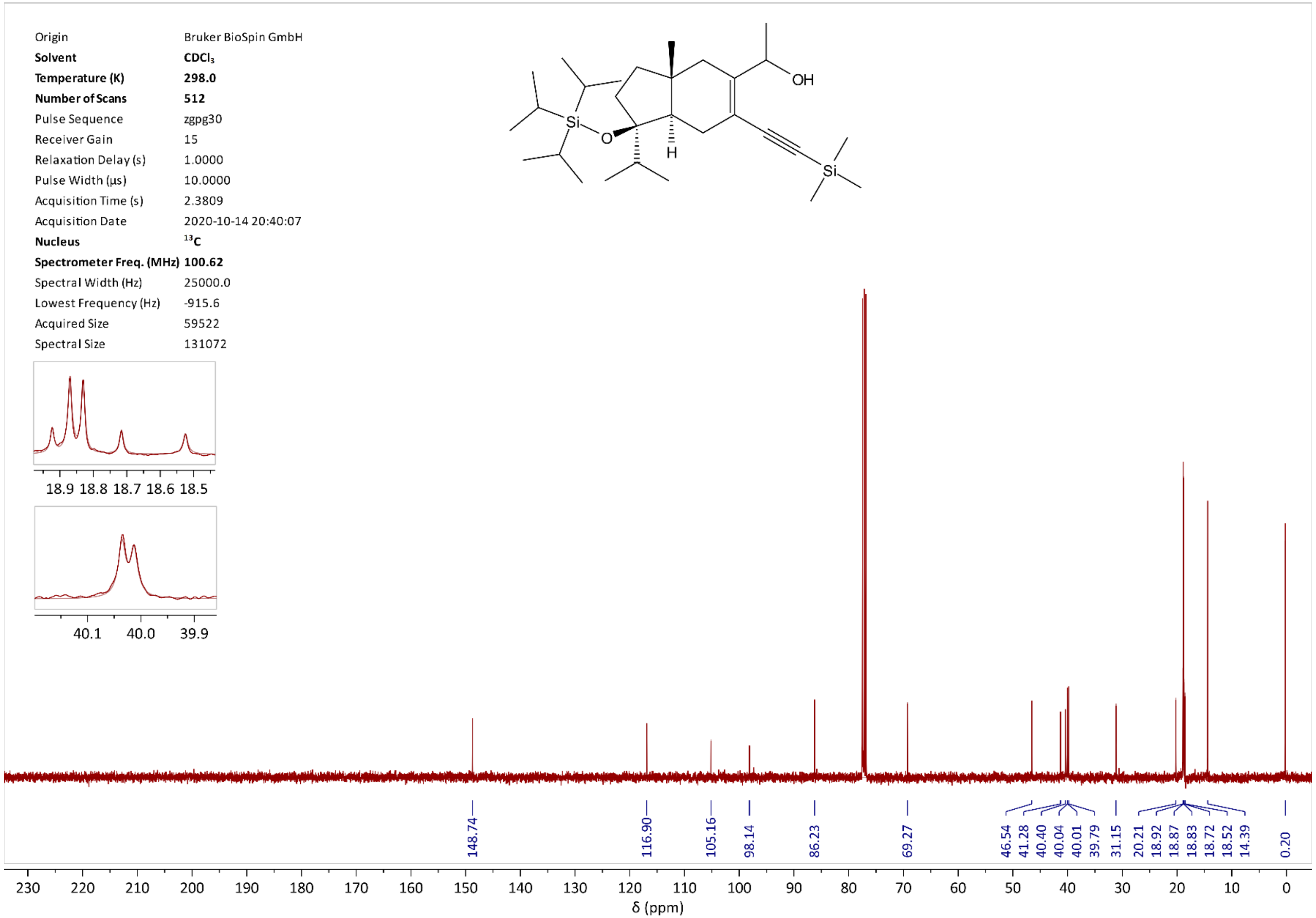

Enynol 42 – Isomer 2

^{1}H NMR Spectrum, $CDCl_3$, 400 MHz

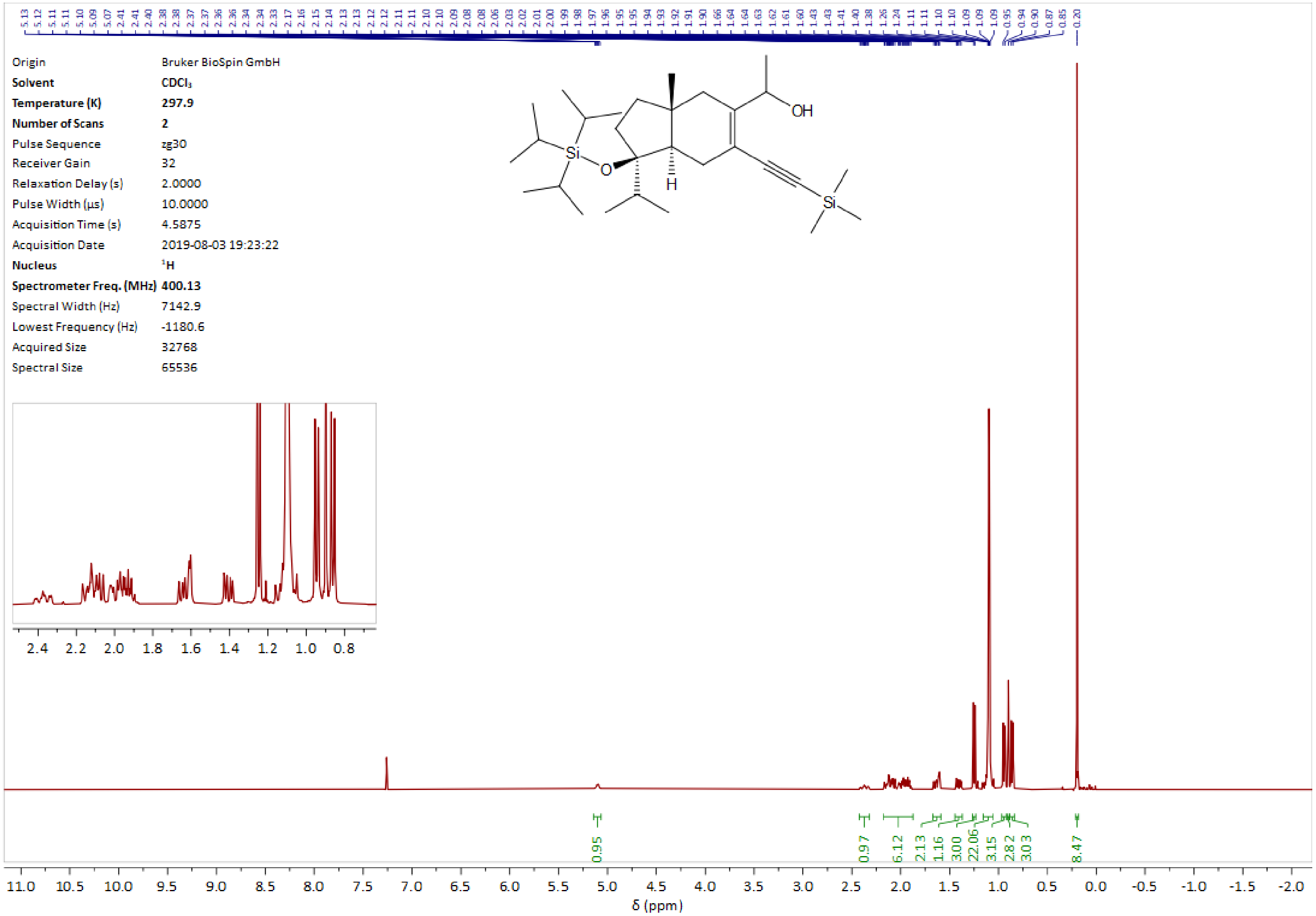

Enynol 42 – Isomer 2

^{13}C NMR Spectrum, $CDCl_3$, 101 MHz

Origin	Bruker BioSpin GmbH
**Solvent**	**$CDCl_3$**
**Temperature (K)**	**298.0**
**Number of Scans**	**512**
Pulse Sequence	zgpg30
Receiver Gain	32
Relaxation Delay (s)	1.0000
Pulse Width (µs)	10.0000
Acquisition Time (s)	2.3809
Acquisition Date	2019-08-03 19:03:09
**Nucleus**	**^{13}C**
**Spectrometer Freq. (MHz)**	**100.62**
Spectral Width (Hz)	25000.0
Lowest Frequency (Hz)	-915.9
Acquired Size	59522
Spectral Size	131072

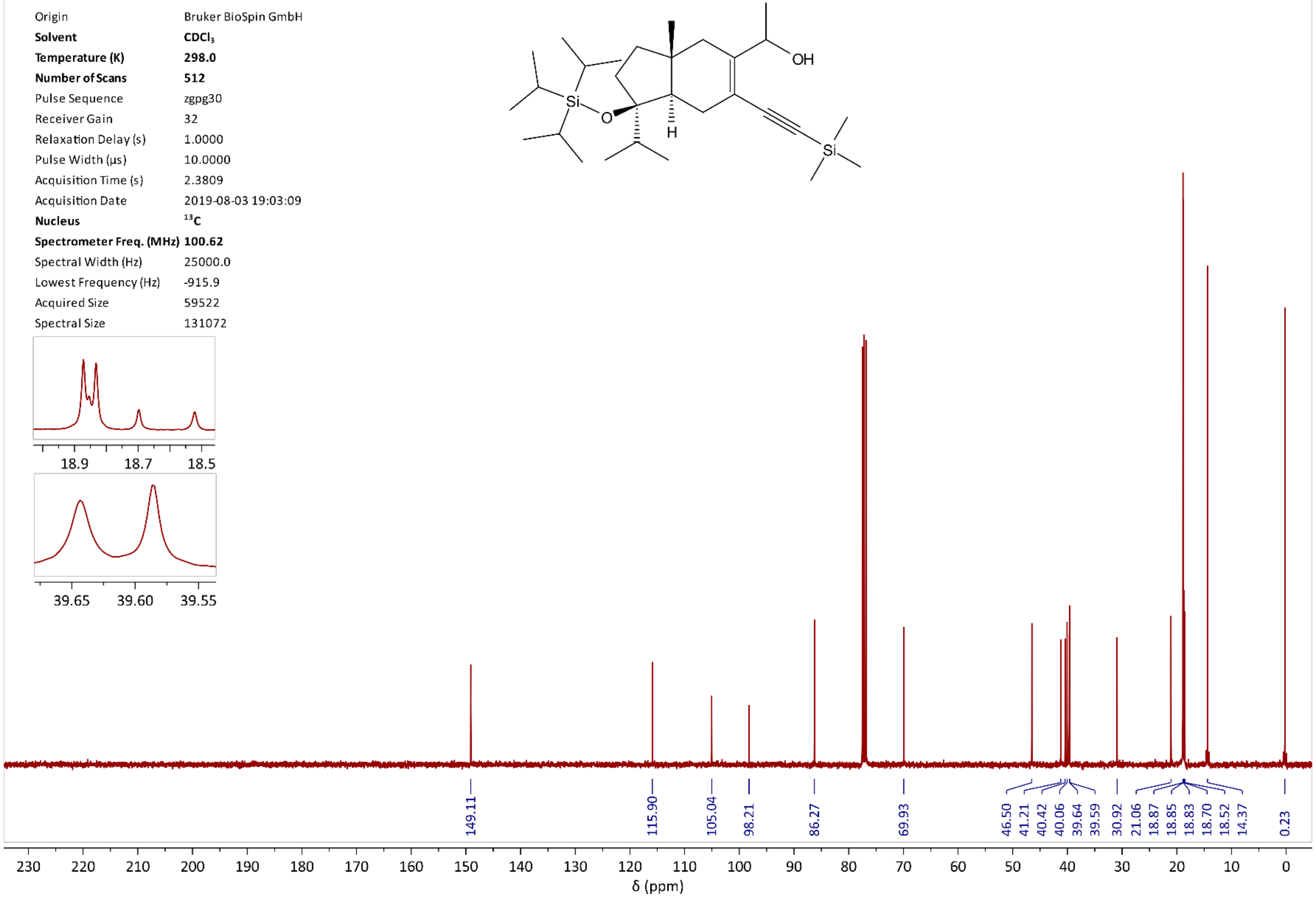

Furan 43

^1H NMR Spectrum, CD_2Cl_2, 400 MHz

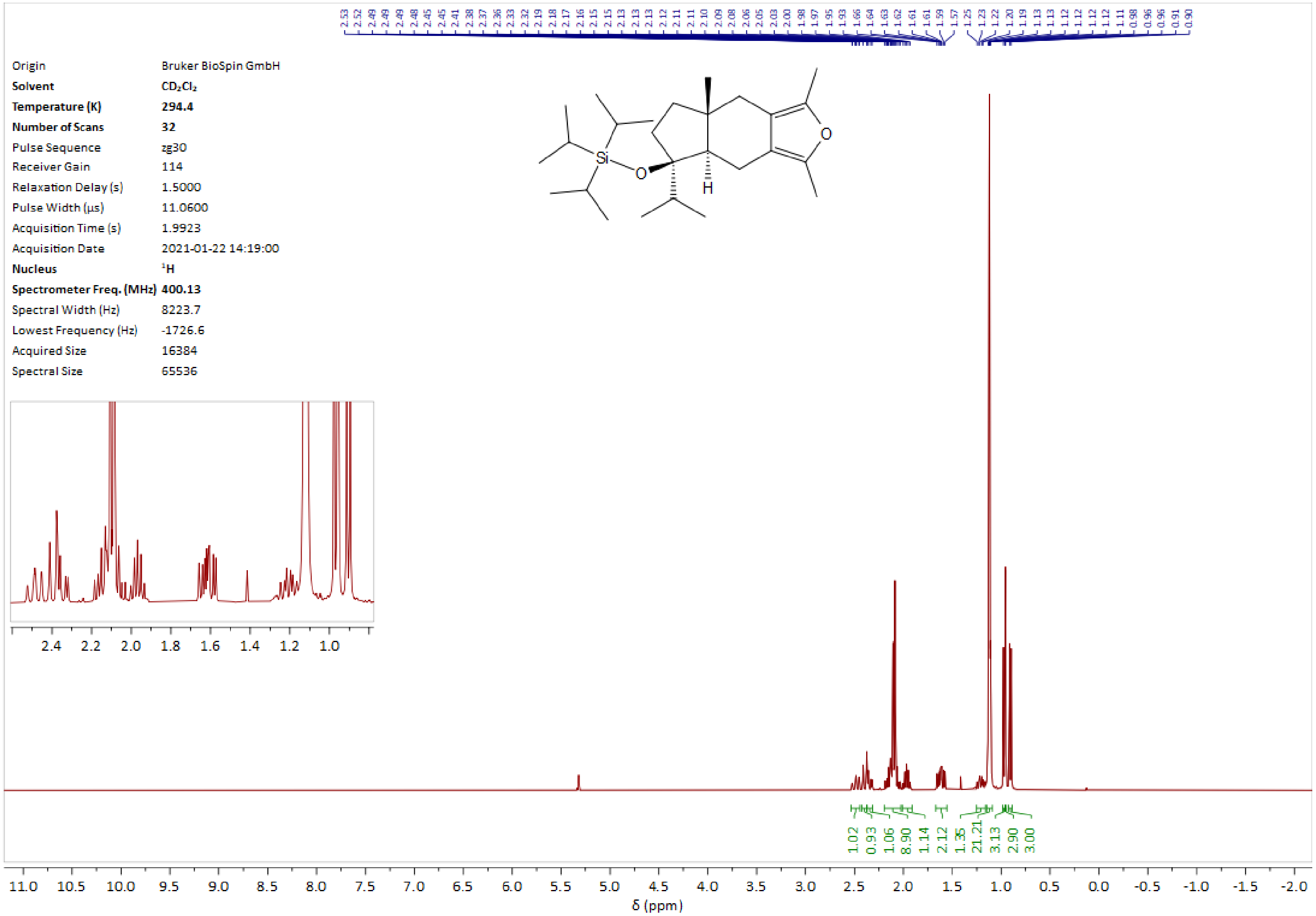

Furan 43

^{13}C NMR Spectrum, CD_2Cl_2, 101 MHz

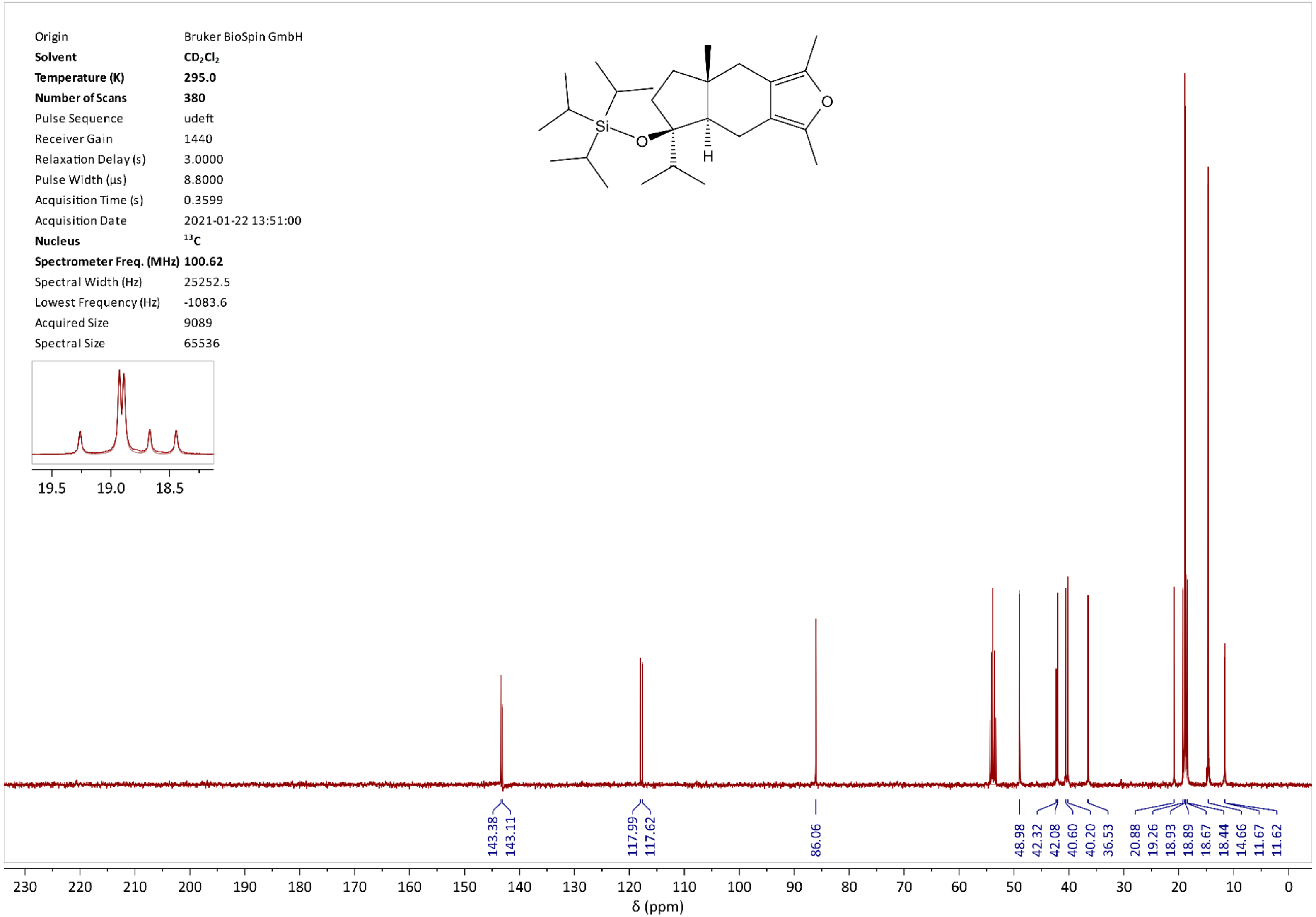

TIPS acrolein 45

^{1}H NMR Spectrum, $CDCl_3$, 400 MHz

Origin	Bruker BioSpin GmbH
**Solvent**	**$CDCl_3$**
**Temperature (K)**	**297.9**
**Number of Scans**	**2**
Pulse Sequence	zg30
Receiver Gain	101
Relaxation Delay (s)	2.0000
Pulse Width (µs)	10.0000
Acquisition Time (s)	4.5875
Acquisition Date	2019-07-26 02:29:25
**Nucleus**	**^{1}H**
**Spectrometer Freq. (MHz)**	**400.13**
Spectral Width (Hz)	7142.9
Lowest Frequency (Hz)	-1180.3
Acquired Size	32768
Spectral Size	65536

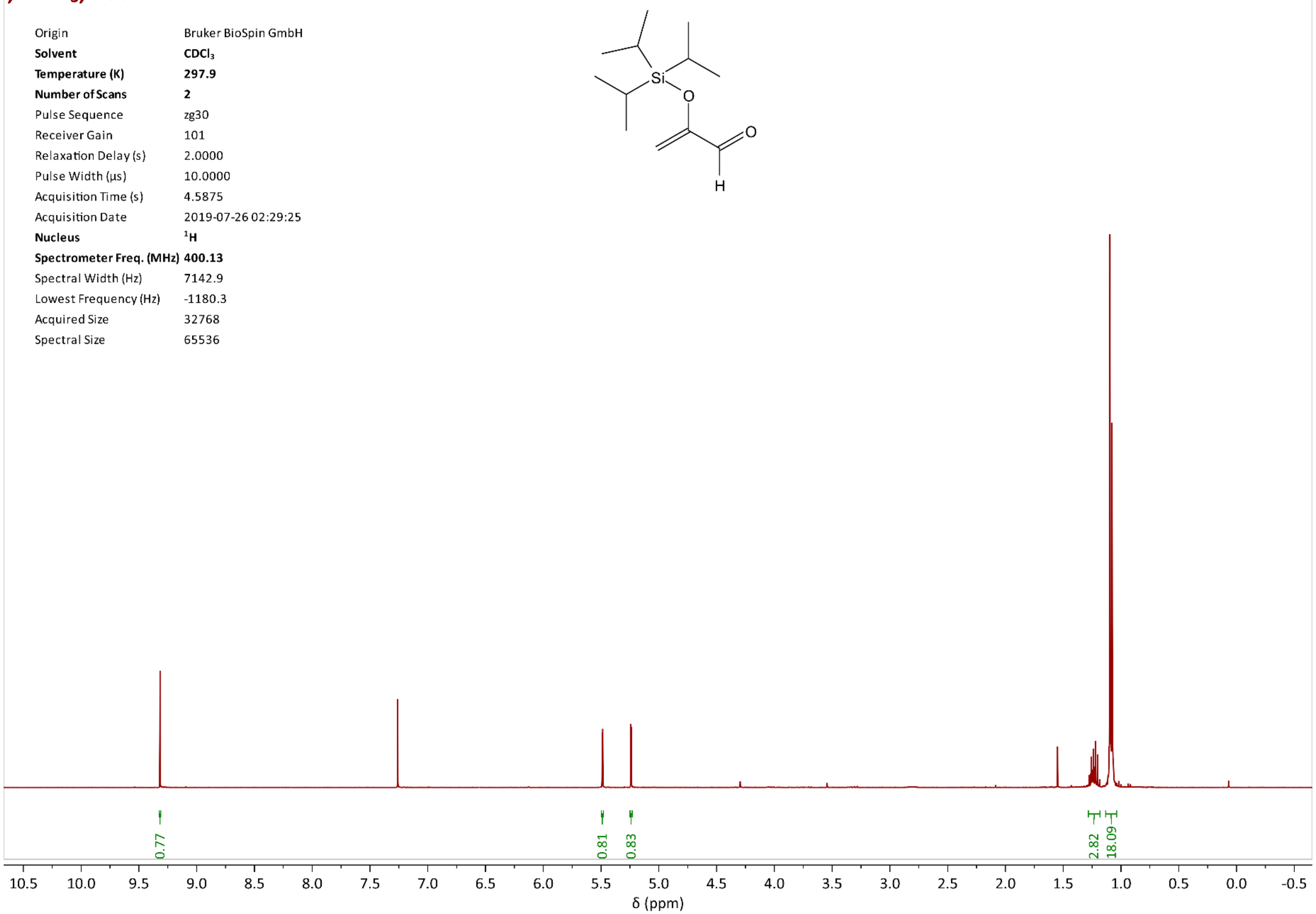

TIPS acrolein 45

^{13}C NMR Spectrum, $CDCl_3$, 101 MHz

Origin	Bruker BioSpin GmbH
**Solvent**	**$CDCl_3$**
**Temperature (K)**	**298.0**
**Number of Scans**	**512**
Pulse Sequence	zgpg30
Receiver Gain	32
Relaxation Delay (s)	1.0000
Pulse Width (µs)	10.0000
Acquisition Time (s)	2.3809
Acquisition Date	2019-07-26 02:28:16
**Nucleus**	**^{13}C**
**Spectrometer Freq. (MHz)**	**100.62**
Spectral Width (Hz)	25000.0
Lowest Frequency (Hz)	-915.3
Acquired Size	59522
Spectral Size	131072

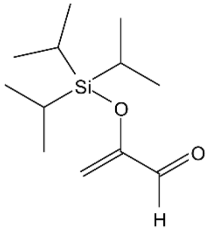

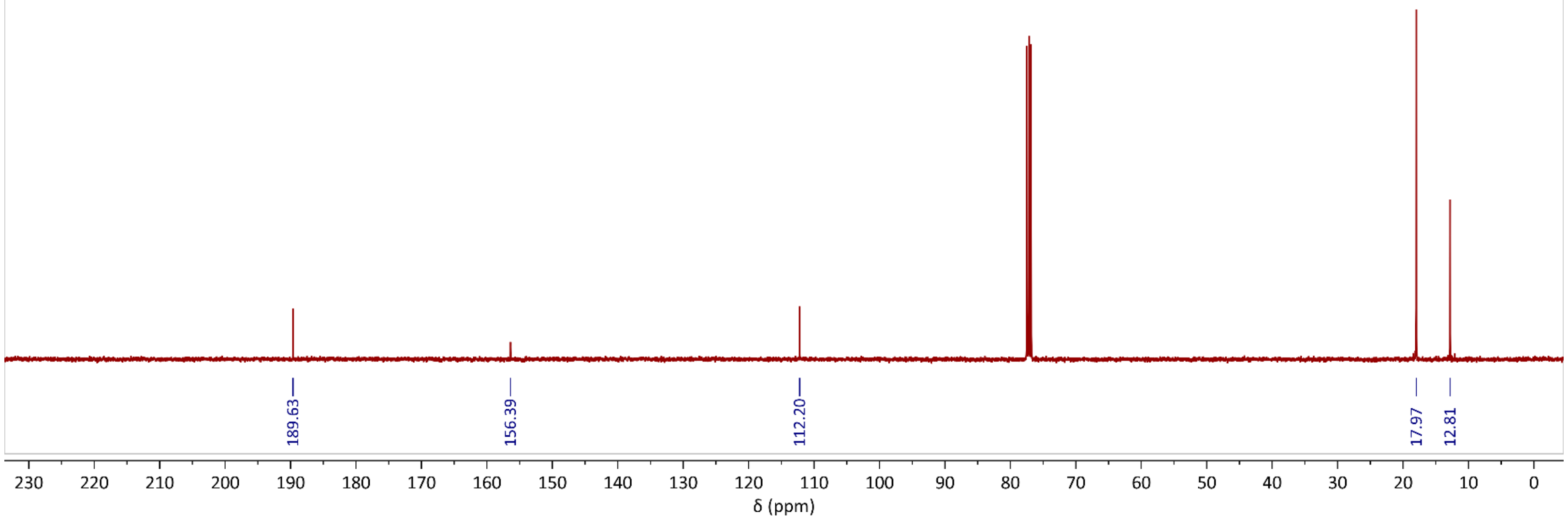

Oxabicyclic ketones 46 and 47

^{1}H NMR Spectrum, $CDCl_3$, 500 MHz

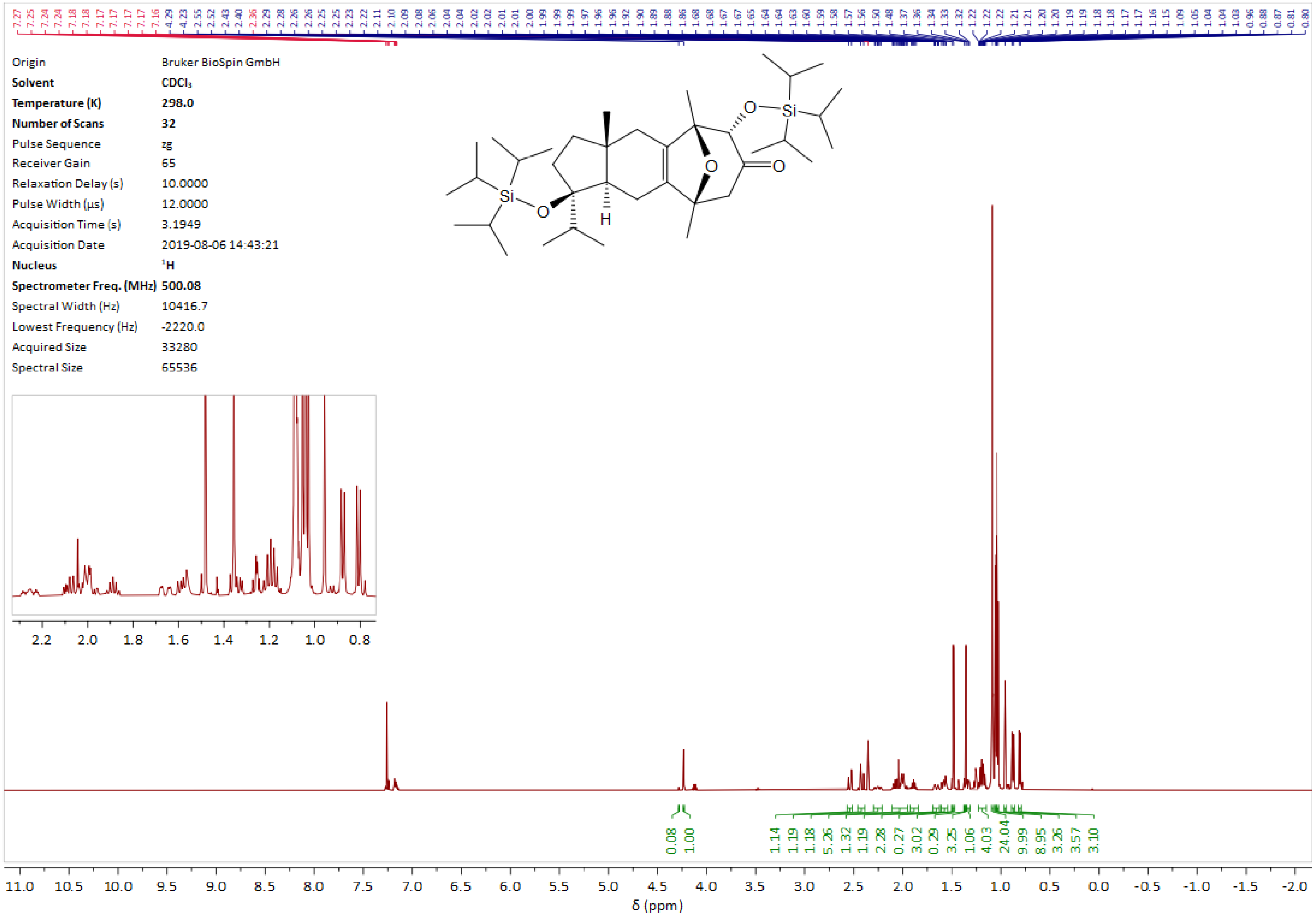

Oxabicyclic ketones 46 and 47

^{13}C NMR Spectrum, $CDCl_3$, 126 MHz

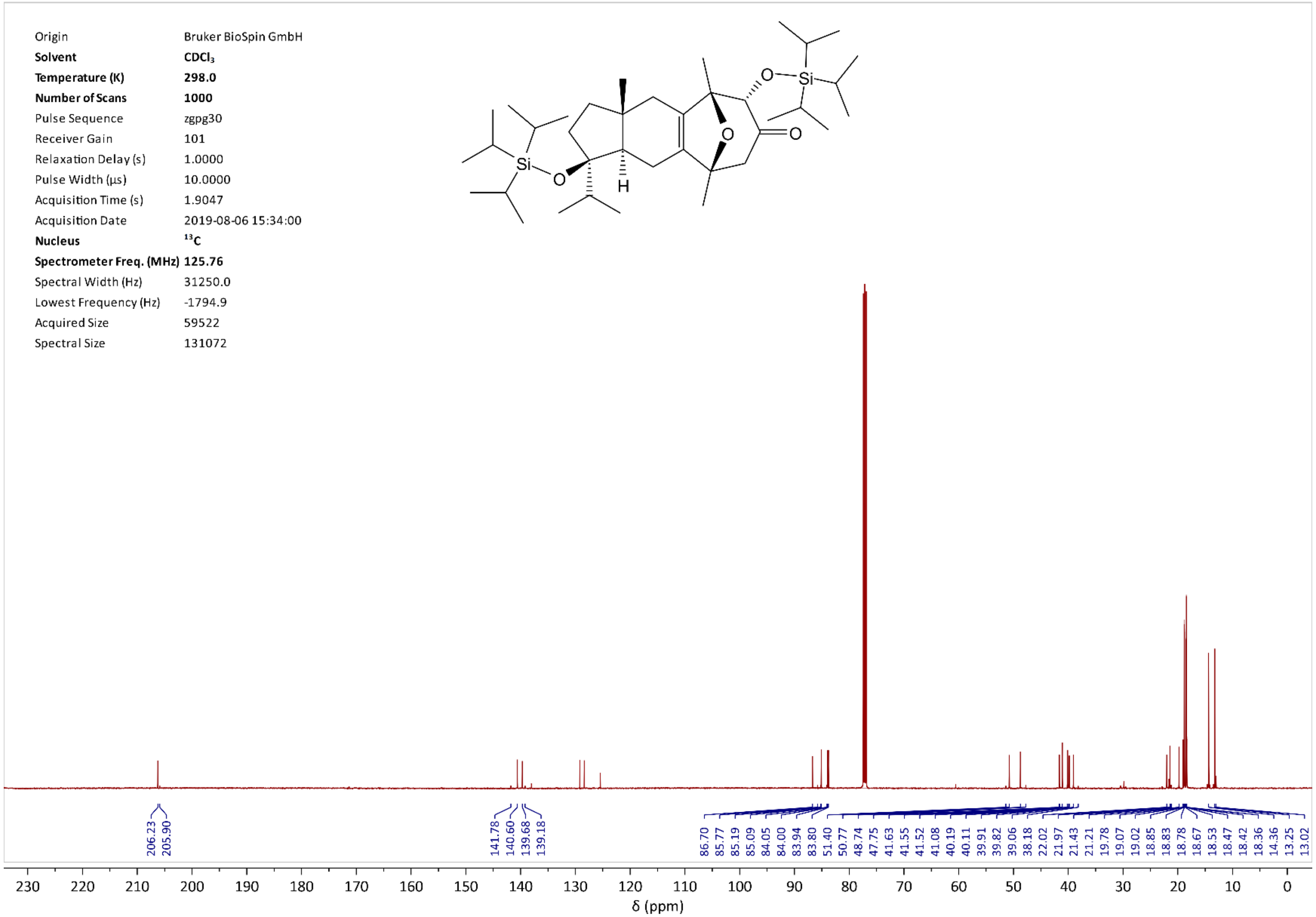

Oxabicyclic ketones 46 and 47

HSQC Spectrum, $CDCl_3$, 500 MHz/126 MHz

Origin	Bruker BioSpin GmbH
**Solvent**	**$CDCl_3$**
**Temperature (K)**	**298.0**
**Number of Scans**	**4**
Pulse Sequence	hsqcedetgpsp.3
Receiver Gain	101
Relaxation Delay (s)	1.0000
Pulse Width (µs)	12.0000
Acquisition Time (s)	0.0870
Acquisition Date	2019-08-06 16:22:11
**Spectrometer Freq. (MHz)**	**(500.08, 125.75)**
Spectral Width	(5882.4, 20746.9)
Lowest Frequency	(-601.5, -944.8)
**Nucleus**	**(^{1}H, ^{13}C)**
Acquired Size	(512, 256)
Spectral Size	(2048, 2048)

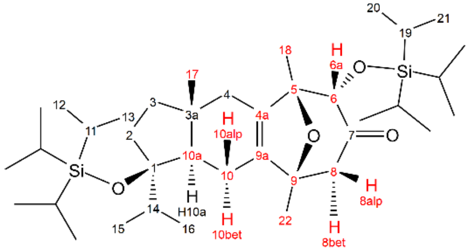

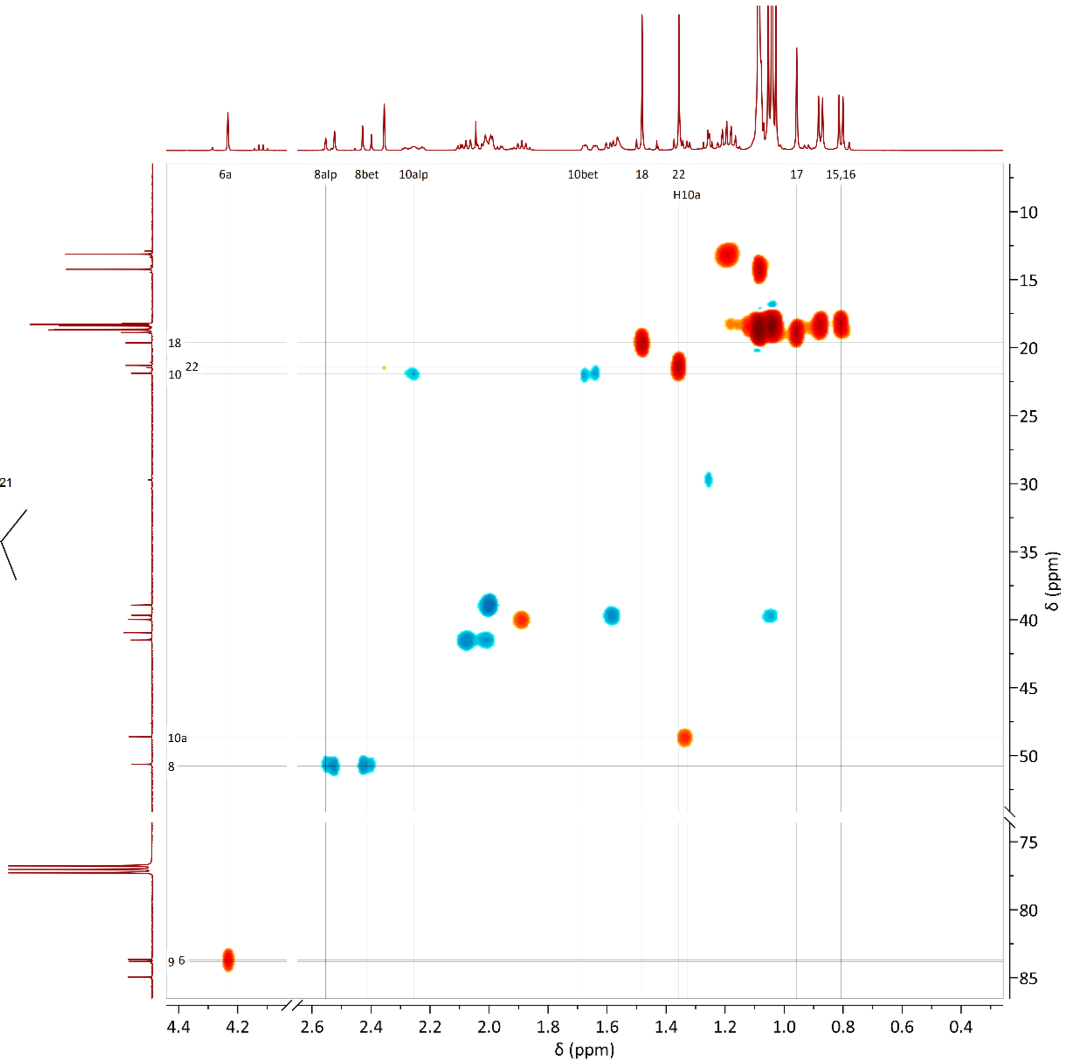

2D correlation maps have been included to help justify the absolute structural assignment of this structure. Not all correlations have been included, only those that help to analyse the absolute structure.

Oxabicyclic ketones 46 and 47

HMBC Spectrum, $CDCl_3$, 500 MHz/126 MHz

Origin	Bruker BioSpin GmbH
**Solvent**	**$CDCl_3$**
**Temperature (K)**	**298.0**
**Number of Scans**	**8**
Pulse Sequence	hmbcetgpl2nd.2
Receiver Gain	101
Relaxation Delay (s)	1.5000
Pulse Width (μs)	12.0000
Acquisition Time (s)	0.3113
Acquisition Date	2019-08-06 18:32:31
**Spectrometer Freq. (MHz)**	**(500.08, 125.76)**
Spectral Width	(6578.9, 30177.3)
Lowest Frequency	(-151.1, -1258.6)
**Nucleus**	**(^{1}H, ^{13}C)**
Acquired Size	(2048, 512)
Spectral Size	(2048, 2048)

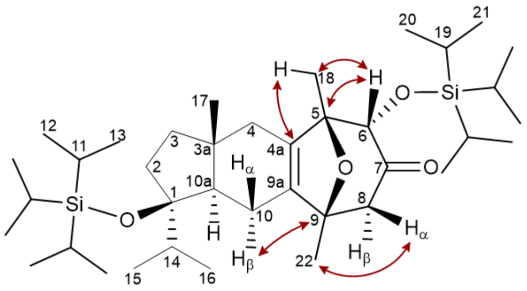

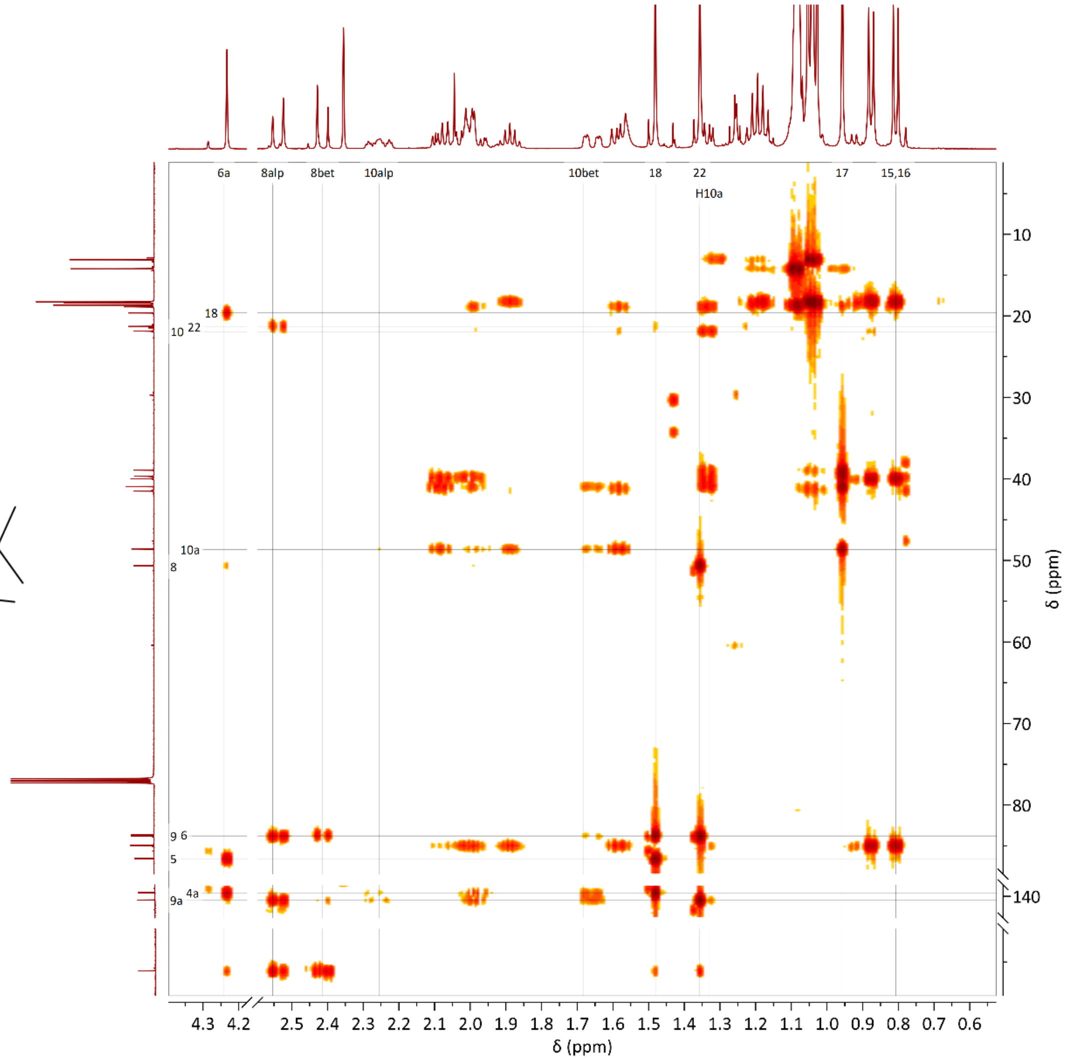

Oxabicyclic ketones 46 and 47

NOESY Spectrum, $CDCl_3$, 500 MHz

Origin	Bruker BioSpin GmbH
**Solvent**	**$CDCl_3$**
**Temperature (K)**	**298.0**
**Number of Scans**	**12**
Pulse Sequence	noesygpphpp
Receiver Gain	65
Relaxation Delay (s)	1.6309
Pulse Width (μs)	12.0000
Acquisition Time (s)	0.2580
Acquisition Date	2019-08-06 22:19:57
**Spectrometer Freq. (MHz)**	**(500.07, 500.07)**
Spectral Width	(3968.3, 3968.3)
Lowest Frequency	(-27.8, -27.8)
**Nucleus**	**(^1H, ^1H)**
Acquired Size	(1024, 440)
Spectral Size	(2048, 2048)

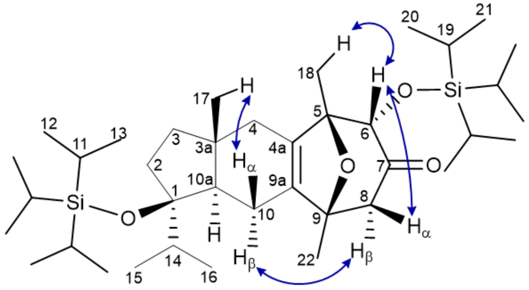

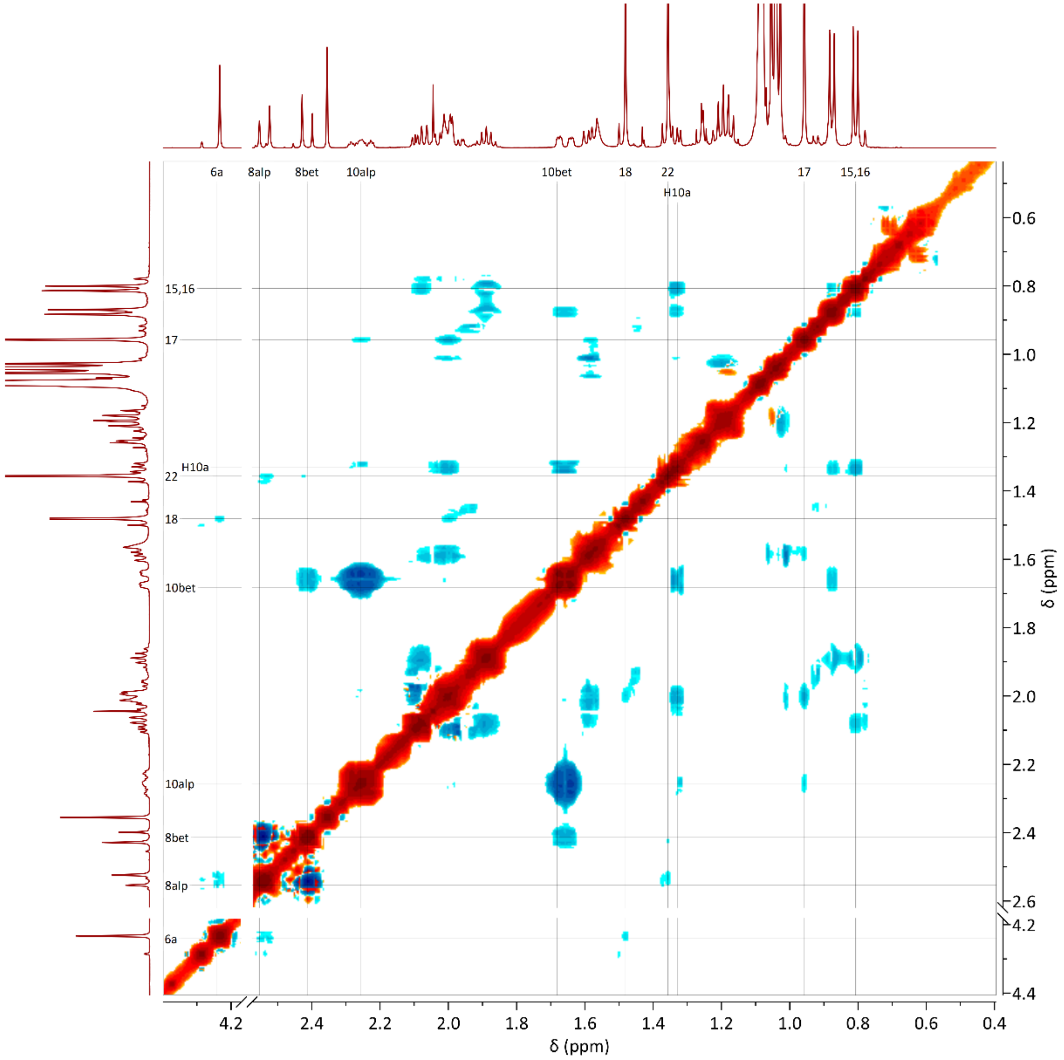

Oxabicyclic ketone 48

^{1}H NMR Spectrum, $CDCl_3$, 400 MHz

Origin	Bruker BioSpin GmbH
**Solvent**	**$CDCl_3$**
**Temperature (K)**	**298.0**
**Number of Scans**	**2**
Pulse Sequence	zg30
Receiver Gain	32
Relaxation Delay (s)	2.0000
Pulse Width (μs)	10.0000
Acquisition Time (s)	4.5875
Acquisition Date	2019-08-21 15:25:46
**Nucleus**	**^{1}H**
**Spectrometer Freq. (MHz)**	**400.13**
Spectral Width (Hz)	7142.9
Lowest Frequency (Hz)	-1206.7
Acquired Size	32768
Spectral Size	65536

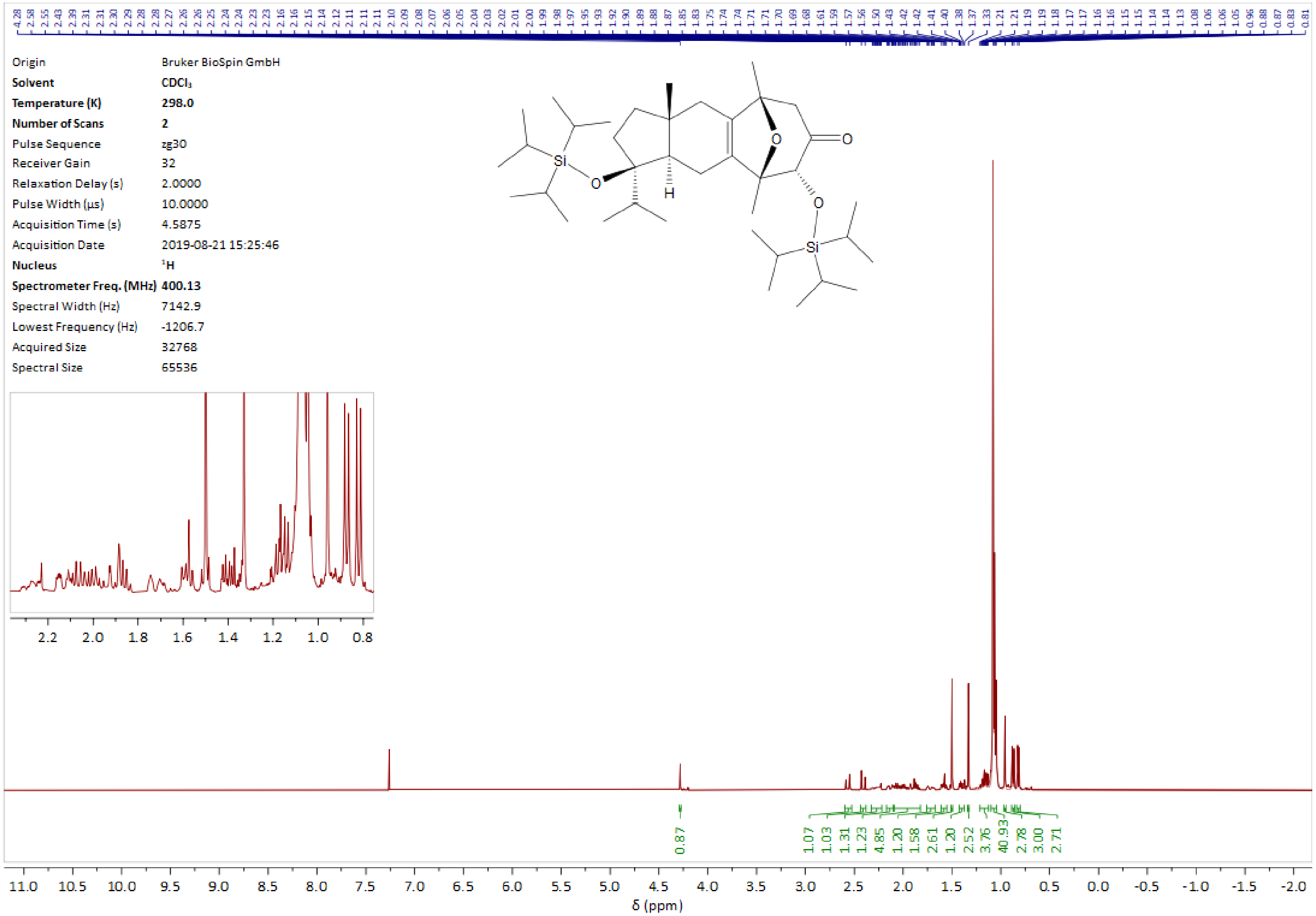

Oxabicyclic ketone 48

^{13}C NMR Spectrum, $CDCl_3$, 101 MHz

Origin	Bruker BioSpin GmbH
**Solvent**	**$CDCl_3$**
**Temperature (K)**	**298.0**
**Number of Scans**	**512**
Pulse Sequence	zgpg30
Receiver Gain	32
Relaxation Delay (s)	1.0000
Pulse Width (µs)	10.0000
Acquisition Time (s)	2.3809
Acquisition Date	2019-08-21 19:45:09
**Nucleus**	**^{13}C**
**Spectrometer Freq. (MHz)**	**100.62**
Spectral Width (Hz)	25000.0
Lowest Frequency (Hz)	-915.7
Acquired Size	59522
Spectral Size	131072

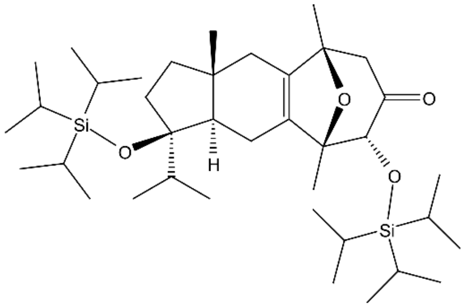

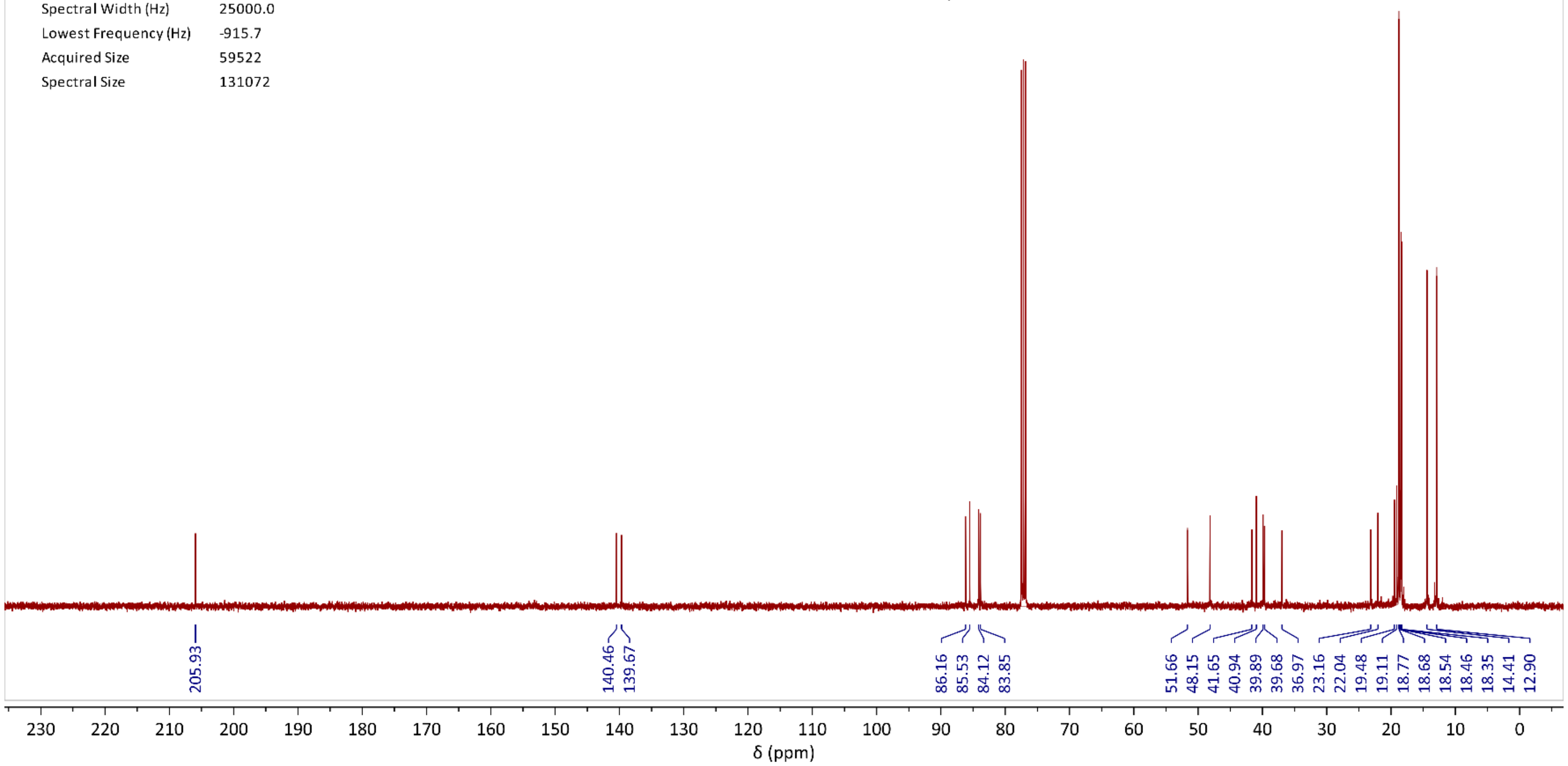

Oxabicyclic ketone 48

HSQC Spectrum, CDCl$_3$, 400 MHz/101 MHz

Origin	Bruker BioSpin GmbH
**Solvent**	**CDCl$_3$**
**Temperature (K)**	**298.3**
**Number of Scans**	**4**
Pulse Sequence	hsqcedetgpsp.3
Receiver Gain	39
Relaxation Delay (s)	1.0000
Pulse Width (µs)	10.0000
Acquisition Time (s)	0.2109
Acquisition Date	2019-08-21 20:38:12
**Spectrometer Freq. (MHz)**	**(400.13, 100.62)**
Spectral Width	(4854.4, 16501.7)
Lowest Frequency	(-636.2, -704.9)
**Nucleus**	**(^{1}H, ^{13}C)**
Acquired Size	(1024, 256)
Spectral Size	(2048, 2048)

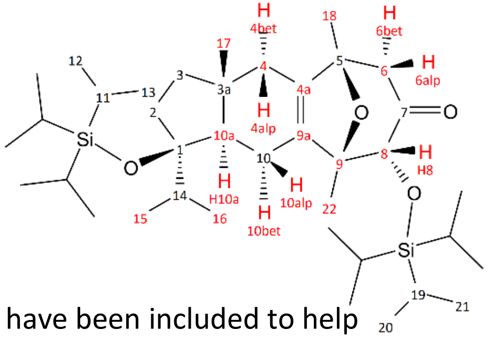

2D correlation maps have been included to help justify the absolute structural assignment of this structure. Not all correlations have been included, only those that help to analyse the absolute structure.

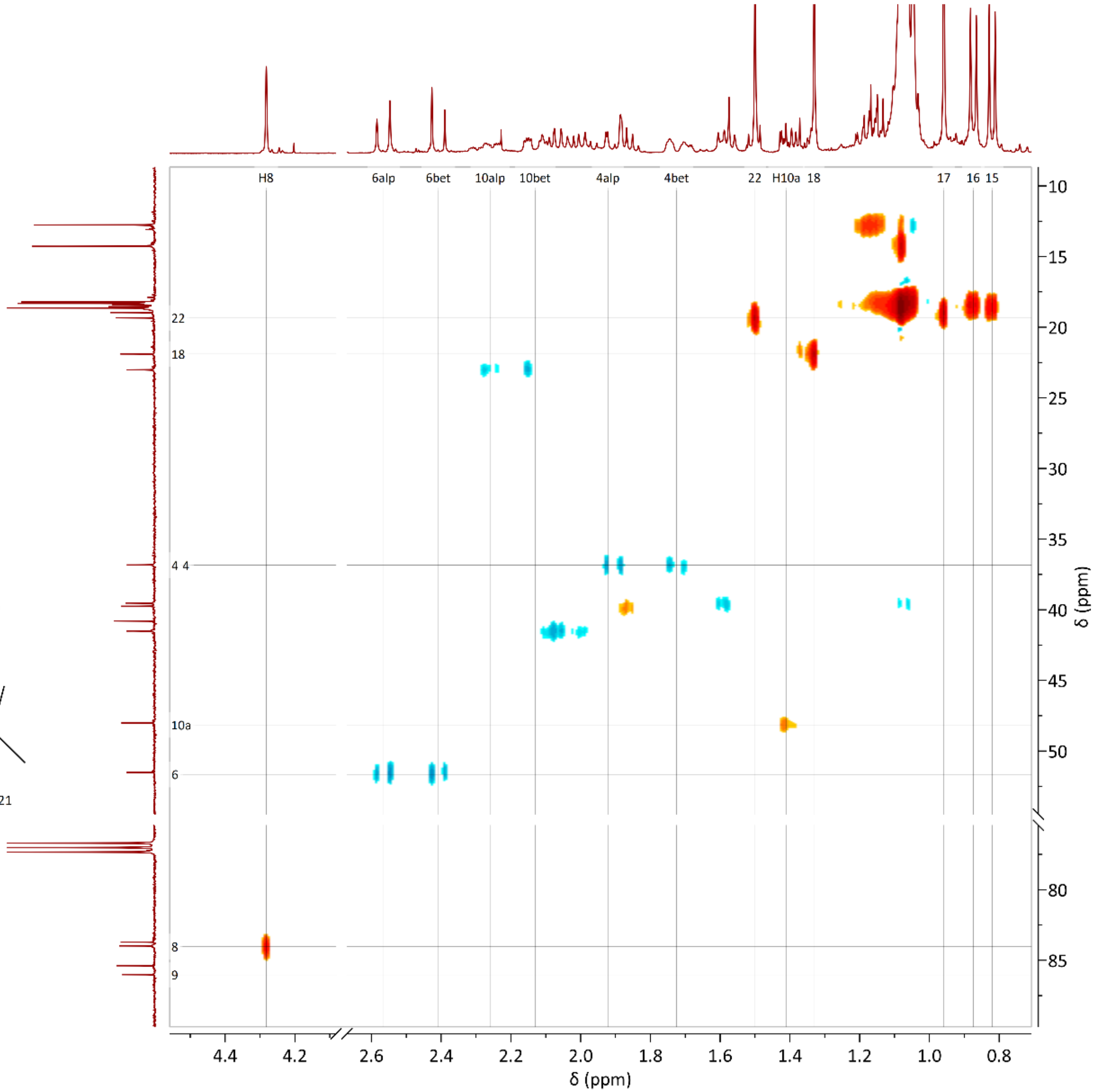

Oxabicyclic ketone 48

HMBC Spectrum, $CDCl_3$, 400 MHz/101 MHz

Origin	Bruker BioSpin GmbH
**Solvent**	**$CDCl_3$**
**Temperature (K)**	**297.9**
**Number of Scans**	**4**
Pulse Sequence	hmbcetgpl2nd.2
Receiver Gain	101
Relaxation Delay (s)	1.0000
Pulse Width (μs)	10.0000
Acquisition Time (s)	0.3686
Acquisition Date	2019-08-21 21:28:37
**Spectrometer Freq. (MHz)**	**(400.13, 100.62)**
Spectral Width	(5555.6, 25000.0)
Lowest Frequency	(-986.8, -929.5)
**Nucleus**	**(^{1}H, ^{13}C)**
Acquired Size	(2048, 512)
Spectral Size	(2048, 2048)

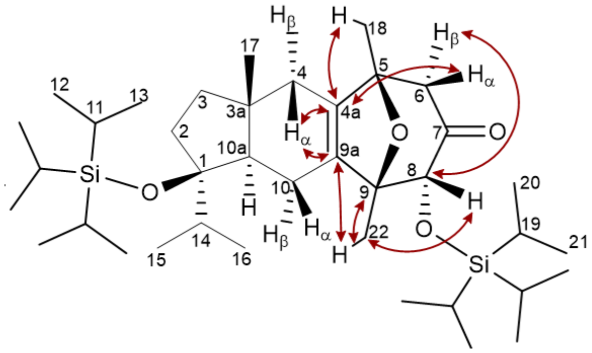

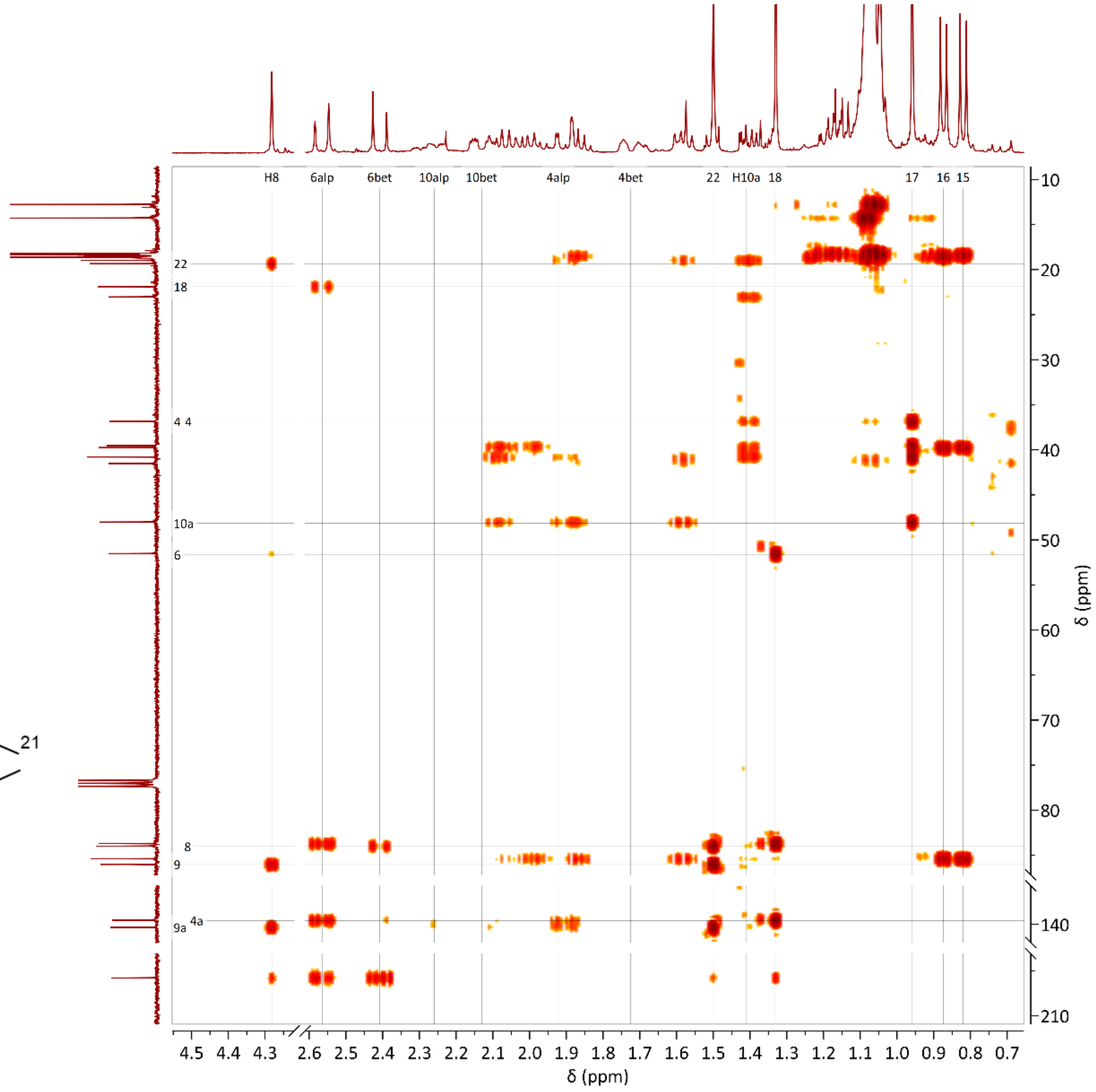

Oxabicyclic ketone 48

NOESY Spectrum, $CDCl_3$, 400 MHz

Origin	Bruker BioSpin GmbH
**Solvent**	**$CDCl_3$**
**Temperature (K)**	**298.0**
**Number of Scans**	**6**
Pulse Sequence	noesygpphpp
Receiver Gain	28
Relaxation Delay (s)	0.7791
Pulse Width (µs)	10.0000
Acquisition Time (s)	0.5530
Acquisition Date	2019-08-21 22:30:05
**Spectrometer Freq. (MHz)**	**(400.13, 400.13)**
Spectral Width	(3703.7, 1785.7)
Lowest Frequency	(-856.1, 102.9)
**Nucleus**	**(^{1}H, ^{1}H)**
Acquired Size	(2048, 300)
Spectral Size	(2048, 2048)

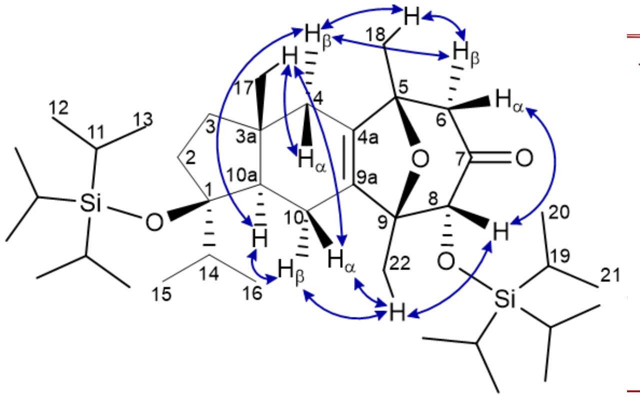

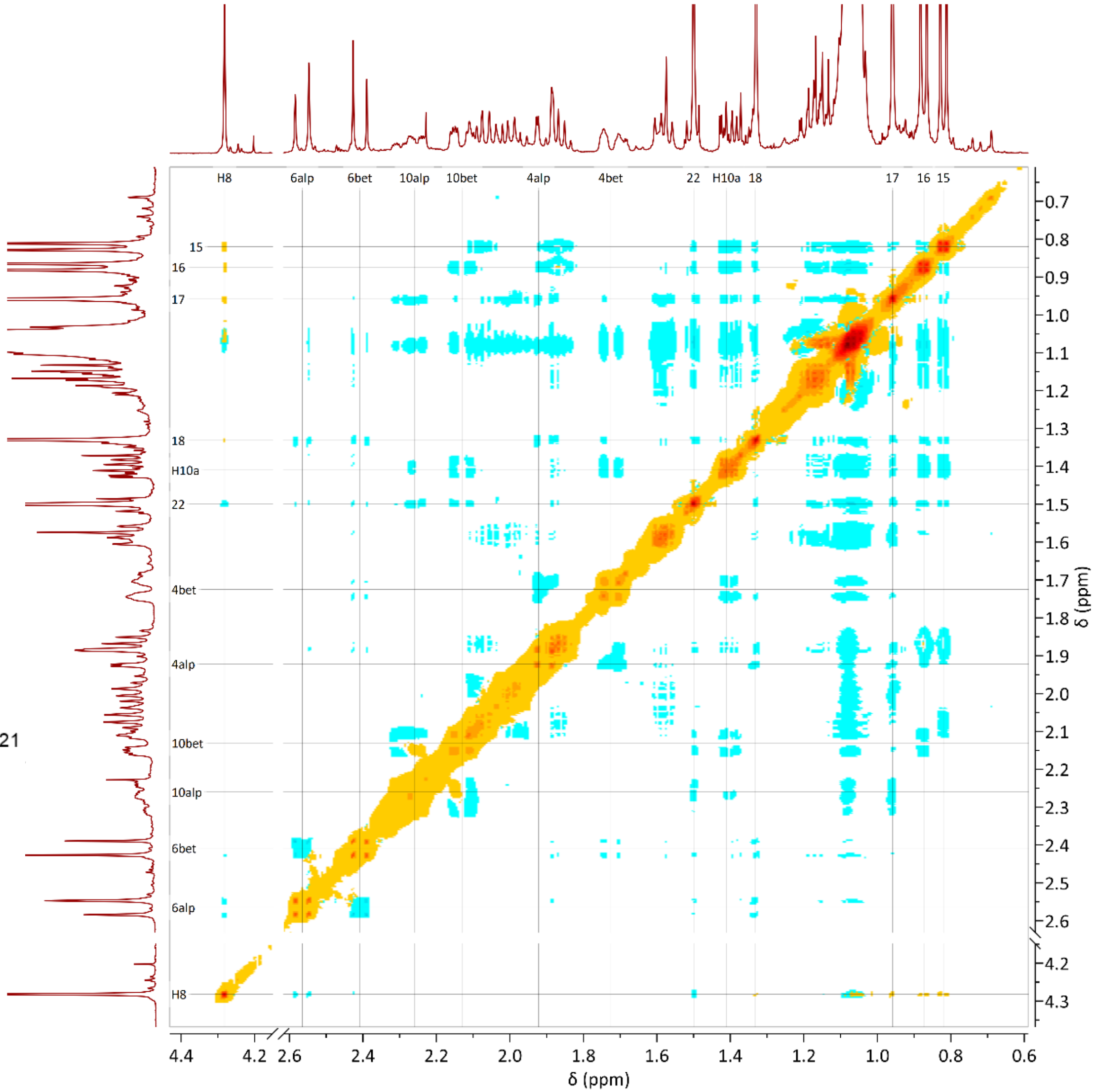

Oxabicyclic ketone 49

^{1}H NMR Spectrum, $CDCl_3$, 500 MHz

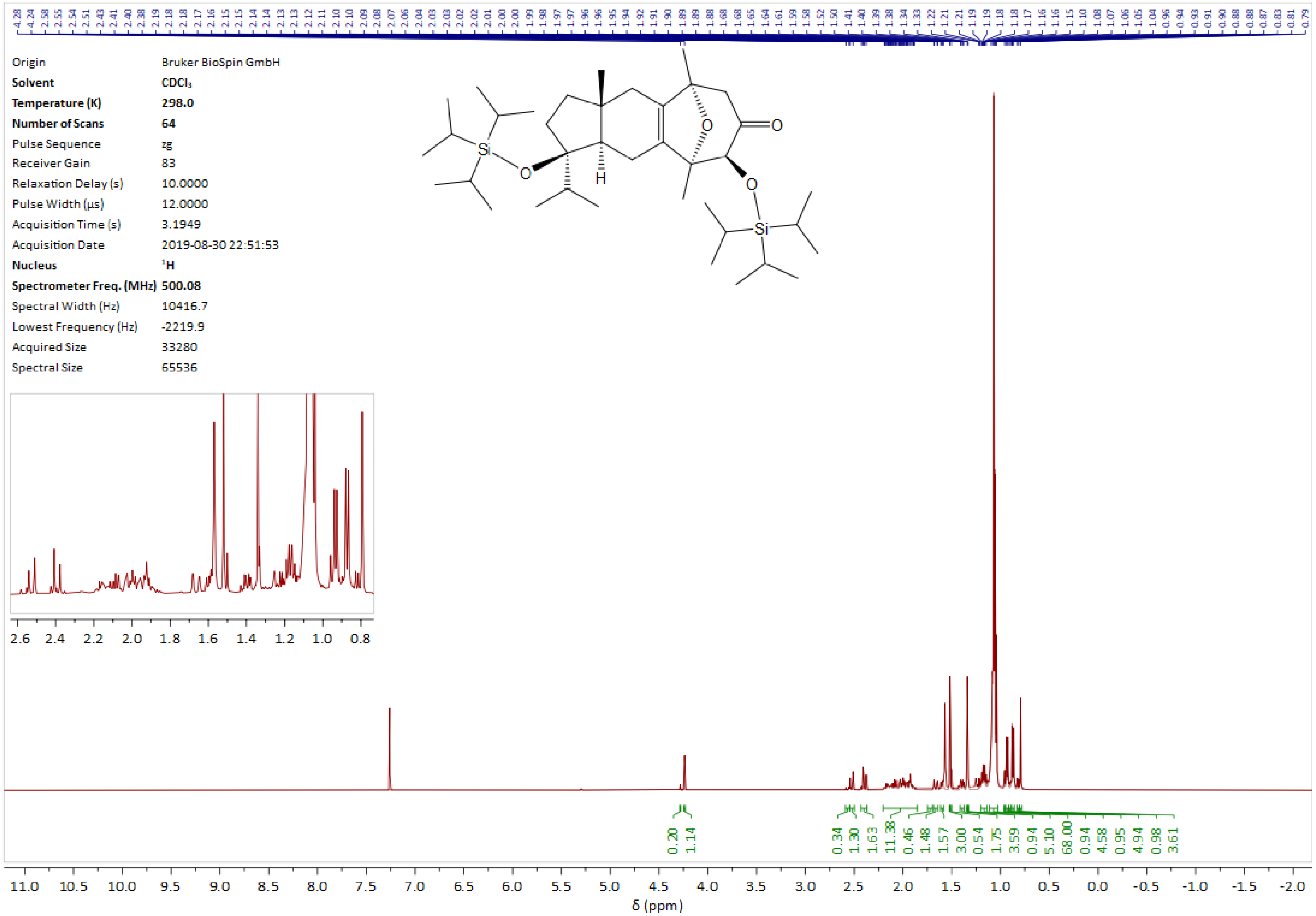

Oxabicyclic ketone 49

^{13}C NMR Spectrum, $CDCl_3$, 126 MHz

Origin	Bruker BioSpin GmbH
**Solvent**	**$CDCl_3$**
**Temperature (K)**	**298.0**
**Number of Scans**	**1000**
Pulse Sequence	zgpg30
Receiver Gain	101
Relaxation Delay (s)	1.0000
Pulse Width (µs)	10.0000
Acquisition Time (s)	1.9047
Acquisition Date	2019-08-30 23:42:11
**Nucleus**	**^{13}C**
**Spectrometer Freq. (MHz)**	**125.76**
Spectral Width (Hz)	31250.0
Lowest Frequency (Hz)	-1777.3
Acquired Size	59522
Spectral Size	131072

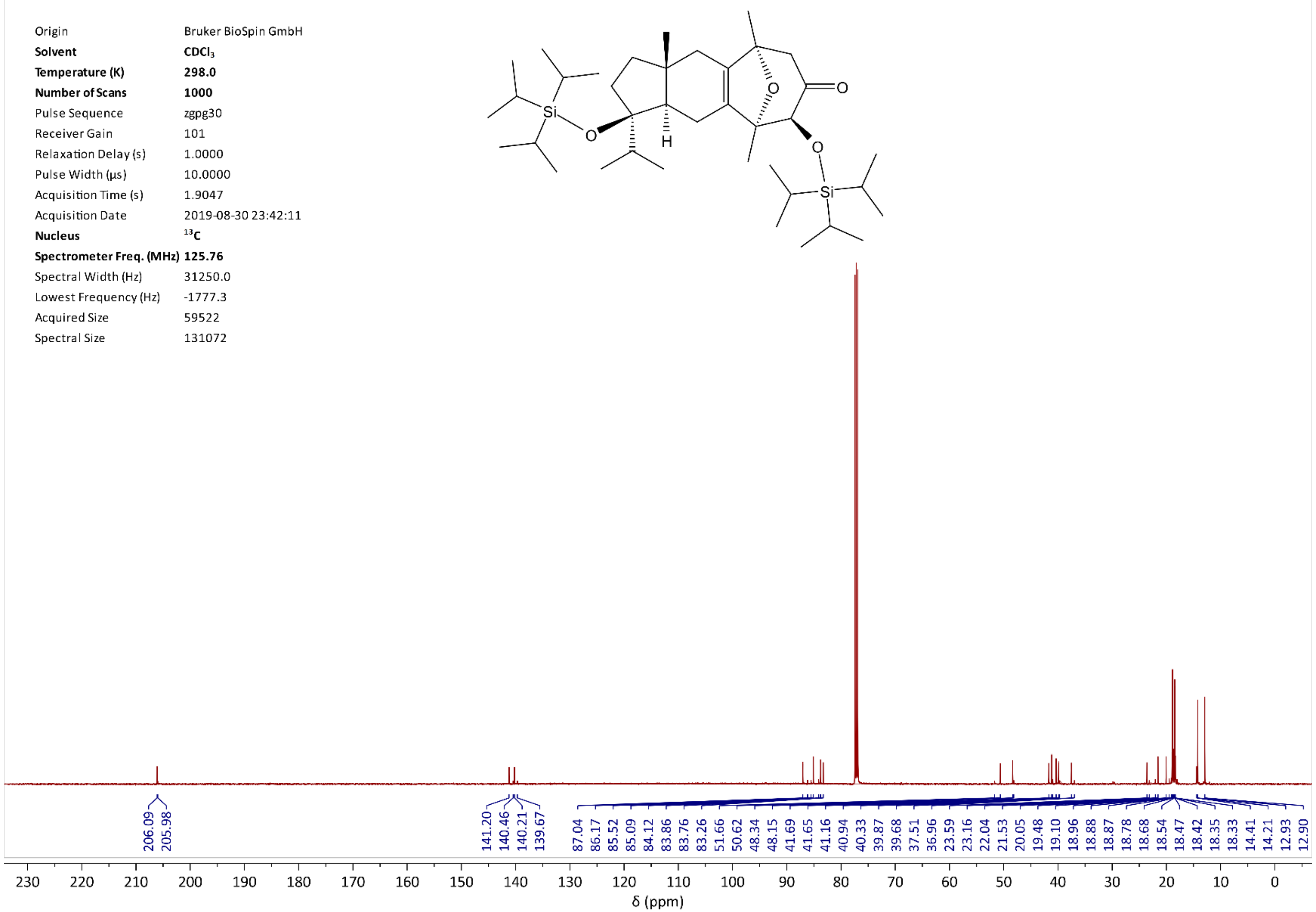

Oxabicyclic ketone 49

HSQC Spectrum, $CDCl_3$, 500 MHz/126 MHz

Origin	Bruker BioSpin GmbH
**Solvent**	**$CDCl_3$**
**Temperature (K)**	**298.0**
**Number of Scans**	**4**
Pulse Sequence	hsqcedetgpsp.3
Receiver Gain	101
Relaxation Delay (s)	1.0000
Pulse Width (μs)	12.0000
Acquisition Time (s)	0.0870
Acquisition Date	2019-08-31 00:23:14
**Spectrometer Freq. (MHz)**	**(500.08, 125.75)**
Spectral Width	(5882.4, 20746.9)
Lowest Frequency	(-601.5, -944.8)
**Nucleus**	**(^{1}H, ^{13}C)**
Acquired Size	(512, 256)
Spectral Size	(2048, 2048)

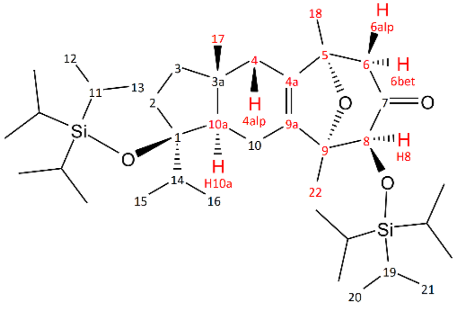

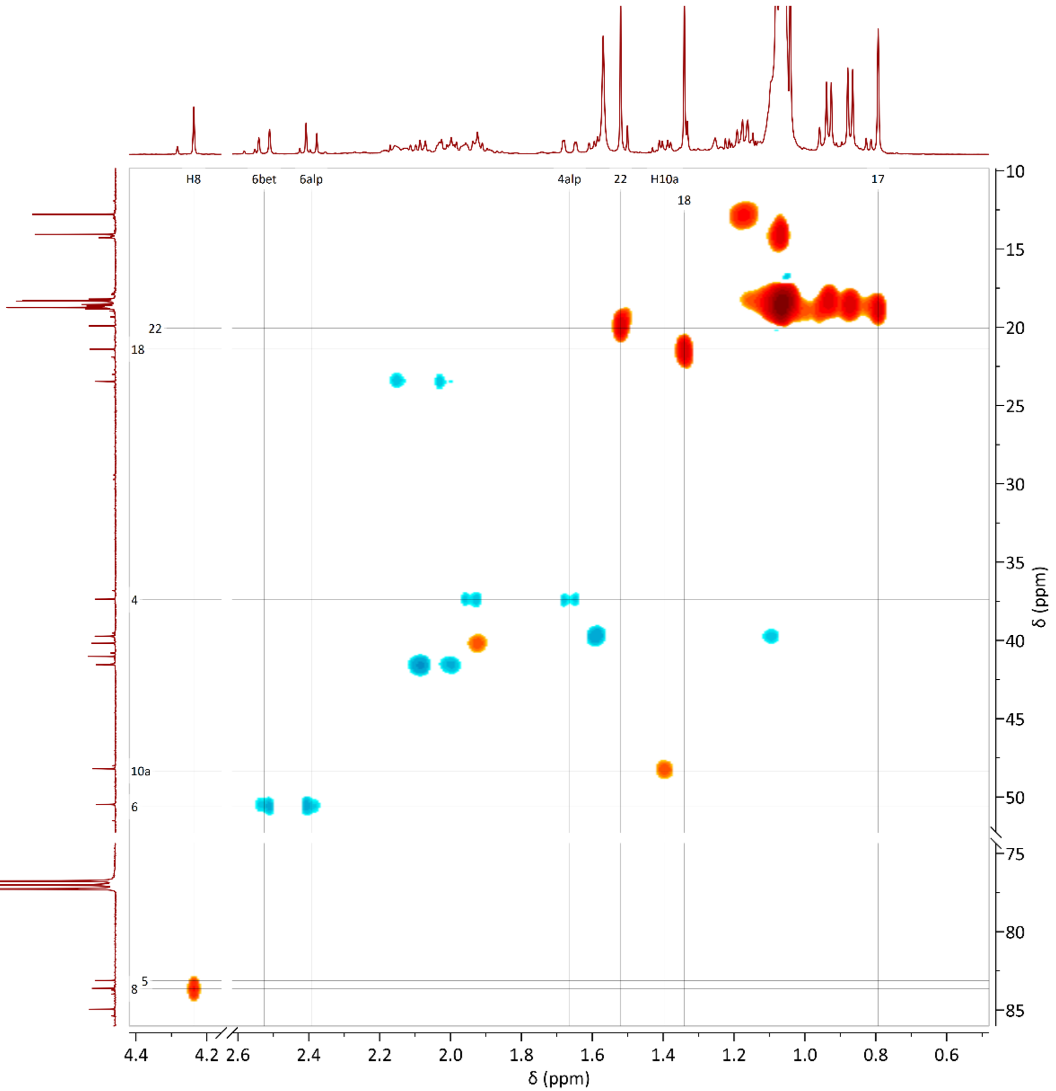

2D correlation maps have been included to help justify the absolute structural assignment of this structure. Not all correlations have been included, only those that help to analyse the absolute structure.

Oxabicyclic ketone 49

HMBC Spectrum, $CDCl_3$, 500 MHz/126 MHz

Origin	Bruker BioSpin GmbH
**Solvent**	**$CDCl_3$**
**Temperature (K)**	**298.0**
**Number of Scans**	**4**
Pulse Sequence	hmbcetgpl2nd.2
Receiver Gain	101
Relaxation Delay (s)	1.5000
Pulse Width (μs)	12.0000
Acquisition Time (s)	0.3113
Acquisition Date	2019-08-31 01:29:26
**Spectrometer Freq. (MHz)**	**(500.08, 125.76)**
Spectral Width	(6578.9, 30177.3)
Lowest Frequency	(-151.1, -1258.6)
**Nucleus**	**(^1H, ^{13}C)**
Acquired Size	(2048, 512)
Spectral Size	(2048, 2048)

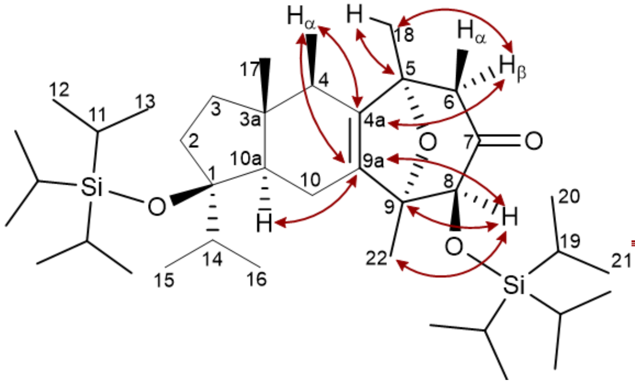

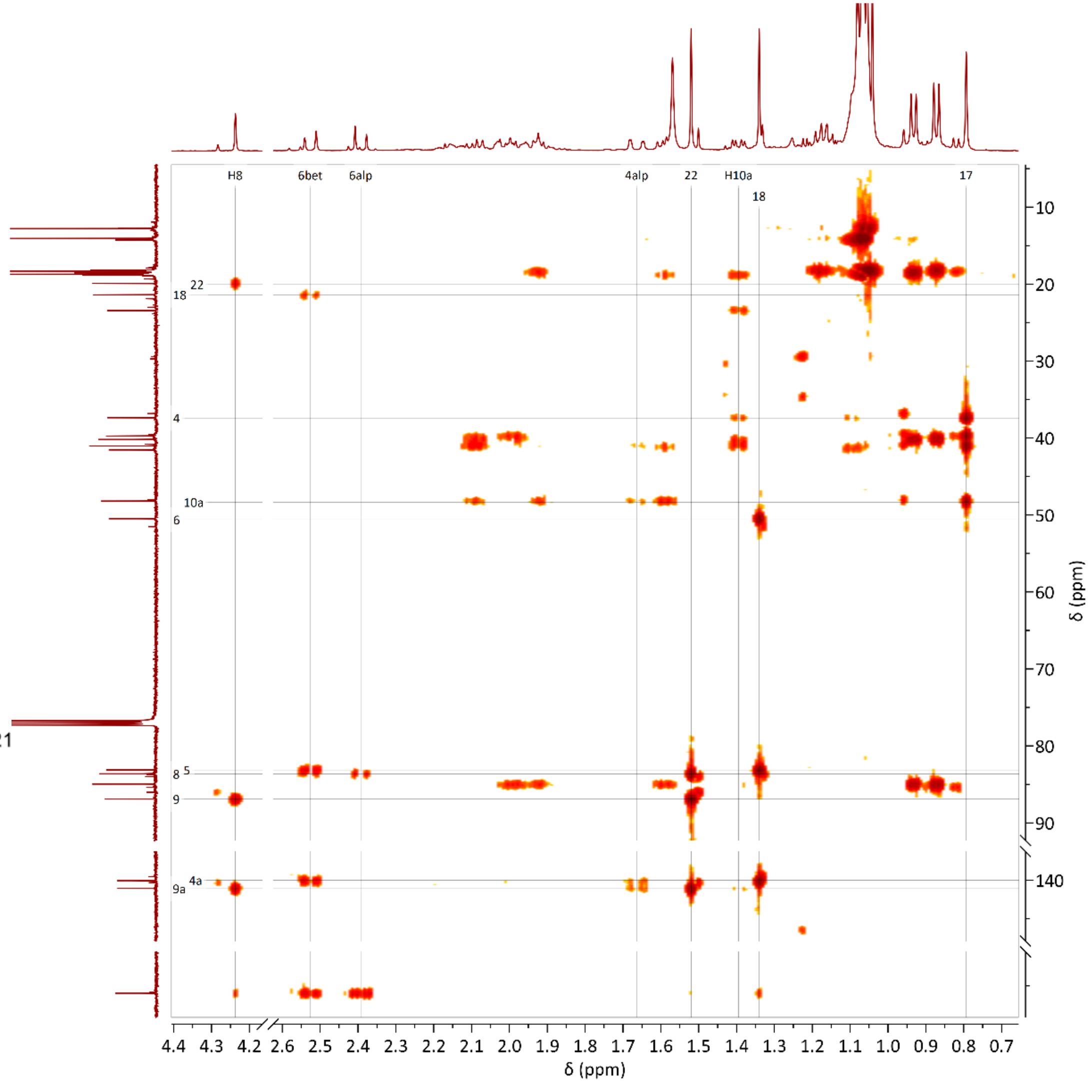

Oxabicyclic ketone 49

NOESY Spectrum, $CDCl_3$, 500 MHz

Origin	Bruker BioSpin GmbH
**Solvent**	**$CDCl_3$**
**Temperature (K)**	**298.0**
**Number of Scans**	**12**
Pulse Sequence	noesygpphpp
Receiver Gain	101
Relaxation Delay (s)	1.4364
Pulse Width (µs)	12.0000
Acquisition Time (s)	0.4506
Acquisition Date	2019-08-31 05:18:21
**Spectrometer Freq. (MHz)**	**(500.07, 500.07)**
Spectral Width	(2272.7, 2272.7)
Lowest Frequency	(105.5, 105.5)
**Nucleus**	**(^{1}H, ^{1}H)**
Acquired Size	(1024, 440)
Spectral Size	(2048, 2048)

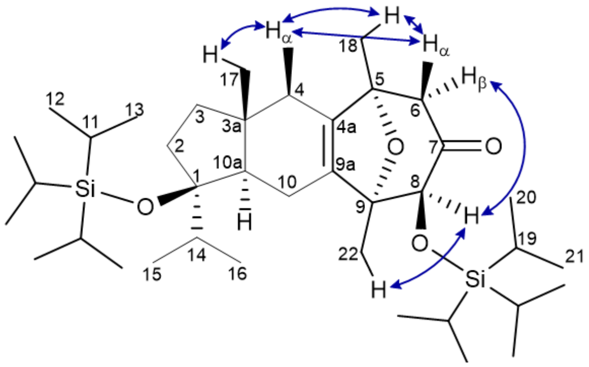

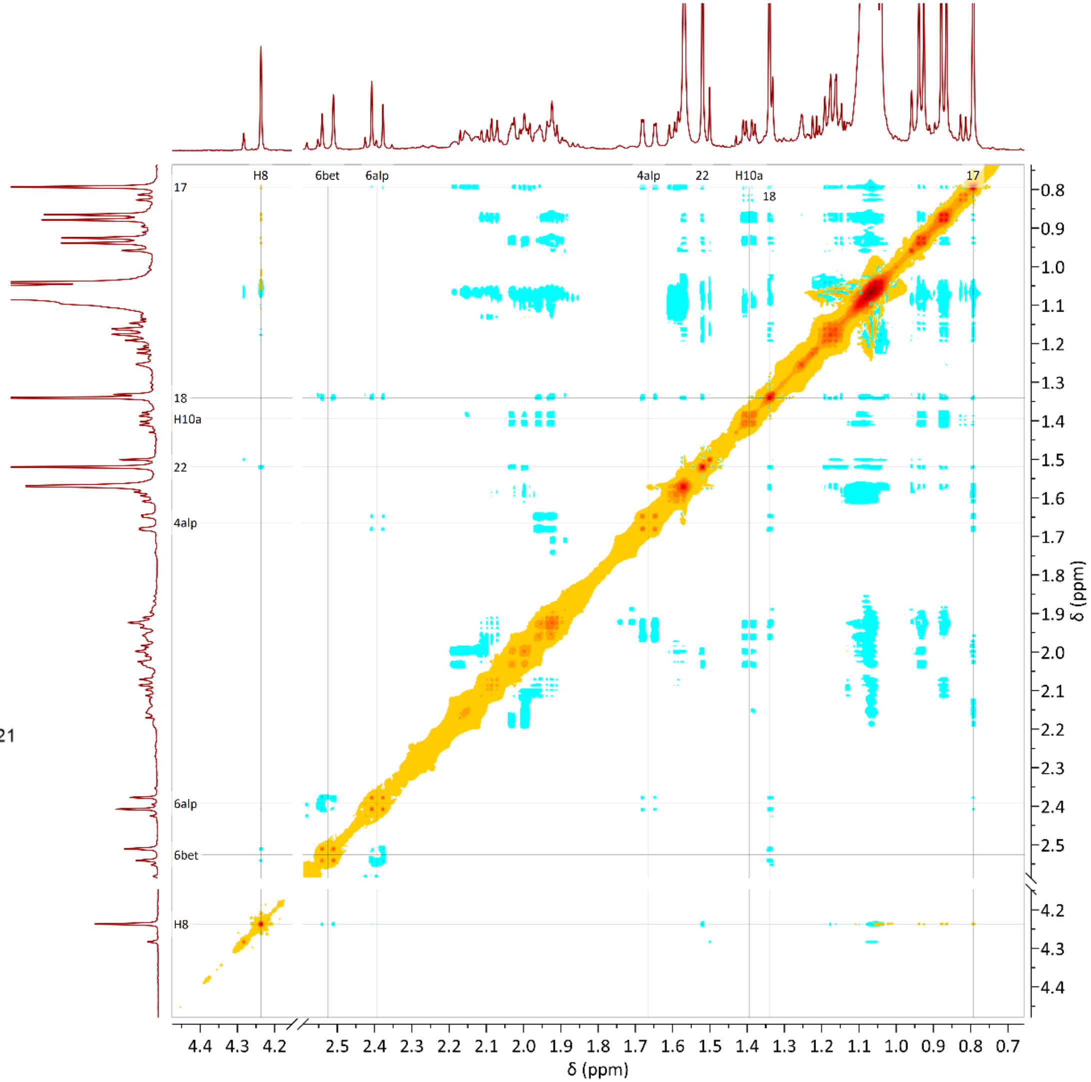

Pentachloroacetone 51

^{1}H NMR Spectrum, $CDCl_3$, 400 MHz

Note: The peak *ca.* 6.5 ppm (integral 0.05) is an impurity.

Origin	Bruker BioSpin GmbH
**Solvent**	**$CDCl_3$**
**Temperature (K)**	**298.0**
**Number of Scans**	**4**
Pulse Sequence	zg30
Receiver Gain	101
Relaxation Delay (s)	2.0000
Pulse Width (µs)	9.4000
Acquisition Time (s)	4.5875
Acquisition Date	2019-11-15 15:52:37
**Nucleus**	**^1H**
**Spectrometer Freq. (MHz)**	**400.13**
Spectral Width (Hz)	7142.9
Lowest Frequency (Hz)	-1180.3
Acquired Size	32768
Spectral Size	65536

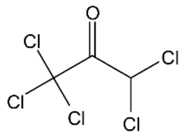

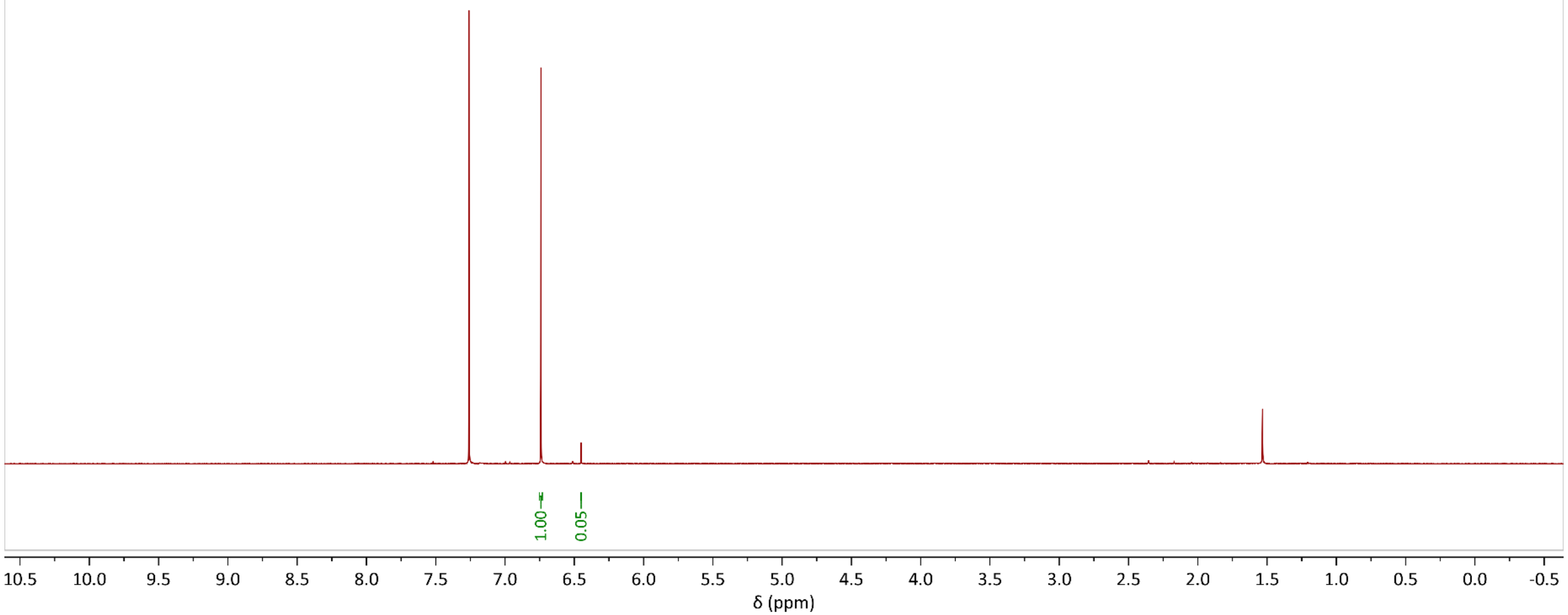

Pentachloroacetone 51

^{13}C NMR Spectrum, $CDCl_3$, 101 MHz

Origin	Bruker BioSpin GmbH
**Solvent**	**$CDCl_3$**
**Temperature (K)**	**298.0**
**Number of Scans**	**512**
Pulse Sequence	zgpg30
Receiver Gain	35
Relaxation Delay (s)	1.0000
Pulse Width (µs)	10.0000
Acquisition Time (s)	2.3809
Acquisition Date	2019-11-16 07:33:47
**Nucleus**	**^{13}C**
**Spectrometer Freq. (MHz)**	**100.62**
Spectral Width (Hz)	25000.0
Lowest Frequency (Hz)	-915.6
Acquired Size	59522
Spectral Size	131072

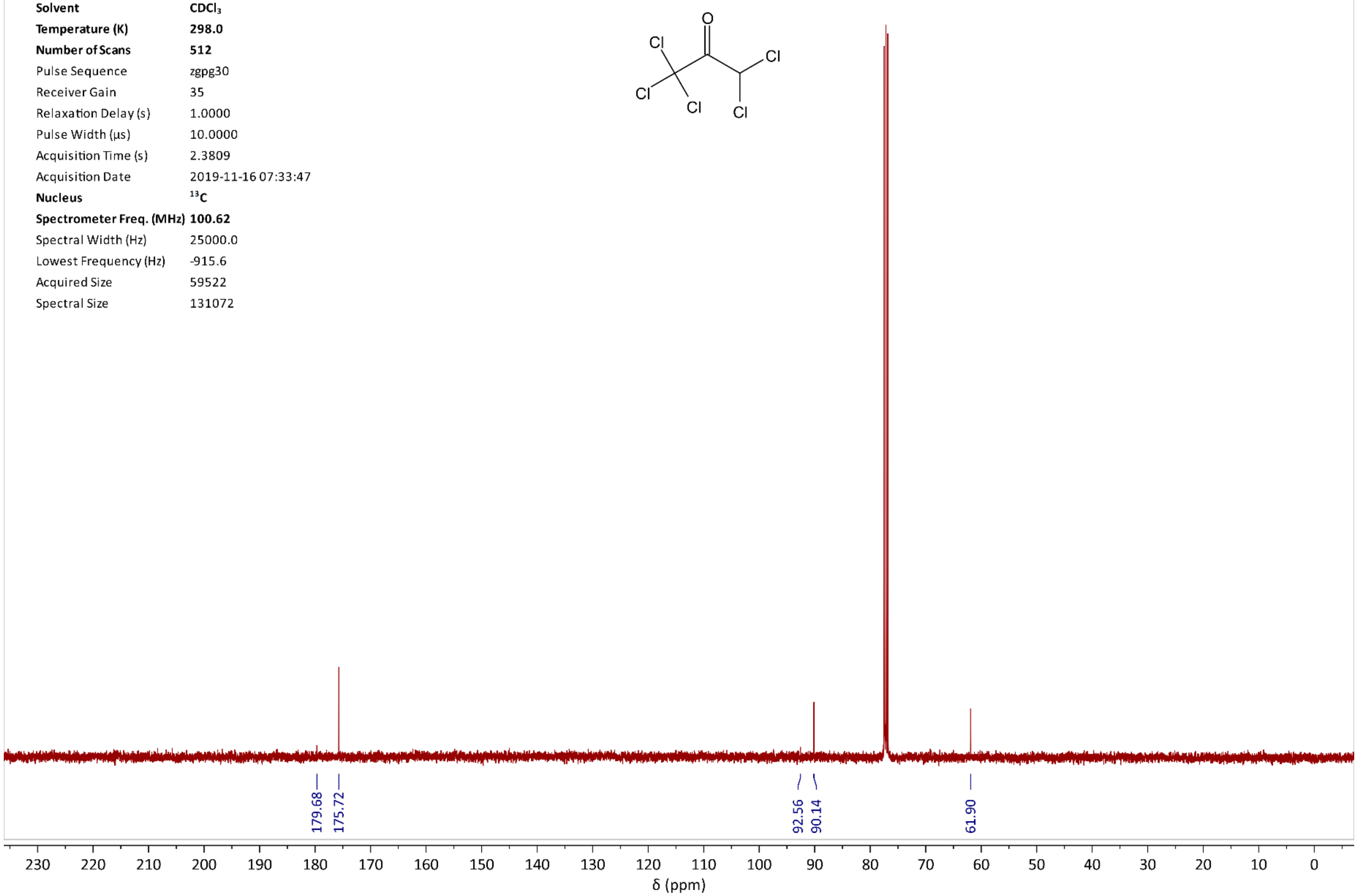

Oxabicyclic ketone 52

^{1}H NMR Spectrum, $CDCl_3$, 500 MHz

Origin	Bruker BioSpin GmbH
**Solvent**	**$CDCl_3$**
**Temperature (K)**	**298.0**
**Number of Scans**	**32**
Pulse Sequence	zg
Receiver Gain	65
Relaxation Delay (s)	10.0000
Pulse Width (µs)	12.0000
Acquisition Time (s)	3.1949
Acquisition Date	2019-09-25 16:33:37
**Nucleus**	**^{1}H**
**Spectrometer Freq. (MHz)**	**500.08**
Spectral Width (Hz)	10416.7
Lowest Frequency (Hz)	-2220.1
Acquired Size	33280
Spectral Size	65536

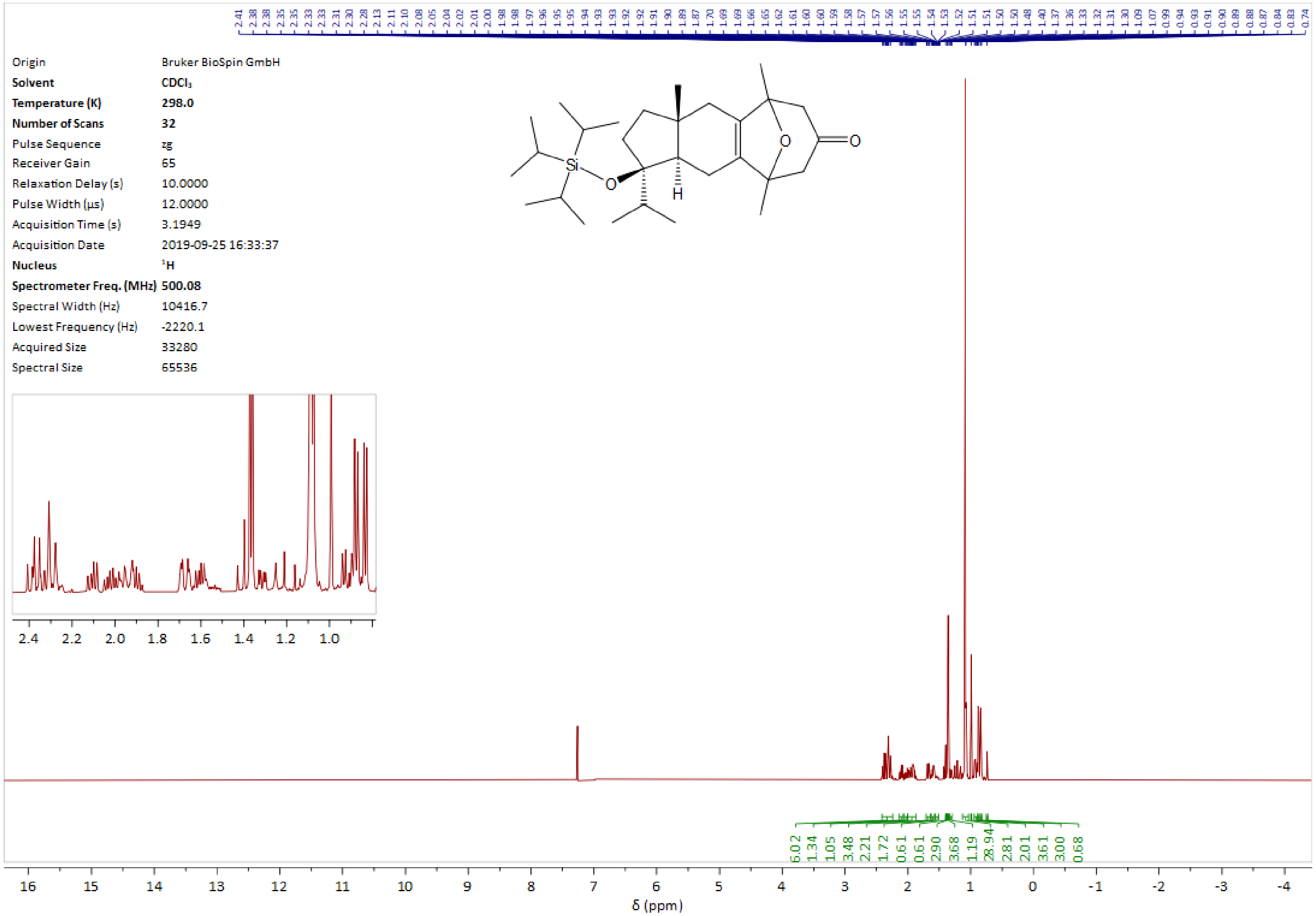

Oxabicyclic ketone 52

^{13}C NMR Spectrum, $CDCl_3$, 126 MHz

Origin	Bruker BioSpin GmbH
**Solvent**	**$CDCl_3$**
**Temperature (K)**	**298.0**
**Number of Scans**	**600**
Pulse Sequence	zgpg30
Receiver Gain	101
Relaxation Delay (s)	1.0000
Pulse Width (μs)	10.0000
Acquisition Time (s)	1.9047
Acquisition Date	2019-09-25 17:04:36
**Nucleus**	**^{13}C**
**Spectrometer Freq. (MHz)**	**125.76**
Spectral Width (Hz)	31250.0
Lowest Frequency (Hz)	-1777.9
Acquired Size	59522
Spectral Size	131072

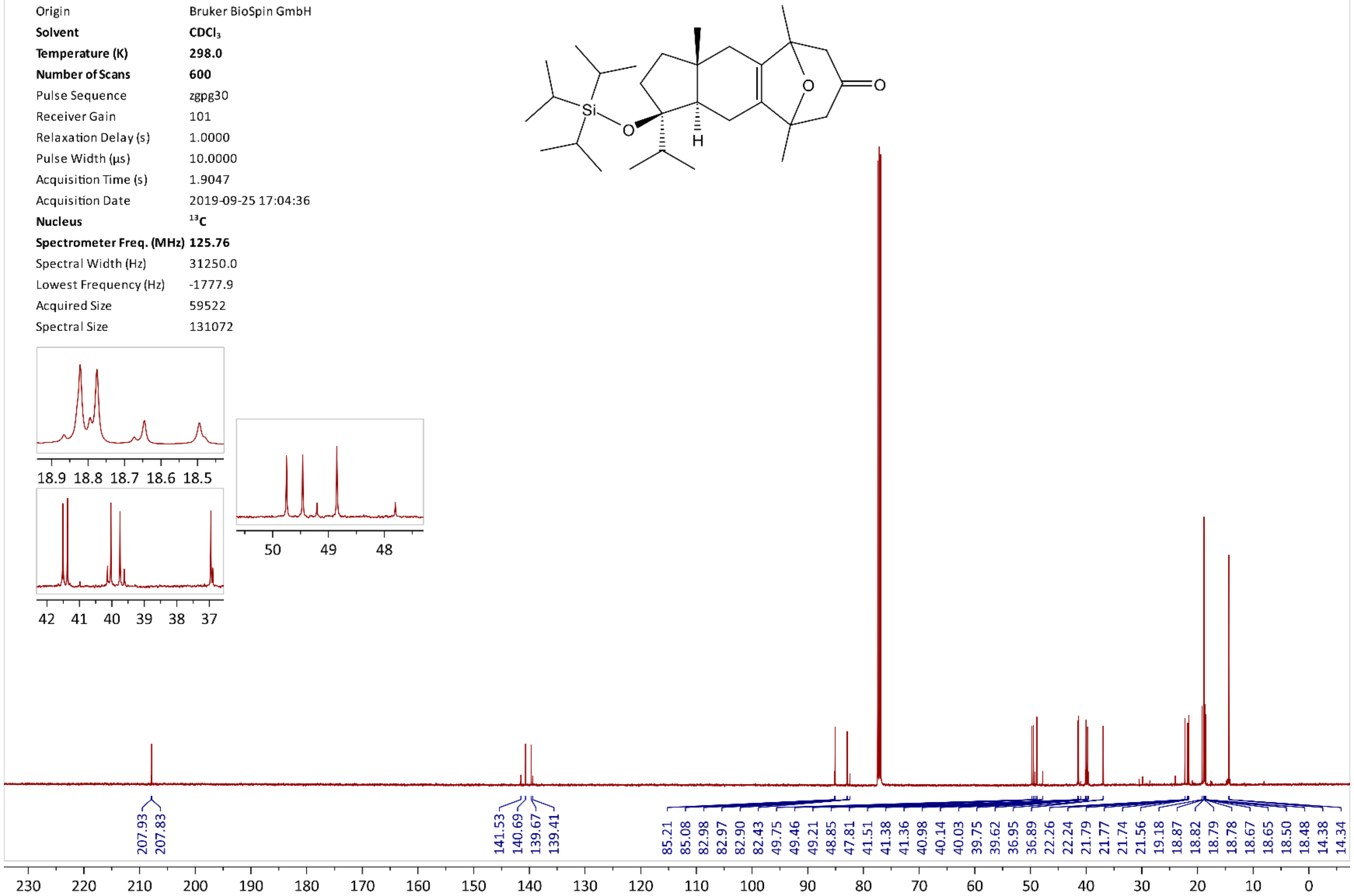

Tetrabromocyclopropene 56

^{13}C NMR Spectrum, $CDCl_3$, 101 MHz

Origin	Bruker BioSpin GmbH
**Solvent**	**$CDCl_3$**
**Temperature (K)**	**294.5**
**Number of Scans**	**380**
Pulse Sequence	udeft
Receiver Gain	2050
Relaxation Delay (s)	3.0000
Pulse Width (µs)	8.8000
Acquisition Time (s)	0.3599
Acquisition Date	2020-01-14 14:16:00
**Nucleus**	**^{13}C**
**Spectrometer Freq. (MHz)**	**100.62**
Spectral Width (Hz)	25252.5
Lowest Frequency (Hz)	-1112.6
Acquired Size	9089
Spectral Size	65536

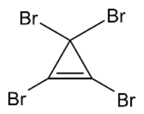

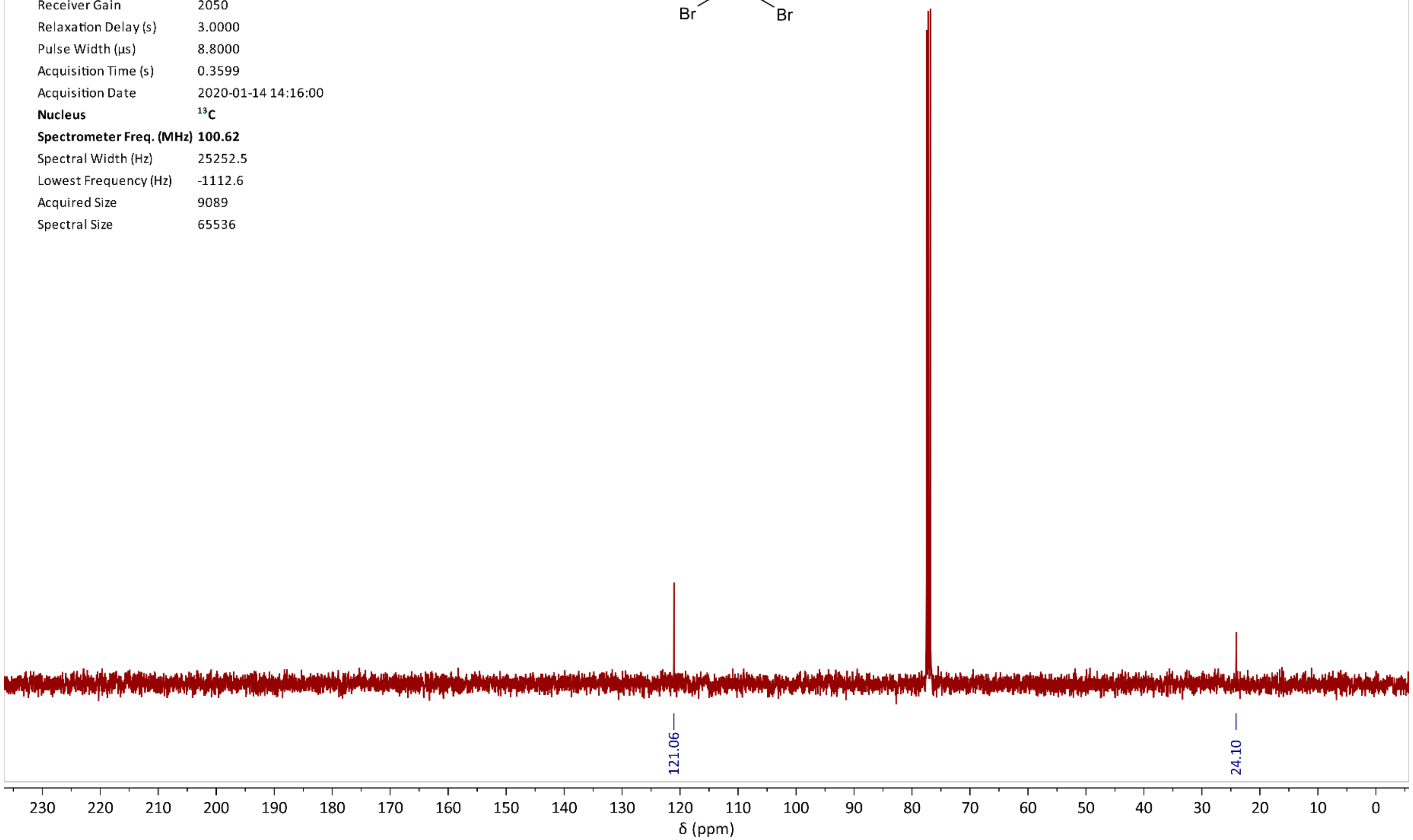

Tetrachlorocyclopropanes 57a and 58a

^{1}H NMR Spectrum, $CDCl_3$, 400 MHz

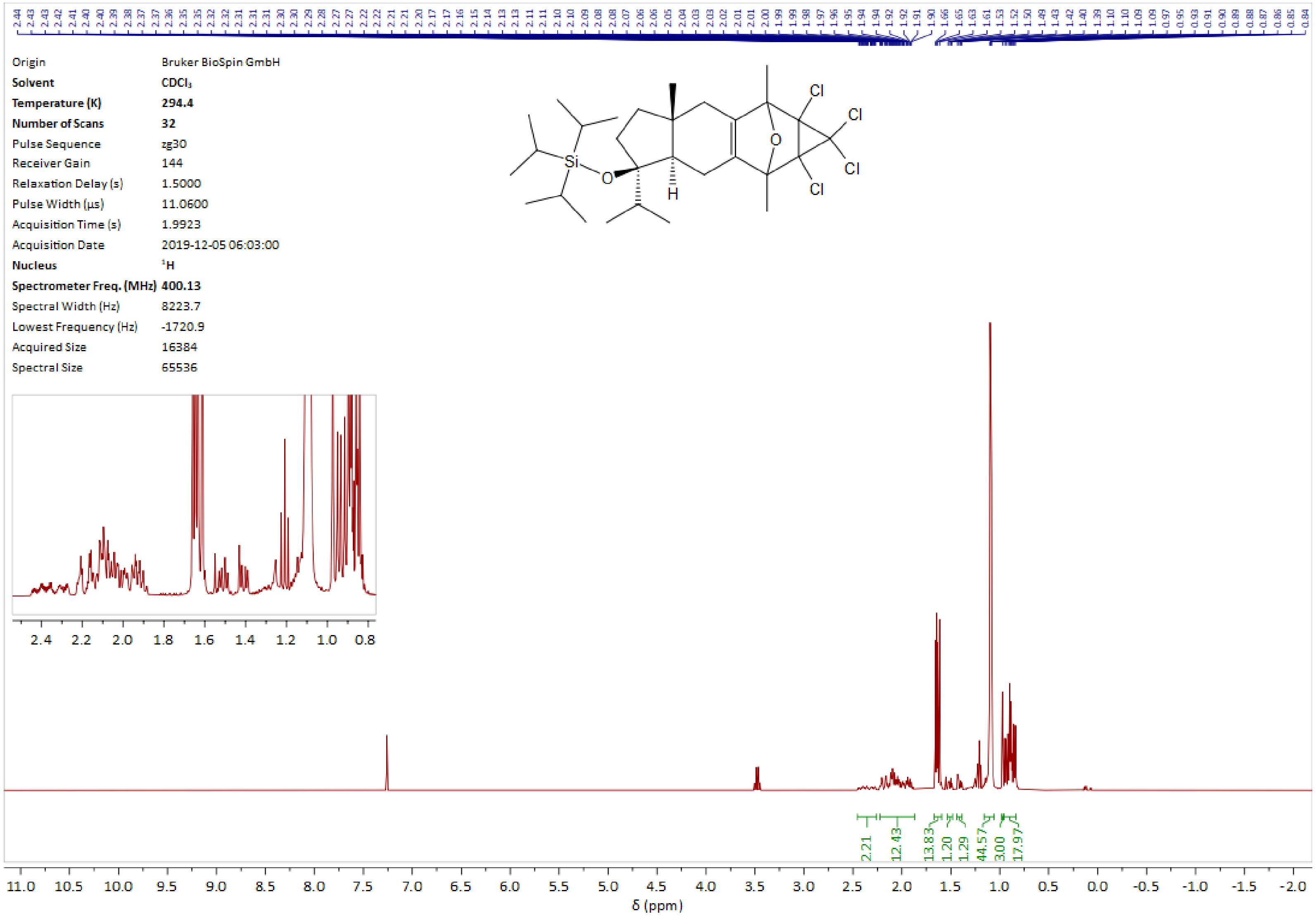

Tetrachlorocyclopropanes 57a and 58a

^{13}C NMR Spectrum, $CDCl_3$, 101 MHz

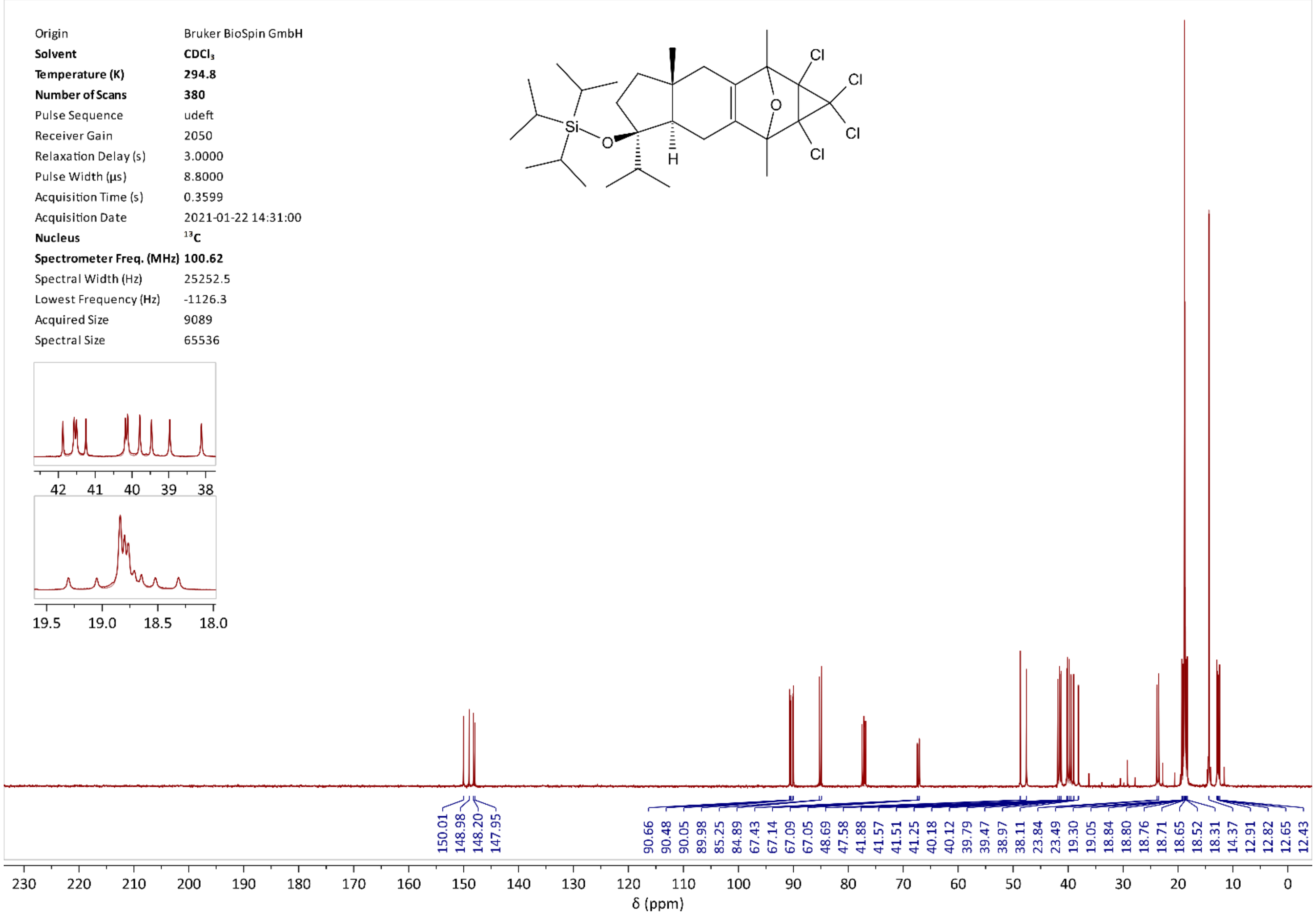

Tetrabromocyclopropanes 57b and 58b

^{1}H NMR Spectrum, $CDCl_3$, 400 MHz

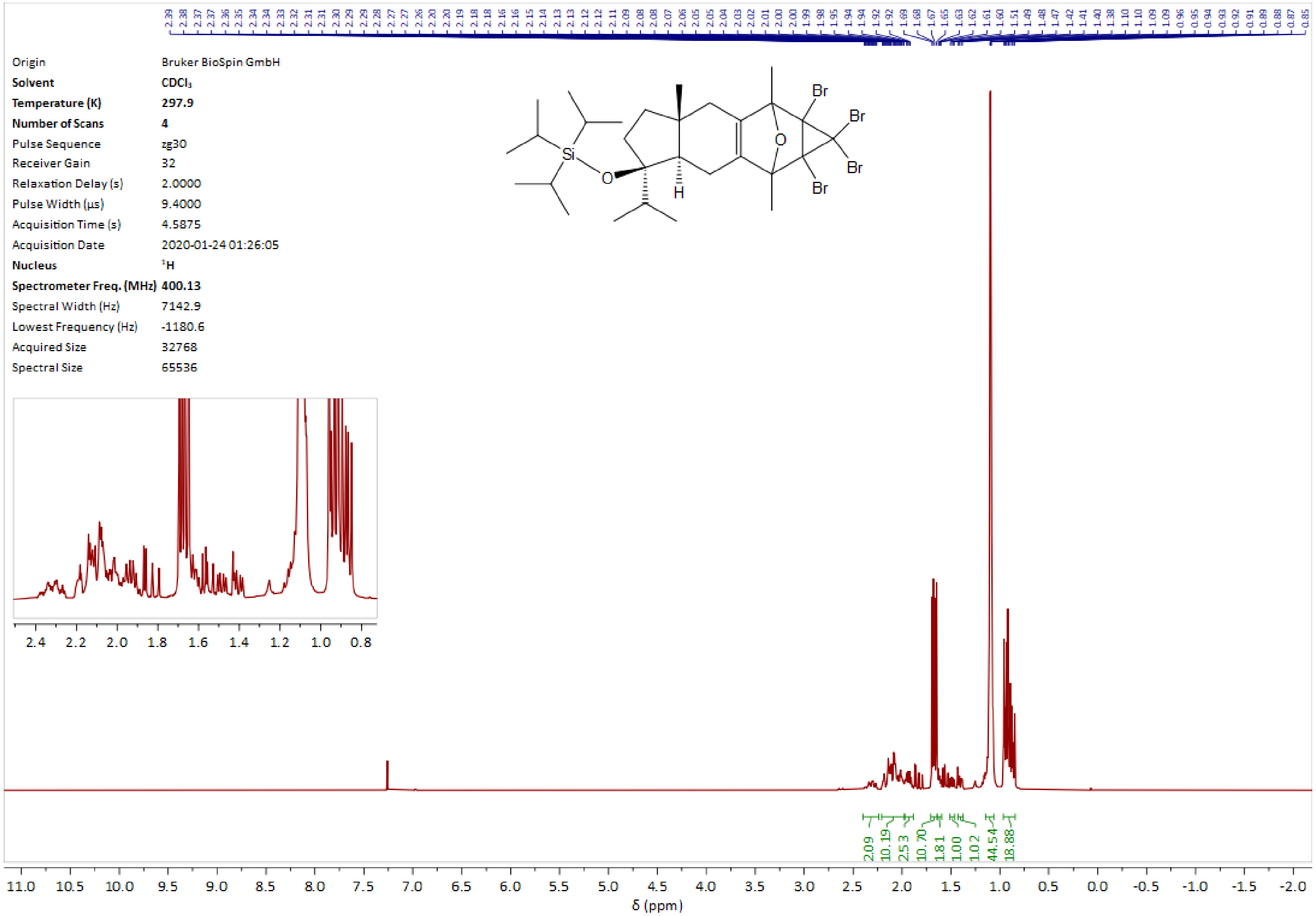

Tetrabromocyclopropanes 57b and 58b

^{13}C NMR Spectrum, $CDCl_3$, 101 MHz

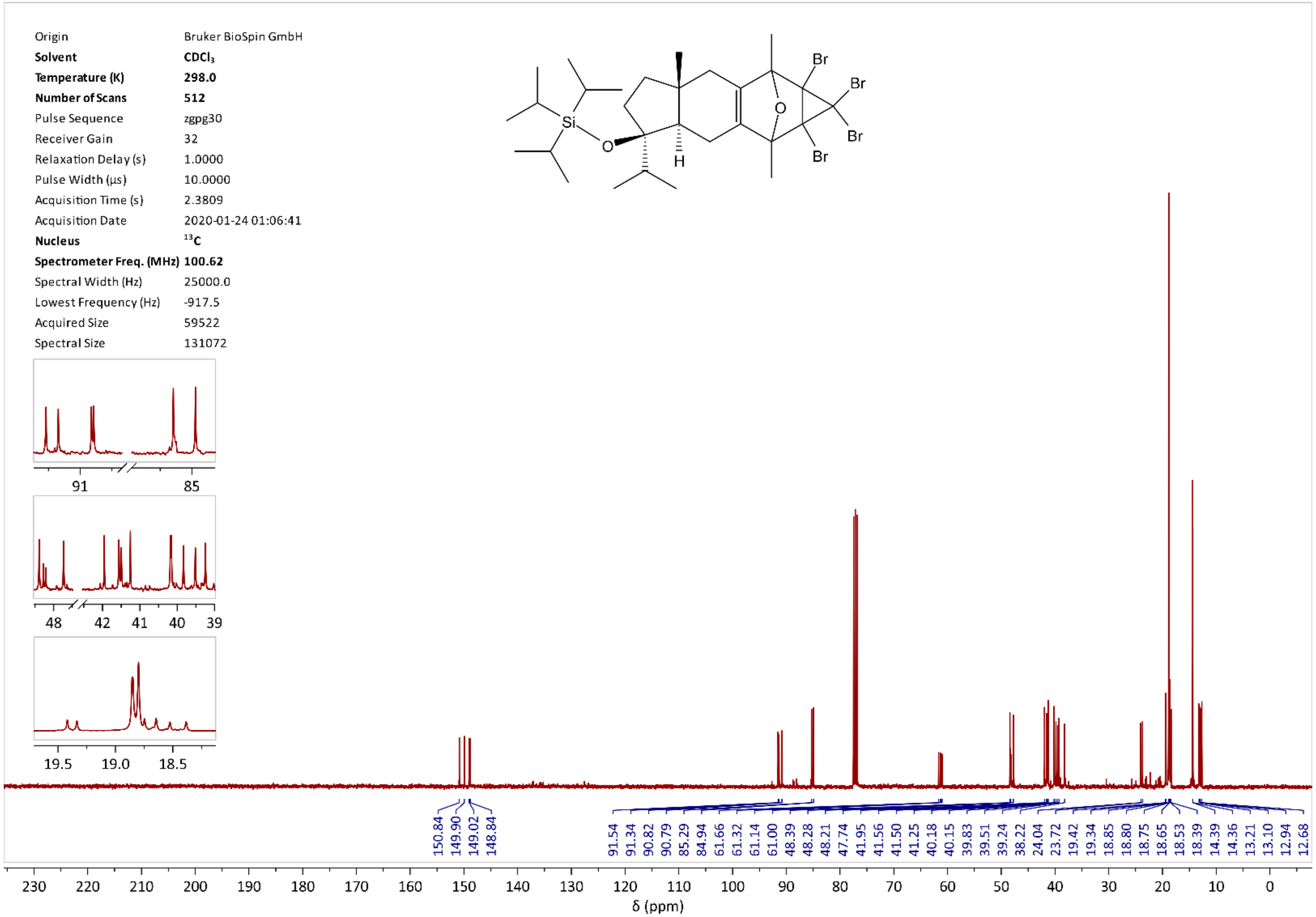

Tetrabromides 59 and 60

^{1}H NMR Spectrum, $CDCl_3$, 400 MHz

Origin	Bruker BioSpin GmbH
**Solvent**	**$CDCl_3$**
**Temperature (K)**	**294.6**
**Number of Scans**	**32**
Pulse Sequence	zg30
Receiver Gain	57
Relaxation Delay (s)	1.5000
Pulse Width (μs)	11.0600
Acquisition Time (s)	1.9923
Acquisition Date	2020-01-25 04:17:00
**Nucleus**	**^{1}H**
**Spectrometer Freq. (MHz)**	**400.13**
Spectral Width (Hz)	8223.7
Lowest Frequency (Hz)	-1747.4
Acquired Size	16384
Spectral Size	65536

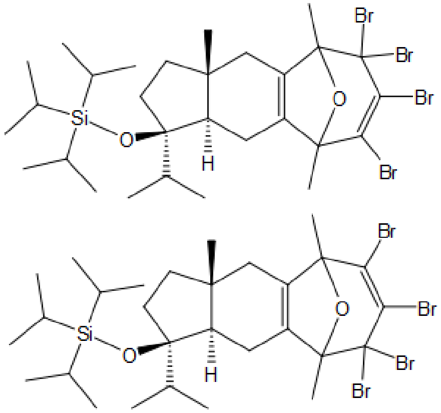

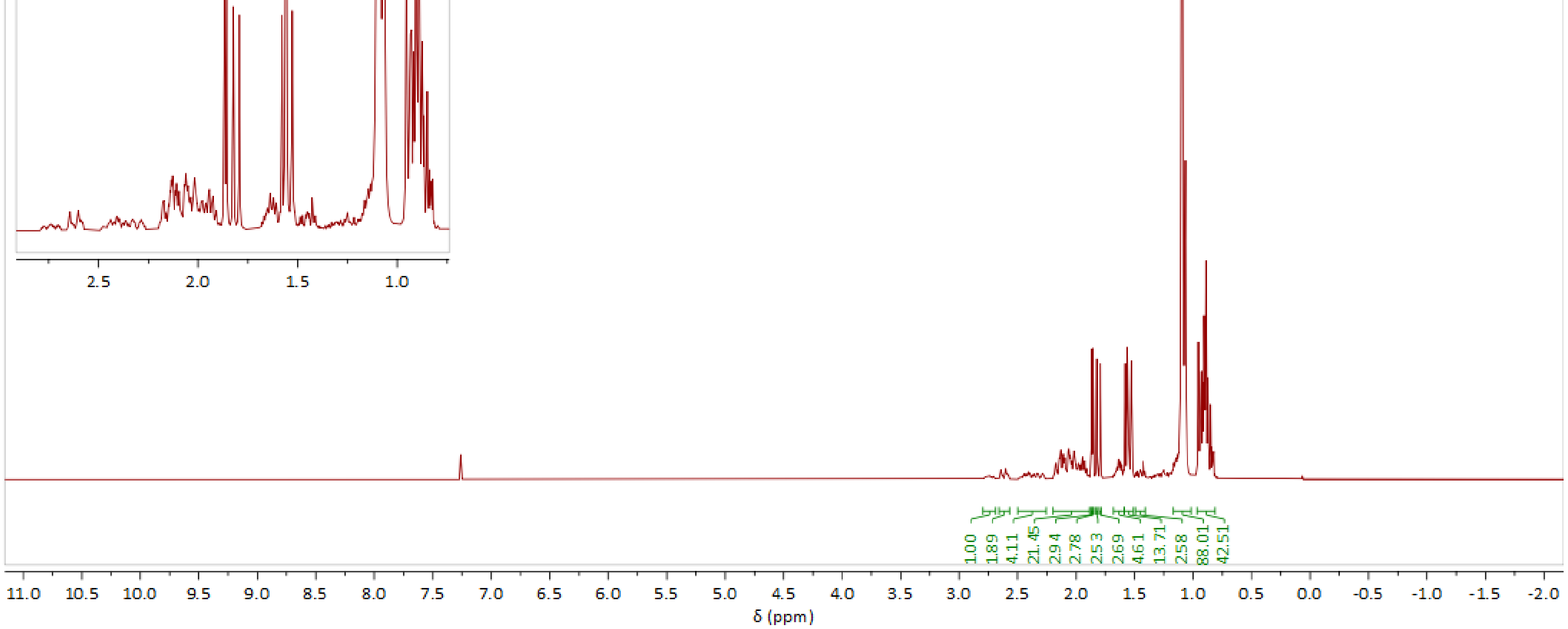

Tetrabromides 59 and 60

^{13}C NMR Spectrum, $CDCl_3$, 101 MHz

Origin	Bruker BioSpin GmbH
**Solvent**	**$CDCl_3$**
**Temperature (K)**	**294.8**
**Number of Scans**	**380**
Pulse Sequence	udeft
Receiver Gain	2050
Relaxation Delay (s)	3.0000
Pulse Width (μs)	8.8000
Acquisition Time (s)	0.3599
Acquisition Date	2020-01-25 03:23:00
**Nucleus**	**^{13}C**
**Spectrometer Freq. (MHz)**	**100.62**
Spectral Width (Hz)	25252.5
Lowest Frequency (Hz)	-1126.3
Acquired Size	9089
Spectral Size	65536

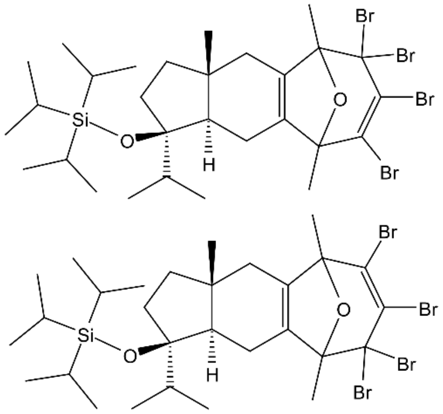

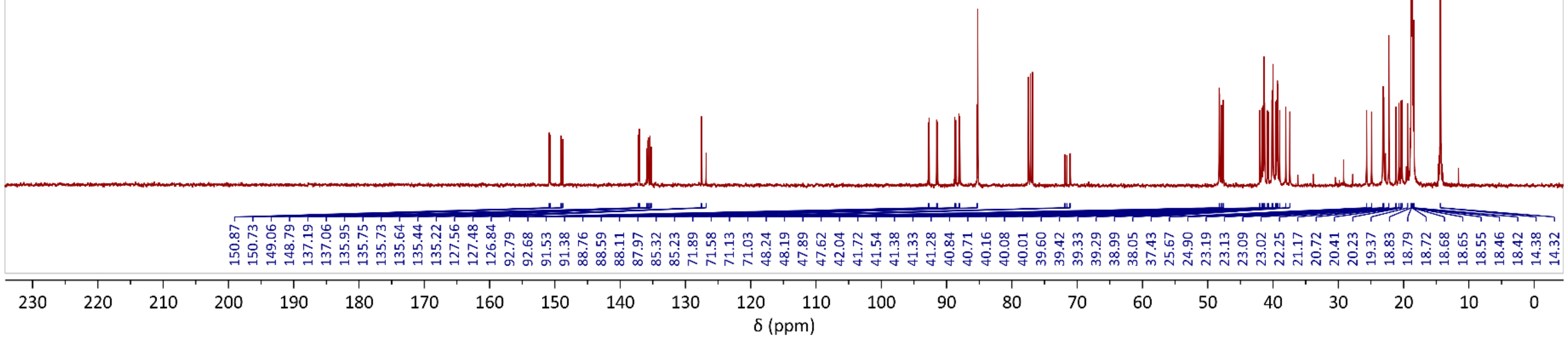

Sulfoxides 66 and 67

^{1}H NMR Spectrum, CD_2Cl_2, 400 MHz

Origin	Bruker BioSpin GmbH
**Solvent**	**CD_2Cl_2**
**Temperature (K)**	**294.0**
**Number of Scans**	**32**
Pulse Sequence	zg30
Receiver Gain	57
Relaxation Delay (s)	1.5000
Pulse Width (µs)	11.0600
Acquisition Time (s)	1.9923
Acquisition Date	2021-01-29 08:57:00
**Nucleus**	**^1H**
**Spectrometer Freq. (MHz)**	**400.13**
Spectral Width (Hz)	8223.7
Lowest Frequency (Hz)	-1726.6
Acquired Size	16384
Spectral Size	65536

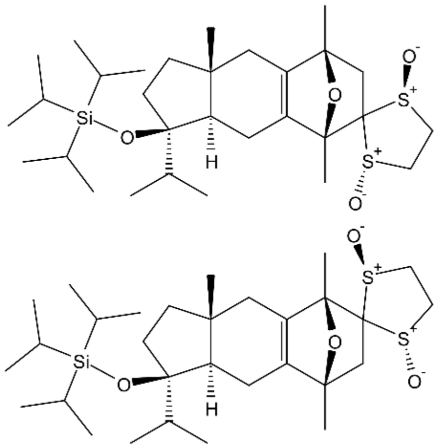

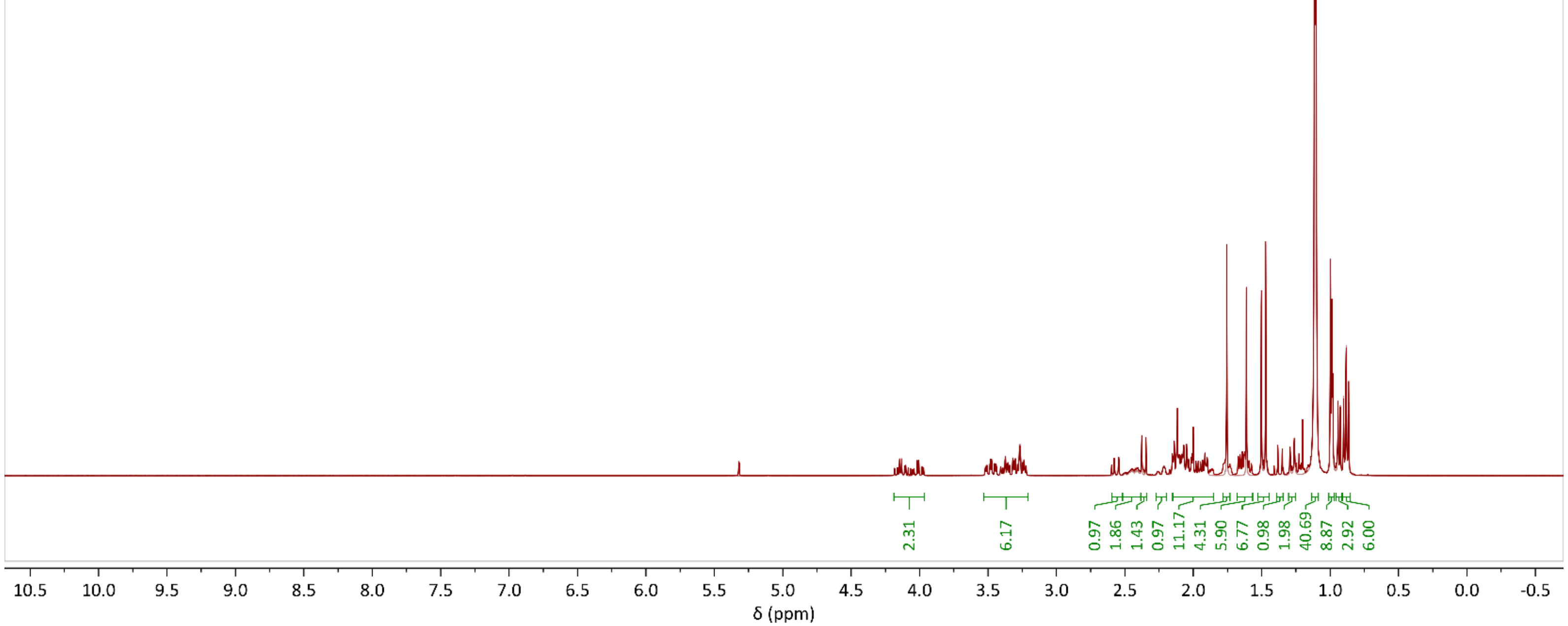

Sulfoxides 66 and 67

^{13}C NMR Spectrum, CD_2Cl_2, 101 MHz

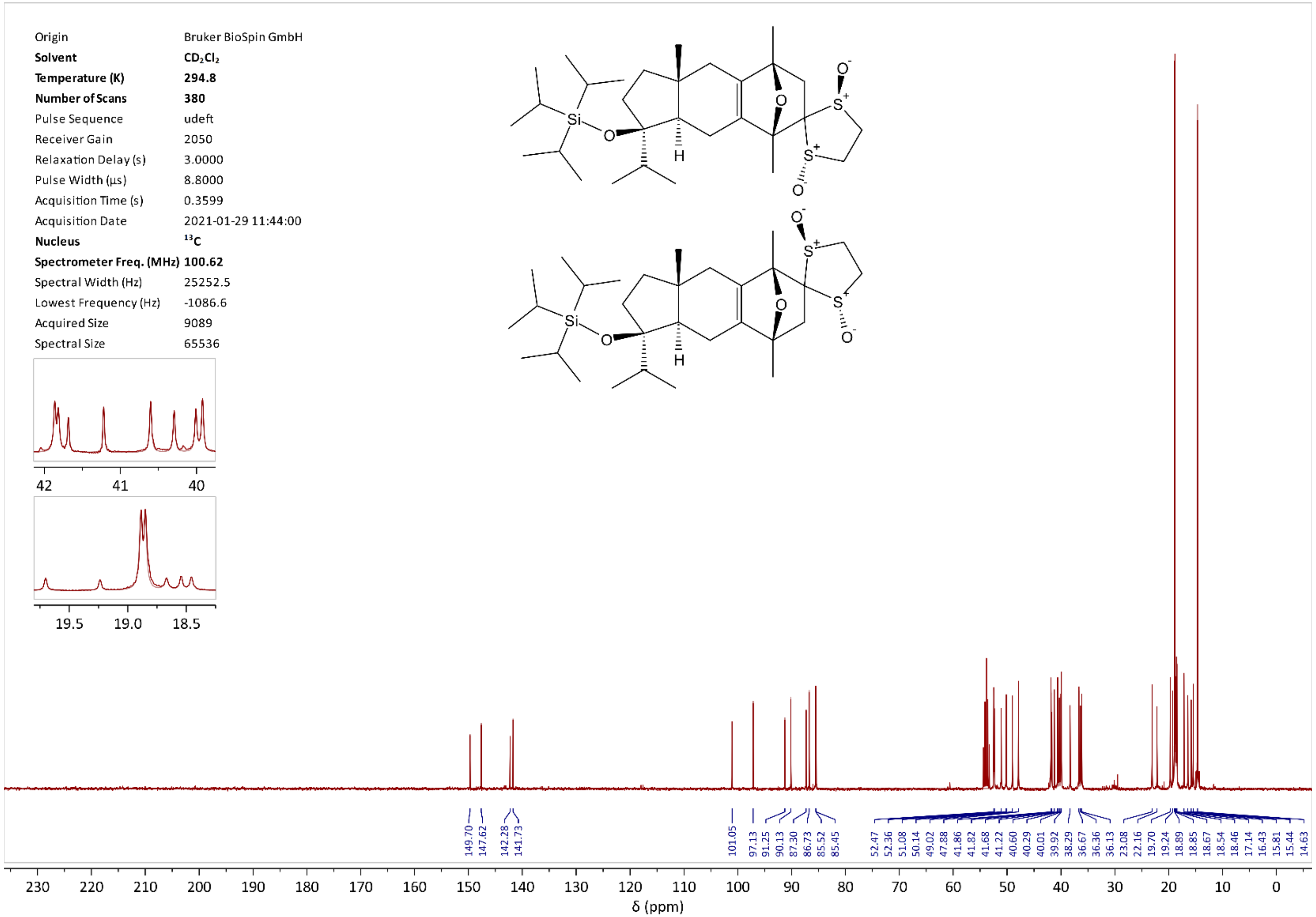

Thioacetals 68 and 69

^{1}H NMR Spectrum, $CDCl_3$, 400 MHz

Origin	Bruker BioSpin GmbH
**Solvent**	**$CDCl_3$**
**Temperature (K)**	**298.0**
**Number of Scans**	**4**
Pulse Sequence	zg30
Receiver Gain	12
Relaxation Delay (s)	2.0000
Pulse Width (µs)	9.4000
Acquisition Time (s)	4.5875
Acquisition Date	2021-01-29 21:13:30
**Nucleus**	**^{1}H**
**Spectrometer Freq. (MHz)**	**400.13**
Spectral Width (Hz)	7142.9
Lowest Frequency (Hz)	-1180.2
Acquired Size	32768
Spectral Size	65536

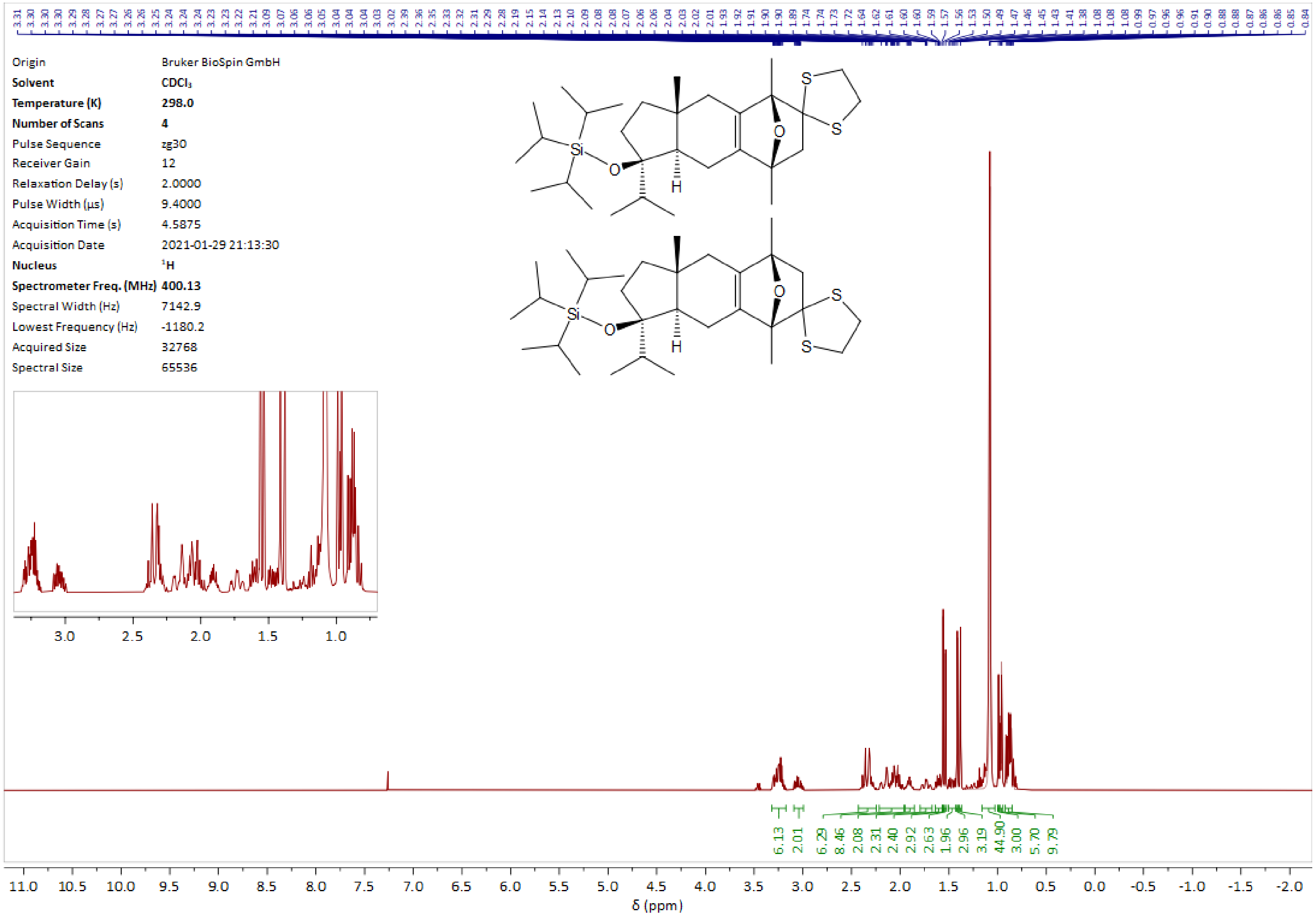

Thioacetals 68 and 69

^{13}C NMR Spectrum, $CDCl_3$, 101 MHz

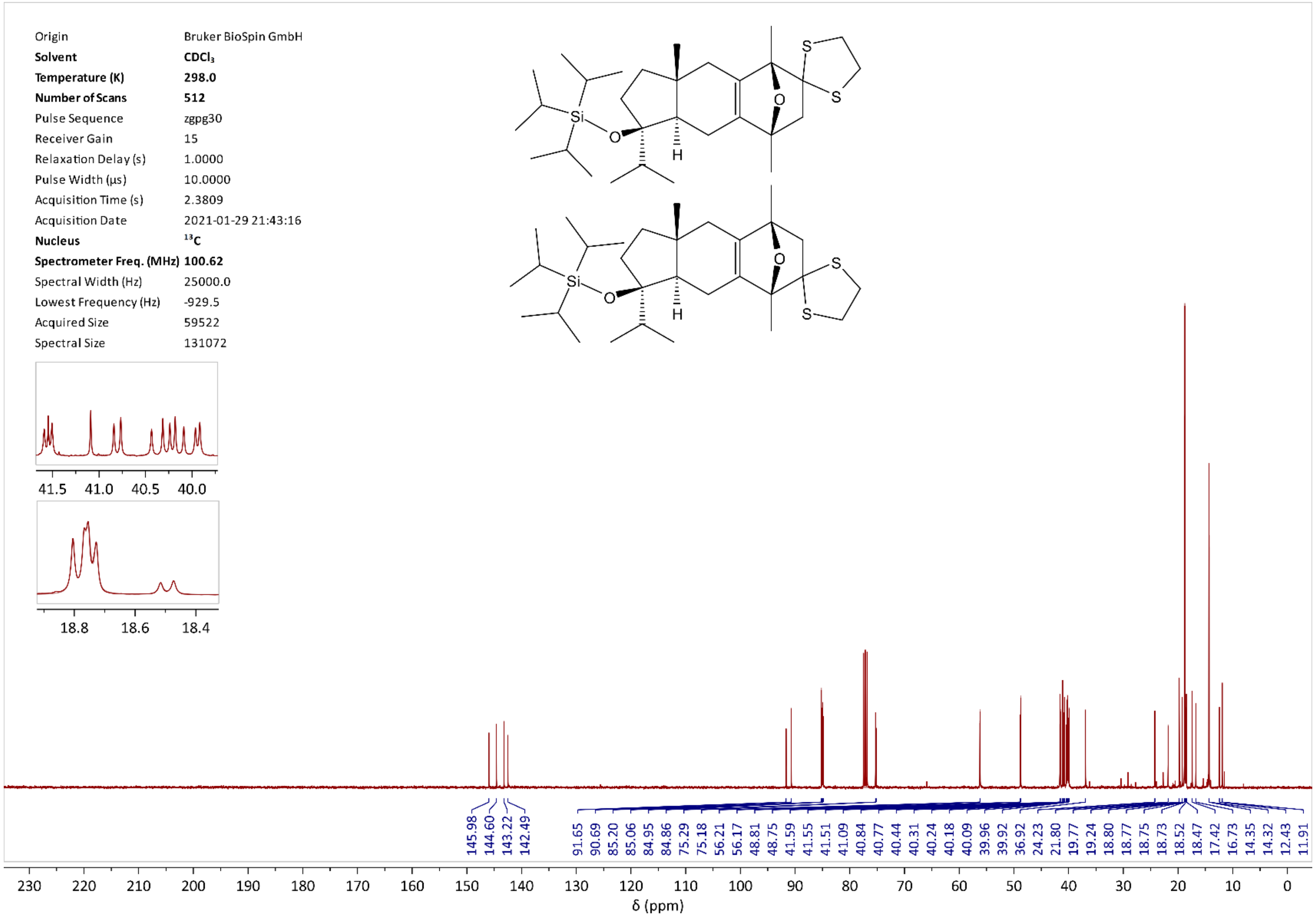

Oxanorbornenone 70

^{1}H NMR Spectrum, CD_2Cl_2, 400 MHz

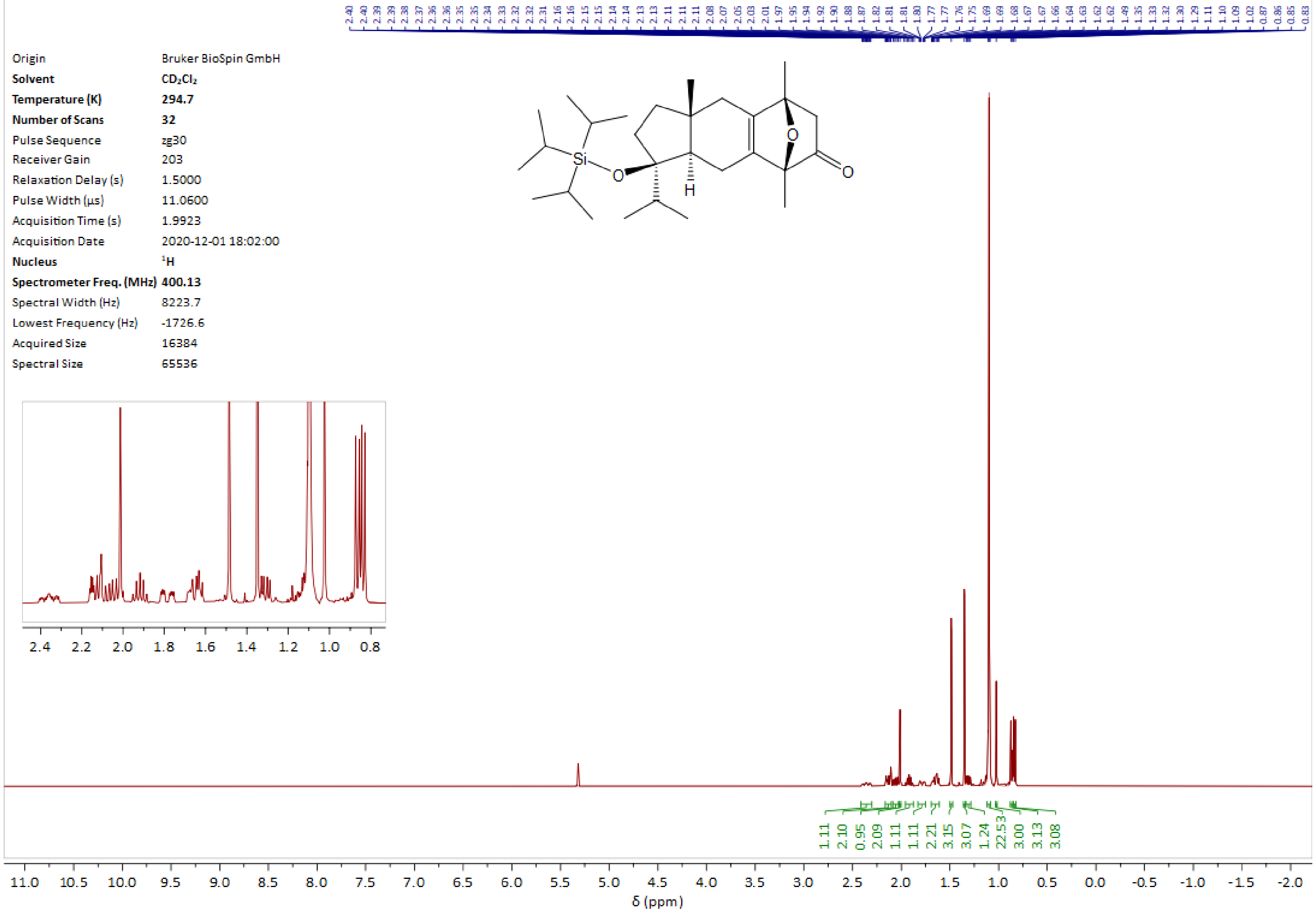

Origin	Bruker BioSpin GmbH
**Solvent**	**CD_2Cl_2**
**Temperature (K)**	**294.7**
**Number of Scans**	**32**
Pulse Sequence	zg30
Receiver Gain	203
Relaxation Delay (s)	1.5000
Pulse Width (μs)	11.0600
Acquisition Time (s)	1.9923
Acquisition Date	2020-12-01 18:02:00
**Nucleus**	**^{1}H**
**Spectrometer Freq. (MHz)**	**400.13**
Spectral Width (Hz)	8223.7
Lowest Frequency (Hz)	-1726.6
Acquired Size	16384
Spectral Size	65536

Oxanorbornenone 70

^{13}C NMR Spectrum, CD_2Cl_2, 101 MHz

Origin	Bruker BioSpin GmbH
**Solvent**	**CD_2Cl_2**
**Temperature (K)**	**295.1**
**Number of Scans**	**380**
Pulse Sequence	udeft
Receiver Gain	1440
Relaxation Delay (s)	3.0000
Pulse Width (µs)	8.8000
Acquisition Time (s)	0.3599
Acquisition Date	2020-12-01 18:12:00
**Nucleus**	**^{13}C**
**Spectrometer Freq. (MHz)**	**100.62**
Spectral Width (Hz)	25252.5
Lowest Frequency (Hz)	-1085.3
Acquired Size	9089
Spectral Size	65536

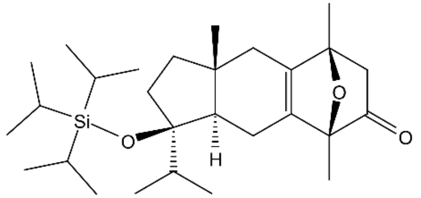

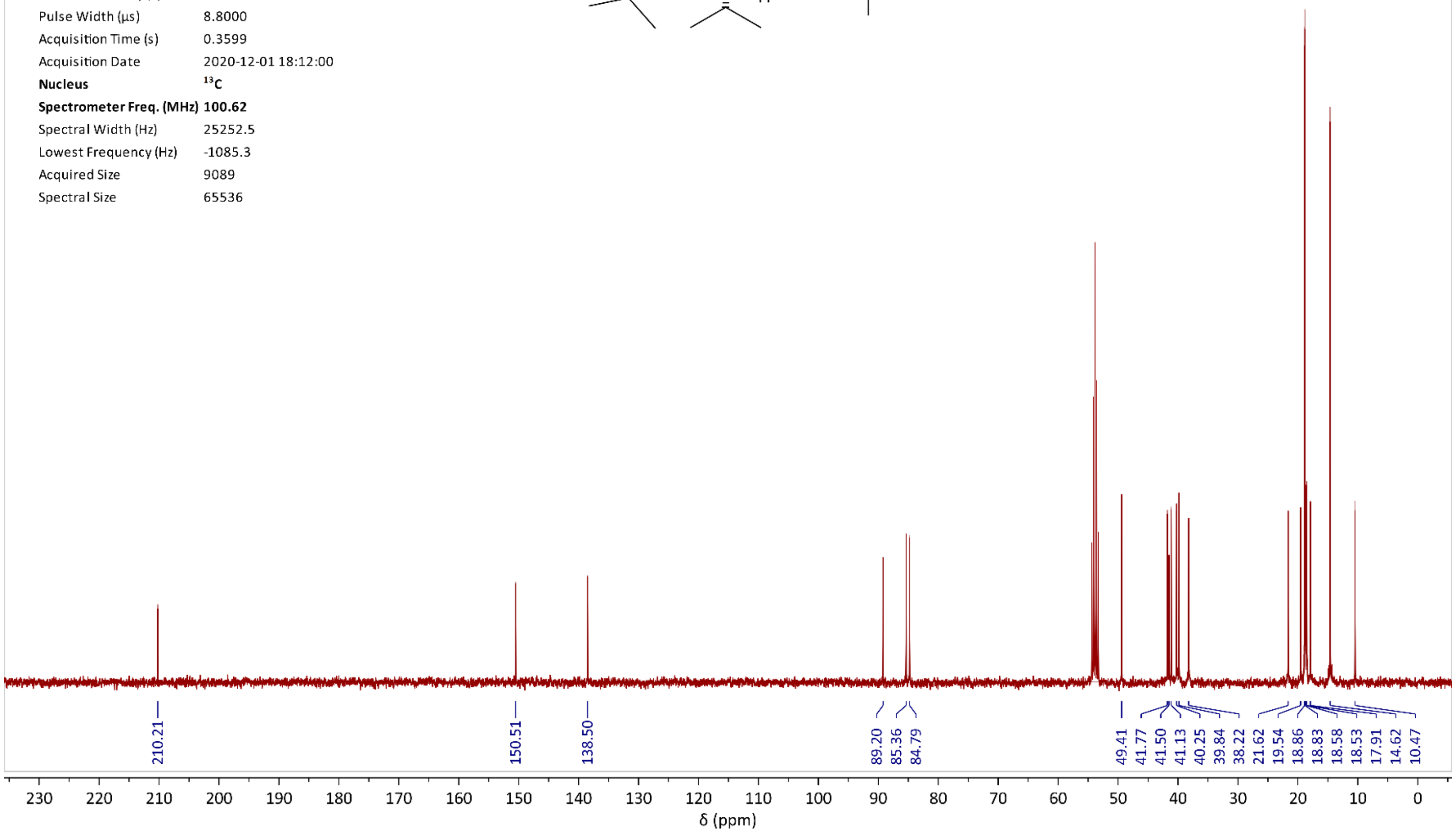

Oxanorbornenone 71

^{1}H NMR Spectrum, CD_2Cl_2, 400 MHz

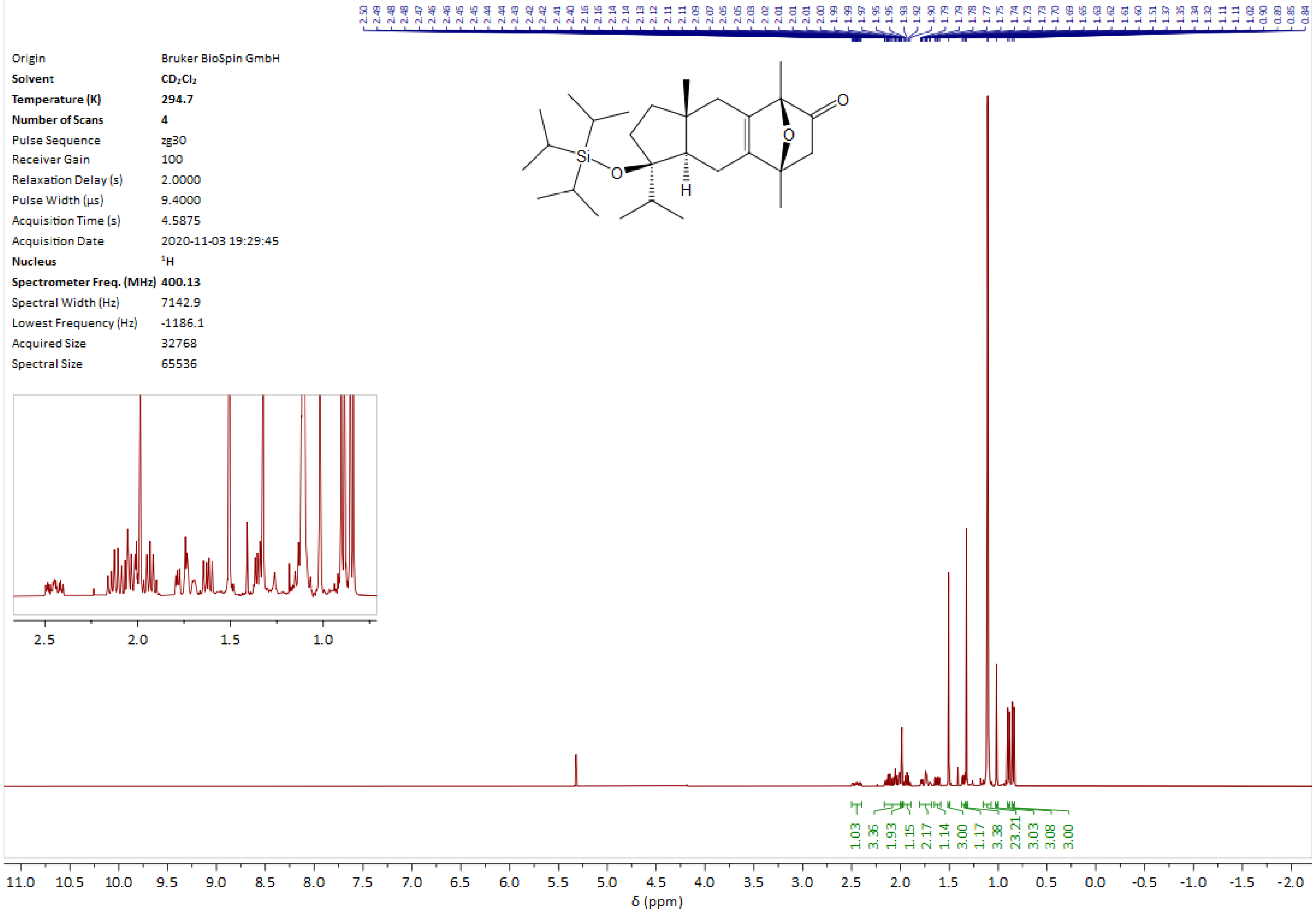

Oxanorbornenone 71

^{13}C NMR Spectrum, CD_2Cl_2, 101 MHz

Origin	Bruker BioSpin GmbH
**Solvent**	**CD_2Cl_2**
**Temperature (K)**	**294.9**
**Number of Scans**	**512**
Pulse Sequence	zgpg30
Receiver Gain	15
Relaxation Delay (s)	1.0000
Pulse Width (µs)	10.0000
Acquisition Time (s)	2.3809
Acquisition Date	2020-11-03 19:10:04
**Nucleus**	**^{13}C**
**Spectrometer Freq. (MHz)**	**100.62**
Spectral Width (Hz)	25000.0
Lowest Frequency (Hz)	-889.4
Acquired Size	59522
Spectral Size	131072

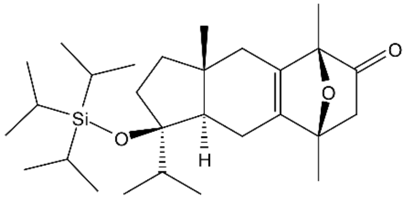

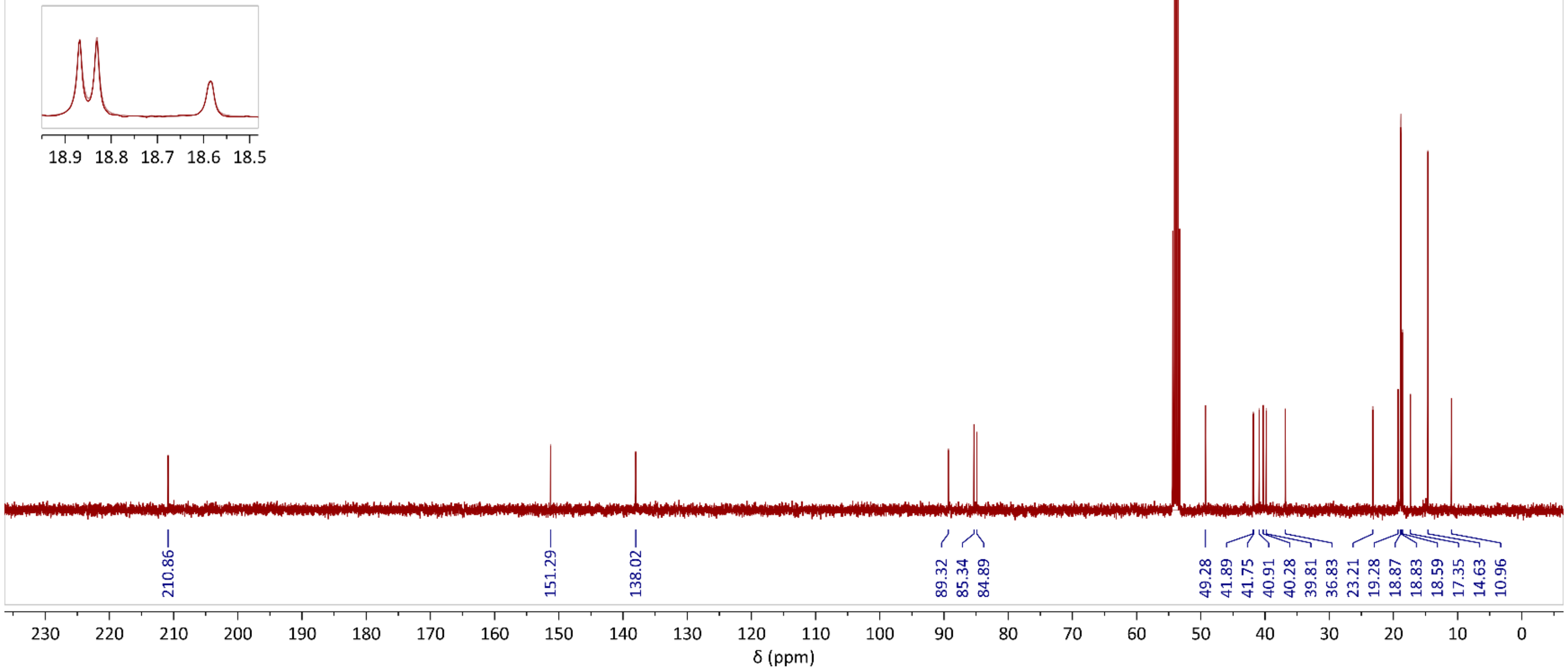

Triketone S1

^1H NMR Spectrum, $CDCl_3$, 400 MHz

Origin	Bruker BioSpin GmbH
**Solvent**	**$CDCl_3$**
**Temperature (K)**	**295.2**
**Number of Scans**	**32**
Pulse Sequence	zg30
Receiver Gain	144
Relaxation Delay (s)	1.5000
Pulse Width (µs)	11.0600
Acquisition Time (s)	1.9923
Acquisition Date	2017-10-26 21:26:00
**Nucleus**	**^1H**
**Spectrometer Freq. (MHz)**	**400.13**
Spectral Width (Hz)	8223.7
Lowest Frequency (Hz)	-1720.9
Acquired Size	16384
Spectral Size	65536

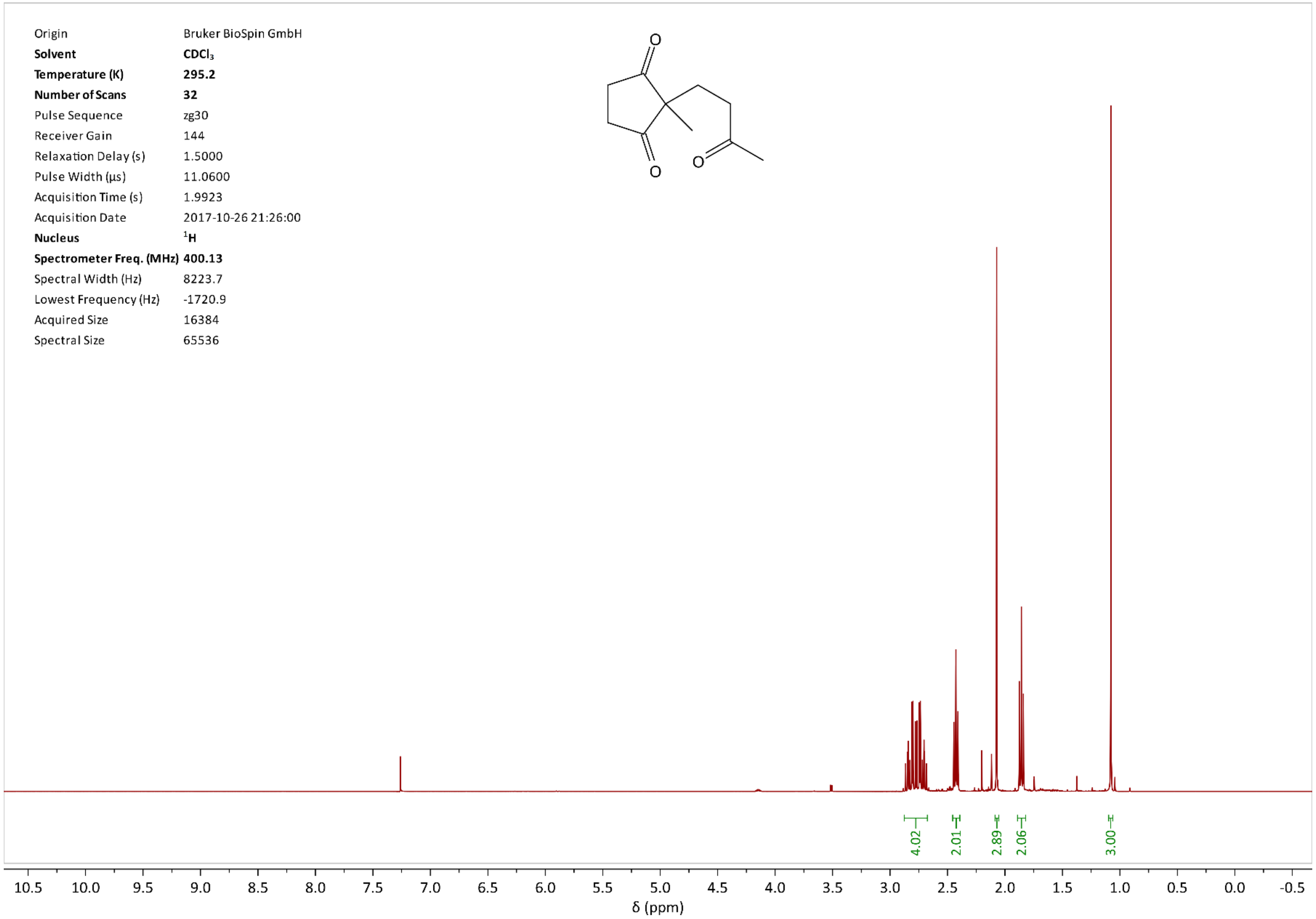

Triketone S1

^{13}C NMR Spectrum, $CDCl_3$, 101 MHz

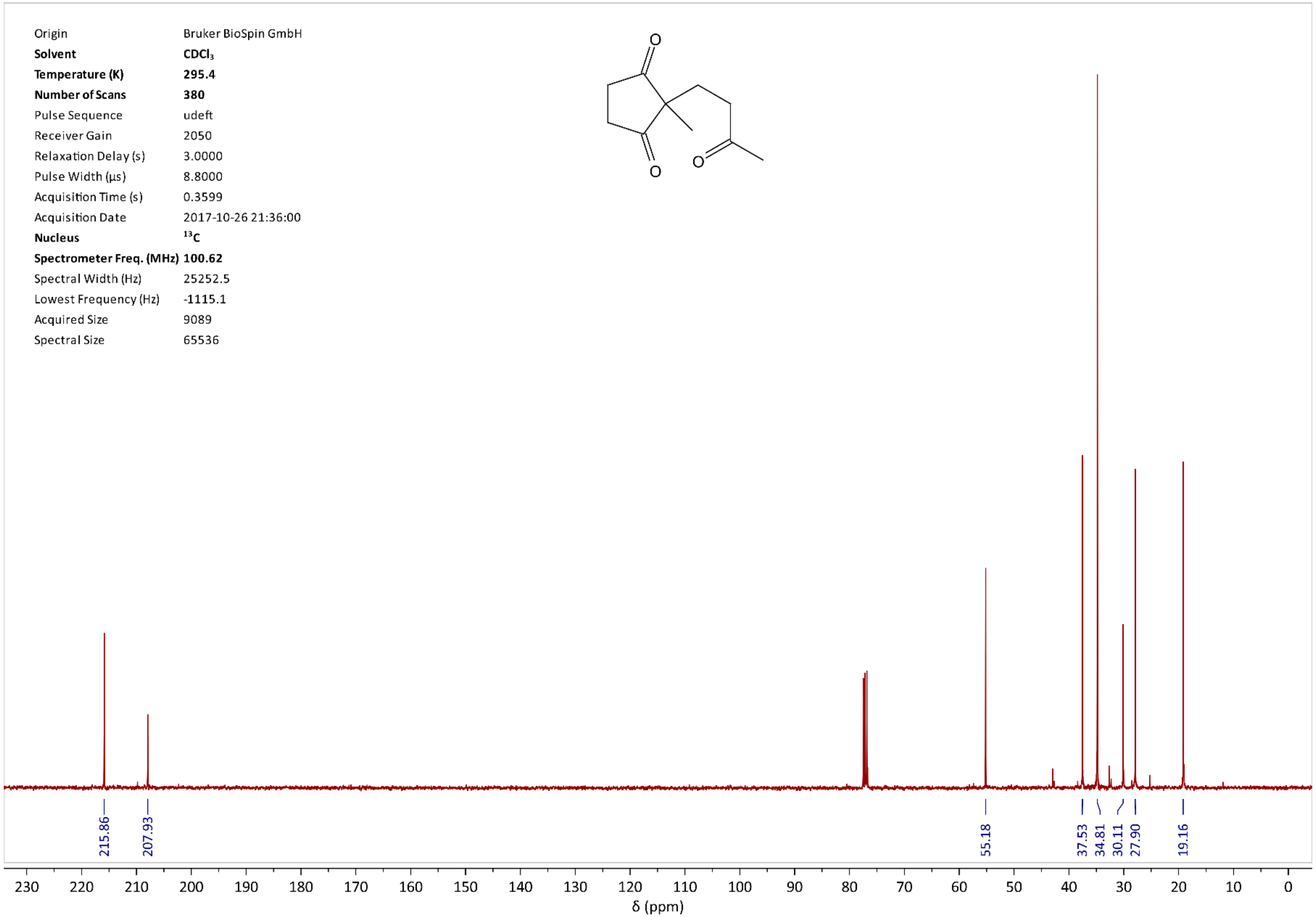

Carbonate S2

^{13}C NMR Spectrum, $CDCl_3$, 101 MHz

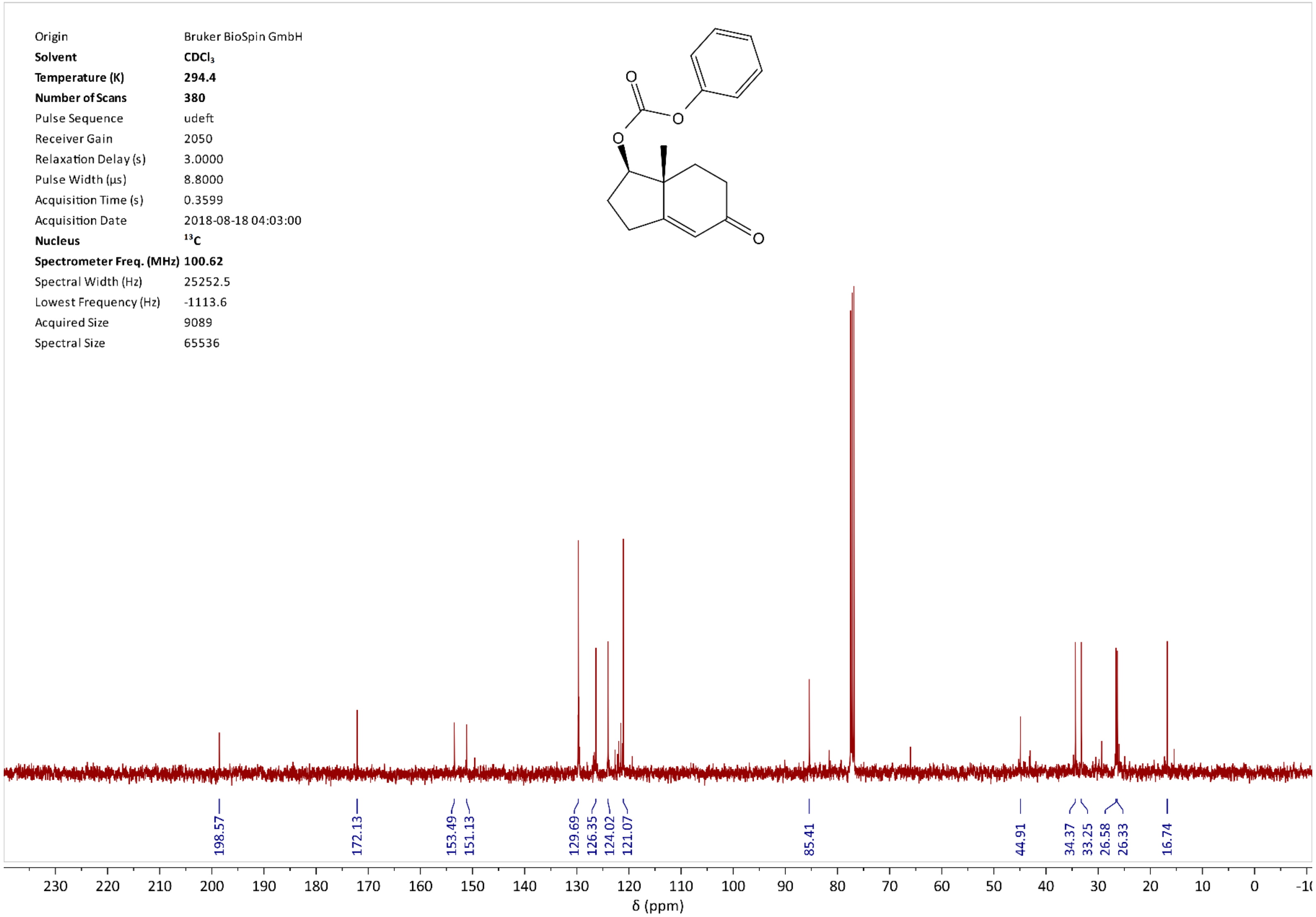

Carbonate S3

^{1}H NMR Spectrum, $CDCl_3$, 400 MHz

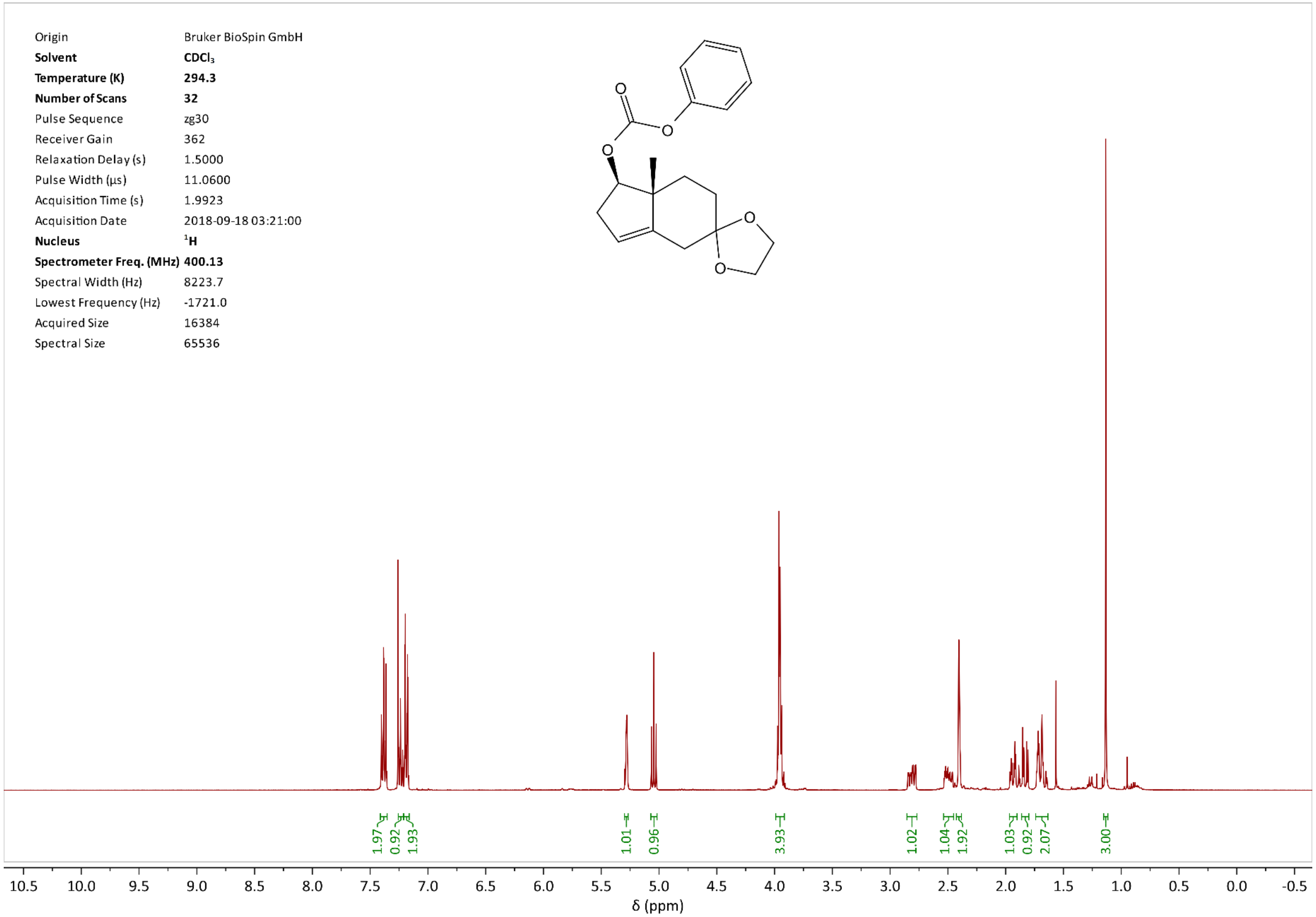

Carbonate S3

^{13}C NMR Spectrum, $CDCl_3$, 101 MHz

Origin	Bruker BioSpin GmbH
**Solvent**	**$CDCl_3$**
**Temperature (K)**	**294.3**
**Number of Scans**	**380**
Pulse Sequence	udeft
Receiver Gain	2050
Relaxation Delay (s)	3.0000
Pulse Width (μs)	8.8000
Acquisition Time (s)	0.3599
Acquisition Date	2018-09-18 03:31:00
**Nucleus**	**^{13}C**
**Spectrometer Freq. (MHz)**	**100.62**
Spectral Width (Hz)	25252.5
Lowest Frequency (Hz)	-1113.7
Acquired Size	9089
Spectral Size	65536

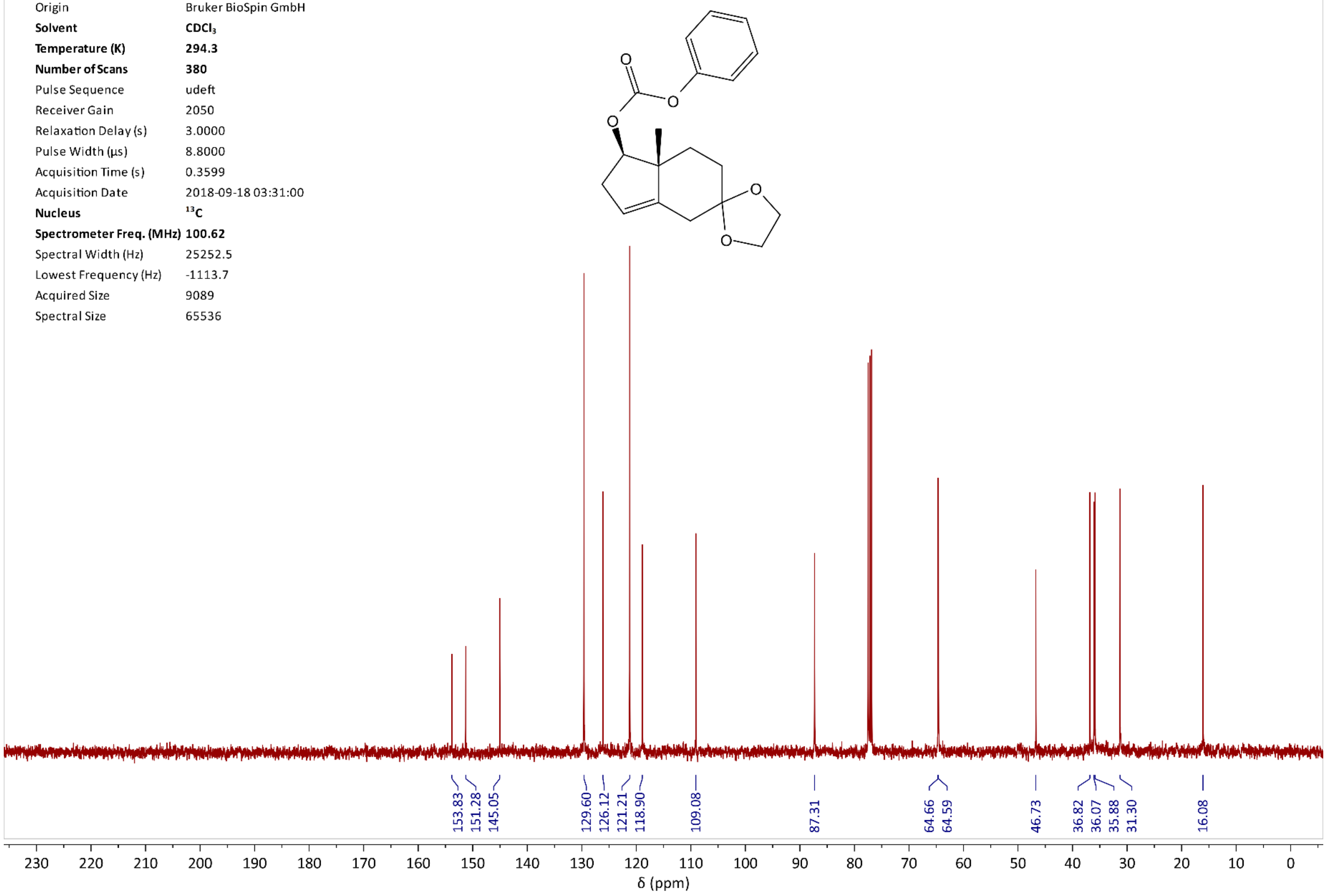

Benzyl ether S4

^{1}H NMR Spectrum, $CDCl_3$, 400 MHz

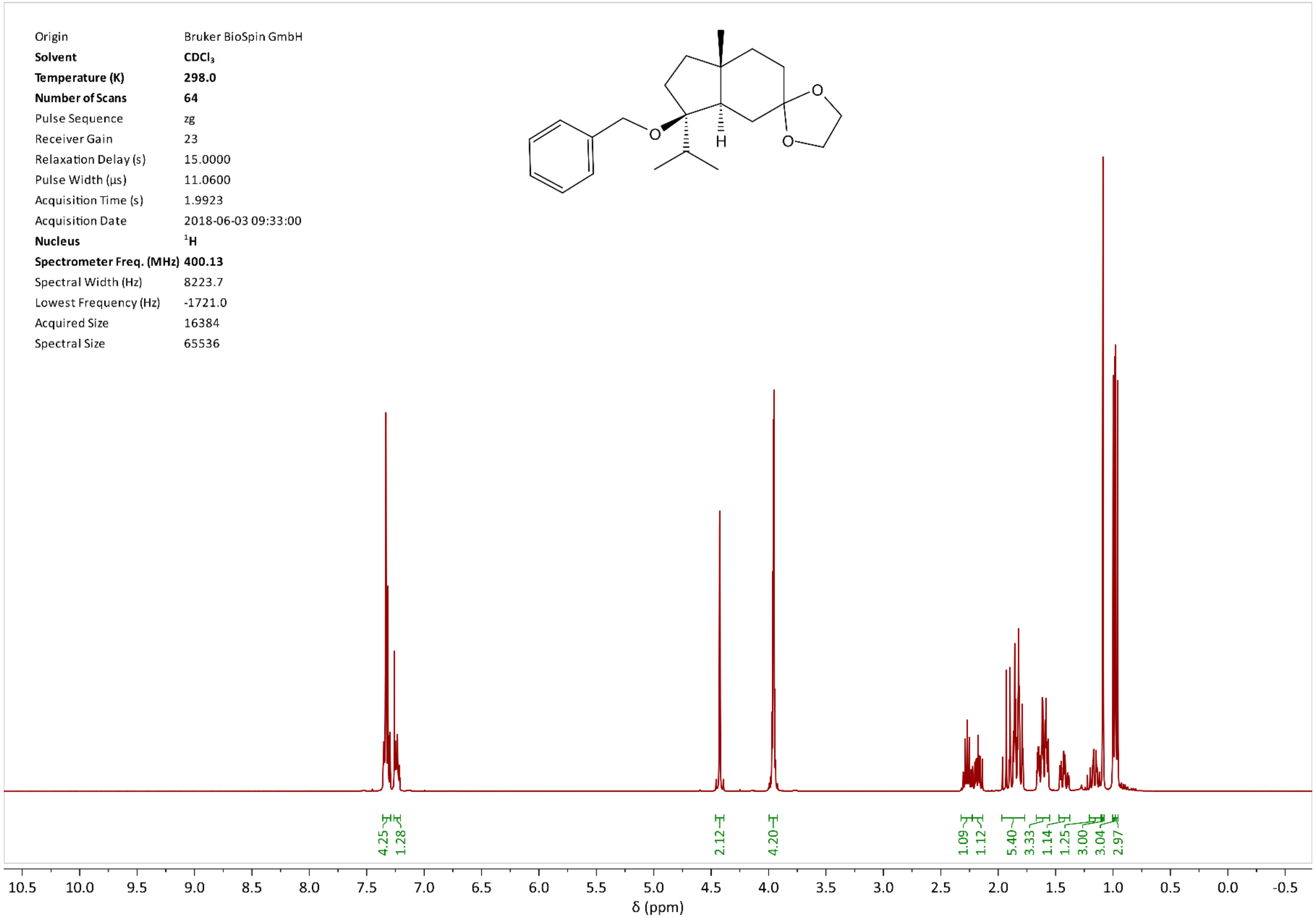

Benzyl ether S4

^{13}C NMR Spectrum, $CDCl_3$, 101 MHz

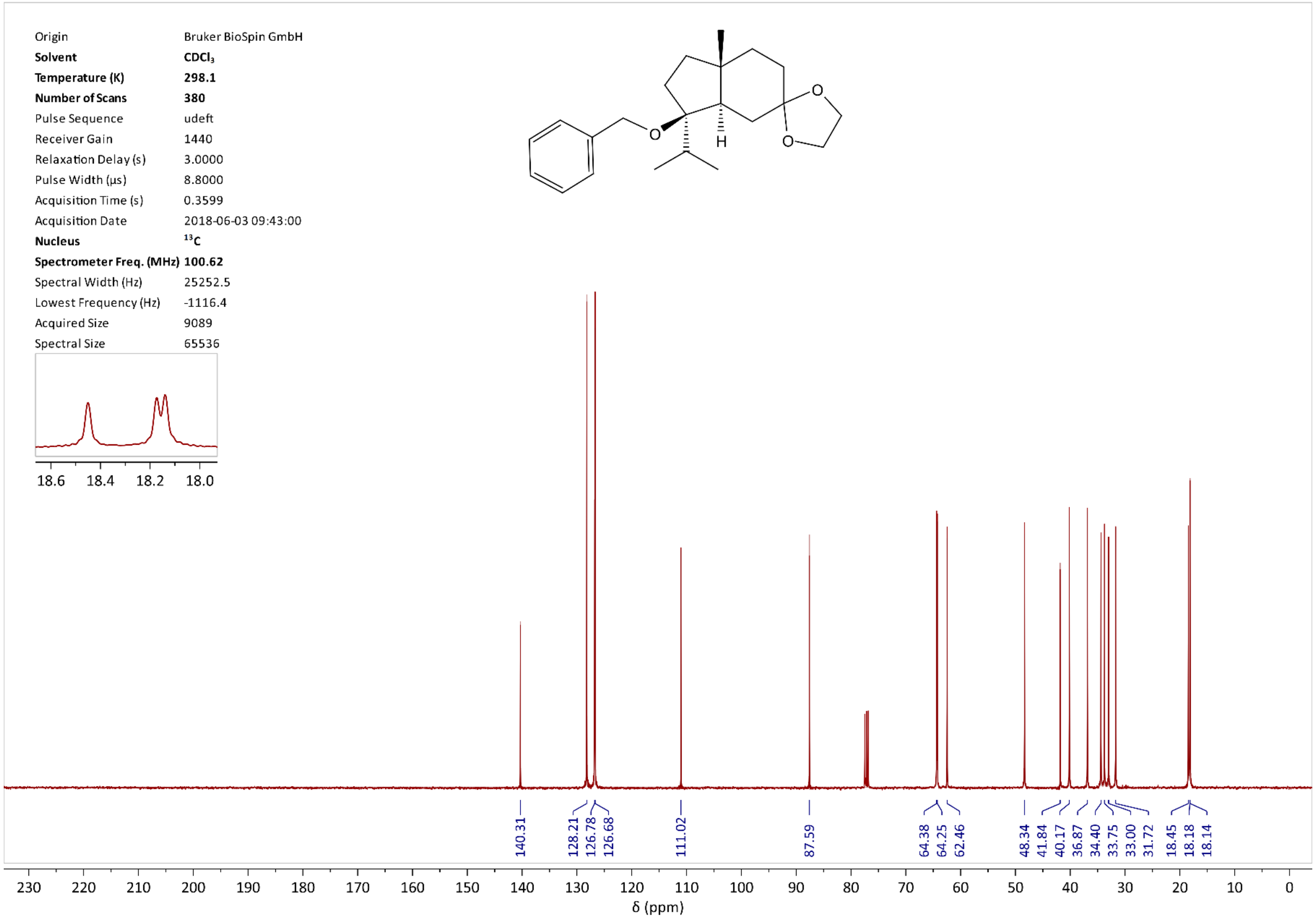

Dibenzyl acetal S6

^{1}H NMR Spectrum, $CDCl_3$, 400 MHz

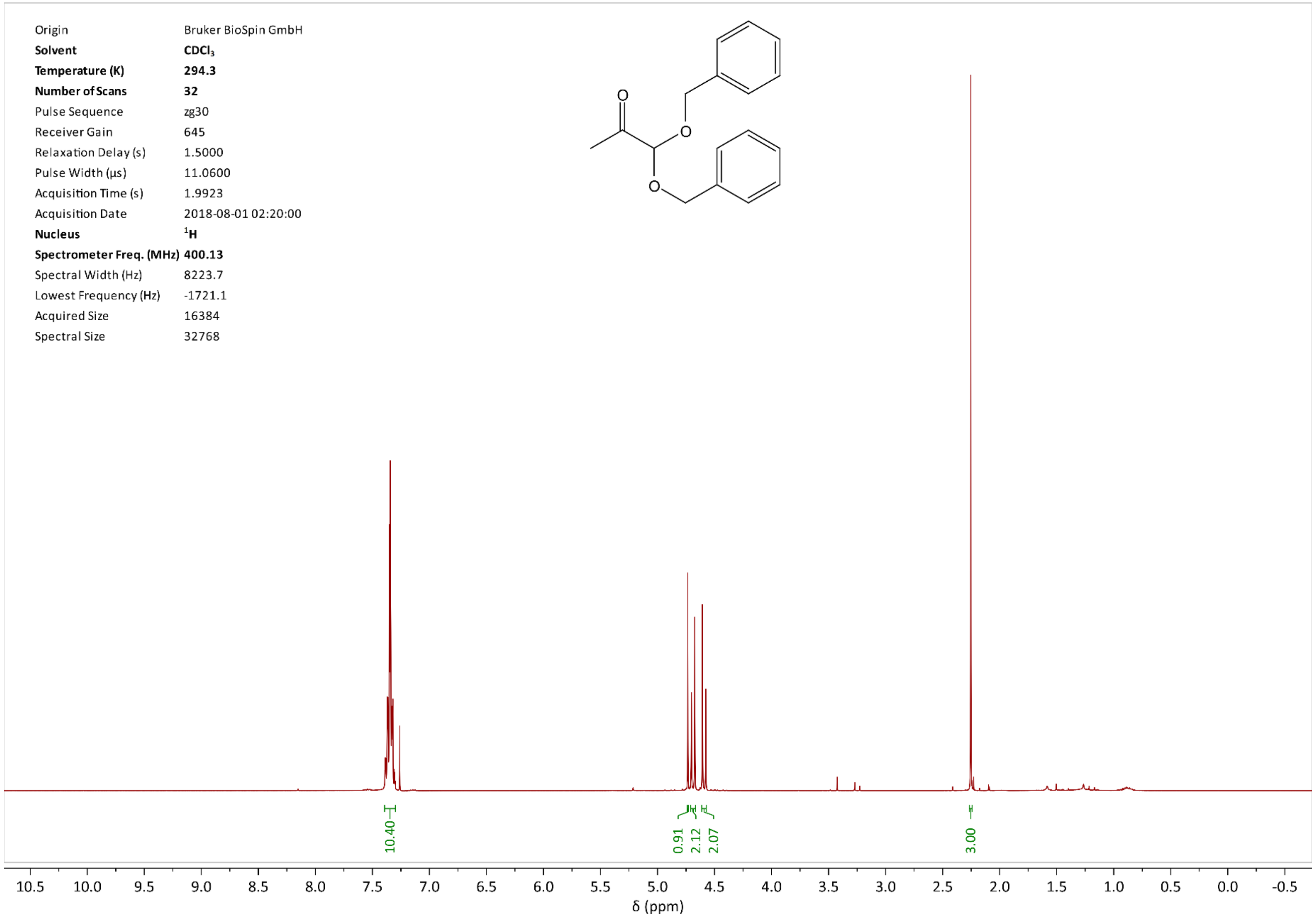

Dibenzyl acetal S6

^{13}C NMR Spectrum, $CDCl_3$, 101 MHz

Origin	Bruker BioSpin GmbH
**Solvent**	**$CDCl_3$**
**Temperature (K)**	**294.8**
**Number of Scans**	**380**
Pulse Sequence	udeft
Receiver Gain	2050
Relaxation Delay (s)	3.0000
Pulse Width (µs)	8.8000
Acquisition Time (s)	0.3599
Acquisition Date	2018-08-01 02:30:00
**Nucleus**	**^{13}C**
**Spectrometer Freq. (MHz)**	**100.61**
Spectral Width (Hz)	25252.5
Lowest Frequency (Hz)	-1112.7
Acquired Size	9089
Spectral Size	32768

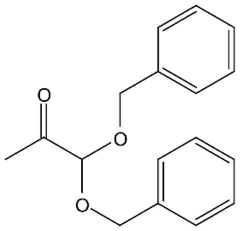

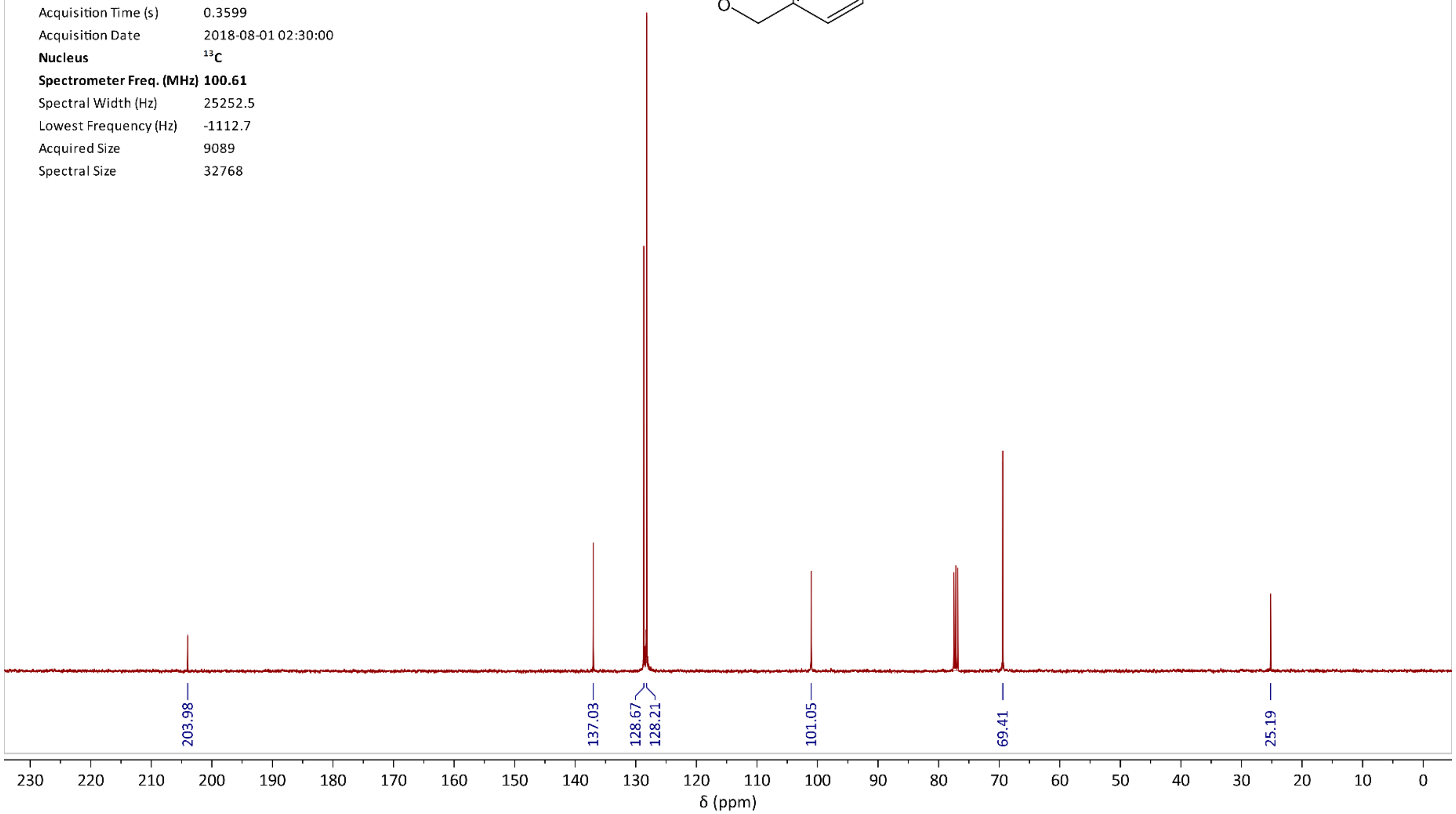

TIPS ether S7

^{1}H NMR Spectrum, $CDCl_3$, 400 MHz

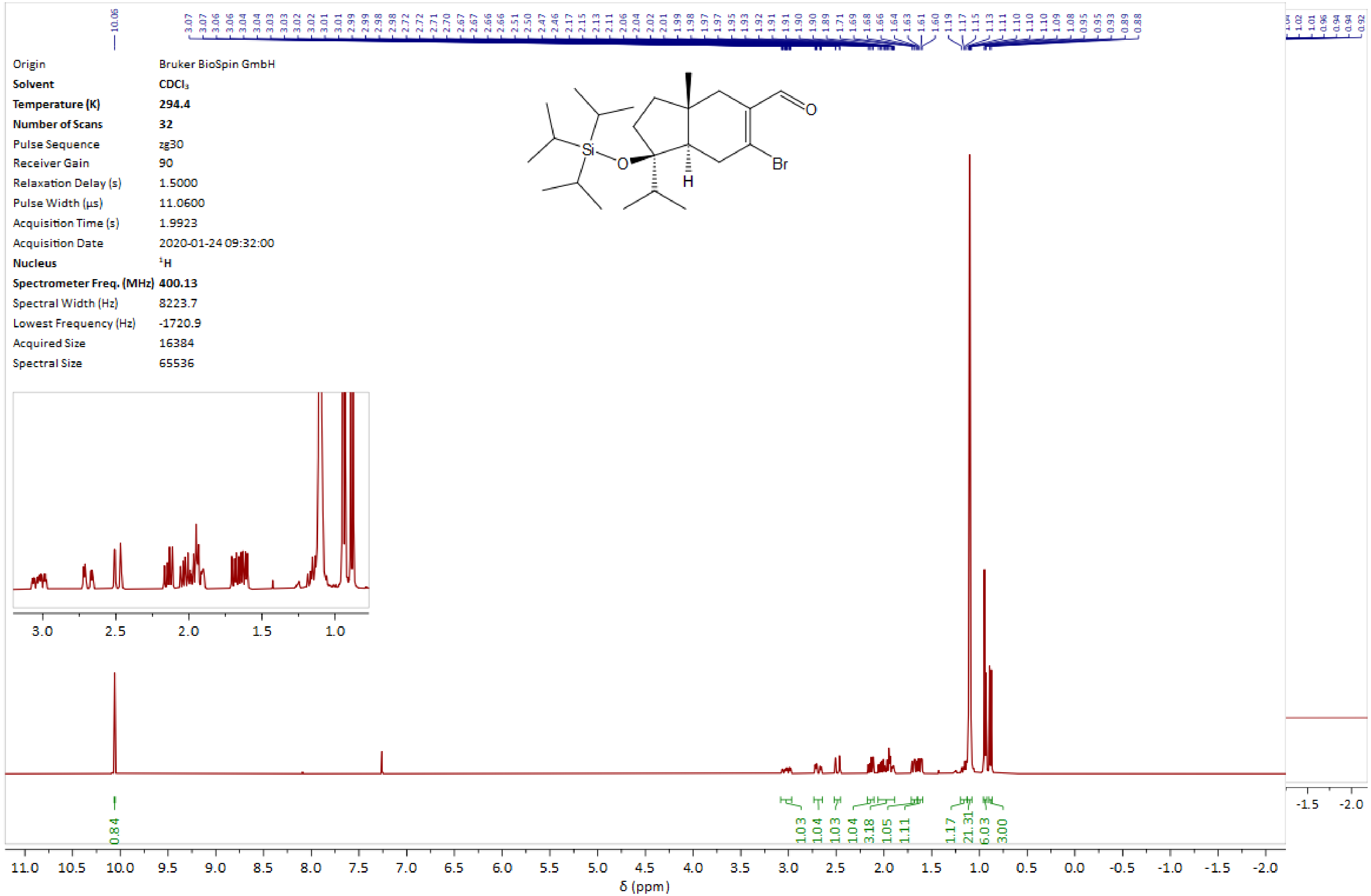
Origin Bruker BioSpin GmbH
Solvent CDCl₃
Temperature (K) 294.4
Number of Scans 32
Pulse Sequence zg30
Receiver Gain 90
Relaxation Delay (s) 1.5000
Pulse Width (μs) 11.0600
Acquisition Time (s) 1.9923
Acquisition Date 2020-01-24 09:32:00
Nucleus ¹H
Spectrometer Freq. (MHz) 400.13
Spectral Width (Hz) 8223.7
Lowest Frequency (Hz) -1720.9
Acquired Size 16384
Spectral Size 65536
Si
O
H
Br
O
10.06
0.84
1.03
1.04
1.03
1.04
3.18
1.05
1.11
1.17
21.31
6.03
3.00
δ (ppm)

TIPS ether S7

^{13}C NMR Spectrum, $CDCl_3$, 101 MHz

Origin	Bruker BioSpin GmbH
**Solvent**	**$CDCl_3$**
**Temperature (K)**	**298.0**
**Number of Scans**	**512**
Pulse Sequence	zgpg30
Receiver Gain	35
Relaxation Delay (s)	1.0000
Pulse Width (µs)	10.0000
Acquisition Time (s)	2.3809
Acquisition Date	2019-05-08 06:30:02
**Nucleus**	**^{13}C**
**Spectrometer Freq. (MHz)**	**100.62**
Spectral Width (Hz)	25000.0
Lowest Frequency (Hz)	-915.4
Acquired Size	59522
Spectral Size	131072

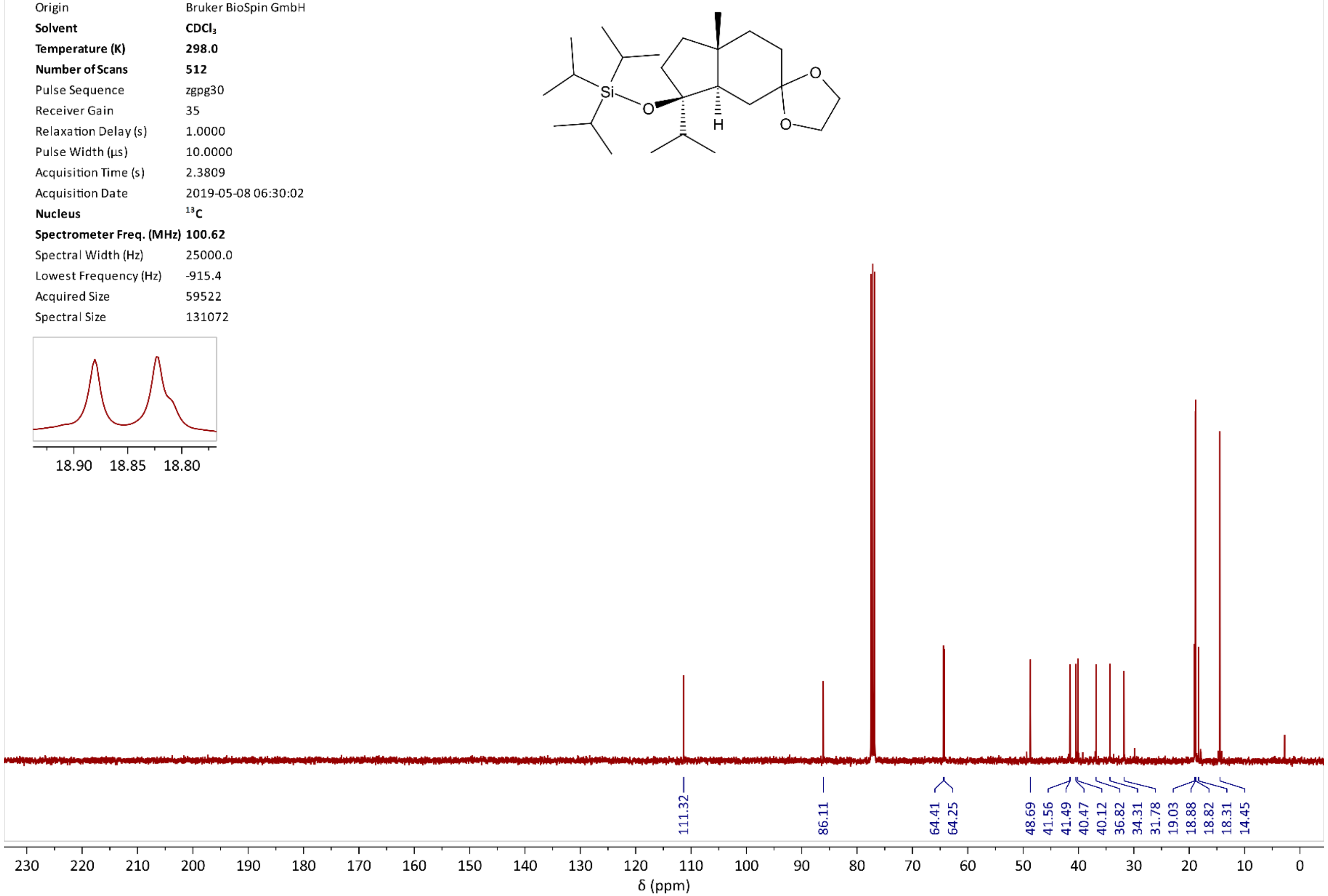

Dimethylamine S10

^{1}H NMR Spectrum, $CDCl_3$, 400 MHz

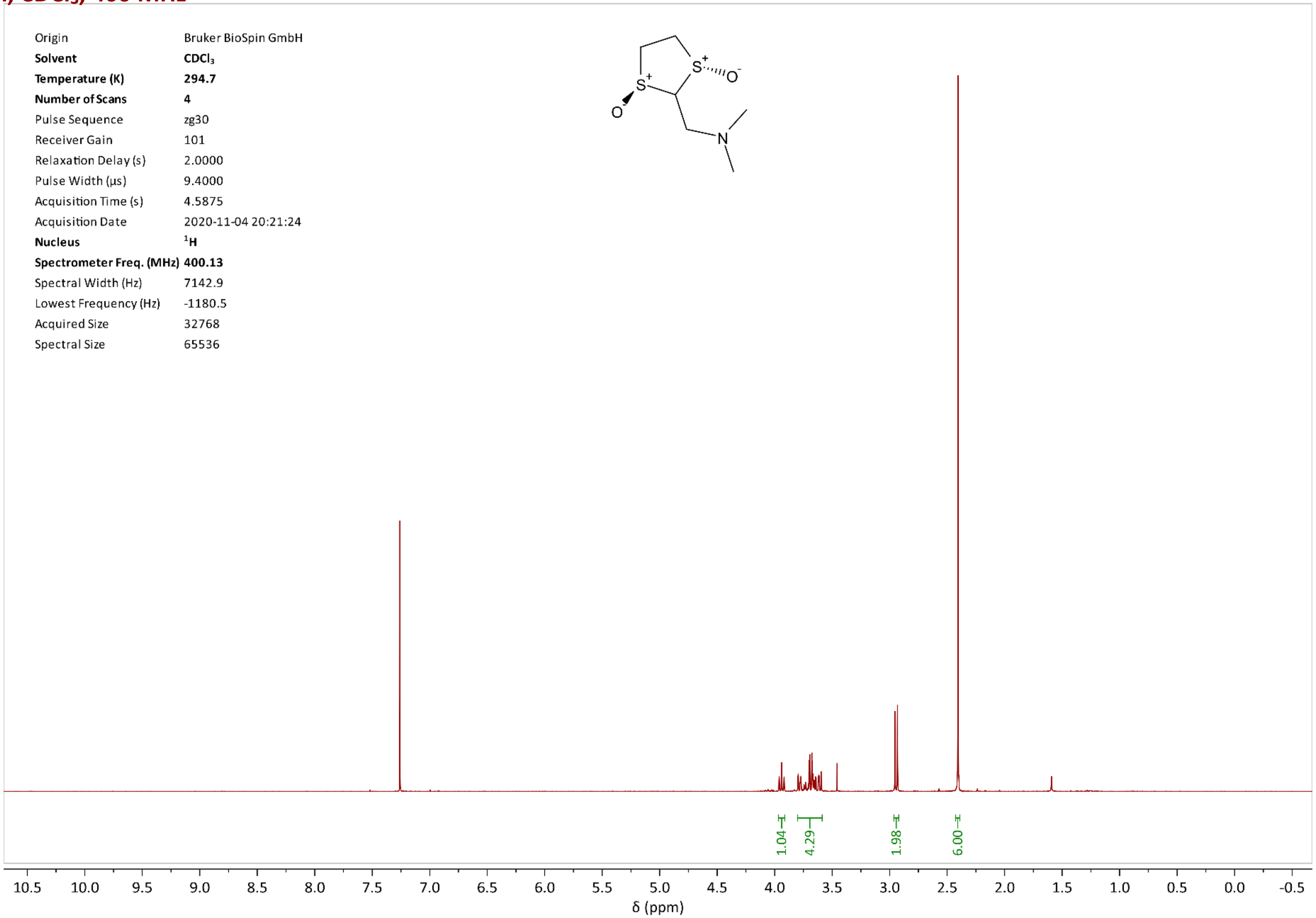

Dimethylamine S10

^{13}C NMR Spectrum, $CDCl_3$, 101 MHz

Origin	Bruker BioSpin GmbH
**Solvent**	**$CDCl_3$**
**Temperature (K)**	**295.1**
**Number of Scans**	**512**
Pulse Sequence	zgpg30
Receiver Gain	15
Relaxation Delay (s)	1.0000
Pulse Width (μs)	10.0000
Acquisition Time (s)	2.3809
Acquisition Date	2020-11-04 20:20:36
**Nucleus**	**^{13}C**
**Spectrometer Freq. (MHz)**	**100.62**
Spectral Width (Hz)	25000.0
Lowest Frequency (Hz)	-917.1
Acquired Size	59522
Spectral Size	131072

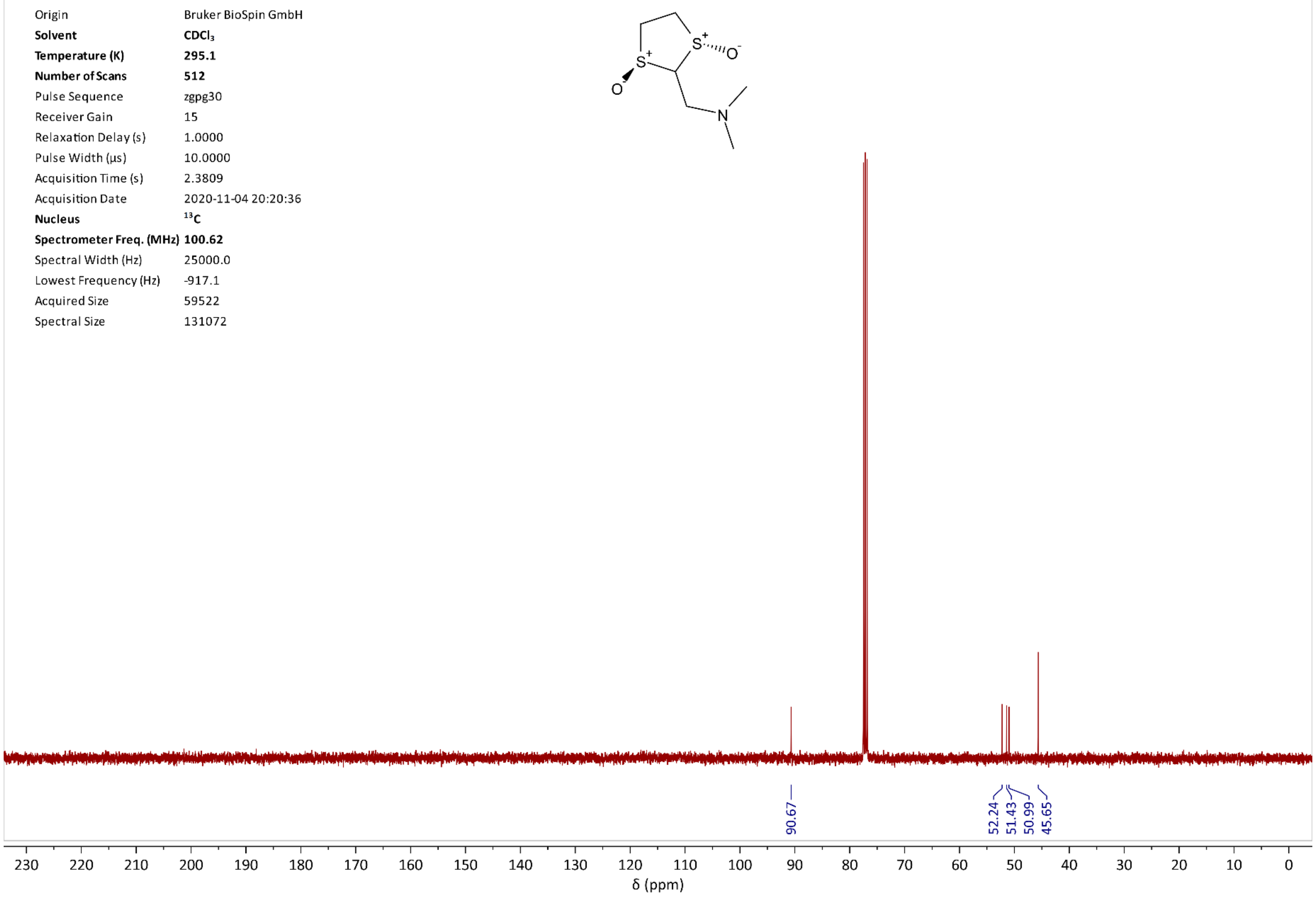

(*S*,*S*)-Bissulfoxide S11

^{1}H NMR Spectrum, $CDCl_3$, 400 MHz

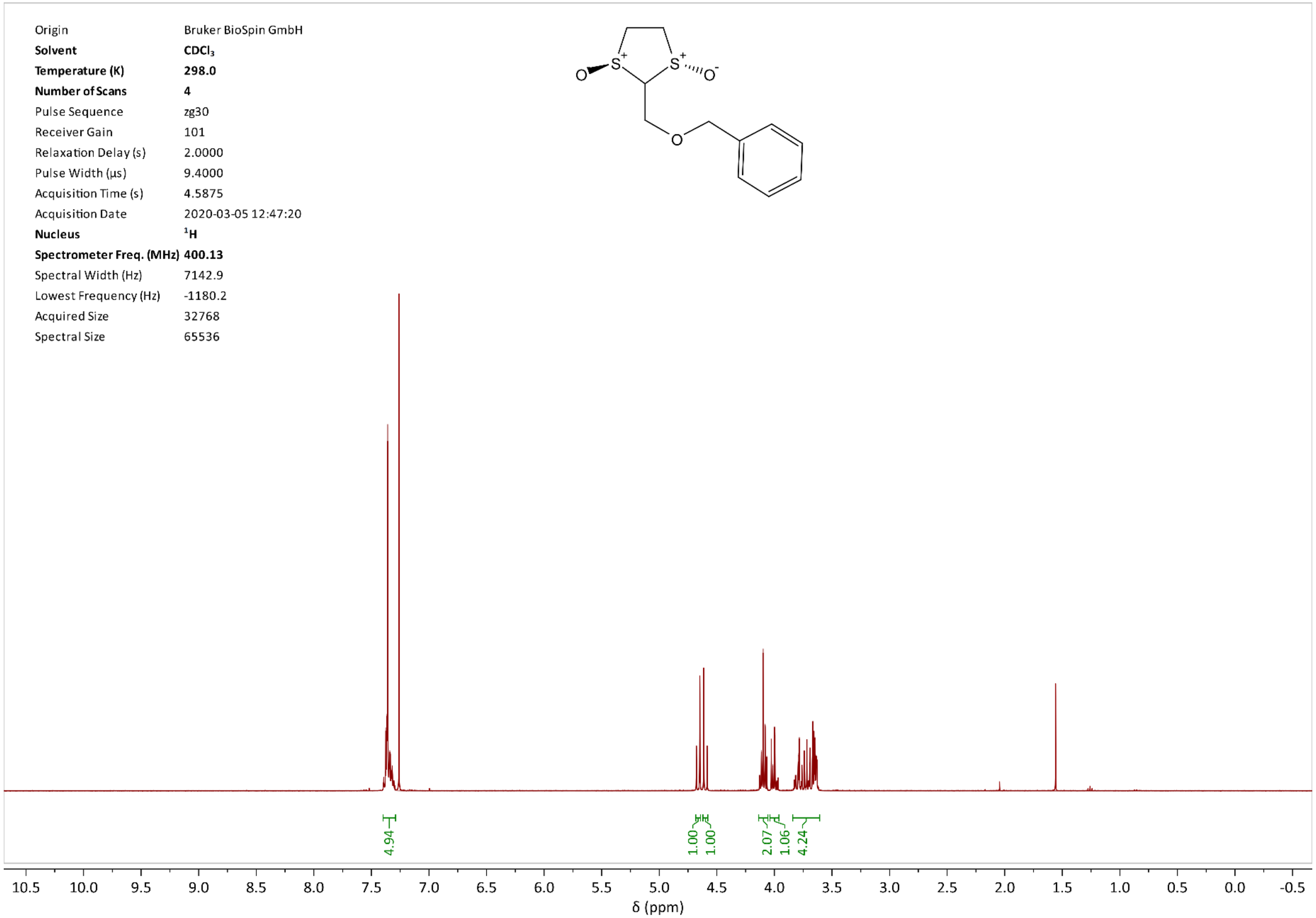

(*S*,*S*)-Bissulfoxide S11

^{13}C NMR Spectrum, $CDCl_3$, 101 MHz

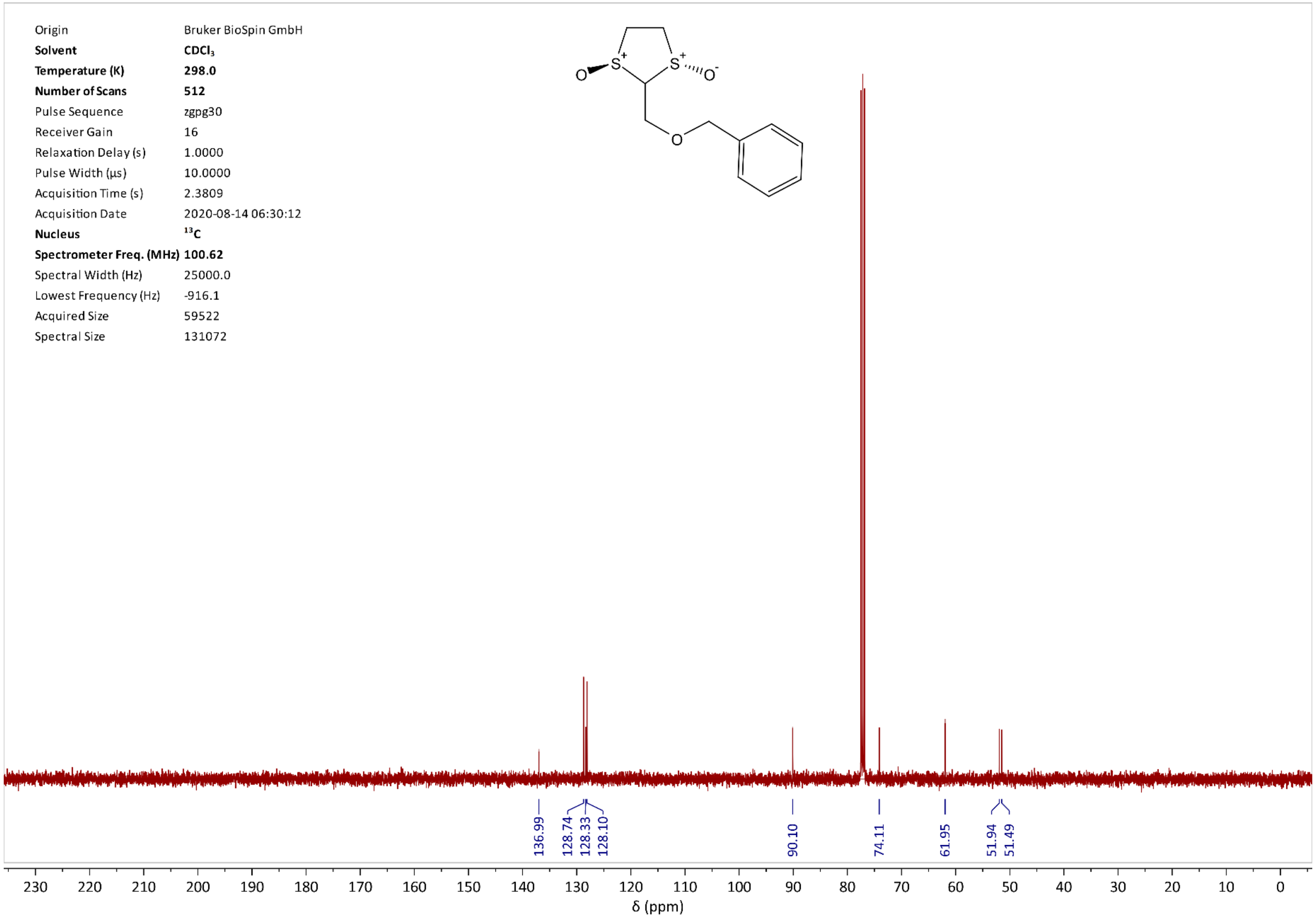

Benzyl ether S12

^{1}H NMR Spectrum, $CDCl_3$, 400 MHz

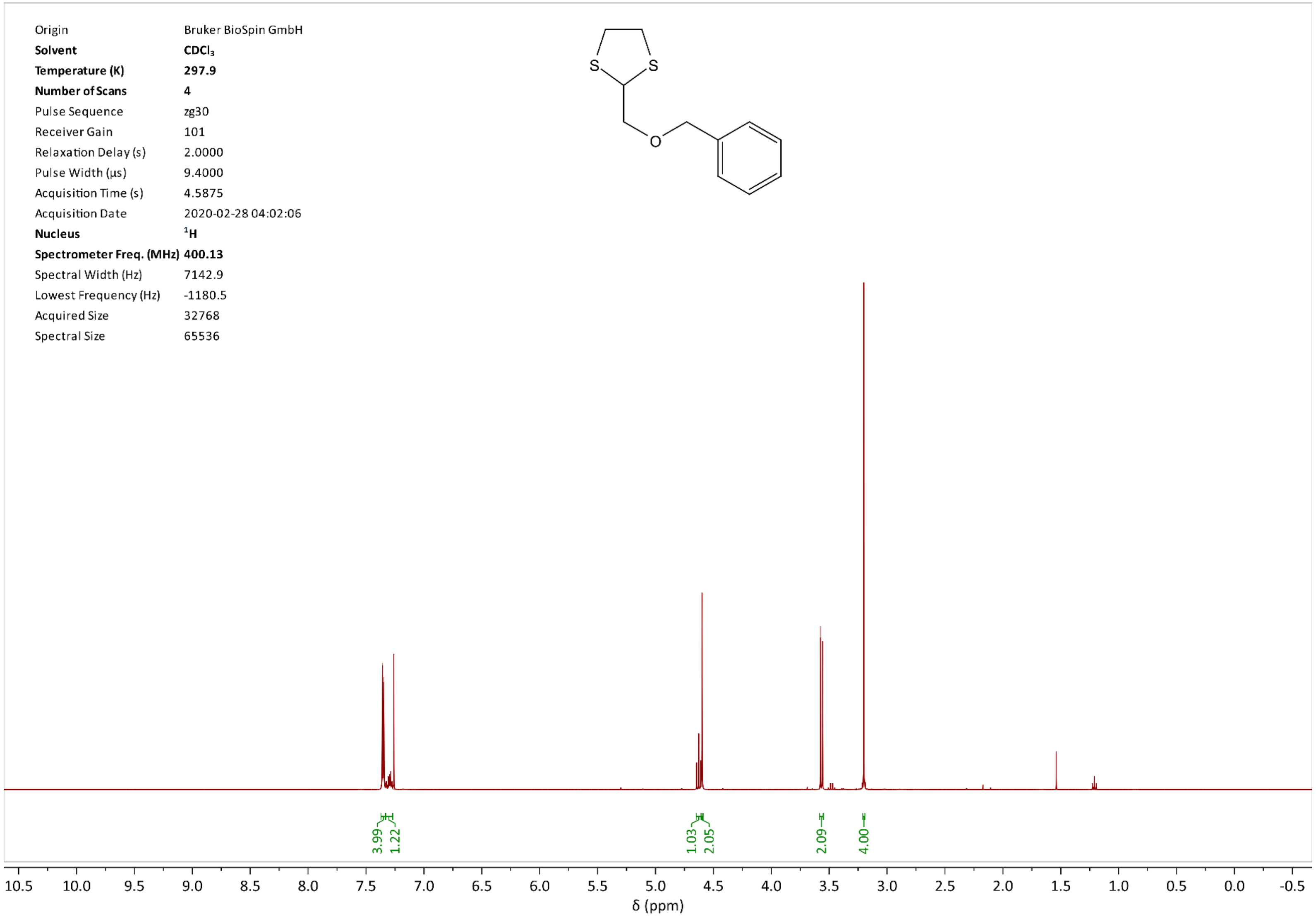

Benzyl ether S12

^{13}C NMR Spectrum, $CDCl_3$, 101 MHz

Origin	Bruker BioSpin GmbH
**Solvent**	**$CDCl_3$**
**Temperature (K)**	**298.0**
**Number of Scans**	**512**
Pulse Sequence	zgpg30
Receiver Gain	17
Relaxation Delay (s)	1.0000
Pulse Width (μs)	10.0000
Acquisition Time (s)	2.3809
Acquisition Date	2020-02-28 03:42:25
**Nucleus**	**^{13}C**
**Spectrometer Freq. (MHz)**	**100.62**
Spectral Width (Hz)	25000.0
Lowest Frequency (Hz)	-916.3
Acquired Size	59522
Spectral Size	131072

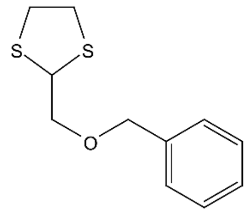

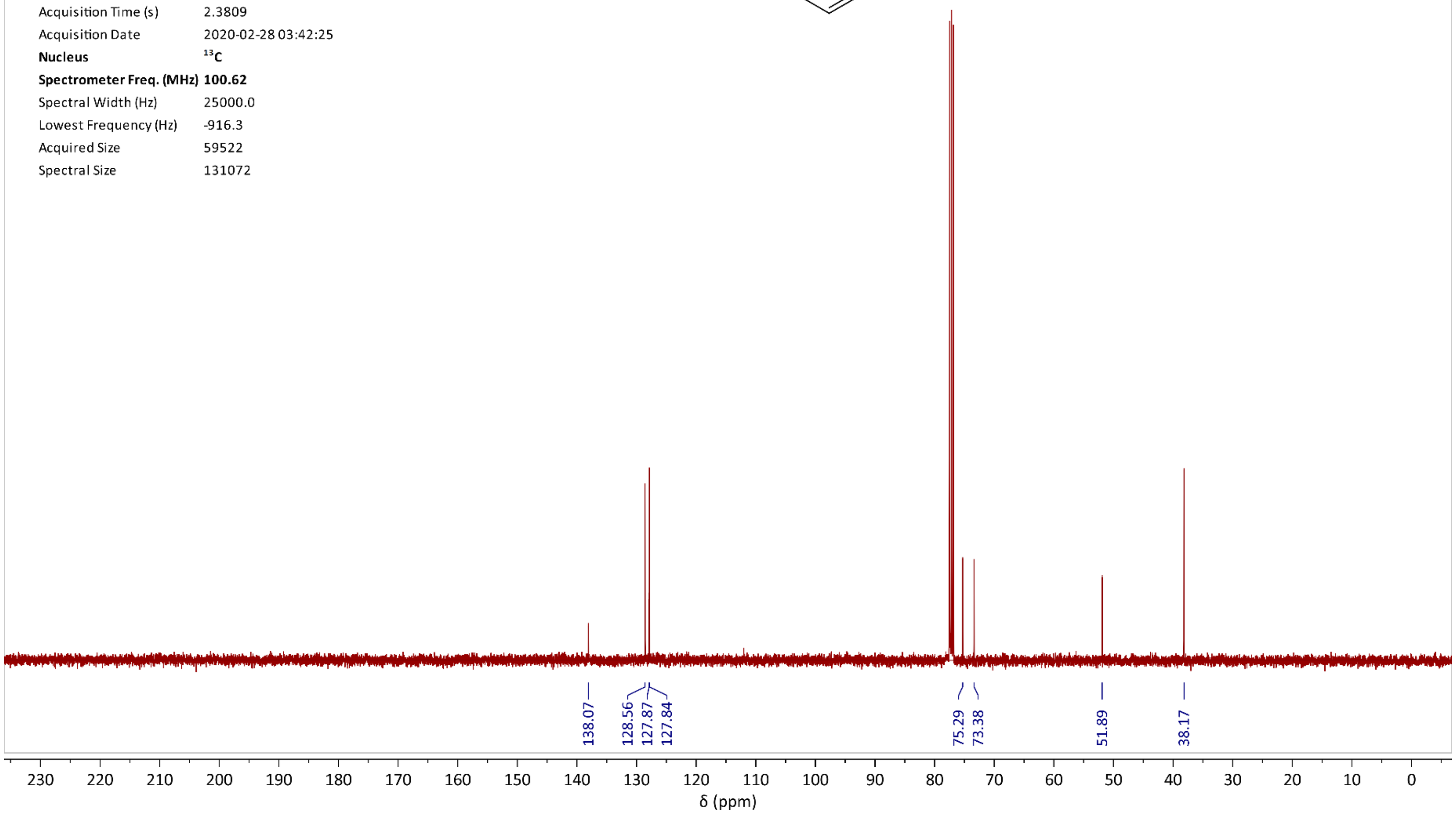

1-Bromo-1-methoxypropan-2-one S14

^{1}H NMR Spectrum, $CDCl_3$, 400 MHz

Origin	Bruker BioSpin GmbH
**Solvent**	**$CDCl_3$**
**Temperature (K)**	**297.9**
**Number of Scans**	**2**
Pulse Sequence	zg30
Receiver Gain	101
Relaxation Delay (s)	2.0000
Pulse Width (μs)	10.0000
Acquisition Time (s)	4.5875
Acquisition Date	2019-06-17 18:27:14
**Nucleus**	**^{1}H**
**Spectrometer Freq. (MHz)**	**400.13**
Spectral Width (Hz)	7142.9
Lowest Frequency (Hz)	-1180.2
Acquired Size	32768
Spectral Size	65536

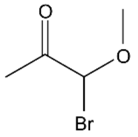

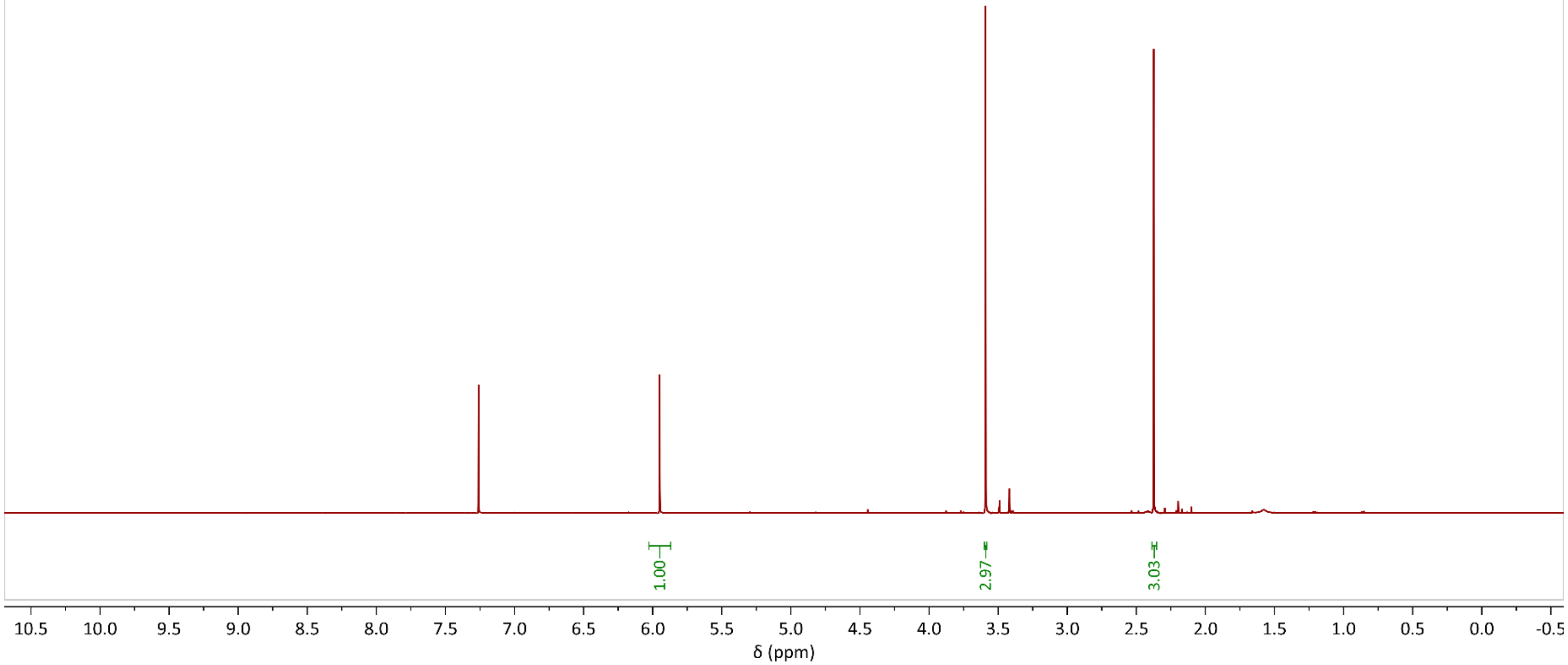

1-Bromo-1-methoxypropan-2-one S14

^{13}C NMR Spectrum, $CDCl_3$, 101 MHz

Origin	Bruker BioSpin GmbH
**Solvent**	**$CDCl_3$**
**Temperature (K)**	**298.0**
**Number of Scans**	**512**
Pulse Sequence	zgpg30
Receiver Gain	32
Relaxation Delay (s)	1.0000
Pulse Width (μs)	10.0000
Acquisition Time (s)	2.3809
Acquisition Date	2019-06-17 18:06:45
**Nucleus**	**^{13}C**
**Spectrometer Freq. (MHz)**	**100.62**
Spectral Width (Hz)	25000.0
Lowest Frequency (Hz)	-915.7
Acquired Size	59522
Spectral Size	131072

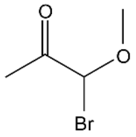

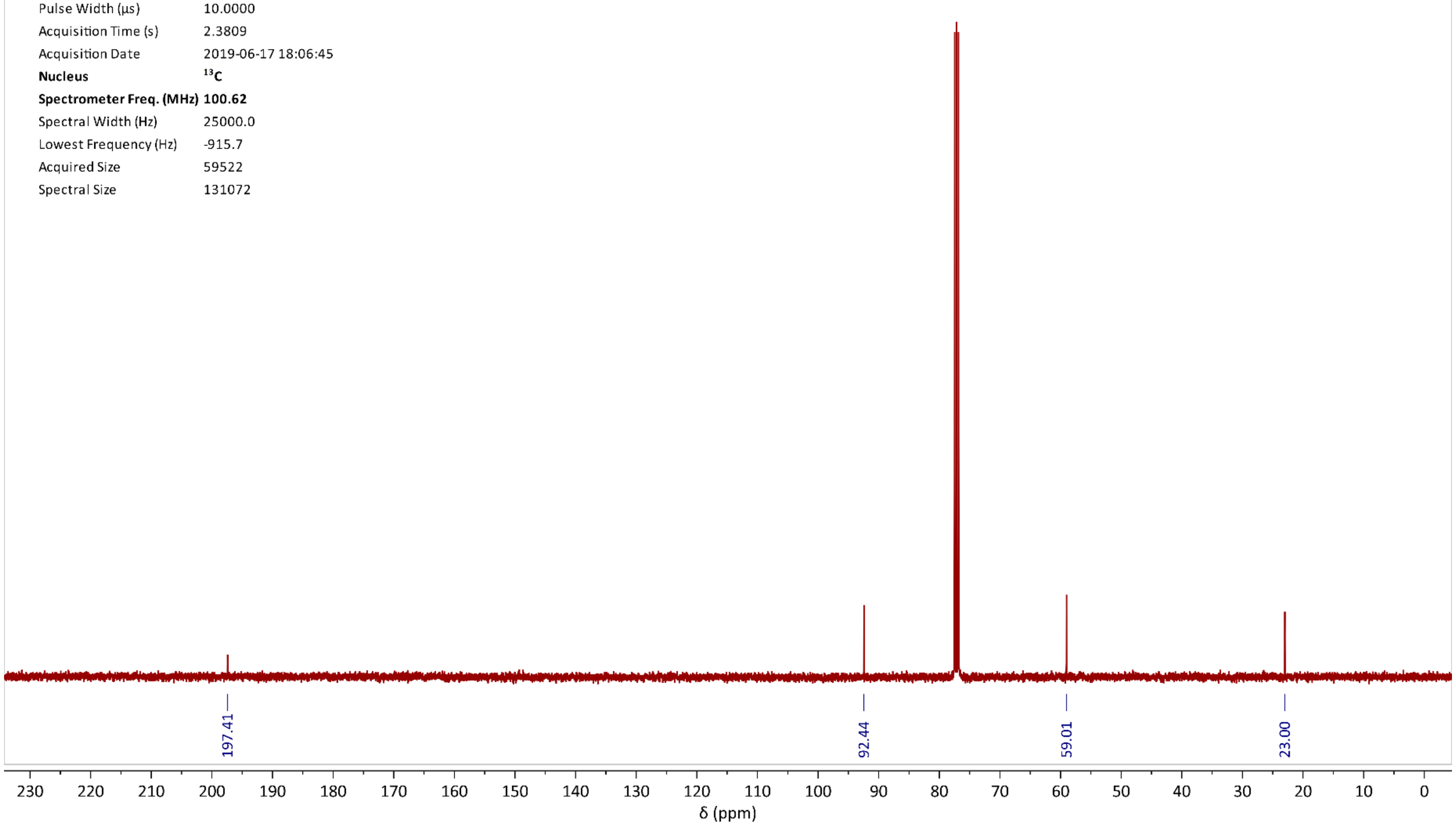

1-Methoxy-1-((R)-1-(naphthalen-2-yl)ethoxy)propan-2-one S15

^{1}H NMR Spectrum, $CDCl_3$, 400 MHz

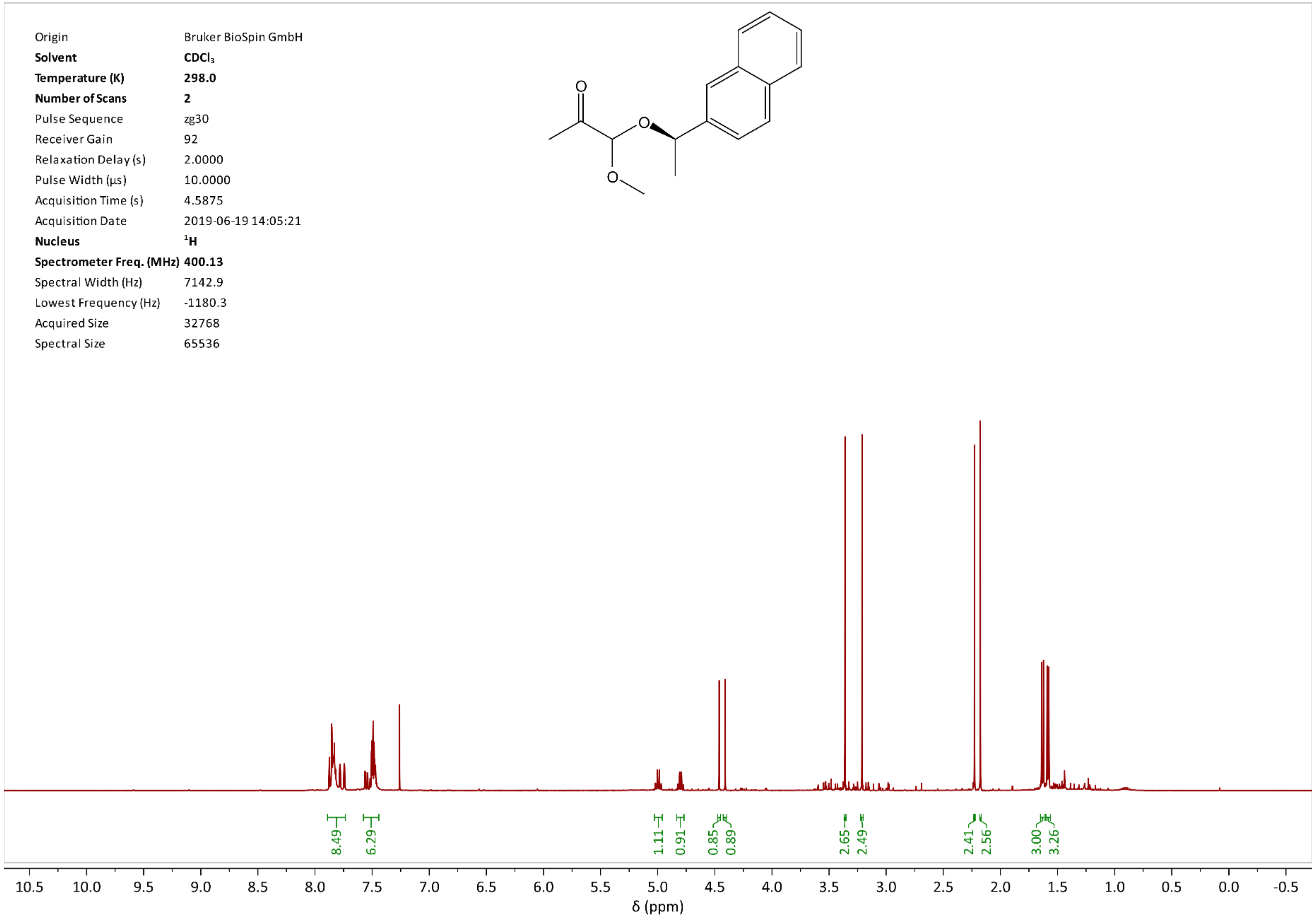

1-Methoxy-1-((*R*)-1-(naphthalen-2-yl)ethoxy)propan-2-one S15

^{13}C NMR Spectrum, $CDCl_3$, 101 MHz

Origin	Bruker BioSpin GmbH
**Solvent**	**$CDCl_3$**
**Temperature (K)**	**298.0**
**Number of Scans**	**512**
Pulse Sequence	zgpg30
Receiver Gain	32
Relaxation Delay (s)	1.0000
Pulse Width (µs)	10.0000
Acquisition Time (s)	2.3809
Acquisition Date	2019-06-19 23:08:50
**Nucleus**	**^{13}C**
**Spectrometer Freq. (MHz)**	**100.62**
Spectral Width (Hz)	25000.0
Lowest Frequency (Hz)	-917.6
Acquired Size	59522
Spectral Size	131072

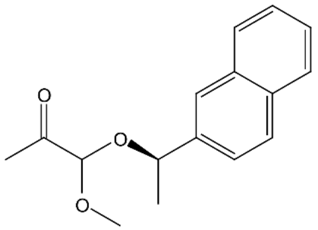

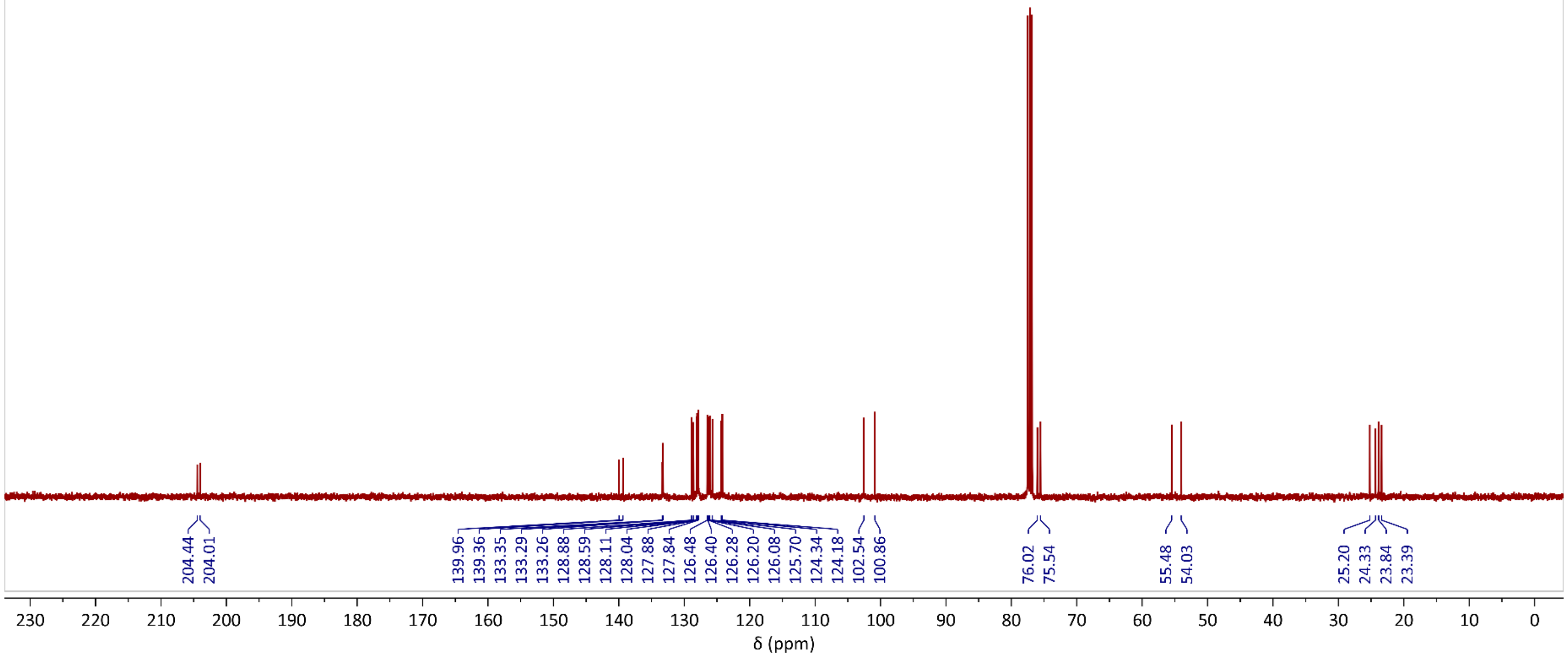

Triethyl((3-methoxy-3-((R)-1-(naphthalen-2-yl)ethoxy)prop-1-en-2-yl)oxy)silane S16

^{1}H NMR Spectrum, CD_2Cl_2, 400 MHz

Origin	Bruker BioSpin GmbH
**Solvent**	**CD_2Cl_2**
**Temperature (K)**	**298.0**
**Number of Scans**	**2**
Pulse Sequence	zg30
Receiver Gain	100
Relaxation Delay (s)	2.0000
Pulse Width (μs)	10.0000
Acquisition Time (s)	4.5875
Acquisition Date	2019-07-16 12:26:15
**Nucleus**	**^{1}H**
**Spectrometer Freq. (MHz)**	**400.13**
Spectral Width (Hz)	7142.9
Lowest Frequency (Hz)	-1186.0
Acquired Size	32768
Spectral Size	65536

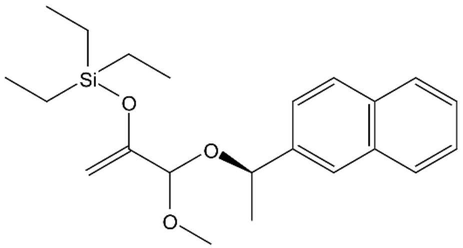

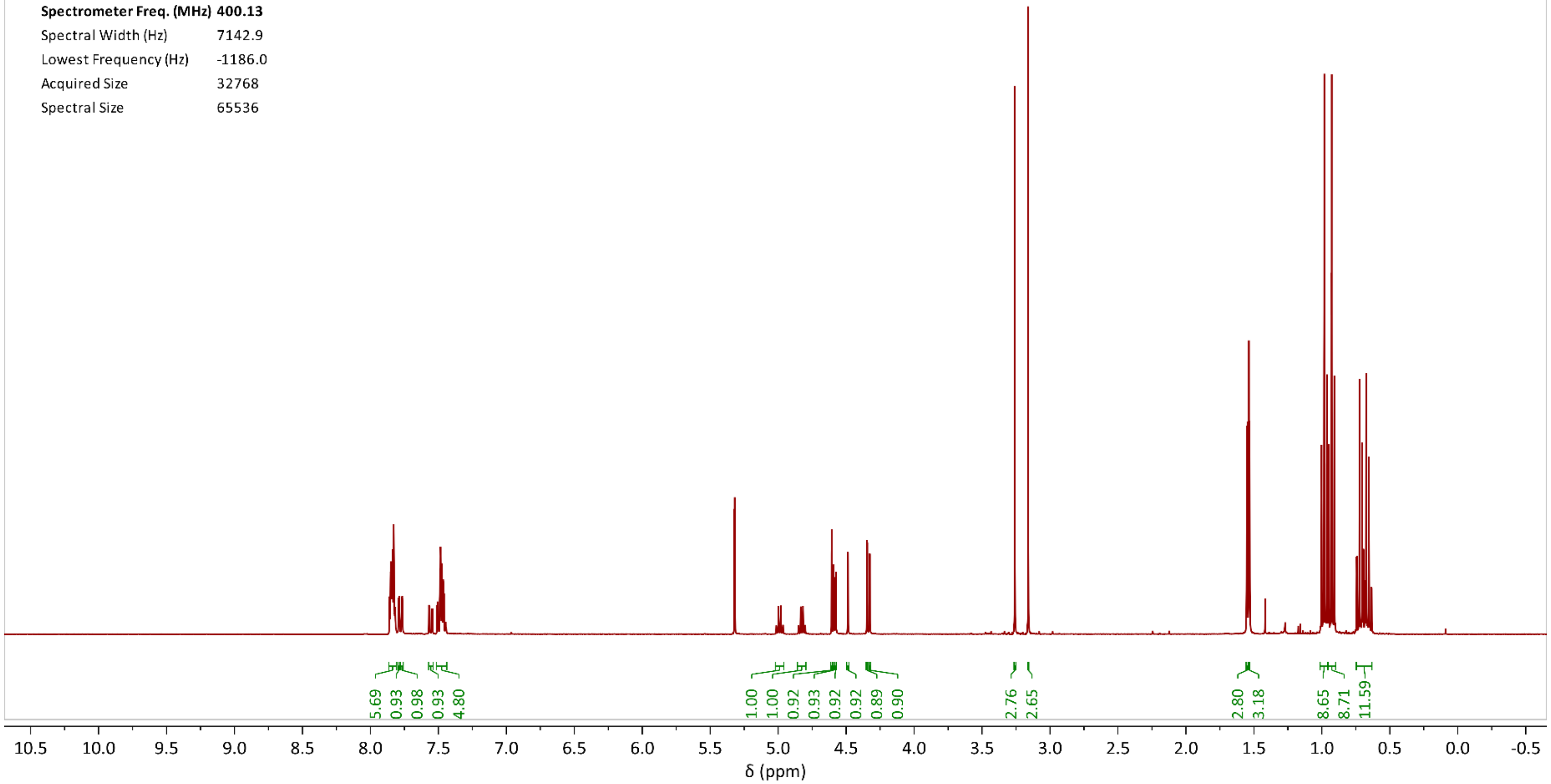

Triethyl((3-methoxy-3-((*R*)-1-(naphthalen-2-yl)ethoxy)prop-1-en-2-yl)oxy)silane S16

^{13}C NMR Spectrum, CD_2Cl_2, 101 MHz

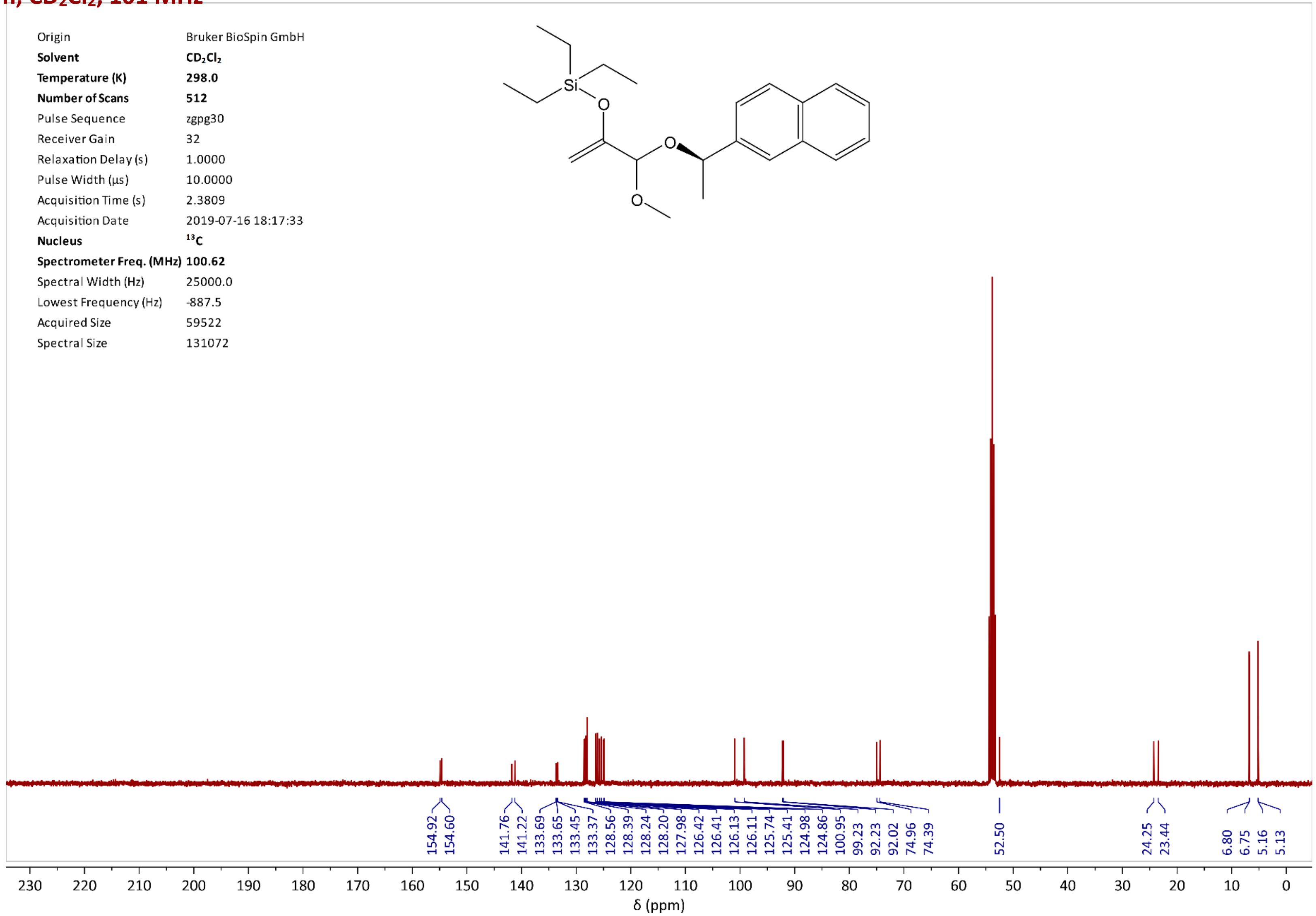

Tosylate S17

^{1}H NMR Spectrum, $CDCl_3$, 400 MHz

Origin	Bruker BioSpin GmbH
**Solvent**	**$CDCl_3$**
**Temperature (K)**	**293.9**
**Number of Scans**	**32**
Pulse Sequence	zg30
Receiver Gain	161
Relaxation Delay (s)	1.5000
Pulse Width (μs)	9.5000
Acquisition Time (s)	1.9923
Acquisition Date	2013-11-04 17:46:00
**Nucleus**	**^{1}H**
**Spectrometer Freq. (MHz)**	**400.13**
Spectral Width (Hz)	8223.7
Lowest Frequency (Hz)	-1673.3
Acquired Size	16384
Spectral Size	65536

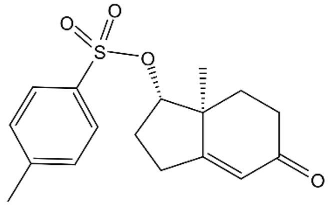

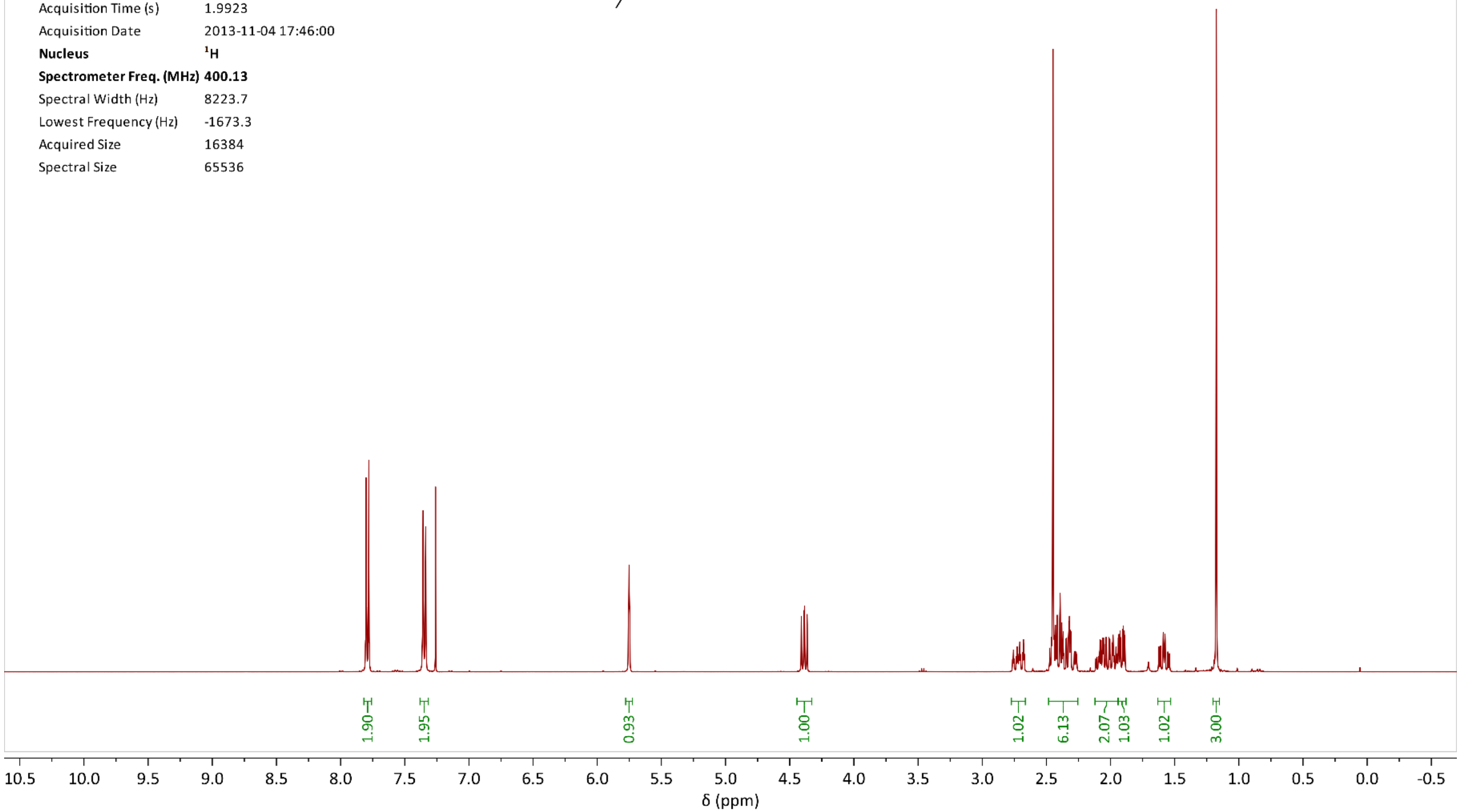

Tosylate S17

^{13}C NMR Spectrum, $CDCl_3$, 101 MHz

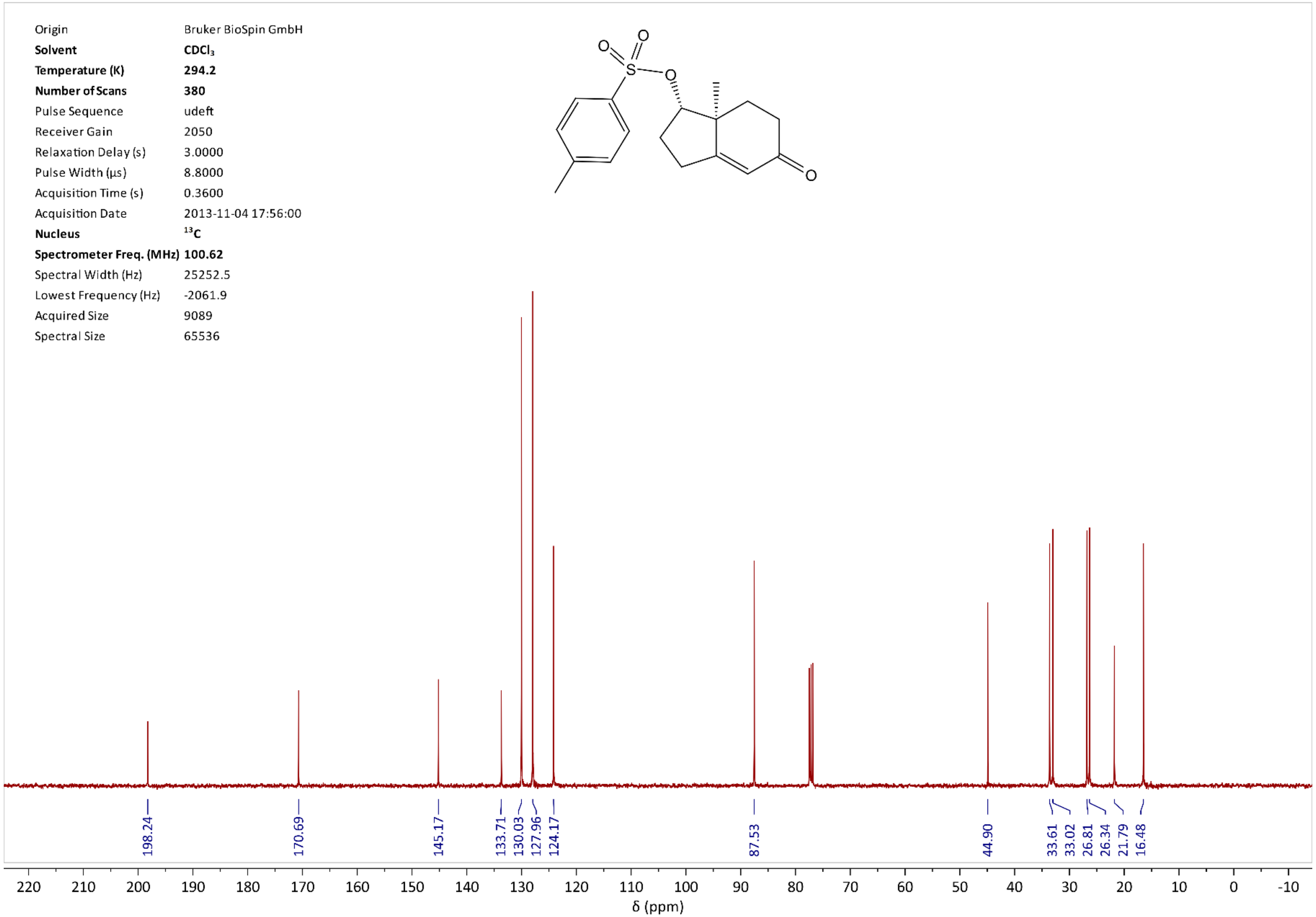

Tosylate acetal S18

^{1}H NMR Spectrum, $CDCl_3$, 400 MHz

Origin	Bruker BioSpin GmbH
**Solvent**	**$CDCl_3$**
**Temperature (K)**	**294.5**
**Number of Scans**	**32**
Pulse Sequence	zg30
Receiver Gain	228
Relaxation Delay (s)	1.5000
Pulse Width (μs)	9.5000
Acquisition Time (s)	1.9923
Acquisition Date	2013-11-18 22:27:00
**Nucleus**	**^{1}H**
**Spectrometer Freq. (MHz)**	**400.13**
Spectral Width (Hz)	8223.7
Lowest Frequency (Hz)	-1650.7
Acquired Size	16384
Spectral Size	65536

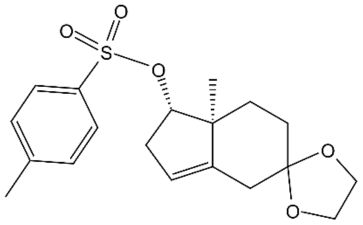

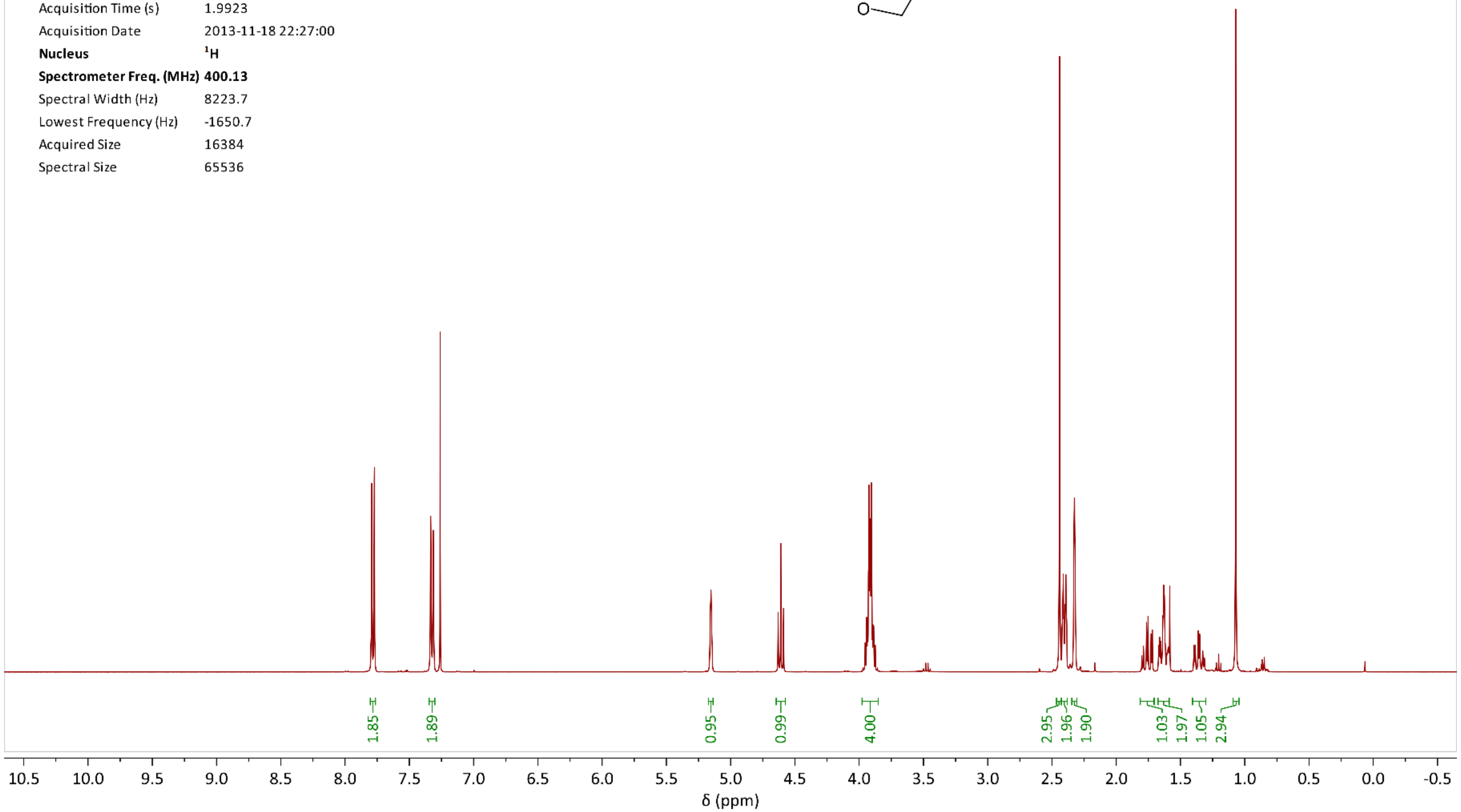

Tosylate acetal S18

^{13}C NMR Spectrum, $CDCl_3$, 101 MHz

Origin	Bruker BioSpin GmbH
**Solvent**	**$CDCl_3$**
**Temperature (K)**	**294.3**
**Number of Scans**	**380**
Pulse Sequence	udeft
Receiver Gain	2050
Relaxation Delay (s)	3.0000
Pulse Width (µs)	8.8000
Acquisition Time (s)	0.3600
Acquisition Date	2013-11-18 22:36:00
**Nucleus**	**^{13}C**
**Spectrometer Freq. (MHz)**	**100.62**
Spectral Width (Hz)	25252.5
Lowest Frequency (Hz)	-2048.9
Acquired Size	9089
Spectral Size	65536

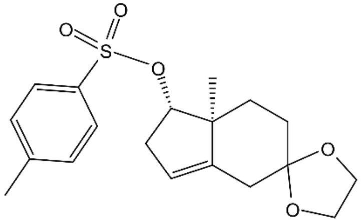

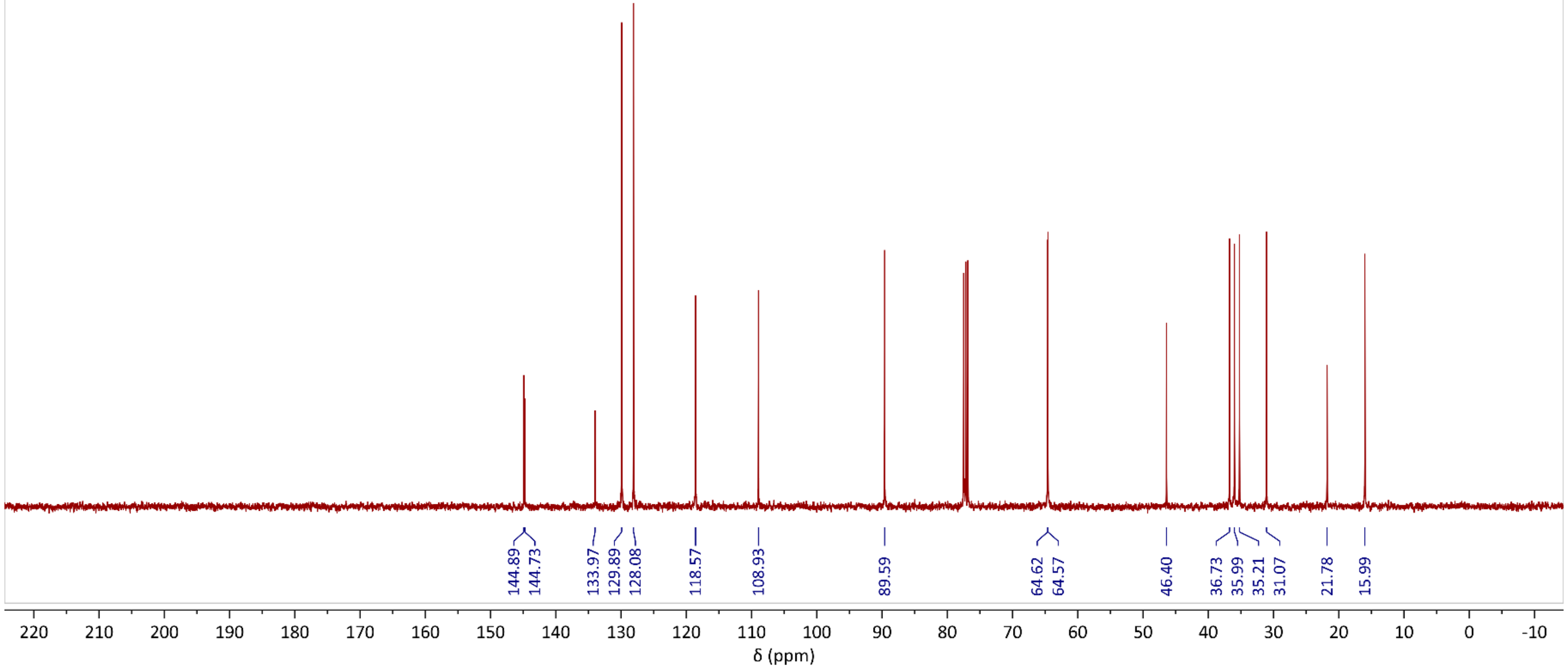

HPLC Data

Hajos-Parrish ketone 15

HPLC, Racemic sample, 20:80 MeOH:CO_2, 40 °C, 125 bar, 4 mL/min

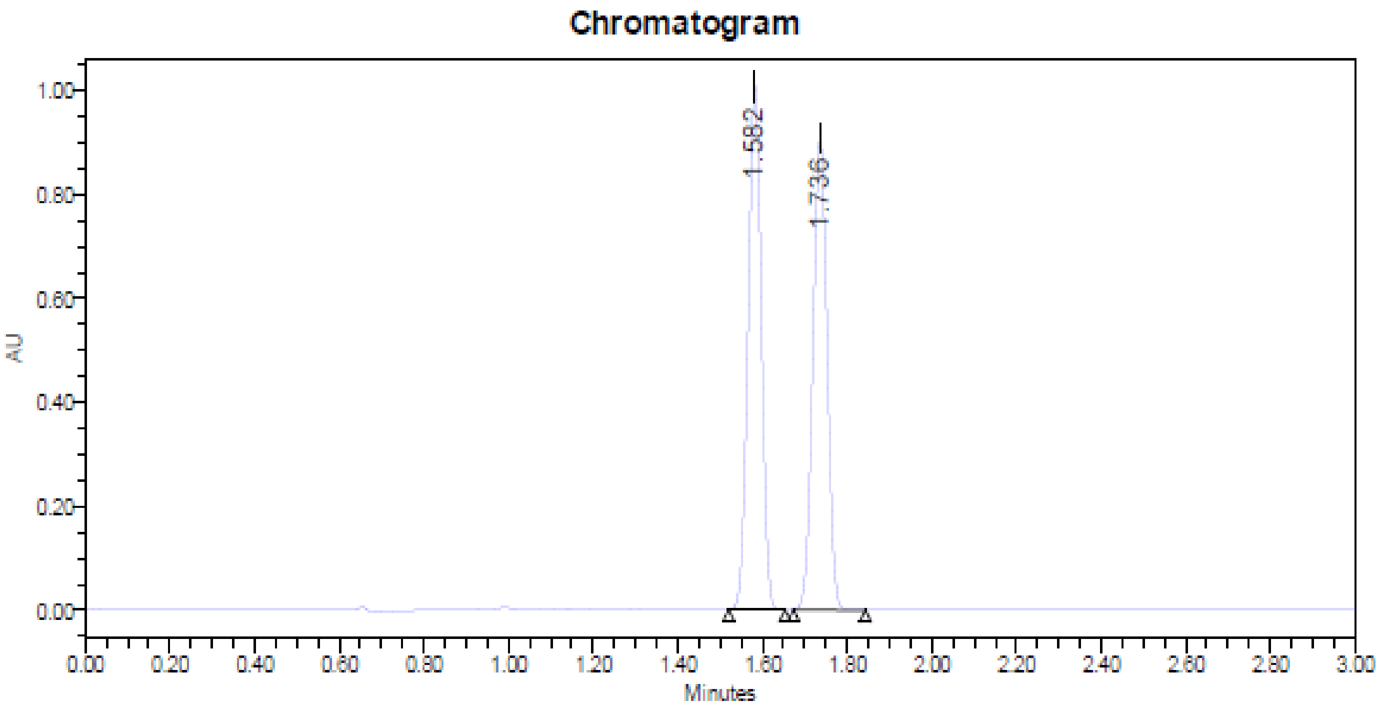

Peak Results

	Retention Time (min)	Area (µV*sec)	% Area	Width @ 50%
1	1.58	2201095	50.9	
2	1.74	2124599	49.1	

Chiral Purity Analysis Conditions;

Column Details	Lux C4 (4.6mm x 250mm, 5um)
Column Temperature	40 C
Flow Rate	4 mL/min
Detector Wavelength	210-400nm
Injection Volume	1.0 uL
BPR	125 BarG
Isocratic Conditions	20:80 MeOH:CO2

Hajos-Parrish ketone 15

HPLC, Asymmetric sample, 20:80 MeOH:CO_2, 40 °C, 125 bar, 4 mL/min

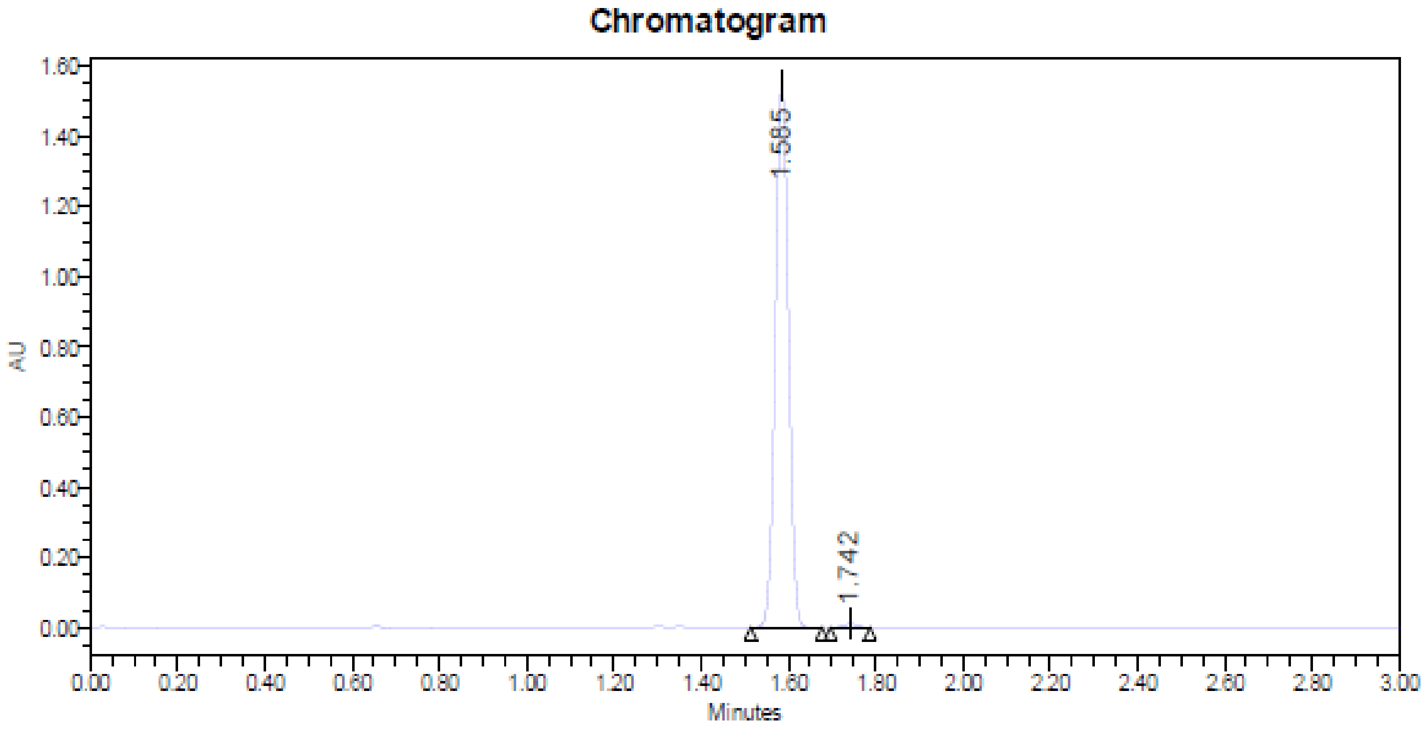

Peak Results

	Retention Time (min)	Area (µV*sec)	% Area	Width @ 50%
1	1.59	3229481	99.4	
2	1.74	20651	0.6	

Chiral Purity Analysis Conditions;

Column Details	Lux C4 (4.6mm x 250mm, 5um)
Column Temperature	40 C
Flow Rate	4 mL/min
Detector Wavelength	210-400nm
Injection Volume	1.0 uL
BPR	125 BarG
Isocratic Conditions	20:80 MeOH:CO2

X-ray Data

Crystal Data for **Alkyne 1,3-diol 36**: $C_{27}H_{42}O_3Si$ (M =442.69 g/mol): monoclinic, space group $P2_1$ (no. 4), a = 10.36850(10) Å, b = 8.35850(10) Å, c = 16.0378(3) Å, β = 105.130(2)°, V = 1341.74(3) Å^3, Z = 2, T = 100.00(10) K, μ(CuKα) = 0.945 mm^{-1}, $Dcalc$ = 1.096 g/cm^3, 25509 reflections measured (8.834° ≤ 2Θ ≤ 150.108°), 5429 unique (R_{int} = 0.0455, R_{sigma} = 0.0297) which were used in all calculations. The final R_1 was 0.0380 (I > 2σ(I)) and wR_2 was 0.1008 (all data).

The structure occupies a chiral space group and the Flack parameter is -0.04(2). It has thus been determined that the crystal contained a single enantiomer and that the chirality of C(1), C(4), C(5), C(9) and C(10) are all *R* and C(2) is *S*.

The hydroxyl hydrogen atoms bonded to O(1) and O(2) were located in the electron density and their positions and thermal parameters were refined freely. All other hydrogen atoms are fixed as riding models with the isotropic thermal parameters (U_{iso}) based on the U_{eq} of the parent atom.

Crystal Data for **Alkyne 1,3-diol 37:** $C_{27}H_{42}O_3Si$ (M =442.69 g/mol): orthorhombic, space group $P2_12_12_1$ (no. 19), a = 8.60590(10) Å, b = 13.05430(10) Å, c = 22.9303(3) Å, V = 2576.08(5) Å^3, Z = 4, T = 100.01(10) K, μ(CuKα) = 0.984 mm^{-1}, $Dcalc$ = 1.141 g/cm^3, 24967 reflections measured (7.71° ≤ 2Θ ≤ 149.198°), 5203 unique (R_{int} = 0.0279, R_{sigma} = 0.0193) which were used in all calculations. The final R_1 was 0.0262 (I > 2σ(I)) and wR_2 was 0.0674 (all data).

The structure occupies a chiral space group and the Flack parameter is 0.002(7). It has thus been determined that the crystal contained a single enantiomer and that the chirality of C(1), C(2), C(4), C(5), C(9) and C(10) are all *R*.

The hydroxyl hydrogen atoms bonded to O(1) and O(2) were located in the electron density and their positions and thermal parameters were refined freely. All other hydrogen atoms are fixed as riding models with the isotropic thermal parameters (U_{iso}) based on the U_{eq} of the parent atom.

Crystal Data for **Alkyne 1,3-diol 38**: $C_{27}H_{42}O_3Si$ (M =442.69 g/mol): orthorhombic, space group $P2_12_12_1$ (no. 19), a = 8.63340(10) Å, b = 13.2582(2) Å, c = 22.9906(3) Å, V = 2631.58(6) Å^3, Z = 4, T = 100.01(10) K, μ(CuKα) = 0.963 mm^{-1}, $Dcalc$ = 1.117 g/cm^3, 25208 reflections measured (7.69° ≤ 2Θ ≤ 148.924°), 5307 unique (R_{int} = 0.0315, R_{sigma} = 0.0211) which were used in all calculations. The final R_1 was 0.0271 (I > 2σ(I)) and wR_2 was 0.0705 (all data).

The structure occupies a chiral space group and the Flack parameter is 0.000(9). It has thus been determined that the crystal contained a single enantiomer and that the chirality of C(1), C(2), C(4), C(5), C(9) are all *R* and C(10) is *S*.
The hydroxyl hydrogen atoms bonded to O(1) and O(2) were located in the electron density and their positions and thermal parameters were refined freely. All other hydrogen atoms are fixed as riding models with the isotropic thermal parameters (U_{iso}) based on the U_{eq} of the parent atom.

Crystal Data for **Oxanorbornenone 70**: $C_{28}H_{48}O_3Si$ (*M* =460.75 g/mol): monoclinic, space group $P2_1$ (no. 4), a = 21.7412(3) Å, b = 8.28630(10) Å, c = 32.7876(4) Å, β = 107.3520(10)°, V = 5638.00(13) Å^3, Z = 8, T = 99.9(3) K, μ(CuKα) = 0.912 mm^{-1}, *Dcalc* = 1.086 g/cm^3, 104893 reflections measured (8.028° ≤ 2Θ ≤ 140.146°), 21345 unique (R_{int} = 0.0439, R_{sigma} = 0.0284) which were used in all calculations. The final R_1 was 0.0479 (I > 2σ(I)) and wR_2 was 0.1325 (all data).
The structure contains four crystallographically independent molecules.
The structure occupies a chiral space group with a refined Flack parameter of 0.007 (12). In all molecules in the structure C(3), C(3A), C(9A) in Molecule 1, C(23), C(23A), C(29A) in Molecule 2, C(43), C(43A), C(49A) in Molecule 3 and C(63), C(63A), C(69A) in Molecule 4 are all *R* while C(5) and C(8) in Molecule 1, C(25) and C(28) in Molecule 2, C(45) and C(48) in Molecule 3 and C(65) and C(68) in Molecule 4 are all *S.*
In Molecule 4, one of the isopropyl groups, C(90A)-C(92A) / C(90C)-C(92C) is disordered over two positions, at a refined percentage occupancy ratio of 50.9 (1.3) : 49.1 (1.3).
The hydrogen atoms were fixed as riding models with the isotropic thermal parameters (U_{iso}) based on the U_{eq} of the parent atom.

Crystal Data for **Oxanorbornenone 71**: $C_{28}H_{48}O_3Si$ (*M* =460.75 g/mol): monoclinic, space group $P2_1$ (no. 4), a = 15.9512(3) Å, b = 9.45430(10) Å, c = 18.5330(3) Å, β = 97.387(2)°, V = 2771.72(8) Å^3, Z = 4, T = 99.9(4) K, μ(CuKα) = 0.928 mm^{-1}, *Dcalc* = 1.104 g/cm^3, 52685 reflections measured (6.888° ≤ 2Θ ≤ 146.168°), 10943 unique (R_{int} = 0.0380, R_{sigma} = 0.0251) which were used in all calculations. The final R_1 was 0.0280 (I > 2σ(I)) and wR_2 was 0.0725 (all data).
The structure contains two crystallographically independent molecules. It occupies a chiral space group with a refined Flack parameter of -0.022 (7) such that in all molecules all chiral centres are *R*.
The hydrogen atoms were fixed as riding models with the isotropic thermal parameters (U_{iso}) based on the U_{eq} of the parent atom.

Crystal Data for **Tosylate acetal S18**: $C_{19}H_{24}O_5S$ (*M* =364.44 g/mol): monoclinic, space group C2/c (no. 15), *a* = 27.0507(2) Å, *b* = 5.81680(10) Å, *c* = 23.3541(2) Å, *β* = 106.5740(10)°, *V* = 3522.05(7) Å^3, *Z* = 8, *T* = 100.00(10) K, μ(Cu Kα) = 1.866 mm^{-1}, *Dcalc* = 1.375 g/cm^3, 59850 reflections measured (6.818° ≤ 2Θ ≤ 147.912°), 3572 unique (R_{int} = 0.0266, R_{sigma} = 0.0082) which were used in all calculations. The final R_1 was 0.0297 (I > 2σ(I)) and wR_2 was 0.0797 (all data).
The space group is centrosymmetric such that in half of the molecules in the unit cell C(1) and C(8) are both *S* and in the other half of the molecules C(1) and C(8) are both *R.*
The hydrogen atoms were fixed as riding models with the isotropic thermal parameters (U_{iso}) based on the U_{eq} of the parent atom.

Crystal Data for **Tosylate S17**: $C_{17}H_{20}O_4S$ (*M* =320.39 g/mol): orthorhombic, space group Pna2$_1$ (no. 33), *a* = 20.0274(7) Å, *b* = 7.3776(2) Å, *c* = 10.7862(3) Å, *V* = 1593.71(8) Å^3, *Z* = 4, *T* = 100.00(10) K, μ(Cu Kα) = 1.939 mm^{-1}, *Dcalc* = 1.335 g/cm^3, 14121 reflections measured (12.788° ≤ 2Θ ≤ 148.888°), 3188 unique (R_{int} = 0.0458, R_{sigma} = 0.0289) which were used in all calculations. The final R_1 was 0.0429 (I > 2σ(I)) and wR_2 was 0.1143 (all data).
The structure occupies a chiral space group and the Flack parameter is 0.001 (12). It has thus been determined that the crystal contained a single enantiomer and that the chirality of C(1) and C(8) are both *S*.
The hydrogen atoms were fixed as riding models with the isotropic thermal parameters (U_{iso}) based on the U_{eq} of the parent atom.

The datasets were measured on an Agilent SuperNova diffractometer using an Atlas detector. The data collections were driven and processed and absorption corrections were applied using CrysAlisPro.[S1] Using Olex2[6], the structures of **Tosylate S17** and **Tosylate acetal S18** were solved using ShelXS[S2] and the remaining structures were solved using ShelXT,[7] and all structures were refined by a full-matrix least-squares procedure on F^2 in ShelXL.[8]
The CIFs for the crystal structures of **Alkyne 1,3-diol 36**, **Alkyne 1,3-diol 37**, **Alkyne 1,3-diol 38**, **Oxanorbornenone 70**, o**xanorbornenone 71**, **Tosylate acetal S18** and **Tosylate S17** have been deposited with the CCDC and have been given the deposition numbers CCDC 2193399 to CCDC 2193405 respectively.

[S1] CrysAlisPro, Agilent Technologies, Version 1.171.37.35, **2014** and Version 1.171.39.46, **2018**.
[S2] G. M. Sheldrick, *Acta Cryst.* **2008**, *A64*, 112-122.

Alkyne 1,3-diol 36

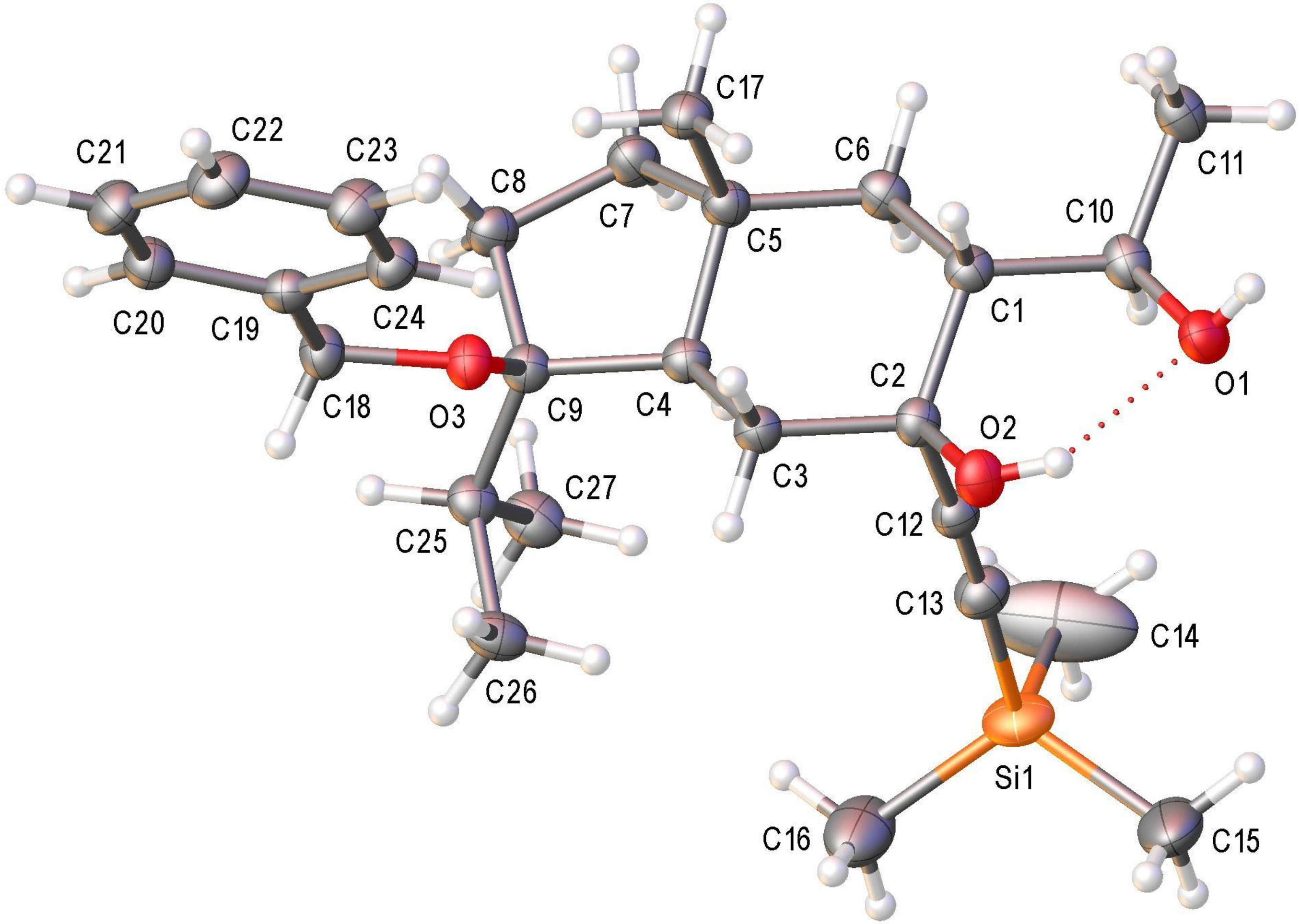

Crystal structure of **Alkyne 1,3-diol 36** with ellipsoids drawn at the 50 % probability level. A single crystal was obtained by the slow evaporation of Et_2O.

Alkyne 1,3-diol 37

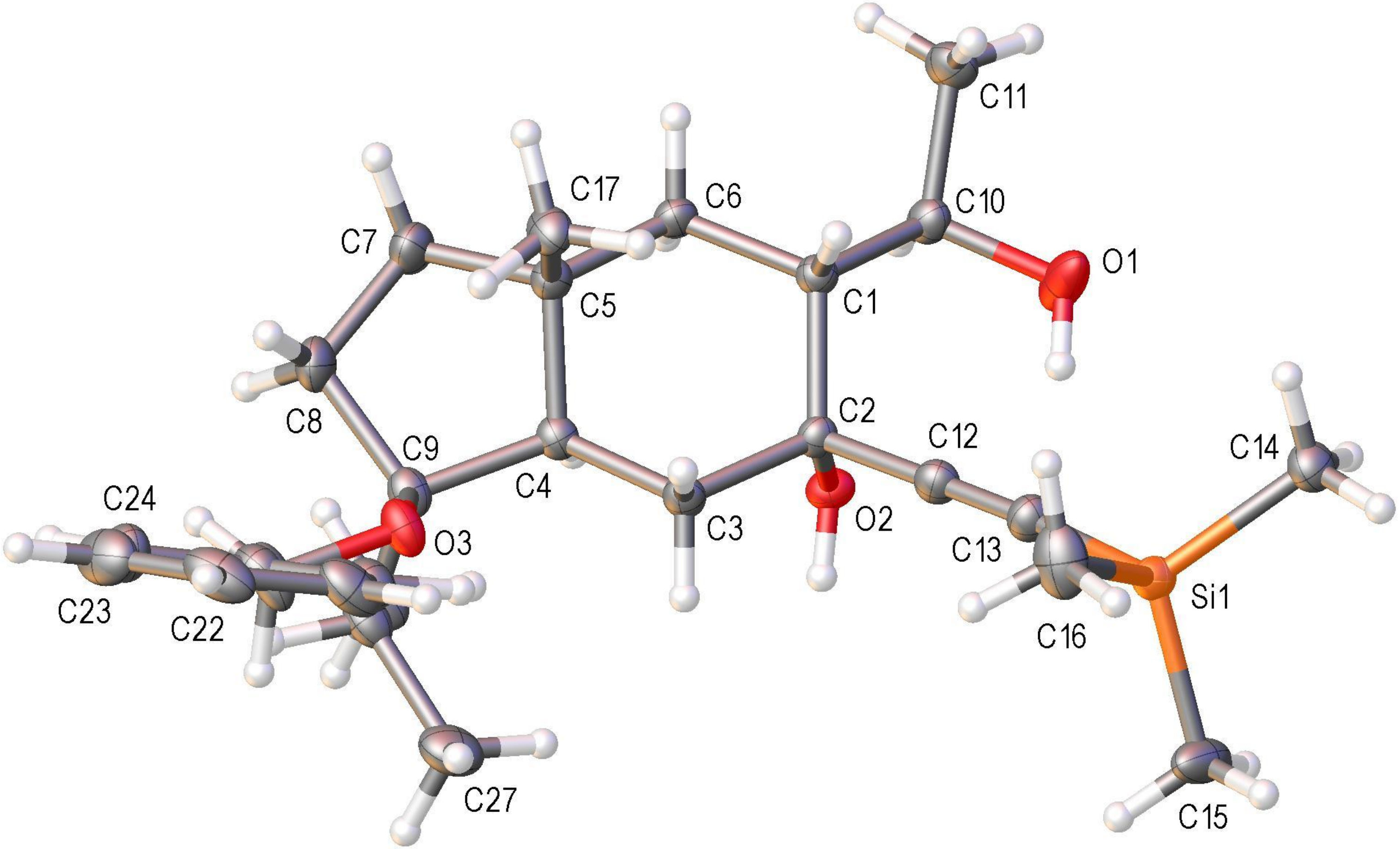

Crystal structure of **Alkyne 1,3-diol 37** with ellipsoids drawn at the 50 % probability level. A single crystal was obtained by the slow evaporation of Et_2O.

Alkyne 1,3-diol 38

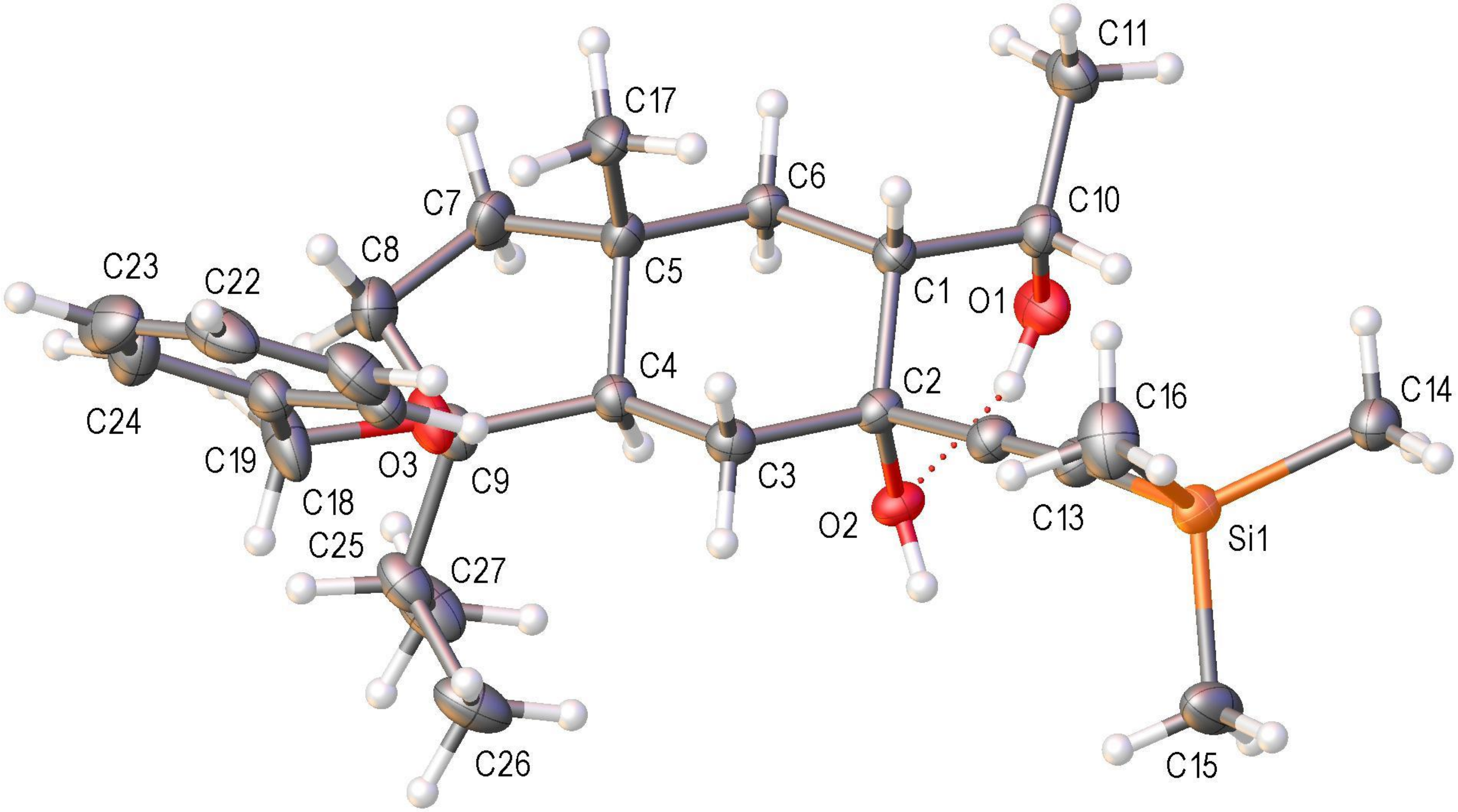

Crystal structure of **Alkyne 1,3-diol 38** with ellipsoids drawn at the 50 % probability level. A single crystal was obtained by the slow evaporation of Et_2O.

Oxanorbornenone 70

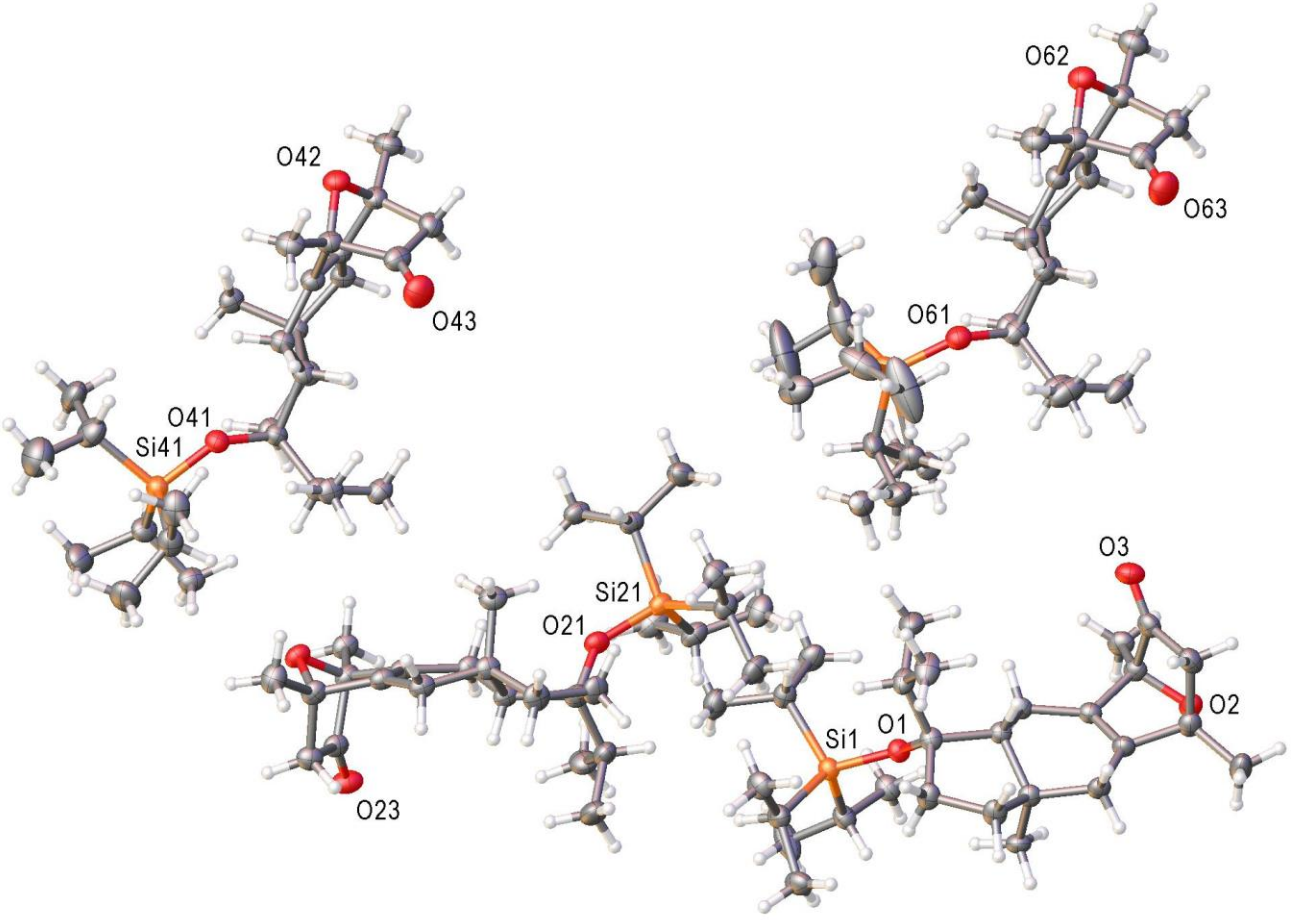

Crystal structure of **Oxanorbornenone 70** with ellipsoids drawn at the 50 % probability level. The structure contains four crystallographically independent molecules. In Molecule 4, one of the isopropyl groups is disordered over two positions, at a refined percentage occupancy ratio of 50.9 (1.3) : 49.1 (1.3). A single crystal was obtained by the slow evaporation of acetone.

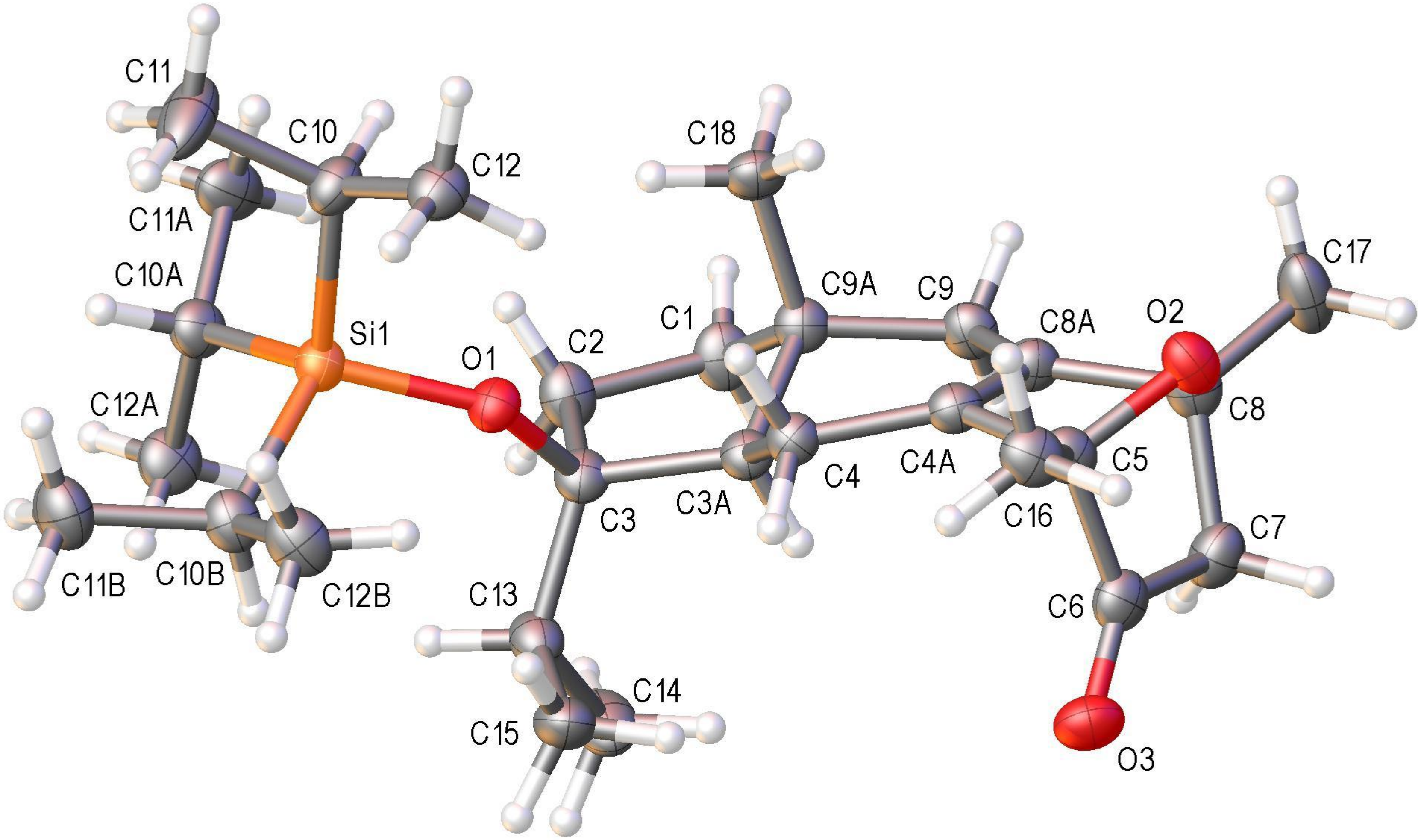

Crystal structure of Molecule 1 of **Oxanorbornenone 70** with ellipsoids drawn at the 50 % probability level. The structure contains four crystallographically independent molecules of which only one is shown.

Oxanorbornenone 71

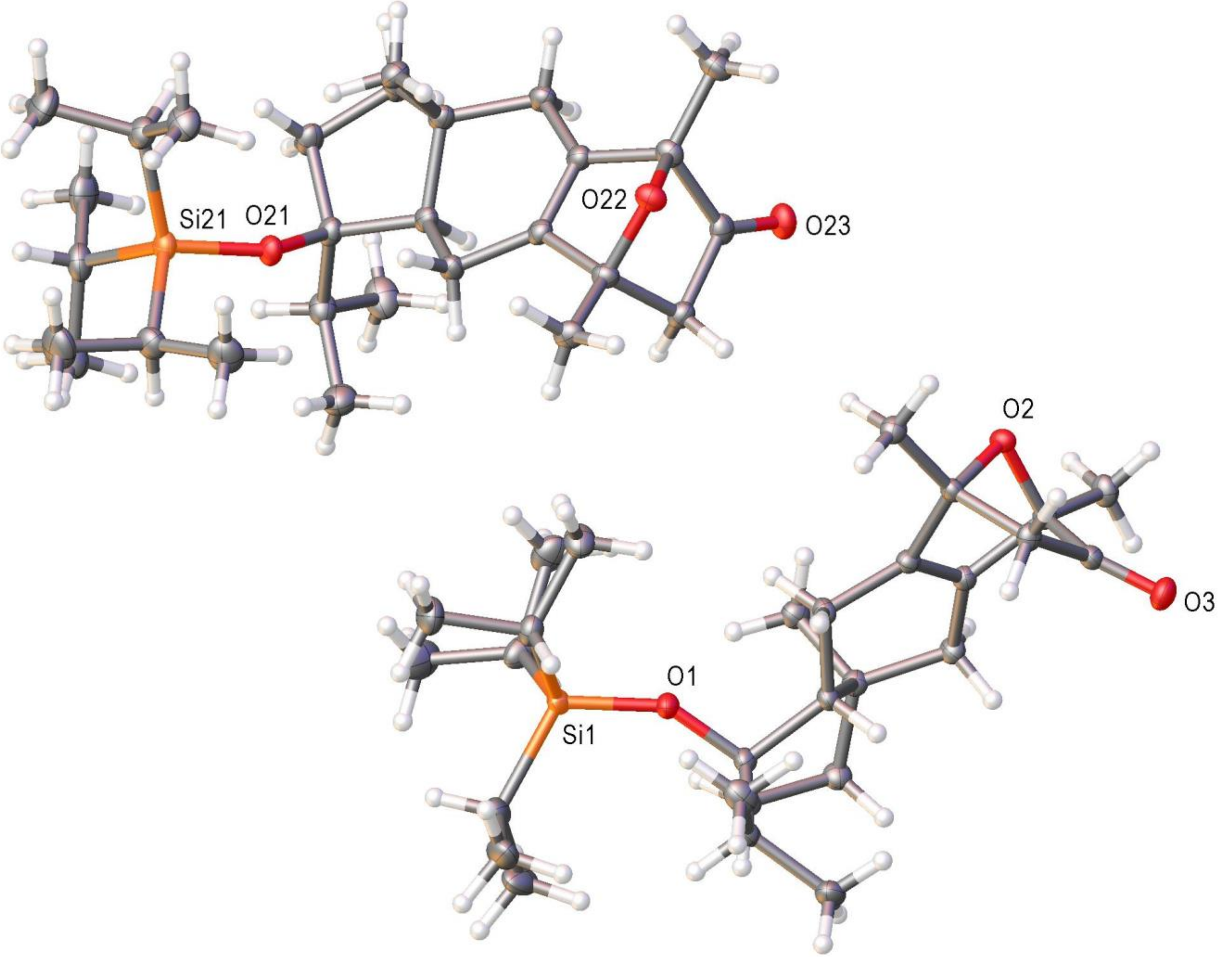

Crystal structure of **Oxanorbornenone 71** with ellipsoids drawn at the 50 % probability level. The structure contains two crystallographically independent molecules. A single crystal was obtained by the slow evaporation of acetone.

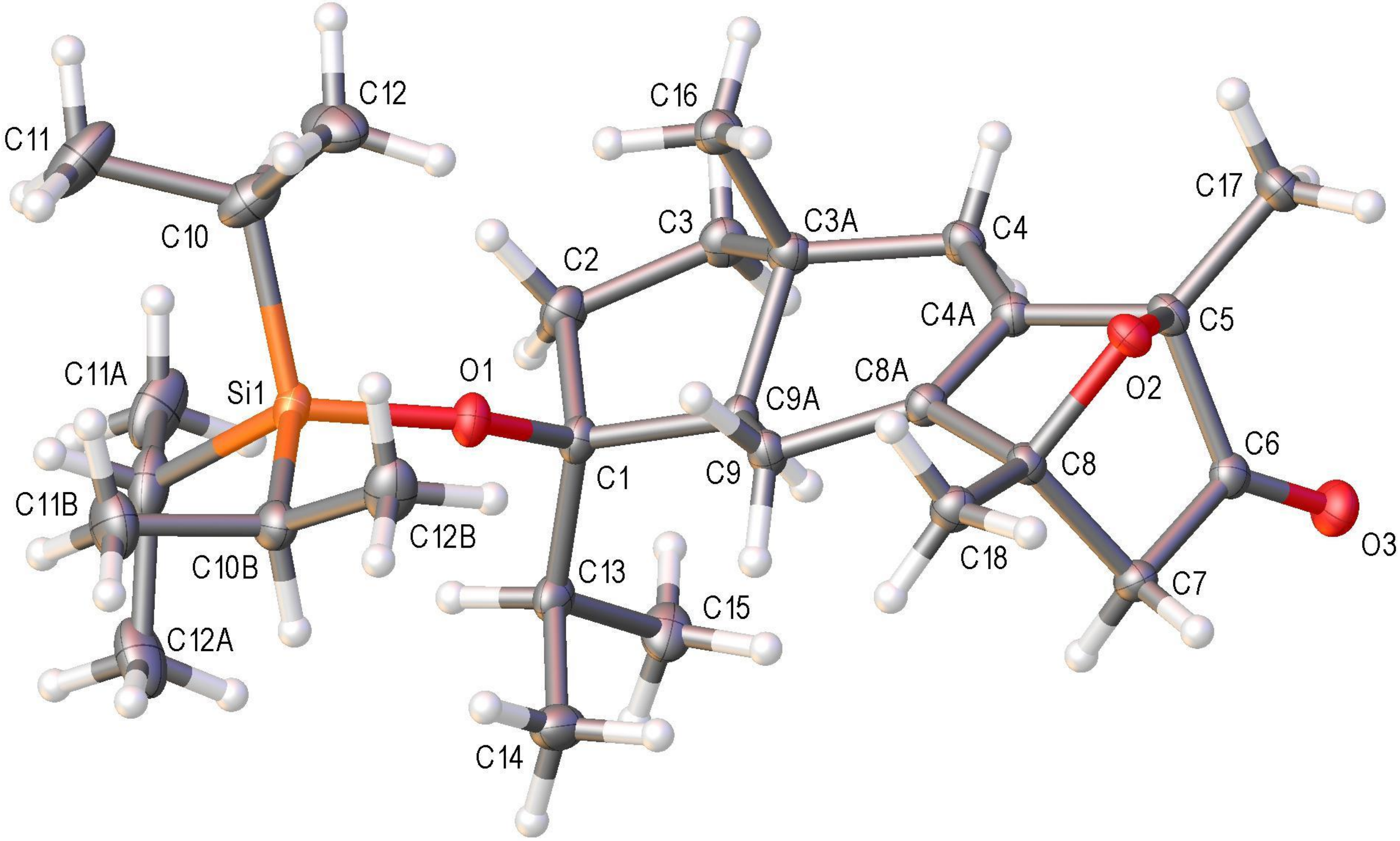

Crystal structure of Molecule 1 of **Oxanorbonenone 71** with ellipsoids drawn at the 50 % probability level. The structure contains two crystallographically independent molecules of which only one is shown.

Tosylate S17

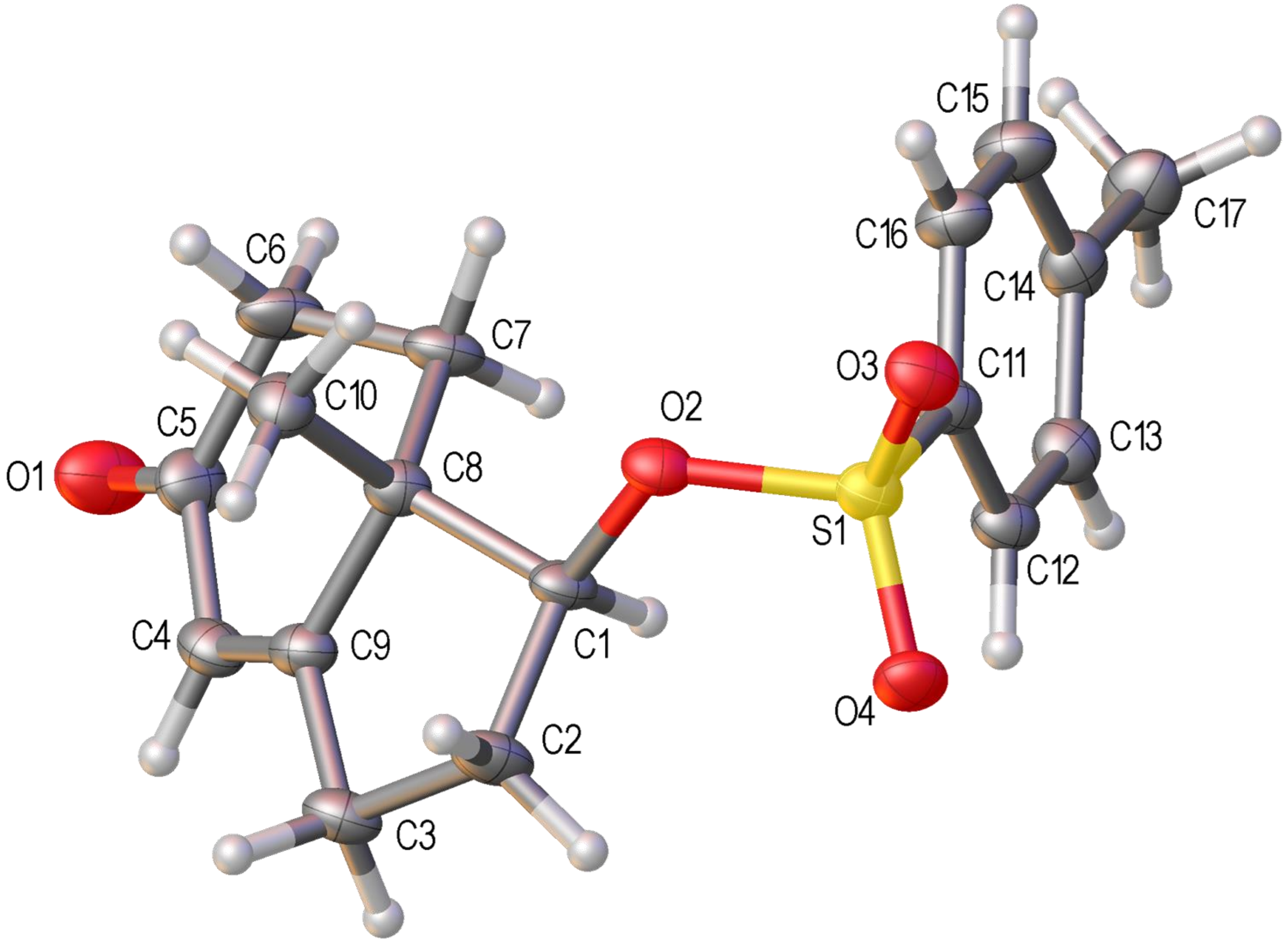

Crystal structure of **Tosylate S17** with ellipsoids drawn at the 50 % probability level.

Tosylate acetal S18

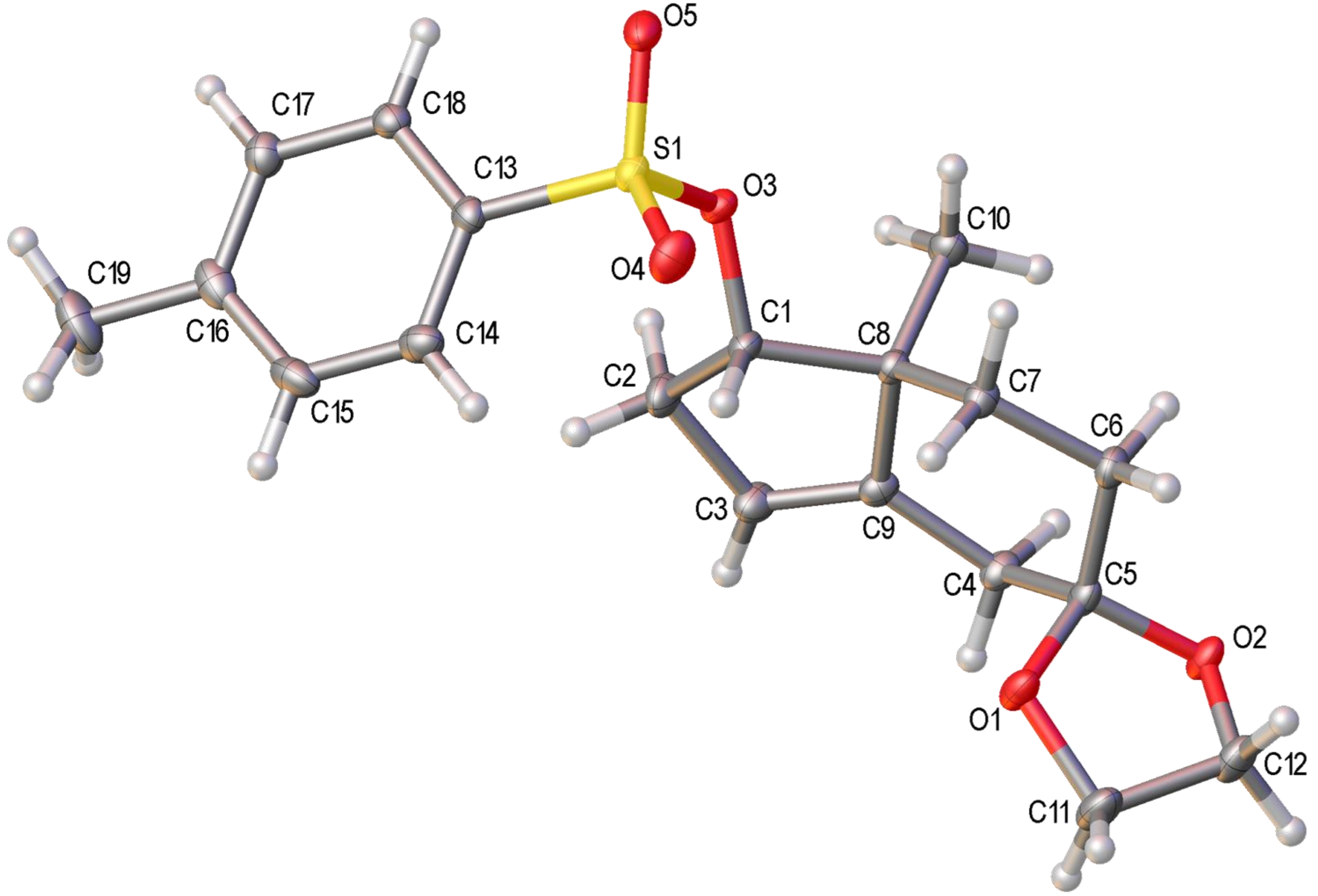

Crystal structure of **Tosylate acetal S18** with ellipsoids drawn at the 50 % probability level. Crystal obtained from slow evaporation of CH_2Cl_2/hexane.

References

[1] H.-S. Lin, L. A. Paquette, *Synth. Commun.* **1994**, *24*, 2503–2506.
[2] L. Duhamel, *J. Organomet. Chem.* **1993**, *448*, 1–3.
[3] V. K. Aggarwal, Z. Gültekin, R. S. Grainger, H. Adams, P. L. Spargo, *J. Chem. Soc., Perkin Trans. 1* **1998**, 2771–2781.
[4] D. B. G. Williams, M. Lawton, *J. Org. Chem.* **2010**, *75*, 8351–8354.
[5] G. R. Fulmer, A. J. M. Miller, N. H. Sherden, H. E. Gottlieb, A. Nudelman, B. M. Stoltz, J. E. Bercaw, K. I. Goldberg, *Organometallics* **2010**, *29*, 2176–2179.
[6] O. V. Dolomanov, L. J. Bourhis, R. J. Gildea, J. A. K. Howard, H. Puschmann, *J. Appl. Crystallogr.* **2009**, *42*, 339–341.
[7]G. M. Sheldrick, *Acta Crystallogr. Sect. A Found. Crystallogr.* **2015**, *71*, 3–8.
[8] G. M. Sheldrick, *Acta Crystallogr. Sect. C Struct. Chem.* **2015**, *71*, 3–8.
[9] B. Defaut, T. B. Parsons, N. Spencer, L. Male, B. M. Kariuki, R. S. Grainger, *Org. Biomol. Chem.* **2012**, *10*, 4926–4932.
[10] C. L. Hugelshofer, T. Magauer, *J. Am. Chem. Soc.* **2016**, *138*, 6420–6423.
[11] T. Imamoto, Y. Sugiura, N. Takiyama, *Tetrahedron Lett.* **1984**, *25*, 4233–4236.
[12] Z. G. Hajos, D. R. Parrish, *Org. Synth.* **1985**, *63*, 26.
[13] J. N. Gardner, B. A. Anderson, E. P. Oliveto, *J. Org. Chem.* **1969**, *34*, 107–112.
[14] R. A. Micheli, Z. G. Hajos, N. Cohen, D. R. Parrish, L. A. Portland, W. Sciamanna, M. A. Scott, P. A. Wehrli, *J. Org. Chem.* **1975**, *40*, 675–681.
[15] H. Hioki, T. Hashimoto, M. Kodama, *Tetrahedron Asymmetry* **2000**, *11*, 829–834.
[16] S. Mahapatra, R. G. Carter, *J. Am. Chem. Soc.* **2013**, *135*, 10792–10803.
[17] J. S. Yadav, V. Rajender, *Eur. J. Org. Chem.* **2010**, *7*, 2148–2156.
[18] H. Ohmiya, H. Yorimitsu, K. Oshima, *J. Am. Chem. Soc.* **2006**, *128*, 1886–1889.
[19] P. Kraft, C. Weymuth, C. Nussbaumer, *Eur. J. Org. Chem.* **2006**, 1403–1412.
[20] S. García-Rubín, C. González-Rodríguez, C. García-Yebra, J. A. Varela, M. A. Esteruelas, C. Saá, *Angew. Chem. Int. Ed.* **2014**, *53*, 1841–1844.
[21] K. Sonogashira, Y. Tohda, N. Hagihara, *Tetrahedron Lett.* **1975**, *16*, 4467–4470.
[22] B. Gabriele, G. Salerno, E. Lauria, *J. Org. Chem.* **1999**, *64*, 7687–7692.
[23] M. Vidal-Pascual, C. Martinez-Lamenca, H. M. R. Hoffmann, *Org. Synth.* **2006**, *83*, 61–69.
[24] J. Wittenberg, W. Beil, H. M. R. Hoffmann, *Tetrahedron Lett.* **1998**, *39*, 8259–8262.
[25] Z. Arnold, A. Holy, *Collect. Czechoslov. Chem. Commun.* **1961**, *26*, 3059–3073.
[26] S. J. Mckerrall, L. Jørgensen, C. A. Kuttru, F. Ungeheuer, P. S. Baran, *J. Am. Chem. Soc.* **2014**, *136*, 5799–5810.
[27] M. Utsugi, M. Miyano, M. Nakada, *Org. Lett.* **2006**, *8*, 2973–2976.
[28] M. Harmata, U. Sharma, *Org. Lett.* **2000**, *2*, 2703–2705.
[29] K. V. Rajendran, D. J. Carr, D. G. Gilheany, *Tetrahedron Lett.* **2011**, *52*, 7113–7115.
[30] B. Föhlisch, E. Gehrlach, R. Herter, *Angew. Chemie Int. Ed. Engl.* **1982**, *21*, 137–137.
[31] S. W. Tobey, R. West, *J. Am. Chem. Soc.* **1966**, *88*, 2481–2488.
[32] R. S. Oruguntу, D. L. Wright, M. A. Battiste, K. A. Abboud, *Org. Lett.* **2002**, *4*, 1997–2000.
[33] M. Fetizon, M. Jurion, *J. Chem. Soc., Chem. Commun.* **1972**, 382–383.
[34] V. K. Aggarwal, J. Drabowicz, R. S. Grainger, Z. Gultekin, M. Lightowler, P. L. Spargo, *J. Org. Chem.* **1995**, *60*, 4962–4963.
[35] K. Schank, A. Weber, *Chem. Ber.* **1972**, *105*, 2188–2196.
[36] C. B. W. Stark, S. Pierau, R. Wartchow, H. M. R. Hoffmann, *Chem. Eur. J.* **2000**, *6*, 684–691.
[37] S. J. Foster, C. W. Rees, *J. Chem. Soc., Perkin Trans. 1* **1985**, 719–722.
